# Evaluating alignment and variant-calling software for mutation identification in *C*. *elegans* by whole-genome sequencing

**DOI:** 10.1371/journal.pone.0174446

**Published:** 2017-03-23

**Authors:** Harold E. Smith, Sijung Yun

**Affiliations:** National Institute of Diabetes and Digestive and Kidney Diseases, National Institutes of Health, Bethesda, Maryland, United States of America; Texas A&M University Health Sciences Center, UNITED STATES

## Abstract

Whole-genome sequencing is a powerful tool for analyzing genetic variation on a global scale. One particularly useful application is the identification of mutations obtained by classical phenotypic screens in model species. Sequence data from the mutant strain is aligned to the reference genome, and then variants are called to generate a list of candidate alleles. A number of software pipelines for mutation identification have been targeted to *C*. *elegans*, with particular emphasis on ease of use, incorporation of mapping strain data, subtraction of background variants, and similar criteria. Although success is predicated upon the sensitive and accurate detection of candidate alleles, relatively little effort has been invested in evaluating the underlying software components that are required for mutation identification. Therefore, we have benchmarked a number of commonly used tools for sequence alignment and variant calling, in all pair-wise combinations, against both simulated and actual datasets. We compared the accuracy of those pipelines for mutation identification in *C*. *elegans*, and found that the combination of BBMap for alignment plus FreeBayes for variant calling offers the most robust performance.

## Introduction

Next-generation sequencing (NGS) has transformed our ability to study genetic variation. Entire genomes can be sequenced in a matter of days at relatively low cost. Comparison of whole-genome data from two or more samples allows the global determination of sequence differences between them. The technology has been applied to species from viruses to humans, and has proven particularly useful for identifying causative mutations obtained in forward genetic screens.

Mutation identification in model organisms typically begins with the alignment of sequence data to a reference genome, followed by the generation of consensus variant calls, and finally the application of data filters dictated by the species, genetic background, and pedigree. The number of software tools available for each of those steps, coupled with their complexity, has slowed the adoption of NGS by many researchers, who may find the analysis daunting. To address that challenge, a variety of integrated variant detection pipelines have been developed with an eye toward ease of use. The integrated pipelines offer attractive features beyond the simple identification of variants: filtering to remove strain-specific variants and common sequencing artifacts, determination of candidate intervals by polymorphism mapping, and/or incorporation of annotations to identify non-synonymous mutations. Several of those pipelines have been tailored to *Caenorhabditis elegans*, including MAQGene [1] CloudMap [2], and MiModD [3] The latter two have been integrated into the popular Galaxy platform [4–6], which provides users a browser-accessible graphical interface. For a more detailed description of those tools, the reader is directed to a recent WormMethod review [7].

Accurate detection of candidate variants is a necessary prerequisite for success in identifying mutations. However, software for sequence alignment and variant calling is under continuous development, and it is seems probable that some of the older components incorporated into those integrated pipelines are not optimal. For example, MAQGene is based on MAQ [8], a combined aligner and variant caller. However, that early-generation tool is unable to align gapped sequences and, therefore, fails to detect most insertions and deletions. Likewise, CloudMap utilizes the GATK UnifiedGenotyper [9] for variant calling, yet the GATK developers recommend the newer HaplotypeCaller as a superior alternative [10–11]. Note that Galaxy-based workflows such as CloudMap and MiModD can be edited easily to incorporate alternative software components, so the added functionality of those pipelines does not preclude the use of improved tools for alignment and variant-calling.

An additional consideration is that much of the NGS analysis software is developed for and benchmarked against human data. However, the human genome differs significantly from the worm in terms of size, degree of repetitive sequences, and zygosity of variant alleles. Those properties impose constraints on algorithm design and influence the heuristics employed to address them. Similarly, best-practices guidelines for variant analysis are usually developed with the human genome in mind, and may not be ideal for other species. Finally, there is the separate issue of software compatibility. Despite the widespread adoption of standard formats, such as SAM/BAM and VCF, differences between format versions or the inclusion of optional field data can produce idiosyncratic interactions between specific software components.

To address those concerns, we performed variant detection with several data sets derived from *C*. *elegans*. We produced simulated reads that replicate the type of data typical for mutation identification in worms. We evaluated a number of sequence aligners and variant callers, in all pair-wise combinations, for sensitivity and selectivity in the detection of known variants. We also characterized the software using actual genome sequence data from strains that contain a large number of polymorphisms, validating the variant calls by prior annotation. We observed that the results were affected by the variant type (SNP, insertion, or deletion), zygosity, and length of insertion or deletion. Although no single pair of components was the best for every category of variant, we have determined that BBMap plus FreeBayes offers robust performance across all of the data that were tested.

## Materials and methods

The following software were used for sequence alignment: BBMap [12], BFAST [13], Bowtie 2 [14], BWA [15] and NovoAlign [16] The software used for variant calling were: FreeBayes [17], GATK HaplotypeCaller [18], SAMtools/BCFtools [19], and VarScan 2 [20]. Additional software utilities included BEDTools [21], Picard [22] VCFLIB [23], and VCFtools [24]. Each software package was installed per the developers’ instructions. Version information and commands for software operation are listed in the Supporting Information (S1 and S2 Files, respectively).

Simulated data were produced using the ‘randomreads’ function of BBMap [12]. Perfect-match data were generated from the *C*. *elegans* reference genome version WS250 (http://www.wormbase.org) with no errors and high base-quality scores (≥35). Genome coverage was calculated after filtering to remove reads with map quality scores ≤3 (~50% likelihood of mismapping). Simulated ethyl methanesulfonate (EMS) mutagenesis-type data were generated from a modified version of the reference genome into which mutations had been introduced by a custom script. The modified reference contained the following mutations (all listed in S1 Table): 1000 homozygous single-nucleotide polymorphisms (SNPs), 1000 heterozygous SNPs, 50 homozygous small (1-15-bp) insertions, 50 heterozygous small insertions, 50 homozygous small (1-15-bp) deletions, 50 heterozygous small deletions, and six each (one per chromosome) homozygous large (100-500-bp) deletions, transposon insertions, and complex insertions/deletions (indels). Single-end 50-bp reads were generated with an error distribution of 0.2% random SNPs and 0.002% random indels. Simulated *Escherichia coli* data were generated from reference MG1655 [25] with the same error distribution.

Sequence data for Hawaiian SNPs have been described previously [26] and are available at the NCBI Sequence Read Archive (BioProject accession number PRJNA305991). For Hawaiian SNP-calling sensitivity, data were combined from wild-type strain N2 Bristol and polymorphic Hawaiian strain CB4856 [27]. Libraries from each strain were constructed using the TruSeq library prep (Illumina, San Diego, CA), and single-end 50-bp sequencing performed on a HiSeq 2500 instrument (Illumina, San Diego, CA). The two libraries were sequenced independently and the data (20-fold total coverage) combined at two different ratios to represent heterozygous (50% Hawaiian) or low-frequency (5% Hawaiian) SNPs. Prior work identified a subset of 103,346 annotated Hawaiian SNPs from genome reference version WS220 suitable for mapping (Haw filtered list) [2]. Conversion to genome reference version WS250 removed twenty SNPs, leaving 103,326 high-quality SNPs (S2 Table); those were used to validate variant calls obtained from the sequence data. One of the variant callers (FreeBayes) classified SNPs in close proximity as multiple nucleotide polymorphisms (MNPs) or complex variants; those were separated into constituent SNPs with the ‘vcfallelicprimitives’ function of VCFLIB [23] prior to Hawaiian SNP annotation. For the Hawaiian mapping plot example, variants were called with BBMap plus FreeBayes using default parameters (minimum 20% variant frequency supported by at least two reads) or declared variables (1% frequency, one supporting read). Hawaiian SNP frequencies with LOESS regressions were plotted against chromosome position with R [28]. Commands for Hawaiian SNP sensitivity detection are available as S3 File.

## Results and discussion

Readers not interested in the details of the comparative analyses can skip to the last section for recommendations on the optimal workflow for mutation identification in *C*. *elegans*.

### Software selection

Our objective was to identify software components and parameters that are optimally suited to the task of mutation identification in *C*. *elegans*. Given the number of options for sequence alignment and variant calling, it was not feasible to undertake a comprehensive survey of all software. Thus, we decided to limit our analysis to open-source software tools that are freely available and have been widely cited in the literature. We selected five alignment tools that were designed specifically for short-read DNA sequencing (DNA-Seq), as opposed to RNA-Seq or genome-to-genome alignment, and four tools for variant calling. We acknowledge that our choices are limited and somewhat arbitrary, and anticipate the availability of superior software. Therefore, we have made our test data available (see Materials and methods) so that the community can evaluate other tools independently. Although we do not include operational metrics (memory requirements and run times), each of the selected software tools can be run on a moderately powerful computer (four cores, 16GB RAM) in a span of hours.

### Perfect-match data

Before proceeding with variant detection, we wanted to address two questions regarding baseline performance of the analysis: 1) How much of the genome is accessible by alignment? 2) How much data is required for comprehensive coverage? To tackle those questions, we evaluated the five aligners using perfect-match data (i.e., with no mutations or errors).

A portion of the *C*. *elegans* genome is repetitive and therefore refractory to mutation identification. The short reads (typically 50-250-bp) generated by most current NGS platforms affect variant detection within repeated sequences in two ways. First, reads derived from identical repeats that are longer than the read length cannot be uniquely aligned to the reference genome. Depending upon the aligner, those can be flagged as multi-mapping reads without positional information, assigned to the first best matching locus, or randomly assigned to one of the matching loci. In none of those cases is the alignment suitable for variant calling. Second, reads derived from nearly identical repeats produce multiple alignments that differ minimally and are assigned low-confidence mapping quality scores. Those values are incorporated into the variant-calling algorithms, producing variant calls with low quality scores that are often filtered as probable false-positives. Consequently, repeated regions of the genome are largely resistant to variant detection by short-read sequencing.

As a practical consideration, the inaccessibility of some repeat sequences has minimal impact on mutation identification. A large fraction is derived from canonical repetitive elements, such as transposons, where mutation within the repeat is unlikely to produce a phenotype. And, while mobilization of a repetitive element and insertion within a gene can disrupt its function, that mechanism is quiescent in the standard *C*. *elegans* laboratory strain. However, some repeats are derived from the duplication of coding sequences. Paralogs, pseudogenes, and conserved protein-coding domains represent potential sources of sequence duplication that might preclude variant detection in some genes. The impact of sequence duplication is not merely hypothetical: the *top-2(it7)* mutation cannot be identified by some variant-calling pipelines due to sequence similarity between *top-2* and its paralog *cin-4* [29].

To our knowledge, the degree of repeated sequence defined by short-read alignment has not been reported for *C*. *elegans*, so we performed that analysis. Twenty-fold genome coverage provides sufficient data to detect 99% of variants in both worms [30] and humans [31], so we began our analysis at that depth. We generated a perfect-match data set of 40 million 50-bp single-end reads from the 100 Mb *C*. *elegans* reference genome, and performed alignment with each of the five selected aligners using default parameters. We determined the percentage of reads that were unmapped or had low mapping quality scores, an indicator of possible mismapping. Those numbers were similar for all of the aligners, ranging from 6.49–7.20% of the total (Table 1). After filtering to remove those reads, we examined the fraction of the genome that was uncovered.

**Table 1:** Perfect-match SE-50bp 20-fold genomes, mapping and genome coverage.

Aligner	Unmapped/low map quality^a^	Uncovered genome^b^	Uncovered CDS (number of genes)^c^
BBMap	7.04%	5.29%	3.00% (2,089)
BFAST	7.20%	5.40%	3.06% (2,134)
Bowtie	6.49%	4.86%	2.96% (2,067)
BWA	6.50%	4.87%	2.96% (2,067)
Novoalign	6.49%	4.86%	2.96% (2,067)

^a^The percentage of reads with map quality scores ≤ 3.

^b^The percentage of nucleotides with read depth coverage < 3.

^c^The percentage of coding sequence in the genome that is uncovered, and the number of genes that contain uncovered coding sequence. Total coding sequence, 25,460,976 bases. Total number of genes, 20,538.

A minimum coverage depth of three independent reads is a reasonable guideline for variant calling, to limit false-positive calls that arise from the combination of low coverage plus sequencing errors, so we used that threshold to discriminate covered from uncovered regions. The percentage of uncovered genome (4.86–5.40%) varied slightly by aligner and correlated with the degree of low-confidence mapping (Table 1). Since the overwhelming majority of mutations that yield phenotypes lie within protein coding sequences, we determined the overlap between the uncovered regions and exons. We found that 2.96–3.06% of total exonic sequences were not covered, encompassed by 2,067–2,134 genes (Table 1). Thus, of the 20,538 *C*. *elegans* genes, ~10% contain some sequences that are not accessible by short-read alignment.

The observed lack of coverage might arise from an insufficient amount of data rather than the presence of repetitive sequences in the genome. However, an increase in the data from 20-fold to 50-fold genome coverage produced only a modest increase (~0.3%) in the covered fraction of the genome and correspondingly little improvement in gene coverage (Table 2). Furthermore, the 50-fold-coverage data set represents an independent random sampling of the genome. If lack of coverage were a consequence of insufficient data, then the uncovered regions of the genome would differ between the two data sets. Instead, the uncovered segments largely corresponded (>99% overlap between the 50-fold and 20-fold data sets; in S4 File). We conclude that ~5% of the genome (and ~3% of coding sequence) is inaccessible by single-end 50-bp sequence data, and that 20-fold coverage is sufficient for the accessible fraction.

**Table 2:** Percentage of uncovered sequence using various perfect-match data sets^a^.

	SE-50bp, 50X^b^			SE-150bp, 20X			PE-50bp, 20X		
Aligner	Unc^c^	CDS	genes	Unc	CDS	genes	Unc	CDS	genes
BBMap	4.94%	2.87%	1,982	2.22%	1.85%	897	2.55%	1.73%	855
BFAST	5.10%	2.91%	2,016	3.05%	2.01%	989	5.40%	3.07%	2,124
Bowtie	4.56%	2.84%	1,960	2.22%	1.85%	897	2.35%	1.61%	781
BWA	4.57%	2.84%	1,960	2.22%	1.85%	897	2.17%	1.61%	785
Novoalign	4.56%	2.84%	1,960	2.22%	1.85%	897	2.41%	1.65%	863

^a^The percentage of nucleotides with read depth coverage < 3.

^b^Types of sequence data (SE, single-end; PE, paired-end), read length, and fold genome coverage.

^c^Values for the percentage of the uncovered genome (Unc), percentage of uncovered coding sequences (CDS), and the number of genes that contain uncovered coding sequence.

Although extra data does not significantly improve genome coverage, we determined that the type of sequence data does have an impact. We generated two additional data sets for comparison: single-end 150-bp reads (the current maximum read length on Illumina’s production-scale sequencers) and paired-end 50-bp reads, each at 20-fold coverage. With the exception of BFAST, which provided the least coverage across all types of data tested, the fraction of uncovered genome was reduced to 2.22% when using single-end 150-bp data, with corresponding reductions in the amounts of uncovered coding sequence (1.85%) and numbers of genes affected (897) (Table 2). Paired-end 50-bp data provided comparable improvement in genome coverage (2.17–2.55% uncovered) and slightly better gene coverage (1.61–1.73% uncovered coding sequence, affecting 781–863 genes) (Table 2). However, the cost for those types of data is significantly higher (roughly twice that of single-end 50-bp sequencing), so the user must balance the superior coverage to be gained against the additional expense.

### EMS-type data

The evaluation of accuracy requires sequence data for which the entire constellation of variants is known, to discriminate true-positive, false-positive, and false-negative variant calls. Therefore, we produced simulated reads from a modified reference genome that contained defined variants. At the same time, we sought to replicate as faithfully as possible the properties of actual data derived from *C*. *elegans*. Ethyl methanesulfonate (EMS) treatment is the most common method of mutagenesis in this species. The spectrum of EMS-induced alleles has been characterized in *C*. *elegans* [32], so we incorporated the same types and frequencies of those variants (predominantly SNPs; see Materials and Methods for details) in our test data. The simulated reads also contained an error distribution typical of Illumina sequencers (the most widely used sequencing platform). The majority of the published data for mutation identification in *C*. *elegans* are single-end 50-bp reads, so we generated data of that type at 20-fold coverage. We also included an additional 5% of *E*. *coli* reads to mimic the bacterial food source that is a common contaminant.

We performed alignment and variant calling in all pair-wise combinations, with default parameters for all software, generating a total of 20 data sets. Three pairs of software yielded unexpected results that suggested idiosyncratic interactions between the components. The BBMap+FreeBayes combination produced only SNP calls but no insertions or deletions. That result was a consequence of how sequence mismatches were represented in the alignment file (specifically, the CIGAR string of the SAM file; see [33] for format details). The behavior was corrected by addition of an optional format flag to the default command (see S2 File for all command-line parameters). BFAST+FreeBayes yielded an excessive number of false-positive variant calls (2940 of 5083 total variants, equaling 58%). Filtering the output by a minimum quality score, as recommended by best-practice guidelines (see below), reduced the number of false-positive variants by >98% while retaining >98% of true-positive calls. BFAST+VarScan2 also produced a large number of false-positive variant calls. VarScan2 represents allele quality by p-value, whereas the other variant callers report PHRED-scaled quality scores as specified by the VCF standards [34] However, we were unable to define a clear-cut threshold for BFAST+VarScan2 to discriminate true-positive from false-positive calls, even after converting quality scores to the specified format. Therefore, we excluded that combination from further analysis, leaving nineteen minimal variant-calling pipelines for evaluation.

**Fig 1:**
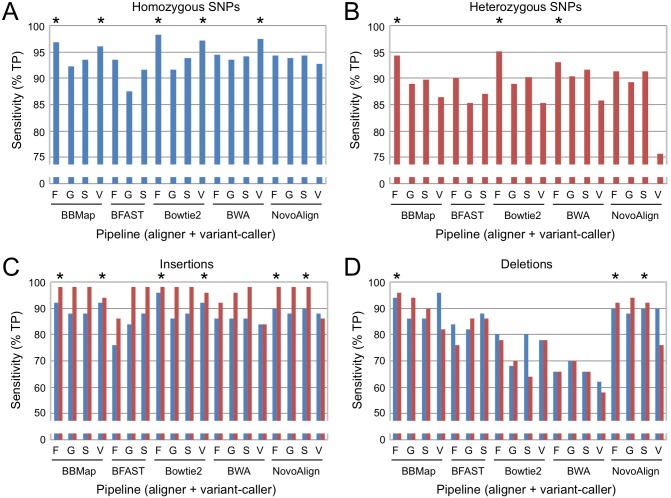
Sensitivity of variant-calling pipelines for EMS-type mutations. The percentage of true-positive (TP) mutation calls is indicated for each combination of aligner plus variant caller (F, FreeBayes; G, GATK HaplotypeCaller; S, SAMtools/BCFtools; V, VarScan2) and plotted separately for different categories of variants. Homozyous (blue) and heterozygous (red) mutation calls are indicated by color. Asterisks (*) indicate the best-performing pipelines in each category. (**A**) Homozygous SNPs. (**B**) Heterozygous SNPs. (**C**) Insertions. (**D**) Deletions.

We compared the output from each pair of software components to the list of known variants and tallied the number that were true-positive, false-positive, or mismatch calls (an incorrect variant call within 20 nucleotides of a *bona fide* variant). We categorized the results by allele type and zygosity, and identified the pipelines in each class with the most sensitivity (highest number of true-positive calls). For homozygous SNPs, sensitivity ranged from 87% to 98%, and five pipelines correctly identified greater than 95% of variants (Fig 1A, indicated by asterisks). All pipelines were less sensitive to heterozygous SNPs, in some cases significantly so (range 76–95%), but three identified at least 93% of variants (Fig 1B). SNP identification was influenced more by the variant caller than aligner; all of the top pipelines employed either VarScan2 (for homozygous SNPs) or FreeBayes (both homozygous and heterozygous SNPs).

The pipelines exhibited different characteristics for insertions and deletions compared to SNPs. First, sensitivity was not consistently affected by zygosity. Six pipelines produced true-positive rates ≥90% for both homozygous and heterozygous insertions, and three of those also identified ≥90% of homozygous and heterozygous deletions (Fig 1C and 1D, respectively). Second, deletion calls (but not insertions) were more dependent upon the aligner than the variant caller, with relative sensitivities of BBMap ≈ Novoalign > BFAST > Bowtie > BWA. Note that this pattern differs from the relative fraction of unaligned reads (Table 1; Bowtie ≈ BWA ≈ Novoalign < BBMap < BFAST), so sensitivity for detection of deletions does not correlate with overall alignment rates.

We also included in our data a small number (six each, one per chromosome) of homozygous structural variants that are not typical of EMS mutagenesis: complex insertion/deletions (indels), large deletions, and transposon insertions. The pipelines that we tested were not designed for such variants, and that limitation was largely borne out by the results; 13 failed to identify even a single structural variant correctly (S1 Fig**)**. However, one combination performed significantly better than anticipated. BBMap+FreeBayes correctly identified four (67%) of the complex indels, and all six (100%) of the large deletions. While failing to identify any of the transposon insertions accurately, BBMap+FreeBayes also reported one or more mismatch calls at each of the transposon loci. Although beyond the scope of the current study, we note that superior detection of indels and structural variants can be obtained using paired-end data and specialized software for that application.

**Fig 2:**
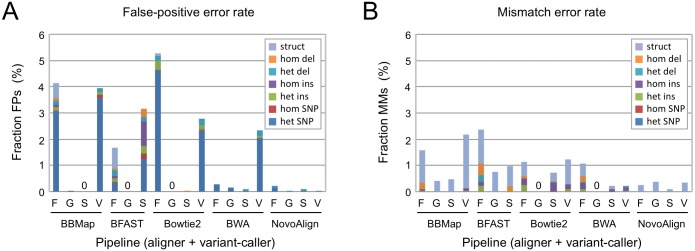
Error rates of variant-calling pipelines. The fraction of (**A**) false-positive (FP) and (**B**) mismatch (MM) mutation calls as a percentage of the total number of variants called by each pipeline. Variant callers are indicated as in Fig 1. Color codes for different categories of variants are indicated by the key (inset). Abbreviations: struct, structural variant; hom, homozygous; het, heterozygous; del, deletion; ins, insertion; SNP, single-nucleotide polymorphism.

We evaluated the error rates for each pair of software, and found them to be low. For false-positive calls, the proportion ranged from 0–5.2% of the total number of variant calls (Fig 2A). We observed that the most sensitive pipelines exhibited the highest error rates (compare with Fig 1A), and that heterozygous SNPs were the most common class of false-positive variants. Similarly, mismatch calls were a small fraction of the total (range 0–2.4%), and predominantly associated with the structural variants (Fig 2B). Those observed error rates would have minimal impact on mutation identification. The number of candidate alleles in a typical mapping interval is low (<10), and candidates can be validated by Sanger sequencing and/or functional criteria such as RNAi, transgene rescue, or CRISPR/Cas9-mediated genome engineering (see [35] for examples). Consequently, false-positive calls are readily eliminated and mismatch calls might even prove useful for identifying the causative locus, even if the molecular description of that variant is incorrect. Therefore, we conclude that errors are not a significant challenge to mutation identification in *C*. *elegans* for data derived from EMS-mutagenized strains.

Although we used default software parameters (with the exceptions noted for idiosyncratic interactions), all of the variant callers offer best-practice guidelines for improved performance. We considered the utility of implementing those guidelines for our analysis, but anticipated minimal changes in our results. Most of those additional steps are designed to reduce false-positive calls, which (with the exception of BFAST+FreeBayes, addressed above) are already a small fraction of the total. Two variant callers (GATK and SAMtools) recommend the same two best-practice additions to the workflow prior to variant calling: 1) the marking or removal of duplicate reads to eliminate error-containing PCR duplicates that could give rise to false-positive calls, and 2) base quality recalibration to discriminate true variants from sequencing errors that might generate false-positive calls. SAMtools further recommends local realignment around indels to resolve alignment ambiguities likely to produce false-negative or mismatch calls. GATK includes variant quality recalibration after variant calling to improve specificity. Finally, all of the variant callers endorse filtering (by read depth, strand bias, mapping quality, and/or other criteria) to remove probable false-positive calls.

While best-practice guidelines are generally applicable to variant calling, many of those steps are poorly suited to our specific example of mutation identification in *C*. *elegans*. Recalibration of both base quality and variant quality requires a list of known variants to establish metrics for true-positive calls. Although that information is available for our simulated reads, it is generally unavailable for actual data obtained from mutant strains. Many of the parameters for filtering false-positive calls are subjective and require optimization for each workflow. Filtering also reduces the sensitivity to true-positive calls and runs the risk of excluding *bona fide* variants. Therefore, we elected to forego many of the complexities of best-practice guidelines. We incorporated duplicate removal and a minimum read depth of three into all of our pipelines (in addition to the pipeline-specific modifications mentioned previously), but otherwise used default parameters for subsequent analyses.

### Hawaiian SNP mapping data

EMS mutagenesis in *C*. *elegans* generates thousands of variants, of which only one is responsible for the phenotype of interest. By using the appropriate strain for crossing, both map position and candidate alleles can be ascertained from the sequence data. One popular method utilizes the highly polymorphic Hawaiian strain isolate [27] for simultaneous mapping and mutation identification [36]. The strain contains >370,000 SNPs [37], of which ~30% have been annotated in the wild-type reference genome ([38–39]; D. Spencer and R. H. Waterston, unpublished data). After crossing and pooling of homozygous mutant F2 animals for sequencing, the Hawaiian SNPs exhibit anti-linkage to the causative mutation: the SNPs are heterozygous at unlinked loci, but the frequency drops to zero near the mutation locus. Hawaiian SNP frequencies are determined by variant calling, and then plotted on the physical map to identify the gap that defines the mutation interval. The density of annotated Hawaiian SNPs (roughly one per kilobase) allows mapping at high resolution, and the method has been widely adopted by the community (e.g., [2, 35–36, 40– 43]).

**Fig 3:**
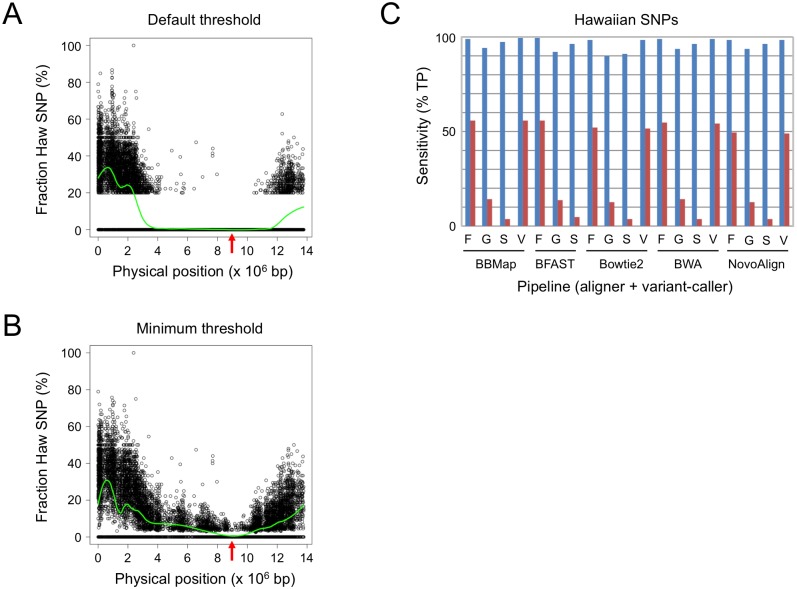
Sensitivity of variant-calling pipelines for Hawaiian SNPs. (**A**) Plot of Hawaiian (Haw) SNP fraction vs. physical map position using the default threshold for variant calling. Shown is a representative example used to map *lin-9(n112)*, located on chromosome III at position 8.9 Mb (red arrow), with BBMap+FreeBayes for variant calling. Green line, LOESS regression of the SNP fraction. (**B**) The same data as A with a minimum threshold of 1% variant call and one supporting read for FreeBayes. Mapping data from [26]. (C) Sensitivity for 50% (blue) and 5% (red) Hawaiian SNPs. The percentage of true-positive (TP) Hawaiian SNP calls are indicated for each pipeline. Variant callers are indicated as in Fig 1.

The popularity of Hawaiian polymorphism mapping led us to evaluate our variant-calling pipelines for that application. The most important feature for accurate mapping is the ability to detect low-frequency SNPs from the pooled sample to delimit the mapping interval. Default thresholds for variant-calling software adversely affect low-frequency SNP detection. The prior expectation is that variants are either homozygous or heterozygous, and zygosity is determined by the fraction of reads that contains the variant call. Those that fail to meet a minimum threshold are filtered by default to remove false-positives arising from sequencing errors. However, the fractional representation of Hawaiian SNPs approaches zero near the mutation locus, so default thresholds remove *bona fide* low-frequency SNPs and reduce mapping resolution. Data from a representative mapping cross illustrate the impact of thresholds on the mapping interval (Fig 3, compare A and B). Therefore, it is necessary to specify a lower threshold during variant calling to capture the low-abundance SNPs for maximum mapping resolution.

We assessed the SNP detection limits of our pipelines from actual (not simulated) sequence data by combining Hawaiian and N2 wild-type samples (single-end, 50-bp, 20-fold coverage) in two different ratios: 50% Hawaiian data, representing unlinked heterozygous SNPs, and 5% Hawaiian data, equal to one SNP-containing read per 20-fold coverage. We performed variant calling with all 19 pipelines, with parameters adjusted to obtain maximum sensitivity (see S3 File), and used 103,326 high-confidence Hawaiian SNPs as our true-positive variants for validation. Sensitivity was high (>90%) for all of the pipelines for the 50% Hawaiian sample, but varied significantly for the 5% Hawaiian sample (Fig 3C). Sensitivity for the latter sample was largely unaffected by the aligner but highly dependent upon the variant-calling software. FreeBayes and VarScan2 were the most sensitive, identifying 49–56% Hawaiian SNPs. GATK was considerably less sensitive, with 12–14% Hawaiian SNPs being identified. SAMtools is not designed to detect low-frequency variants from pooled samples and, as a result, offered the worst performance (only 3–4% Hawaiian SNPs detected). Note that the parameters required for maximum sensitivity cannot discriminate low-frequency SNPs from sequencing errors and are not suitable for identifying candidate mutations; those should be called independently using default parameters as described previously.

### Recommendations

Our objective was to identify an optimized workflow for mutant allele detection in *C*. *elegans* from whole-genome sequence data. For sequencing, the most complete coverage of the genome was obtained using paired-end sequencing with 50-bp (or longer) reads at 20-fold depth of coverage. Absent other considerations, that would be the preferred choice; however, that option may not be particularly cost-effective. We find that single-end 50-bp sequencing at 20-fold depth of coverage is sufficient to detect mutations in ~97% of protein-coding sequences, providing a reasonable balance between coverage and cost.

For data analysis, we recommend the combination of BBMap for alignment and FreeBayes for variant calling (a detailed, step-by-step workflow is diagrammed as S2 Fig with command-line instructions as S5 File). The most important criterion for mutation identification in *C*. *elegans* is sensitivity to true-positive variant calls: candidate alleles can be validated by independent methods, but only if detected by the pipeline. In contrast, the error rate is not a significant concern with the recommended pipeline, because false-positive and mismatch calls represent only a small fraction of the total and can be readily eliminated during candidate allele validation. Although no single pipeline was the most sensitive for every class of variant, the combination of BBMap for alignment and FreeBayes for variant calling was consistently among the top performers across all categories of EMS-generated mutations. In addition, BBMap+FreeBayes was able to detect a significant number of the structural variants that were largely missed by all of the other pipelines. This combination also offered robust performance for low-frequency Hawaiian SNP detection and interval mapping using appropriate parameters. While continual improvements in sequencing platforms and alignment and variant-calling software will require ongoing evaluation, our current recommendation is BBMap plus FreeBayes as the preferred tools for mutation identification in *C*. *elegans*.

## Supporting information

S1 Fig Detection of structural variants. The number of structural variants (SVs) in each category that were identified by true-positive (top) or mismatch (bottom) calls for each pipeline. Color codes for different categories of structural variants are indicated by the key.
(TIFF)

S2 Fig Schematic diagram of recommended workflow for variant calling. (TIFF)

S1 File List of software tools and versions used. (DOCX)

S2 File Software commands for default variant-calling pipelines. (DOCX)

S3 File Software commands for low-sensitivity Hawaiian SNP calling. (DOCX)

S4 File BED file of uncovered regions shared by SE-50bp data at 20-fold and 50-fold genome coverage. (DOCX)

S5 File Command-line instructions for recommended BBMap + FreeBayes pipeline. (DOCX)

S1 Table List of EMS-type variants. (XLSX)

S2 Table List of 103,326 Hawaiian SNPs from reference genome version WS250 in VCF file format. (TXT)
