# Supplementary figures and images for: Evaluating alignment and variant-calling software for mutation identification in C. elegans by whole-genome sequencing
Source: PLoS One. 2017 Mar 23;12(3):e0174446. doi: 10.1371/journal.pone.0174446 (PMC5363872; doi:10.1371/journal.pone.0174446)

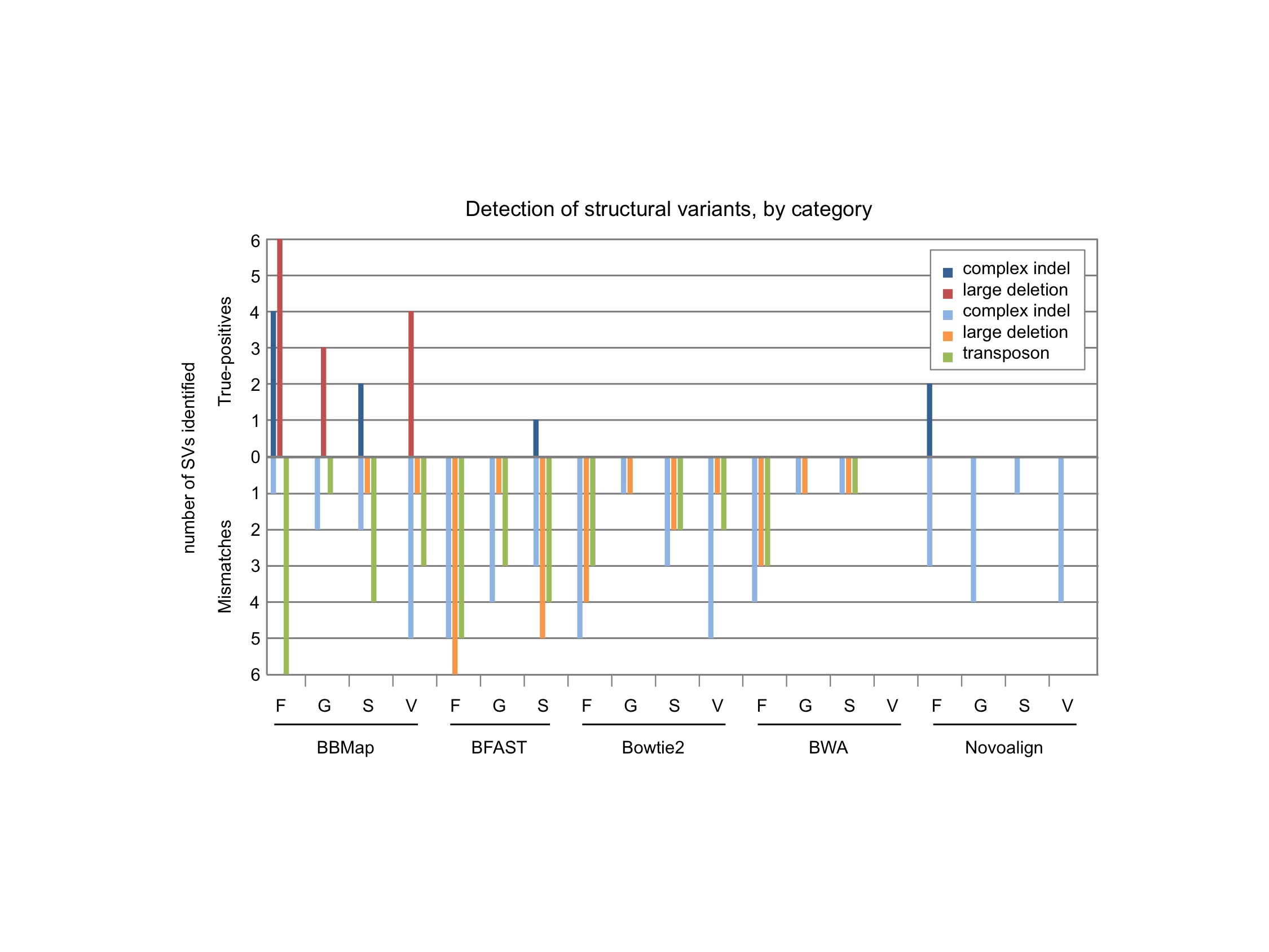

Supplement: S1 Fig — The number of structural variants (SVs) in each category that were identified by true-positive (top) or mismatch (bottom) calls for each pipeline. Color codes for different categories of structural variants are indicated by the key. (TIFF) [file pone.0174446.s001.tiff]

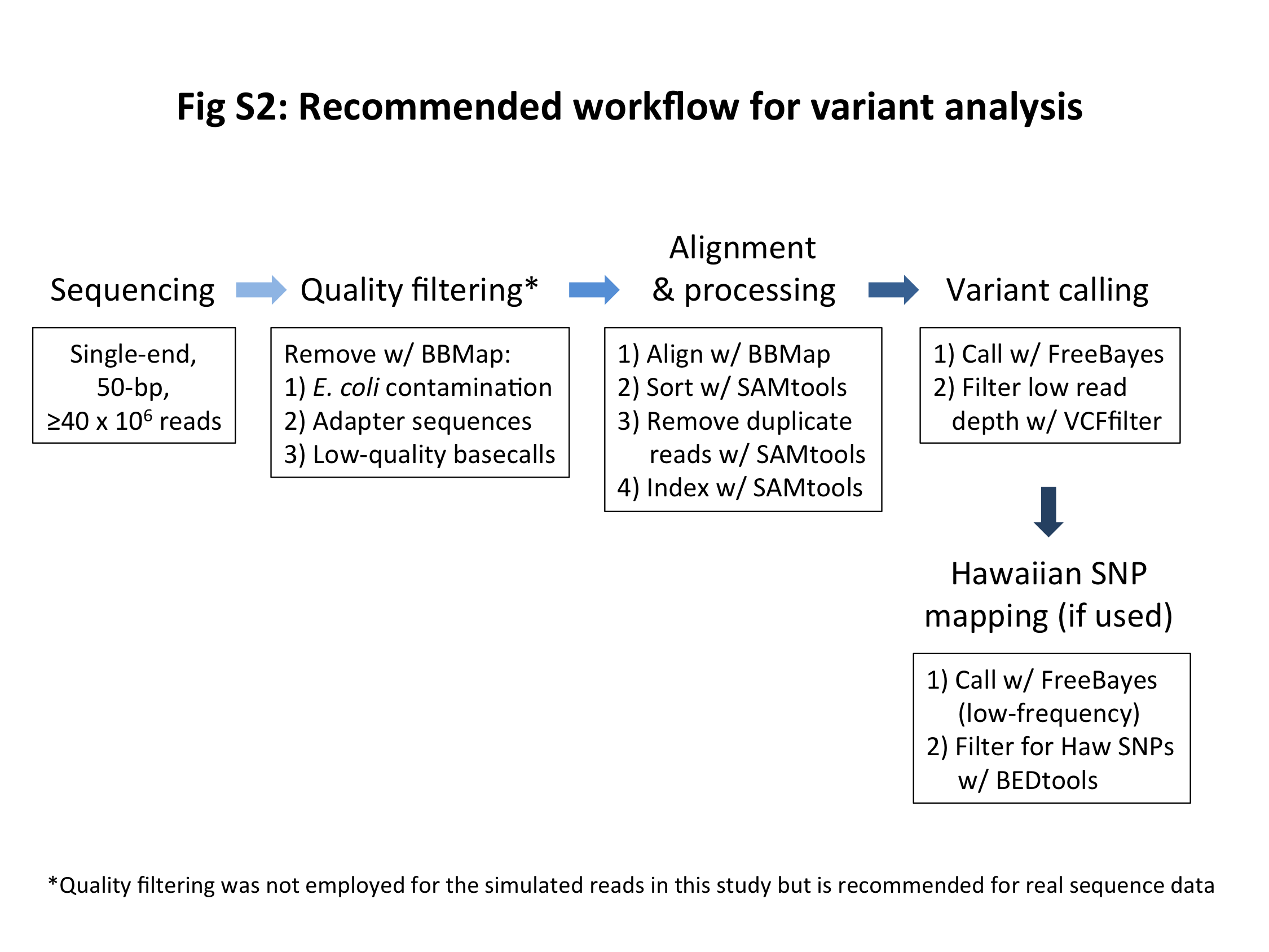

Supplement: S2 Fig — (TIFF) [file pone.0174446.s002.tiff]
