# Supplementary material for: Evaluating alignment and variant-calling software for mutation identification in C. elegans by whole-genome sequencing
Source: PLoS One. 2017 Mar 23;12(3):e0174446. doi: 10.1371/journal.pone.0174446 (PMC5363872; doi:10.1371/journal.pone.0174446)
Supplement: S1 File — (DOCX) [file pone.0174446.s003.docx]

**SUPPLEMENT FILE S1: SOFTWARE TOOLS**

BBMap, version 34.08, https://sourceforge.net/projects/bbmap/

BFAST, version 0.7.0a, https://sourceforge.net/projects/bfast/

BWA, version 0.7.12-r1039, https://github.com/lh3/bwa

Bowtie 2, version 2.2.5, http://bowtie-bio.sourceforge.net/bowtie2/index.shtml

NovoAlign, version V3.02.10, http://www.novocraft.com/products/novoalign/

FreeBayes, version v1.0.2-16-gd466dde, https://github.com/ekg/freebayes

GATK, version 3.4-0-g7e26428, https://software.broadinstitute.org/gatk/download/

SAMtools/BCFtools, version 1.3.1, https://sourceforge.net/projects/samtools/files/samtools/

VarScan2, version v2.3.9, https://sourceforge.net/projects/varscan/files/

Picard, version 1.134, https://broadinstitute.github.io/picard/

VCFtools, version 0.1.15, https://vcftools.github.io/index.html

BEDtools, version 2.15.0, https://github.com/arq5x/bedtools2

VCFLIB, version v1.0.0, https://github.com/vcflib/vcflib
