# Supplementary material for: Evaluating alignment and variant-calling software for mutation identification in C. elegans by whole-genome sequencing
Source: PLoS One. 2017 Mar 23;12(3):e0174446. doi: 10.1371/journal.pone.0174446 (PMC5363872; doi:10.1371/journal.pone.0174446)
Supplement: S2 File — (DOCX) [file pone.0174446.s004.docx]

**SUPPLEMENT FILE S2: SOFTWARE COMMANDS**

# CONVENTIONS FOR COMMAND-LINE INSTRUCTIONS:

# Comments are preceded by hash (#)

# Commands are preceded by greater-than sign (>)

# User-specific variables are shown in *UPPER_CASE_ITALICS*

# Single-line commands that are too long are split by backslash (\)

# (note: do not include backslash in command)

# Download a copy of the reference genome from WormBase

# (available via FTP at ftp.wormbase.org/pub/wormbase/species/c_elegans/\

# sequence/genomic/c_elegans.PRJNA13758.*WS_VERSION*.genomic.fa.gz)

# Extract and rename the reference genome

> gunzip c_elegans.PRJNA13758.*WS_VERSION*.genomic.fa.gz

> mv c_elegans.PRJNA13758.*WS_VERSION*.genomic.fa *WS_VERSION.FA*

# I. INDEX THE REFERENCE GENOME FOR VARIOUS SOFTWARE TOOLS

# (note: indexing is performed only once)

# (note: BBMap indexing of reference genome is created during alignment)

# Commands for BFAST:

> bfast fasta2brg -f *WS_VERSION.FA*

> bfast index -f *WS_VERSION.FA* -m 1111111111111111111111 -w 14 -i 1

> bfast index -f *WS_VERSION.FA* -m 1111101110111010100101011011111 -w 14 -i 2

> bfast index -f *WS_VERSION.FA* -m 1011110101101001011000011010001111111 -w 14 -i 3

> bfast index -f *WS_VERSION.FA* -m 10111001101001100100111101010001011111 -w 14 -i 4

> bfast index -f *WS_VERSION.FA* -m 11111011011101111011111111 -w 14 -i 5

> bfast index -f *WS_VERSION.FA* -m 111111100101001000101111101110111 -w 14 -i 6

> bfast index -f *WS_VERSION.FA* -m 11110101110010100010101101010111111 -w 14 -i 7

> bfast index -f *WS_VERSION.FA* -m 111101101011011001100000101101001011101 -w 14 -i 8

> bfast index -f *WS_VERSION.FA* -m 1111011010001000110101100101100110100111 -w 14 -i 9

> bfast index -f *WS_VERSION.FA* -m 1111010010110110101110010110111011 -w 14 -i 10

# Command for Bowtie2

> bowtie2-build -f *WS_VERSION.FA*

# Command for BWA

> bwa index *WS_VERSION.FA*

# Command for Novoalign

> novoindex *WS_VERSION* *WS_VERSION.FA*

# Command for GATK

> java -jar picard.jar CreateSequenceDictionary REFERENCE=*WS_VERSION.FA* \

OUTPUT=*WS_VERSION*.*DICT*

# Command for SAMtools (note: required for GATK and VarScan2)

> samtools faidx *WS_VERSION.FA*

# II. ALIGN SEQUENCE DATA TO REFERENCE GENOME

# Command for BBMap

# (note: optional flag 'sam=1.3' is used for compatibility with variant caller)

> bbmap.sh ref=*WS_VERSION.FA* sam=1.3 in=*SEQUENCE_DATA.FASTQ* out=*SEQUENCE_DATA.SAM*

# Command for BFAST

# (note: optional flag '-n 4' specifies the number of threads to use)

> bfast easyalign -f *WS_VERSION.FA* -r *SEQUENCE_DATA.FASTQ* -n 4 > *SEQUENCE_DATA.SAM*

# Command for Bowtie2

> bowtie2 -x *WS_VERSION* -U *SEQUENCE_DATA.FASTQ* -S *SEQUENCE_DATA.SAM*

# Commands for BWA

> bwa aln *WS_VERSION.FA* *SEQUENCE_DATA.FASTQ* > *SEQUENCE_DATA.SAI*

> bwa samse *WS_VERSION.FA* *SEQUENCE_DATA.SAI* *SEQUENCE_DATA.FASTQ* > *SEQUENCE_DATA.SAM*

# III. PREPARE EACH ALIGNED DATA SET FOR VARIANT CALLING

# Convert data from SAM to BAM format

# (note: optional flag ‘-q 4’ removes reads with mapping quality < 4)

> samtools view –b –q 4 -o *SEQUENCE_DATA.BAM* *SEQUENCE_DATA.SAM*

# Sort data by chromosome/position

# (note: optional flag '-@ 4' specifies the number of threads)

> samtools sort -O bam -o *SEQUENCE_DATA_SORTED.BAM* -T *TEMP* -@ 4 *SEQUENCE_DATA.BAM*

# Remove duplicate reads from data

> samtools rmdup -s *SEQUENCE_DATA_SORTED.BAM SEQUENCE_DATA_DEDUP.BAM*

# Index data

> samtools index *SEQUENCE_DATA_DEDUP.BAM*

# IV. PERFORM VARIANT CALLING FOR EACH DATA SET

# Command for FreeBayes

> freebayes -f *WS_VERSION.FA* *SEQUENCE_DATA_DEDUP.BAM* > *SEQUENCE_DATA.VCF*

# (note: for BFAST data, filter using VCFLIB 'vcffilter' command)

> vcffilter -f "QUAL > 1" *SEQUENCE_DATA.VCF* > *SEQUENCE_DATA_Q1.VCF*

# Commands for GATK

> java -jar picard.jar AddOrReplaceReadGroups I=*SEQUENCE_DATA_DEDUP.BAM* \

O=*SEQUENCE_DATA_RG.BAM* RGLB=*WS_VERSION* RGPL=*SEQUENCING_PLATFORM* \

RGPU=*INDEX_SEQUENCE* RGSM=*SAMPLE_NAME*

> samtools index *SEQUENCE_DATA_RG.BAM*

> java -jar GenomeAnalysisTK.jar -T HaplotypeCaller -R *WS_VERSION.FA* \

-I *SEQUENCE_DATA_RG.BAM* -o *SEQUENCE_DATA.VCF*

# Command for SAMTools/BCFTools

> samtools mpileup -ugf *WS_VERSION.FA* *SEQUENCE_DATA_DEDUP.BAM* | \

bcftools call -vmO v -o *SEQUENCE_DATA.VCF*

# Commands for VarScan2

> samtools mpileup -Bf *WS_VERSION.FA* *SEQUENCE_DATA_DEDUP.BAM* > *SEQUENCE_DATA.MPILEUP*

> java -jar *VARSCAN.VERSION.JAR* mpileup2cns *SEQUENCE_DATA.MPILEUP* \

--output-vcf 1 --variants > *SEQUENCE_DATA.VCF*

# Filter for a minimum read depth of three

> vcffilter -f "DP > 2" *SEQUENCE_DATA.VCF* > *FILTERED_DATA.VCF*

# V. VARIANT-CALLING THRESHOLD FOR HAWAIIAN SNP MAPPING PLOTS

# (note: use same workflow as above through step III)

# (note: optional flags '-F 0.01' for 1% variant calls

# and '-C 1' supported by at least one read)

> freebayes -f *WS_VERSION.FA* -F 0.01 -C 1 *SEQUENCE_DATA_DEDUP.BAM* > *SEQUENCE_DATA.VCF*
