# Supplementary material for: Evaluating alignment and variant-calling software for mutation identification in C. elegans by whole-genome sequencing
Source: PLoS One. 2017 Mar 23;12(3):e0174446. doi: 10.1371/journal.pone.0174446 (PMC5363872; doi:10.1371/journal.pone.0174446)
Supplement: S3 File — (DOCX) [file pone.0174446.s005.docx]

**SUPPLEMENT FILE S3: COMMANDS FOR LOW-SENSITIVITY HAW SNP CALLING**

**CONVENTIONS FOR COMMAND-LINE INSTRUCTIONS**

A. Commands are preceded by greater-than sign (>)

B. User-specific variables are shown in *UPPER_CASE_ITALICS*

C. Single-line commands that are too long are split by backslash (\)

(**note**: do not include backslash in command)

**1. Use same default alignment parameters as Supplement File S2**

**2. Convert SAM to BAM with SAMtools**

(**note:** filter reads with low mapping quality with ‘-q 4’ flag)

> samtools view -b -q 4 -o *DATA.BAM* > *DATA.SAM*

**3. Sort data by chromosome/position with SAMtools**

(**note**: optional flag '-@ 4' specifies the number of threads)

> samtools sort -O bam -o *DATA_SORTED.BAM* -T temp -@ 4 *DATA.BAM*

**4. Index sorted data using SAMtools**

> samtools index *DATA_SORTED.BAM*

**5. Perform variant calling with FreeBayes**

> freebayes -f *WS_VERSION.FA* -F 0.01 -C 1 --pooled-continuous *DATA_SORTED.BAM* > \

*DATA_FB.VCF*

**6. Perform variant calling with GATK**

> java -jar picard.jar AddOrReplaceReadGroups I=*DATA_SORTED.BAM* O=*DATA_RG.BAM* \

VALIDATION_STRINGENCY=LENIENT RGLB=*WS_VERSION* RGPL=*PLATFORM* \

RGPU=*INDEX_SEQUENCE* RGSM=*SAMPLE_NAME*

> samtools index *DATA_RG.BAM*

> java -jar GenomeAnalysisTK.jar -T HaplotypeCaller -R *WS_VERSION.FA* \

-I *DATA_RG.BAM* -ploidy 40 -o *DATA_GATK.VCF*

**7. Perform variant calling with SAMtools/BCFtools**

> samtools mpileup -Bugf *WS_VERSION.FA* *DATA_SORTED.BAM* | bcftools call \

-vmO v -o *DATA_SAM.VCF*

**8. Perform variant calling with VarScan2**

> samtools mpileup -Bf *WS_VERSION.FA*  *DATA_SORTED.BAM* > \

*DATA_SORTED.MPILEUP*

> java -jar VarScan.jar mpileup2snp *DATA_SORTED.MPILEUP* --min-reads2 1 \

--min-coverage 5 --min-var-freq 0.01 --p-value 1.0 --output-vcf 1 > *DATA_VAR.VCF*
