# Supplementary material for: Evaluating alignment and variant-calling software for mutation identification in C. elegans by whole-genome sequencing
Source: PLoS One. 2017 Mar 23;12(3):e0174446. doi: 10.1371/journal.pone.0174446 (PMC5363872; doi:10.1371/journal.pone.0174446)
Supplement: S4 File — (DOCX) [file pone.0174446.s006.docx]

I 0 386

I 477 535

I 635 681

I 757 800

I 2094 2164

I 2271 2312

I 2372 2421

I 2509 2581

I 2796 2995

I 3094 3313

I 3484 3485

I 3486 3494

I 11924 12305

I 13654 13670

I 15606 15711

I 22372 22408

I 22506 22522

I 22872 22893

I 23020 23066

I 23699 23711

I 23990 23994

I 24422 24434

I 31277 31291

I 31390 31400

I 31622 31634

I 38458 38477

I 45035 45141

I 45322 45325

I 45412 45445

I 45613 45649

I 46608 46660

I 51477 51528

I 51671 51890

I 52010 52101

I 57750 57807

I 58313 58353

I 58475 58664

I 58756 58819

I 60859 60919

I 61013 61203

I 61325 61360

I 65343 65364

I 67163 67175

I 67358 67473

I 67600 67695

I 67807 67818

I 95717 95722

I 114846 114874

I 115350 115356

I 136926 136946

I 138224 138248

I 138434 138447

I 138722 138747

I 139102 139239

I 139468 139504

I 140781 140848

I 140973 140996

I 143255 143273

I 147463 147483

I 148413 148573

I 150607 150637

I 150756 150821

I 152233 152268

I 152498 152635

I 152994 153014

I 153293 153300

I 153498 153521

I 185207 185944

I 186031 186889

I 187071 188539

I 196960 197120

I 198330 198403

I 198784 198814

I 205848 205860

I 206137 206314

I 208744 208764

I 210730 210820

I 211171 211181

I 211265 211333

I 211382 211521

I 211701 211734

I 211989 212003

I 212093 212163

I 212261 212462

I 212589 212652

I 212746 212769

I 212827 212860

I 213179 213205

I 213668 213818

I 213919 214059

I 214166 214230

I 214323 214333

I 214449 214473

I 214655 214661

I 214756 214952

I 215058 215107

I 215254 215305

I 215400 215422

I 215544 215561

I 216086 216143

I 216239 216425

I 216585 216695

I 216787 216842

I 216969 216990

I 217097 217163

I 217494 217502

I 217595 217635

I 217728 217739

I 217828 217861

I 218786 218794

I 218889 218899

I 219146 219223

I 220027 220227

I 221085 221109

I 221199 221262

I 223663 225034

I 225130 225354

I 225512 226058

I 226221 226329

I 226547 226621

I 226795 226838

I 226976 227111

I 227192 227436

I 232796 232839

I 233080 233091

I 233183 233232

I 233453 233455

I 233549 233582

I 233934 233992

I 234063 234492

I 234808 235087

I 235241 235295

I 235364 235468

I 235554 235726

I 235836 235954

I 236085 236122

I 236288 236449

I 236542 236808

I 236990 237046

I 237195 237233

I 237331 237350

I 237624 237732

I 238565 238653

I 238744 238880

I 238966 239072

I 239161 239205

I 239298 239310

I 239394 239493

I 239587 239640

I 242295 242350

I 242444 242540

I 242627 242638

I 242807 242884

I 243100 243208

I 243372 243919

I 244077 244303

I 244399 245772

I 252162 252283

I 252376 252395

I 252481 252676

I 252861 252985

I 253157 253198

I 253360 253469

I 253744 253790

I 253975 253996

I 254296 254393

I 254490 254540

I 254631 254644

I 254744 254775

I 255036 255065

I 255156 255200

I 255252 255285

I 255375 255395

I 256792 256811

I 256994 257084

I 263851 263972

I 264061 264105

I 280565 280695

I 280788 281467

I 281582 281596

I 281710 282066

I 282162 282373

I 282458 282582

I 305602 305657

I 315776 315924

I 316288 316521

I 316613 316651

I 316826 316888

I 317100 317124

I 317216 317286

I 317375 317393

I 317499 317621

I 317992 318157

I 326368 326370

I 328512 328551

I 328627 328800

I 329017 329058

I 329162 329203

I 329296 329359

I 329451 329545

I 329685 329730

I 334723 334745

I 334961 334984

I 335046 335048

I 335333 335393

I 335485 335556

I 336512 336546

I 338609 338646

I 338753 338837

I 338926 340217

I 340353 340441

I 340515 340963

I 341026 341113

I 354898 355046

I 355146 355208

I 359198 359206

I 362462 362500

I 366844 366857

I 375379 375556

I 375718 375770

I 376735 376912

I 377082 377138

I 401141 401606

I 401699 401726

I 401821 401869

I 403951 403958

I 404015 404019

I 405862 405863

I 406790 406811

I 410151 410200

I 413351 413376

I 414211 414416

I 414551 414655

I 414828 414915

I 415000 415266

I 415341 415407

I 415498 415711

I 425655 425690

I 425872 425880

I 431641 431685

I 431784 431876

I 432041 432118

I 432250 432267

I 432357 432376

I 475405 475436

I 487136 487148

I 492660 492669

I 494837 494874

I 494937 494943

I 495387 495396

I 495747 495748

I 500508 500539

I 500699 500779

I 504669 504707

I 505067 505106

I 505195 505196

I 510341 510365

I 512195 512211

I 514427 514432

I 514634 514636

I 533380 533454

I 533647 533656

I 543115 543145

I 543718 543728

I 557772 557843

I 558090 558174

I 561060 561072

I 571352 571405

I 580854 580859

I 580951 580952

I 594137 594227

I 594348 594444

I 594652 594653

I 596980 596986

I 598790 599080

I 599170 599189

I 599281 601072

I 605899 605913

I 606003 606233

I 609905 609985

I 610043 610260

I 610348 610427

I 610512 610927

I 611129 611192

I 616765 616870

I 617070 617226

I 617442 617577

I 617817 617827

I 618455 618515

I 618608 618741

I 618873 618964

I 619261 619805

I 619941 620179

I 620272 620588

I 620683 621316

I 621907 621979

I 622245 622275

I 622632 622734

I 622950 623022

I 623790 623852

I 623923 623987

I 629711 629739

I 629809 629815

I 630239 630257

I 633129 633235

I 633587 633661

I 633756 633807

I 646153 646164

I 646291 646318

I 650075 650784

I 650859 651561

I 655433 655435

I 669722 669823

I 669915 669918

I 670036 670184

I 670534 670763

I 671030 671173

I 671338 671529

I 673838 673854

I 676225 676567

I 676648 676664

I 676828 677396

I 677727 677863

I 678023 678084

I 678168 678279

I 678371 678848

I 678961 679393

I 695590 695734

I 696269 696313

I 698669 698838

I 699023 699348

I 699403 699729

I 701068 701087

I 701183 701187

I 705913 705932

I 706019 706044

I 706277 706327

I 710762 710771

I 713972 713981

I 714120 714139

I 718483 718596

I 722562 722590

I 722715 722725

I 722822 722916

I 722977 723143

I 723271 723429

I 727365 727757

I 727864 727867

I 727917 728010

I 728102 728133

I 728260 728297

I 728412 728557

I 728642 728680

I 728771 728922

I 729014 729075

I 729259 729261

I 729350 729354

I 729356 729359

I 729441 729450

I 729542 729658

I 729757 729886

I 729936 730007

I 739974 739979

I 741186 741200

I 747960 747964

I 749311 749448

I 749551 749583

I 749647 749686

I 752036 752090

I 755659 755714

I 765745 765786

I 765836 765868

I 767863 767879

I 767972 768075

I 769853 769869

I 770074 770179

I 779204 779211

I 790195 790197

I 825912 825913

I 829106 829130

I 829227 829271

I 830594 830608

I 833487 833519

I 833613 833852

I 833942 833974

I 834067 834309

I 836726 836784

I 839377 839421

I 845245 845269

I 854820 854875

I 854973 855254

I 855465 855477

I 856392 856402

I 857455 857741

I 857840 857893

I 859522 859565

I 859638 859823

I 859981 860046

I 862898 862899

I 863612 863624

I 867849 867911

I 868141 868145

I 868359 868417

I 869558 869627

I 869782 869971

I 870059 870093

I 888718 888792

I 889477 889518

I 892988 893020

I 893252 893629

I 893716 893845

I 893921 894076

I 898721 898818

I 899782 900079

I 900143 900443

I 902063 902073

I 904907 904959

I 905348 905370

I 905544 905614

I 908520 908572

I 908663 908693

I 908771 908777

I 909560 909566

I 915118 915367

I 915492 915731

I 923570 923801

I 923859 923998

I 924088 924309

I 924400 924435

I 924590 924606

I 924689 924720

I 924780 924892

I 924970 925001

I 930141 930155

I 930488 930644

I 930814 930960

I 931732 932897

I 933359 933374

I 933559 933639

I 933687 934712

I 942790 943013

I 946830 946841

I 953944 953945

I 958178 958180

I 958263 958268

I 967174 967218

I 967392 967405

I 972259 972296

I 972539 972729

I 973978 974210

I 976089 976129

I 976369 976559

I 979939 979955

I 983939 983949

I 1001383 1001426

I 1007725 1007777

I 1012120 1012131

I 1012634 1012640

I 1012715 1012859

I 1012962 1013132

I 1013334 1013344

I 1013423 1013469

I 1015465 1015494

I 1015578 1015583

I 1017755 1017768

I 1019330 1019340

I 1021435 1021473

I 1022733 1022772

I 1025476 1025513

I 1025594 1025613

I 1025706 1025726

I 1025845 1025857

I 1025984 1026103

I 1026831 1027063

I 1027159 1027251

I 1027333 1027429

I 1027512 1027594

I 1027686 1027795

I 1027868 1027977

I 1028056 1028110

I 1028322 1028323

I 1028449 1028580

I 1031592 1031660

I 1043071 1043119

I 1045166 1045372

I 1045457 1045485

I 1045730 1045740

I 1045844 1045858

I 1045920 1046171

I 1050169 1050184

I 1053013 1053063

I 1056787 1056803

I 1061070 1061074

I 1065445 1065461

I 1076641 1076648

I 1077996 1078025

I 1079629 1079674

I 1079834 1079843

I 1080021 1080083

I 1080178 1080205

I 1080296 1080369

I 1080488 1080609

I 1080705 1080773

I 1080839 1080867

I 1080957 1081214

I 1081294 1081375

I 1081473 1081738

I 1081865 1081891

I 1081983 1082246

I 1082550 1082597

I 1083368 1083381

I 1083537 1083556

I 1086324 1086330

I 1087061 1087091

I 1095278 1095360

I 1099639 1099661

I 1114381 1114386

I 1116438 1116484

I 1116945 1116954

I 1117015 1117025

I 1117098 1117107

I 1117169 1118825

I 1118880 1119225

I 1119473 1119633

I 1119842 1120139

I 1120248 1122034

I 1122125 1122368

I 1122423 1123238

I 1123314 1123495

I 1123571 1123890

I 1123961 1124031

I 1124092 1124340

I 1124631 1124639

I 1124697 1124698

I 1125846 1125859

I 1127591 1127597

I 1134122 1135065

I 1139887 1139907

I 1140051 1140100

I 1142689 1142734

I 1143791 1143799

I 1143934 1144114

I 1144253 1144438

I 1144571 1144582

I 1144792 1144840

I 1145192 1145270

I 1145345 1145391

I 1145480 1145481

I 1145482 1145483

I 1149173 1149178

I 1149484 1149492

I 1149608 1149618

I 1149731 1149796

I 1151284 1151370

I 1151476 1151513

I 1151668 1151810

I 1151897 1151995

I 1154925 1154927

I 1155062 1155076

I 1158597 1158598

I 1158694 1158733

I 1158825 1158987

I 1159239 1159268

I 1159466 1159504

I 1160198 1160215

I 1160502 1160552

I 1160752 1160761

I 1161133 1161164

I 1161278 1161389

I 1163359 1163385

I 1163477 1163506

I 1163595 1163597

I 1169299 1169305

I 1169394 1169481

I 1171365 1171405

I 1171612 1171617

I 1171760 1171768

I 1171994 1172049

I 1173006 1173057

I 1173278 1173289

I 1173438 1173441

I 1174464 1174503

I 1175137 1175172

I 1177193 1177222

I 1177274 1177275

I 1177348 1177356

I 1179594 1179630

I 1179773 1179809

I 1181928 1181981

I 1184644 1184702

I 1185627 1185637

I 1185982 1186219

I 1186315 1186368

I 1186462 1186491

I 1186559 1186576

I 1194681 1194691

I 1194832 1194852

I 1197799 1197800

I 1197801 1197802

I 1197907 1197996

I 1198177 1198230

I 1198469 1198473

I 1199352 1199499

I 1199591 1199620

I 1199678 1199760

I 1200253 1200258

I 1202496 1202604

I 1202776 1202787

I 1202850 1202868

I 1202959 1202962

I 1206534 1206614

I 1206710 1206756

I 1206805 1206932

I 1207092 1207217

I 1209359 1209387

I 1209516 1209733

I 1210041 1210137

I 1210208 1210459

I 1215027 1215067

I 1216590 1216634

I 1216781 1216816

I 1216888 1216892

I 1218489 1218532

I 1219957 1219960

I 1220047 1220058

I 1220360 1220373

I 1220459 1220462

I 1221495 1221498

I 1232920 1232924

I 1233016 1233049

I 1233466 1233468

I 1235921 1235922

I 1236433 1236437

I 1236541 1236552

I 1236931 1236945

I 1237356 1237366

I 1237745 1237756

I 1237857 1237860

I 1238326 1238373

I 1238533 1238581

I 1241524 1241541

I 1241543 1241544

I 1241652 1241659

I 1243492 1243527

I 1244414 1244428

I 1244792 1244805

I 1245456 1245489

I 1245579 1245593

I 1245693 1245720

I 1245766 1245872

I 1246112 1246145

I 1249567 1249734

I 1249824 1249827

I 1249921 1249948

I 1250038 1250084

I 1252361 1252463

I 1254052 1254106

I 1255091 1255151

I 1258904 1258909

I 1259396 1259409

I 1276988 1276989

I 1277057 1277063

I 1281156 1281183

I 1281562 1281592

I 1283222 1283254

I 1286076 1286354

I 1286447 1286728

I 1286820 1286876

I 1297382 1297407

I 1297466 1297474

I 1306246 1306260

I 1306739 1306787

I 1311372 1311395

I 1311602 1311623

I 1314704 1314724

I 1314941 1314960

I 1317531 1317936

I 1318023 1318162

I 1318333 1318352

I 1318404 1318506

I 1318594 1318694

I 1318746 1318791

I 1318981 1319254

I 1329480 1329669

I 1331377 1331632

I 1331768 1332032

I 1333547 1333555

I 1337558 1337568

I 1339067 1339076

I 1347723 1347730

I 1347857 1347943

I 1348090 1348140

I 1348358 1348392

I 1355938 1355981

I 1356872 1357031

I 1357277 1357432

I 1358811 1358884

I 1359135 1359157

I 1360859 1360864

I 1361016 1361029

I 1361939 1361958

I 1362409 1362492

I 1362777 1362834

I 1364153 1364171

I 1364281 1364310

I 1364520 1364609

I 1369065 1369147

I 1369274 1369447

I 1369553 1369684

I 1369768 1369770

I 1371416 1371476

I 1371528 1372227

I 1372319 1372703

I 1372774 1373386

I 1373503 1373628

I 1376241 1376356

I 1376489 1376512

I 1376603 1376651

I 1376796 1376949

I 1377627 1377662

I 1377990 1378123

I 1381635 1381813

I 1381908 1381971

I 1382076 1382139

I 1382233 1382393

I 1387192 1387282

I 1388506 1388537

I 1388594 1388737

I 1388932 1389017

I 1389140 1389163

I 1399591 1399852

I 1399985 1400040

I 1400116 1400119

I 1400194 1400289

I 1401701 1401718

I 1404934 1404940

I 1407402 1407409

I 1408751 1408850

I 1409145 1409248

I 1409339 1409354

I 1409915 1409943

I 1410160 1410168

I 1410462 1410550

I 1410645 1410669

I 1410779 1410793

I 1410896 1411006

I 1413291 1413358

I 1413554 1413622

I 1414769 1414782

I 1418367 1418396

I 1418459 1418467

I 1418613 1418706

I 1419521 1419545

I 1419632 1419711

I 1419760 1419892

I 1420055 1420124

I 1422210 1422285

I 1422425 1422436

I 1423843 1423853

I 1423911 1423919

I 1425998 1426084

I 1426342 1426355

I 1426460 1426551

I 1428941 1428971

I 1429106 1429169

I 1429256 1429270

I 1429478 1429558

I 1429653 1429680

I 1429771 1430133

I 1430258 1430305

I 1430609 1430610

I 1430806 1430819

I 1434618 1434621

I 1434798 1434832

I 1437212 1437309

I 1437872 1437925

I 1438028 1438072

I 1438197 1438301

I 1438592 1438648

I 1438740 1438967

I 1439187 1439411

I 1439507 1439559

I 1439871 1439965

I 1440137 1440142

I 1440246 1440257

I 1440313 1440369

I 1446169 1446175

I 1453713 1453936

I 1458722 1458739

I 1462247 1462426

I 1462494 1463187

I 1468436 1468454

I 1471702 1471917

I 1472140 1472174

I 1472538 1472645

I 1472757 1473327

I 1473410 1473548

I 1473835 1473929

I 1474030 1474134

I 1474219 1474329

I 1474451 1474504

I 1474593 1474773

I 1476095 1476182

I 1480786 1480828

I 1485730 1485745

I 1491603 1491740

I 1491882 1492016

I 1492679 1492710

I 1492838 1492866

I 1493725 1493831

I 1494113 1494158

I 1494281 1494292

I 1494381 1494427

I 1496333 1496343

I 1496653 1496670

I 1497463 1497474

I 1497868 1497896

I 1498317 1498329

I 1498506 1498591

I 1500121 1500143

I 1500219 1500238

I 1505026 1505079

I 1505739 1505781

I 1506019 1506034

I 1506194 1506319

I 1507666 1507674

I 1508129 1508144

I 1508403 1508418

I 1508738 1508744

I 1508827 1508846

I 1509133 1509262

I 1509438 1509564

I 1511125 1511136

I 1512293 1512307

I 1515356 1515482

I 1515899 1515905

I 1516009 1516020

I 1519495 1519590

I 1519839 1519935

I 1520672 1520682

I 1520933 1520946

I 1521403 1521624

I 1521689 1521857

I 1522064 1522109

I 1524917 1524984

I 1525101 1525106

I 1525305 1525334

I 1525383 1525400

I 1527525 1527576

I 1530692 1530723

I 1530863 1531003

I 1531175 1531268

I 1531480 1531482

I 1534521 1535182

I 1535274 1535342

I 1535436 1535718

I 1542160 1542169

I 1542618 1542677

I 1543161 1543222

I 1546383 1546436

I 1546520 1546576

I 1547707 1547712

I 1550908 1551254

I 1555396 1555400

I 1556812 1556826

I 1559817 1559873

I 1560007 1560061

I 1561688 1562140

I 1562233 1562392

I 1562485 1562557

I 1564892 1564923

I 1565862 1566051

I 1566321 1566537

I 1569551 1569672

I 1569764 1569811

I 1570076 1570229

I 1570935 1571016

I 1571160 1571169

I 1571694 1571704

I 1572180 1572270

I 1572643 1572649

I 1573245 1573246

I 1573247 1573249

I 1573499 1573514

I 1580473 1580497

I 1581683 1581694

I 1587658 1587672

I 1587876 1587898

I 1591924 1591964

I 1593084 1593094

I 1593630 1594295

I 1597149 1597168

I 1597245 1597251

I 1598240 1598264

I 1599279 1599309

I 1599522 1599536

I 1608508 1608532

I 1609286 1609308

I 1612738 1612802

I 1613053 1613070

I 1613942 1613991

I 1614119 1614341

I 1614433 1614530

I 1614598 1614657

I 1617087 1617204

I 1617477 1617588

I 1619264 1619266

I 1619267 1619269

I 1619415 1619416

I 1622994 1623010

I 1624018 1624059

I 1626927 1626933

I 1629794 1629813

I 1630121 1630138

I 1632014 1632273

I 1632422 1632445

I 1632577 1632597

I 1635284 1635285

I 1652347 1652366

I 1652429 1652434

I 1652576 1652630

I 1658480 1658519

I 1658569 1658570

I 1658734 1660392

I 1661659 1662166

I 1662236 1662264

I 1662328 1662825

I 1662918 1663044

I 1663933 1663986

I 1664139 1664163

I 1664255 1664272

I 1664524 1664604

I 1664687 1664689

I 1664780 1664806

I 1665161 1665256

I 1670621 1670644

I 1682426 1682443

I 1686773 1686794

I 1687335 1687342

I 1687393 1687464

I 1697333 1697369

I 1697526 1697624

I 1700428 1700440

I 1706810 1706825

I 1711772 1711783

I 1712450 1712529

I 1717201 1717206

I 1723511 1724680

I 1725500 1725524

I 1741852 1741894

I 1741975 1742036

I 1742128 1742191

I 1745049 1745088

I 1746571 1746798

I 1746847 1747078

I 1747194 1747330

I 1747543 1747556

I 1748937 1748956

I 1749021 1749028

I 1750386 1750394

I 1751968 1752027

I 1752115 1752202

I 1752339 1752436

I 1755691 1755714

I 1755774 1756093

I 1756166 1757088

I 1757187 1757550

I 1757632 1757831

I 1757879 1758045

I 1758132 1758705

I 1760955 1761229

I 1761364 1761629

I 1761848 1761859

I 1764237 1764316

I 1767438 1767518

I 1772000 1772187

I 1773817 1773840

I 1773948 1773955

I 1774386 1774414

I 1774504 1774514

I 1778097 1778103

I 1783131 1783361

I 1784337 1784448

I 1784507 1784658

I 1787522 1787584

I 1787642 1787703

I 1795783 1795802

I 1798126 1798188

I 1800726 1800737

I 1803116 1803141

I 1803192 1803194

I 1805025 1805042

I 1812130 1812177

I 1812242 1812285

I 1813745 1813746

I 1816649 1816785

I 1821746 1821750

I 1824624 1824629

I 1824711 1824731

I 1825026 1825083

I 1825229 1825240

I 1825633 1825732

I 1826076 1826189

I 1831788 1831886

I 1847745 1847756

I 1847957 1847959

I 1848756 1848782

I 1850714 1850723

I 1852151 1852174

I 1852526 1852529

I 1852614 1852632

I 1852946 1852949

I 1853196 1853212

I 1854269 1854292

I 1855047 1855055

I 1863610 1863615

I 1866270 1866280

I 1866990 1867011

I 1868688 1868692

I 1868893 1869006

I 1879285 1879300

I 1887037 1887094

I 1887275 1888592

I 1888866 1890148

I 1890206 1890228

I 1890389 1890499

I 1891374 1891407

I 1895916 1895956

I 1896163 1896260

I 1899675 1899867

I 1899992 1900014

I 1900172 1900234

I 1900344 1900497

I 1900587 1900864

I 1901359 1901443

I 1901758 1901766

I 1903560 1903565

I 1903748 1903756

I 1913043 1913050

I 1920213 1920412

I 1920472 1920579

I 1920687 1920769

I 1920864 1921062

I 1921198 1921201

I 1921258 1921523

I 1921646 1922032

I 1922194 1922211

I 1923894 1923897

I 1925639 1925660

I 1926708 1927133

I 1931333 1931343

I 1931439 1931463

I 1932655 1932672

I 1933280 1933340

I 1934006 1934075

I 1934170 1934186

I 1935299 1935502

I 1935578 1935615

I 1935701 1936033

I 1936092 1936318

I 1936483 1936754

I 1936845 1936852

I 1936936 1936982

I 1937067 1937303

I 1937360 1937414

I 1937506 1937515

I 1937599 1937851

I 1937946 1937953

I 1938186 1938192

I 1939535 1939739

I 1939796 1939808

I 1939906 1939965

I 1940215 1940252

I 1940408 1940437

I 1940521 1940597

I 1940697 1940735

I 1946802 1946822

I 1947580 1947599

I 1948917 1948932

I 1954286 1954307

I 1954468 1954504

I 1968957 1969063

I 1969113 1969611

I 1971124 1971179

I 1975161 1975297

I 1975382 1975537

I 1975678 1975704

I 1975791 1975830

I 1975915 1976036

I 1976126 1976431

I 1976594 1976959

I 1977051 1977193

I 1977280 1977410

I 1977579 1977580

I 1979141 1979147

I 1980022 1980053

I 1980151 1980411

I 1980562 1980614

I 1980982 1981012

I 1982674 1982704

I 1982980 1983017

I 1983770 1983771

I 1984577 1984587

I 1987567 1987621

I 1992022 1992102

I 1992160 1992261

I 1992327 1992410

I 1993720 1993745

I 1994095 1994136

I 2002746 2002758

I 2002945 2003432

I 2004443 2004523

I 2005246 2005272

I 2012126 2012268

I 2012405 2012435

I 2014308 2014314

I 2017858 2017938

I 2018436 2018517

I 2021108 2021110

I 2021585 2021722

I 2021928 2021930

I 2021931 2021934

I 2023655 2023679

I 2023919 2023949

I 2024293 2024323

I 2025782 2025800

I 2030603 2030646

I 2031072 2031152

I 2034052 2034056

I 2035109 2035111

I 2035315 2035324

I 2036160 2037563

I 2037629 2038626

I 2038722 2039333

I 2039429 2039516

I 2039605 2040330

I 2042819 2042825

I 2043226 2043303

I 2044158 2044225

I 2044579 2044627

I 2045370 2045396

I 2045466 2045484

I 2045575 2045756

I 2045924 2045963

I 2046123 2046265

I 2046599 2047092

I 2047360 2047880

I 2048094 2048115

I 2048205 2048299

I 2048469 2048576

I 2050201 2050252

I 2050334 2050732

I 2050829 2051867

I 2052005 2052471

I 2052624 2052737

I 2052829 2053016

I 2053261 2053305

I 2053391 2053497

I 2053570 2053674

I 2053762 2054510

I 2054602 2054716

I 2056368 2056375

I 2056471 2056824

I 2060152 2060503

I 2060599 2060606

I 2060860 2060966

I 2061087 2061101

I 2061192 2061258

I 2066407 2066413

I 2067447 2067470

I 2072887 2072893

I 2072894 2072895

I 2075546 2075550

I 2075756 2075762

I 2082471 2082490

I 2094519 2094549

I 2095538 2095642

I 2095873 2095892

I 2098041 2098051

I 2100210 2100217

I 2103487 2103489

I 2103645 2103702

I 2104266 2104444

I 2104574 2104591

I 2104793 2104808

I 2104919 2104939

I 2105034 2105037

I 2105965 2105975

I 2111104 2111135

I 2112964 2113004

I 2120480 2120530

I 2122278 2122320

I 2122441 2122618

I 2122755 2122800

I 2124918 2124974

I 2129053 2129429

I 2129613 2129645

I 2129767 2129791

I 2130311 2130357

I 2130420 2130551

I 2130682 2130815

I 2130910 2131158

I 2131251 2131788

I 2132057 2132111

I 2132208 2132501

I 2132580 2132682

I 2132775 2132808

I 2132896 2132948

I 2133627 2133647

I 2137362 2137370

I 2138034 2138067

I 2141650 2141660

I 2142197 2142199

I 2146915 2147035

I 2147225 2147515

I 2148068 2148075

I 2148166 2148197

I 2151125 2151140

I 2151507 2151519

I 2152278 2152289

I 2152440 2152458

I 2162337 2162346

I 2162602 2162611

I 2168704 2168835

I 2168908 2169043

I 2169107 2169242

I 2171187 2171241

I 2171429 2171467

I 2171518 2171882

I 2172654 2172671

I 2172772 2172801

I 2175379 2175559

I 2175691 2175824

I 2179644 2179699

I 2183886 2183913

I 2191882 2191913

I 2197473 2197530

I 2198978 2199040

I 2199137 2199138

I 2199584 2199588

I 2199706 2199724

I 2212961 2213291

I 2213361 2213393

I 2213579 2213644

I 2213762 2214064

I 2214128 2214195

I 2214265 2214286

I 2214436 2214631

I 2214706 2214878

I 2214970 2215020

I 2215111 2215216

I 2215330 2215434

I 2215531 2215828

I 2221200 2221234

I 2221303 2221348

I 2248667 2248688

I 2248860 2248862

I 2248863 2248866

I 2255017 2255440

I 2255499 2255693

I 2255781 2255808

I 2263675 2263691

I 2263845 2263860

I 2275440 2275474

I 2279136 2279137

I 2279138 2279139

I 2279715 2279722

I 2280145 2280181

I 2282353 2282356

I 2282450 2282490

I 2282563 2282588

I 2282638 2282708

I 2295698 2295732

I 2296693 2296756

I 2296996 2296998

I 2298833 2298877

I 2322714 2322745

I 2322867 2322929

I 2323596 2323600

I 2323923 2324012

I 2330272 2330387

I 2331203 2331220

I 2340492 2340594

I 2340687 2340823

I 2340916 2341044

I 2341363 2341492

I 2341590 2341721

I 2341816 2341918

I 2343428 2343492

I 2344396 2344434

I 2356359 2356406

I 2356511 2356585

I 2360996 2360997

I 2365465 2365482

I 2365572 2365657

I 2365749 2365944

I 2366025 2366105

I 2366265 2366318

I 2366407 2366416

I 2366557 2366634

I 2366724 2366771

I 2366835 2366873

I 2367004 2367116

I 2367174 2367275

I 2367331 2367610

I 2367668 2367711

I 2367771 2367810

I 2367879 2367969

I 2368194 2368300

I 2368439 2368582

I 2368640 2368668

I 2371720 2371775

I 2371839 2371869

I 2372010 2372022

I 2372154 2372281

I 2372486 2372504

I 2373869 2373875

I 2380712 2380782

I 2380842 2380912

I 2385492 2385506

I 2385605 2385670

I 2385832 2385859

I 2385923 2385930

I 2386696 2386794

I 2387324 2387364

I 2394759 2394818

I 2394911 2394937

I 2399556 2399650

I 2399819 2399912

I 2406765 2406920

I 2407098 2407266

I 2410635 2410655

I 2410734 2410907

I 2410994 2411051

I 2411148 2411164

I 2411360 2411532

I 2411589 2411614

I 2413829 2413841

I 2423471 2423515

I 2424595 2424616

I 2425940 2426004

I 2426708 2426790

I 2426921 2426995

I 2427067 2427124

I 2427227 2427322

I 2427776 2427796

I 2427886 2427999

I 2429458 2429493

I 2430636 2430733

I 2430811 2430904

I 2441782 2441788

I 2449127 2449215

I 2449319 2449339

I 2449462 2449478

I 2449600 2449629

I 2451612 2451654

I 2459200 2459395

I 2459615 2459623

I 2459709 2459790

I 2459865 2459871

I 2460198 2460420

I 2461342 2461395

I 2461445 2461490

I 2461547 2461756

I 2461853 2461900

I 2462017 2462080

I 2462204 2462489

I 2466917 2468064

I 2478945 2478958

I 2481313 2481319

I 2486238 2486294

I 2486579 2486603

I 2495552 2495645

I 2495739 2495763

I 2496261 2496289

I 2496383 2496473

I 2499675 2499677

I 2499679 2499682

I 2506956 2507068

I 2508473 2508636

I 2508719 2508780

I 2508874 2508903

I 2508992 2509035

I 2509303 2509304

I 2509424 2509450

I 2509557 2509617

I 2509900 2509964

I 2515623 2515699

I 2542787 2542806

I 2542962 2543482

I 2543535 2543952

I 2544234 2544235

I 2550285 2550297

I 2554192 2554223

I 2554303 2554340

I 2554498 2554507

I 2554600 2554616

I 2554708 2554835

I 2567666 2567736

I 2567831 2567868

I 2579685 2579705

I 2582250 2582292

I 2584495 2584503

I 2586945 2586950

I 2587034 2587154

I 2610806 2610819

I 2611129 2611140

I 2617881 2617900

I 2618508 2618513

I 2626072 2626421

I 2626514 2626611

I 2626742 2626769

I 2626894 2626899

I 2631332 2631340

I 2631464 2631490

I 2632417 2632435

I 2633137 2633190

I 2633285 2633634

I 2633802 2633812

I 2635077 2635283

I 2635411 2635419

I 2635506 2635613

I 2635694 2635812

I 2635909 2636102

I 2636225 2636337

I 2636420 2636443

I 2636723 2636741

I 2637152 2637171

I 2637269 2637272

I 2637404 2637513

I 2638323 2638384

I 2638472 2638503

I 2641073 2641113

I 2641510 2641513

I 2642413 2642453

I 2644442 2644473

I 2644662 2644693

I 2650658 2650765

I 2651805 2651942

I 2691459 2691545

I 2691632 2691862

I 2694619 2694632

I 2699962 2699986

I 2700081 2700214

I 2700310 2700377

I 2700926 2700951

I 2701133 2701860

I 2702049 2702069

I 2702162 2702163

I 2715873 2715949

I 2716608 2716672

I 2719451 2719459

I 2719552 2719573

I 2719668 2719713

I 2722187 2722203

I 2724545 2724660

I 2724816 2724855

I 2729623 2729640

I 2733554 2733618

I 2733715 2733949

I 2734512 2734745

I 2734839 2734912

I 2769423 2769433

I 2772532 2772708

I 2773070 2773082

I 2773278 2773497

I 2773619 2773669

I 2778268 2778428

I 2784707 2784723

I 2785020 2785110

I 2787072 2787161

I 2787455 2787467

I 2827493 2827514

I 2832651 2832664

I 2843177 2843707

I 2854063 2854083

I 2854270 2855001

I 2855181 2855207

I 2855787 2855856

I 2855951 2856087

I 2856182 2856201

I 2865998 2866086

I 2873445 2873534

I 2873627 2873795

I 2903268 2903301

I 2904935 2904979

I 2905088 2905126

I 2905232 2905286

I 2905455 2905488

I 2906367 2906373

I 2906467 2906472

I 2907899 2907964

I 2908155 2908214

I 2908624 2909134

I 2909220 2909333

I 2909419 2909454

I 2909540 2909562

I 2909642 2909916

I 2910421 2910539

I 2910959 2911035

I 2911995 2912058

I 2912182 2912201

I 2913802 2913823

I 2913917 2913925

I 2914029 2914040

I 2914273 2914290

I 2914380 2914406

I 2916582 2916592

I 2917381 2917463

I 2917784 2917828

I 2918033 2918041

I 2918180 2918215

I 2918626 2918636

I 2918761 2918855

I 2918948 2919052

I 2919524 2919672

I 2921079 2921087

I 2923004 2923006

I 2923151 2923153

I 2923241 2923251

I 2923473 2923475

I 2923854 2923882

I 2923979 2923998

I 2924218 2924264

I 2924358 2924403

I 2924517 2924585

I 2924852 2924872

I 2927149 2927167

I 2927259 2927272

I 2927408 2927429

I 2930816 2930830

I 2933198 2933225

I 2933654 2933657

I 2938858 2939274

I 2942325 2942343

I 2942645 2942670

I 2944791 2944894

I 2944979 2945061

I 2967059 2967084

I 2989010 2989069

I 2989330 2989338

I 2989460 2989544

I 2991775 2991783

I 2996235 2996276

I 2997100 2997131

I 2997279 2997289

I 3000495 3000525

I 3000650 3000682

I 3002096 3002202

I 3013870 3013907

I 3016673 3016688

I 3016767 3016768

I 3016770 3016861

I 3017115 3017145

I 3017348 3017499

I 3027113 3027121

I 3032181 3032183

I 3034216 3034218

I 3036072 3036098

I 3036342 3036464

I 3036637 3036651

I 3036839 3036869

I 3037263 3037342

I 3037870 3037888

I 3038001 3038073

I 3040734 3040813

I 3041640 3041643

I 3052400 3052423

I 3052585 3052602

I 3053105 3053106

I 3063506 3063572

I 3063726 3063849

I 3063929 3064556

I 3073263 3073396

I 3073462 3073486

I 3073778 3073831

I 3074316 3074325

I 3079177 3079349

I 3079850 3079865

I 3081016 3081063

I 3087469 3087621

I 3087710 3087864

I 3111641 3111668

I 3111746 3111770

I 3111851 3111858

I 3112085 3112110

I 3112421 3112429

I 3112525 3112683

I 3112779 3112810

I 3112975 3112997

I 3113134 3113160

I 3113256 3113345

I 3114656 3114814

I 3133645 3133825

I 3133914 3133989

I 3134099 3134454

I 3134547 3134574

I 3146861 3146867

I 3146963 3146971

I 3146973 3146974

I 3150430 3150504

I 3151248 3151255

I 3151355 3151356

I 3151546 3151601

I 3153151 3153158

I 3153254 3153286

I 3155678 3155707

I 3156365 3156395

I 3156978 3157012

I 3158579 3158655

I 3171717 3171819

I 3171898 3172096

I 3172234 3172343

I 3181830 3181877

I 3181968 3182007

I 3182257 3182258

I 3182261 3182262

I 3182422 3182425

I 3182518 3182586

I 3197179 3197213

I 3197344 3197355

I 3198170 3198205

I 3198297 3198306

I 3200462 3200518

I 3200665 3200692

I 3200782 3200796

I 3200889 3200972

I 3201345 3201367

I 3201577 3201587

I 3202486 3202508

I 3202639 3202658

I 3202755 3202784

I 3209555 3209622

I 3212426 3212436

I 3213568 3213625

I 3221841 3221852

I 3228864 3228969

I 3229280 3229330

I 3231120 3231132

I 3231222 3231227

I 3231343 3231438

I 3254698 3254707

I 3255178 3255181

I 3258640 3258711

I 3258912 3259013

I 3259077 3259162

I 3259193 3259265

I 3260285 3260286

I 3263719 3263733

I 3270662 3270692

I 3270844 3270899

I 3271069 3271070

I 3271134 3271321

I 3271474 3271600

I 3271694 3271959

I 3272032 3272131

I 3272346 3272356

I 3272514 3272805

I 3276101 3276110

I 3278715 3278735

I 3286892 3286895

I 3294708 3294794

I 3294943 3294952

I 3324283 3324293

I 3325327 3325336

I 3325404 3325412

I 3325663 3325672

I 3325768 3325792

I 3325869 3325909

I 3333347 3333352

I 3335160 3335183

I 3349022 3349033

I 3360213 3360291

I 3360904 3360940

I 3361035 3361109

I 3361199 3361277

I 3361406 3361698

I 3361935 3361951

I 3362118 3362154

I 3362468 3362486

I 3362843 3363127

I 3363221 3363363

I 3363430 3363437

I 3363592 3363599

I 3364052 3364081

I 3364314 3364349

I 3364509 3364537

I 3364630 3364647

I 3364741 3364758

I 3365050 3365123

I 3365324 3365526

I 3365621 3365849

I 3366354 3366363

I 3366417 3366441

I 3366728 3366757

I 3367037 3367209

I 3372515 3372565

I 3390135 3390147

I 3390238 3390475

I 3390533 3391115

I 3391197 3391256

I 3391323 3391748

I 3391798 3392040

I 3392191 3392678

I 3392774 3393025

I 3393153 3393237

I 3393438 3393492

I 3400724 3400747

I 3405028 3405035

I 3405433 3405439

I 3411848 3411865

I 3412038 3412092

I 3412253 3412256

I 3427477 3427506

I 3427791 3427848

I 3428038 3428100

I 3428150 3428226

I 3428418 3428496

I 3428586 3428685

I 3428950 3429098

I 3429191 3429206

I 3441287 3441337

I 3441570 3441623

I 3442714 3442726

I 3442785 3442787

I 3442925 3442981

I 3443137 3443144

I 3443270 3443275

I 3443358 3443401

I 3443532 3443574

I 3443744 3443752

I 3450006 3450008

I 3450221 3450234

I 3453374 3453461

I 3460249 3460334

I 3468976 3468987

I 3469081 3469087

I 3485204 3485423

I 3485496 3487551

I 3487642 3487649

I 3487717 3487728

I 3491110 3491117

I 3491237 3491488

I 3491631 3491942

I 3492103 3492164

I 3492281 3492665

I 3505595 3505602

I 3510889 3510917

I 3511606 3511694

I 3511782 3511894

I 3514874 3514963

I 3515250 3515254

I 3517294 3517319

I 3517612 3517662

I 3523766 3523767

I 3523770 3523772

I 3523952 3524030

I 3524183 3524204

I 3524361 3524433

I 3524603 3524623

I 3524719 3524738

I 3524999 3525050

I 3540246 3540250

I 3545129 3545139

I 3545283 3545289

I 3547744 3547784

I 3548222 3548256

I 3552366 3552494

I 3552762 3552766

I 3552924 3553178

I 3553271 3553409

I 3553551 3553633

I 3553845 3554069

I 3554154 3554267

I 3554359 3554415

I 3554512 3554536

I 3554624 3554665

I 3554809 3554979

I 3555220 3555458

I 3555645 3555665

I 3555845 3555873

I 3556101 3556285

I 3556630 3556874

I 3557058 3557069

I 3557165 3557356

I 3557499 3557613

I 3558428 3558547

I 3561445 3561562

I 3565461 3565566

I 3565643 3565800

I 3566077 3566174

I 3566263 3566265

I 3566356 3566362

I 3566629 3566941

I 3586467 3586513

I 3591444 3591449

I 3591604 3591627

I 3592793 3592801

I 3598416 3598440

I 3609619 3609629

I 3609996 3610028

I 3610679 3610714

I 3619949 3619973

I 3644118 3644149

I 3644233 3644269

I 3644319 3644355

I 3644447 3644480

I 3644576 3644658

I 3664231 3665722

I 3665797 3665867

I 3665936 3666085

I 3682388 3682411

I 3699578 3699606

I 3699696 3699797

I 3699985 3700114

I 3700166 3700244

I 3700338 3700574

I 3700667 3700903

I 3700991 3701029

I 3701707 3701837

I 3701975 3702015

I 3702409 3702444

I 3702645 3702715

I 3702850 3702868

I 3703006 3703177

I 3703616 3703774

I 3703883 3703889

I 3703998 3704083

I 3704213 3704236

I 3704326 3704462

I 3704594 3704613

I 3704705 3704891

I 3704985 3705068

I 3705208 3705418

I 3705468 3705507

I 3707502 3707545

I 3726419 3726445

I 3726815 3726837

I 3727165 3727342

I 3727636 3727805

I 3727901 3727928

I 3728064 3728102

I 3728312 3728383

I 3728607 3728645

I 3728825 3728831

I 3728956 3729014

I 3729125 3729127

I 3729222 3729454

I 3729716 3729804

I 3730003 3730038

I 3730131 3730132

I 3730227 3730440

I 3730535 3730579

I 3730688 3730798

I 3730974 3731001

I 3731455 3731516

I 3731641 3731669

I 3731760 3731801

I 3731986 3732021

I 3732112 3732133

I 3732292 3732344

I 3732440 3732566

I 3732846 3732902

I 3733018 3733033

I 3733238 3733274

I 3733367 3733409

I 3745818 3746425

I 3746926 3747395

I 3761357 3761612

I 3761778 3761850

I 3761934 3762089

I 3762260 3762466

I 3763200 3763890

I 3763941 3764070

I 3764123 3765225

I 3765298 3765891

I 3796925 3796950

I 3836090 3836184

I 3836270 3836437

I 3836574 3836674

I 3838627 3838682

I 3843334 3843496

I 3847416 3847533

I 3847765 3847838

I 3847930 3848341

I 3848432 3848494

I 3848657 3848702

I 3849050 3849135

I 3849230 3849235

I 3849355 3849425

I 3849520 3849536

I 3849865 3849927

I 3850209 3850273

I 3850406 3850495

I 3850974 3851001

I 3851119 3851196

I 3851290 3851393

I 3851473 3851529

I 3851620 3851644

I 3851769 3851808

I 3851899 3851938

I 3851997 3852047

I 3852241 3852407

I 3866877 3866920

I 3896024 3896117

I 3896211 3896536

I 3896714 3896802

I 3896899 3896919

I 3897504 3897508

I 3932826 3932973

I 3933066 3933240

I 3933337 3933366

I 3933459 3933517

I 3933685 3934480

I 3961314 3962428

I 3962885 3963998

I 3964177 3969231

I 3970207 3975261

I 3980252 3980437

I 3980496 3980579

I 3980774 3981015

I 3981099 3981249

I 3981341 3981477

I 3981571 3981593

I 3990393 3990396

I 3990508 3990544

I 3991674 3991679

I 3991777 3991834

I 3997104 3997129

I 3997235 3997268

I 3997735 3997786

I 3997877 3998040

I 3998120 3998262

I 3998356 3998390

I 3998437 3998603

I 4099066 4099089

I 4113153 4113178

I 4113420 4113429

I 4113672 4113688

I 4113927 4113954

I 4115196 4115251

I 4115339 4115380

I 4115760 4115763

I 4115764 4115765

I 4116082 4116148

I 4116497 4116511

I 4116675 4116790

I 4116919 4116954

I 4117080 4117113

I 4117228 4117253

I 4117562 4117601

I 4119240 4119339

I 4119526 4119573

I 4119670 4119798

I 4119865 4120004

I 4120509 4120519

I 4120628 4121118

I 4121210 4121304

I 4121394 4121622

I 4121836 4122038

I 4122148 4122400

I 4122487 4123085

I 4144456 4144512

I 4144749 4144811

I 4144931 4144947

I 4145042 4145050

I 4145149 4145240

I 4145370 4145444

I 4145538 4145541

I 4145818 4145855

I 4145952 4145956

I 4146175 4146177

I 4146460 4146683

I 4146933 4147074

I 4147590 4147678

I 4167893 4167977

I 4168050 4168129

I 4169317 4170112

I 4170282 4170337

I 4170432 4170462

I 4170558 4170729

I 4170825 4170974

I 4185872 4185931

I 4186159 4186168

I 4186810 4186869

I 4186963 4186991

I 4187107 4187196

I 4187328 4187417

I 4187617 4187618

I 4187709 4188254

I 4188395 4188626

I 4188719 4189034

I 4189130 4189767

I 4190352 4190428

I 4190691 4190722

I 4221305 4221348

I 4221440 4221470

I 4236881 4236886

I 4237464 4237481

I 4237578 4237666

I 4237848 4238169

I 4238264 4238362

I 4239564 4239656

I 4239818 4239955

I 4240055 4240190

I 4240279 4240382

I 4240472 4240661

I 4240866 4240925

I 4241140 4241190

I 4276657 4276696

I 4280169 4280273

I 4280420 4280421

I 4280544 4280752

I 4280804 4280966

I 4281222 4281232

I 4281350 4281369

I 4281626 4281689

I 4281776 4284051

I 4284106 4284305

I 4284375 4286041

I 4286091 4286245

I 4286326 4286747

I 4286831 4287242

I 4287313 4287959

I 4288033 4289469

I 4289535 4292329

I 4292397 4293383

I 4293470 4294585

I 4313358 4313408

I 4339180 4339305

I 4339355 4339478

I 4340920 4344359

I 4344455 4345252

I 4345356 4357536

I 4375326 4375494

I 4382262 4382304

I 4382378 4382405

I 4385393 4385421

I 4385598 4385602

I 4417721 4417860

I 4417950 4418017

I 4423429 4423447

I 4456423 4456885

I 4456999 4457263

I 4459254 4459717

I 4459820 4460093

I 4466212 4466237

I 4466363 4466414

I 4477933 4477935

I 4477936 4477939

I 4478022 4478081

I 4494995 4495038

I 4504163 4504176

I 4504234 4504297

I 4537183 4537301

I 4540446 4540533

I 4540585 4540793

I 4540875 4540908

I 4540964 4541103

I 4541151 4541252

I 4541448 4541543

I 4541745 4541773

I 4541823 4542219

I 4542410 4542460

I 4542508 4542591

I 4542641 4542834

I 4543013 4543069

I 4543146 4543214

I 4543331 4543342

I 4543549 4543631

I 4544078 4544236

I 4544311 4544437

I 4544555 4544802

I 4544897 4545358

I 4545443 4545553

I 4545642 4546324

I 4546538 4546807

I 4546893 4547533

I 4547627 4547688

I 4548353 4548437

I 4548572 4548609

I 4572938 4573082

I 4573161 4573324

I 4573393 4573663

I 4573749 4573806

I 4573900 4574006

I 4574097 4574109

I 4574211 4574267

I 4574489 4574494

I 4574496 4574499

I 4574631 4574698

I 4587263 4587339

I 4660381 4660397

I 4661488 4661496

I 4661585 4661633

I 4662309 4662360

I 4663321 4663400

I 4663531 4663537

I 4663679 4663752

I 4663875 4663895

I 4664232 4664249

I 4709597 4709650

I 4715878 4715951

I 4716067 4716072

I 4716277 4716283

I 4716366 4716394

I 4716484 4716553

I 4716641 4716715

I 4717648 4717655

I 4718254 4718298

I 4718389 4719507

I 4752059 4753226

I 4753936 4753958

I 4754069 4754136

I 4754185 4754245

I 4754641 4754703

I 4754845 4754970

I 4755151 4755331

I 4755426 4755436

I 4760931 4761895

I 4761986 4762950

I 4763309 4764423

I 4764518 4764564

I 4765106 4765118

I 4765841 4765880

I 4765950 4765956

I 4765957 4765958

I 4766551 4766577

I 4766897 4766930

I 4767219 4767376

I 4767499 4767622

I 4767940 4767947

I 4768150 4768158

I 4768357 4768386

I 4768459 4768508

I 4769123 4769147

I 4769243 4769346

I 4769586 4769634

I 4779453 4779468

I 4779740 4779784

I 4808595 4808667

I 4814081 4814121

I 4814211 4814289

I 4814383 4814505

I 4814703 4814835

I 4814926 4814992

I 4815084 4815164

I 4815256 4815366

I 4815458 4815823

I 4815897 4815945

I 4816255 4816279

I 4816378 4816588

I 4816743 4816812

I 4816954 4817087

I 4817180 4817202

I 4817324 4817364

I 4958993 4959172

I 4959391 4960237

I 4960399 4960675

I 4960825 4960874

I 4960971 4961098

I 4961151 4961278

I 4961551 4961603

I 4961738 4961776

I 4962004 4962137

I 4978576 4978811

I 4978903 4978979

I 4979128 4979146

I 4979232 4979344

I 4979442 4979608

I 5004501 5004509

I 5004729 5004985

I 5005211 5005263

I 5005565 5005763

I 5006140 5006162

I 5006780 5006806

I 5006947 5006966

I 5014936 5014954

I 5015044 5015061

I 5032266 5032279

I 5040087 5040156

I 5040246 5040308

I 5040391 5040401

I 5040499 5040569

I 5045733 5045737

I 5088763 5088790

I 5088957 5089029

I 5091332 5091346

I 5091813 5091859

I 5091949 5092004

I 5092408 5092437

I 5092488 5092540

I 5122352 5122362

I 5142518 5142682

I 5142777 5143206

I 5143283 5143513

I 5144880 5144893

I 5145187 5145231

I 5145714 5145716

I 5145802 5145908

I 5150473 5150638

I 5150729 5150888

I 5150981 5152528

I 5152631 5152961

I 5153011 5153728

I 5153806 5153881

I 5155412 5155414

I 5157892 5157914

I 5158015 5158247

I 5162665 5162801

I 5163217 5163218

I 5163312 5163384

I 5175804 5175982

I 5176283 5176359

I 5256638 5256746

I 5275430 5275583

I 5275840 5275880

I 5275986 5276064

I 5276180 5276346

I 5276538 5276646

I 5276745 5276811

I 5297092 5297459

I 5297548 5297550

I 5297646 5297773

I 5297827 5297894

I 5297946 5298181

I 5298286 5298356

I 5298538 5298571

I 5335709 5335718

I 5335959 5335970

I 5336061 5336104

I 5336461 5336486

I 5336574 5336580

I 5336750 5336751

I 5368887 5369144

I 5369296 5369350

I 5369444 5369445

I 5369583 5369592

I 5390151 5390160

I 5390253 5390260

I 5452406 5452450

I 5452542 5452587

I 5454249 5454332

I 5454604 5454643

I 5455149 5455151

I 5455579 5455671

I 5456483 5456496

I 5458394 5458405

I 5459368 5459404

I 5459561 5459631

I 5459737 5459752

I 5471090 5471091

I 5471092 5471093

I 5504572 5504618

I 5509533 5510324

I 5510410 5511200

I 5531734 5531746

I 5554429 5554540

I 5555450 5555471

I 5555522 5555557

I 5555673 5555717

I 5556070 5556096

I 5556187 5556210

I 5562191 5562205

I 5562294 5562313

I 5563108 5563187

I 5584912 5585404

I 5585575 5585587

I 5593983 5594082

I 5594215 5594233

I 5594328 5594478

I 5594635 5594646

I 5594766 5594897

I 5594956 5595021

I 5595110 5595201

I 5621498 5621541

I 5621631 5621654

I 5621726 5621752

I 5638063 5638234

I 5638300 5638420

I 5638512 5638513

I 5638625 5638641

I 5638792 5638797

I 5639391 5639415

I 5639600 5640026

I 5640166 5640357

I 5640489 5640958

I 5641047 5641140

I 5641284 5641368

I 5641457 5641533

I 5641625 5641891

I 5641975 5642002

I 5642120 5642219

I 5642288 5642369

I 5660050 5660077

I 5678788 5678870

I 5707946 5707998

I 5708143 5708174

I 5708265 5708331

I 5708421 5708444

I 5708592 5708657

I 5708764 5708793

I 5708888 5709128

I 5709226 5709281

I 5709423 5709485

I 5745979 5746159

I 5762920 5762924

I 5763013 5763090

I 5772192 5772315

I 5772401 5772421

I 5779615 5779746

I 5780044 5780138

I 5780204 5780280

I 5841301 5841350

I 5841439 5841604

I 5863780 5863820

I 5906831 5907010

I 5908128 5908296

I 5911457 5911634

I 5913394 5913432

I 5913536 5913781

I 5914138 5914152

I 5914436 5914624

I 5914881 5915071

I 5915167 5915182

I 5961078 5961098

I 5961545 5961563

I 5961739 5961746

I 5974745 5975120

I 5975262 5975455

I 5975530 5975579

I 5975655 5976072

I 5976167 5976310

I 5976397 5976421

I 5976504 5976807

I 5976894 5976992

I 5977170 5977179

I 5977269 5977290

I 5977375 5977414

I 5977549 5977719

I 5978851 5980135

I 5980291 5980395

I 5980577 5980579

I 5980940 5980993

I 5981086 5981173

I 5981663 5981664

I 5981816 5982934

I 5983051 5983080

I 6051691 6051725

I 6051816 6052156

I 6052238 6052280

I 6052365 6052374

I 6052464 6052493

I 6052582 6053213

I 6055277 6055384

I 6057680 6057787

I 6086142 6086182

I 6086320 6086352

I 6086443 6086456

I 6088042 6088486

I 6088675 6088796

I 6088913 6088926

I 6089015 6089359

I 6099021 6099339

I 6115304 6115610

I 6115700 6116106

I 6116223 6116261

I 6160155 6160162

I 6160542 6160600

I 6160697 6160740

I 6160837 6160915

I 6161112 6161147

I 6161421 6161496

I 6161645 6161655

I 6181834 6182017

I 6182107 6182132

I 6182219 6182337

I 6182477 6182555

I 6195151 6195176

I 6195261 6195314

I 6223364 6223410

I 6223607 6223661

I 6223747 6223786

I 6224189 6224248

I 6224590 6224598

I 6224802 6224898

I 6225024 6225054

I 6225306 6225309

I 6225403 6225446

I 6225642 6225673

I 6225767 6225809

I 6225903 6225908

I 6226044 6226140

I 6226237 6226298

I 6226408 6226460

I 6226772 6227061

I 6227151 6227195

I 6227485 6227613

I 6228022 6228065

I 6228484 6228520

I 6228611 6228841

I 6228933 6228958

I 6229247 6229264

I 6229373 6229400

I 6229535 6229549

I 6229645 6229783

I 6230115 6230351

I 6230508 6230553

I 6230786 6230862

I 6231603 6232211

I 6234625 6235232

I 6235297 6235357

I 6265033 6265039

I 6274909 6275225

I 6305739 6305742

I 6306242 6306373

I 6307328 6307469

I 6307930 6307935

I 6325548 6325599

I 6329644 6329690

I 6330136 6330196

I 6350383 6350497

I 6350589 6350636

I 6350730 6350757

I 6354474 6354510

I 6354648 6354671

I 6392850 6392862

I 6392949 6393005

I 6393278 6393323

I 6393411 6393546

I 6414975 6415042

I 6436609 6436633

I 6449905 6449908

I 6450439 6450661

I 6450896 6450905

I 6451255 6451421

I 6451555 6451595

I 6466537 6466594

I 6466682 6466683

I 6473565 6473573

I 6473669 6473744

I 6473861 6473904

I 6500071 6500102

I 6500373 6500423

I 6500558 6500583

I 6502200 6502204

I 6502716 6502779

I 6502876 6502920

I 6503015 6503093

I 6503292 6503325

I 6503610 6503686

I 6503836 6503840

I 6510543 6510552

I 6514324 6514338

I 6514443 6514446

I 6514527 6514558

I 6516322 6516370

I 6570836 6570846

I 6573444 6573607

I 6590288 6590712

I 6590801 6590919

I 6591077 6591095

I 6591138 6591614

I 6591702 6591757

I 6603265 6603333

I 6603478 6603489

I 6603572 6603615

I 6603705 6603925

I 6604175 6604201

I 6604289 6604431

I 6604555 6604603

I 6647932 6648147

I 6648197 6648293

I 6648360 6648434

I 6648529 6648565

I 6648661 6648705

I 6649185 6649209

I 6658089 6658110

I 6665708 6665790

I 6682168 6682177

I 6697932 6697968

I 6722456 6722512

I 6722596 6722632

I 6723224 6724228

I 6724315 6724477

I 6738674 6738806

I 6738895 6738978

I 6739144 6739145

I 6739230 6739292

I 6762191 6762374

I 6762802 6762977

I 6771008 6771009

I 6772006 6772309

I 6772389 6772859

I 6772942 6773720

I 6773770 6773911

I 6854462 6854592

I 6856407 6856456

I 6898152 6898154

I 6898248 6898453

I 6898540 6898667

I 6931845 6932364

I 6932517 6932538

I 6932634 6932639

I 6932963 6933491

I 6933593 6933634

I 6933716 6933743

I 6949120 6949130

I 7002539 7002557

I 7003010 7003328

I 7004213 7004221

I 7004316 7004396

I 7004518 7004537

I 7004628 7004655

I 7004851 7004936

I 7005151 7005186

I 7018296 7018439

I 7018569 7018596

I 7019428 7019443

I 7052061 7052092

I 7053217 7053227

I 7053323 7053355

I 7086390 7086401

I 7098352 7098380

I 7098488 7098638

I 7098845 7098959

I 7099483 7099585

I 7099688 7099716

I 7178119 7178435

I 7186268 7186322

I 7237662 7237664

I 7245406 7245409

I 7286000 7286110

I 7324859 7324897

I 7324976 7324982

I 7330863 7330979

I 7331054 7331062

I 7336408 7336545

I 7336719 7336728

I 7336890 7337241

I 7337330 7337419

I 7401511 7401666

I 7401757 7402145

I 7402241 7402349

I 7402444 7402761

I 7403285 7403604

I 7403698 7403807

I 7403903 7404291

I 7404385 7404536

I 7431818 7431852

I 7431928 7431953

I 7432395 7432415

I 7432713 7432723

I 7469659 7469843

I 7536303 7536801

I 7536899 7537398

I 7571545 7571701

I 7571899 7572027

I 7572138 7572398

I 7572516 7572559

I 7572685 7572727

I 7572788 7572800

I 7572858 7572876

I 7573014 7573095

I 7646305 7646327

I 7653002 7653024

I 7665849 7665873

I 7699088 7699136

I 7718991 7719682

I 7719762 7719790

I 7719923 7720219

I 7720447 7720544

I 7720696 7720781

I 7721147 7721258

I 7721326 7721364

I 7721456 7721467

I 7721557 7721612

I 7721760 7721819

I 7721911 7722121

I 7722244 7722282

I 7722338 7722432

I 7722619 7722647

I 7722891 7722992

I 7723090 7723118

I 7723260 7723286

I 7740853 7740874

I 7740998 7741011

I 7741055 7741102

I 7741236 7741282

I 7741459 7741488

I 7741542 7741618

I 7741936 7742035

I 7742146 7742148

I 7742481 7742526

I 7742617 7742825

I 7760381 7760398

I 7776383 7779472

I 7779562 7779740

I 7779833 7779929

I 7780470 7780483

I 7780665 7780676

I 7781597 7781697

I 7781782 7781812

I 7781914 7781938

I 7782035 7785584

I 7795446 7795484

I 7842719 7844710

I 7844804 7846150

I 7868207 7868278

I 7868428 7868456

I 7868588 7868792

I 7868890 7868961

I 7869207 7869628

I 7869775 7869889

I 7919242 7919398

I 7919608 7919620

I 7919713 7919765

I 7935876 7935883

I 7935954 7936047

I 7960860 7960875

I 7980376 7980480

I 7980633 7980692

I 7980746 7980872

I 7981107 7981388

I 7986028 7986137

I 8004725 8004741

I 8067861 8067914

I 8068070 8068079

I 8085942 8086023

I 8086087 8086113

I 8105419 8105528

I 8119739 8119842

I 8131480 8131571

I 8131668 8132286

I 8132377 8132712

I 8132888 8133103

I 8153876 8154183

I 8154277 8154519

I 8158791 8158878

I 8162513 8162825

I 8162918 8163159

I 8167599 8167688

I 8213710 8213768

I 8213855 8213906

I 8279300 8279345

I 8279572 8279880

I 8279932 8280096

I 8309657 8309661

I 8325917 8325943

I 8371511 8371548

I 8371710 8371722

I 8372160 8372210

I 8372516 8372547

I 8372643 8373800

I 8374059 8374156

I 8375762 8375770

I 8375924 8376005

I 8376198 8376230

I 8376454 8376458

I 8376609 8376653

I 8376768 8376769

I 8377278 8377428

I 8377492 8377536

I 8377880 8377909

I 8433111 8433131

I 8433229 8433249

I 8433353 8433417

I 8433512 8433584

I 8433681 8433860

I 8434183 8434203

I 8438947 8438972

I 8439209 8439233

I 8441102 8441118

I 8441356 8441386

I 8441479 8441485

I 8464527 8464721

I 8464782 8465220

I 8465308 8465695

I 8485031 8485070

I 8493929 8493987

I 8494056 8494125

I 8494225 8494288

I 8496497 8496510

I 8496850 8496861

I 8541481 8541501

I 8541766 8541828

I 8541901 8541926

I 8542086 8542257

I 8542350 8542526

I 8542591 8542710

I 8542778 8542887

I 8542979 8543420

I 8543518 8543544

I 8543637 8543733

I 8543812 8543824

I 8546020 8546110

I 8618718 8619148

I 8619243 8619319

I 8619408 8619746

I 8619836 8619923

I 8620015 8620830

I 8620926 8622445

I 8622540 8622970

I 8628016 8628025

I 8629947 8629956

I 8631026 8631065

I 8631223 8631290

I 8631392 8631407

I 8647232 8647840

I 8647902 8648160

I 8648435 8648483

I 8648565 8648681

I 8648764 8649236

I 8649344 8649646

I 8664475 8664485

I 8681463 8681526

I 8700584 8700688

I 8757935 8757964

I 8761163 8761220

I 8774103 8774134

I 8774295 8774346

I 8778249 8778259

I 8781625 8781646

I 8783222 8783280

I 8791251 8791547

I 8791675 8791694

I 8808199 8808400

I 8844574 8844583

I 8855753 8855912

I 8856076 8856322

I 8856414 8856473

I 8863298 8863354

I 8942620 8942647

I 8949183 8949233

I 8949311 8949880

I 8949975 8950281

I 8950400 8950529

I 8950668 8950761

I 8950918 8951153

I 8951248 8951399

I 8951494 8951509

I 8951602 8951667

I 8952121 8952345

I 8952422 8952784

I 8952869 8952996

I 8953093 8953126

I 9027453 9027466

I 9028095 9028216

I 9028503 9028587

I 9028713 9028763

I 9029089 9029397

I 9029484 9029715

I 9029793 9029823

I 9029969 9030015

I 9030110 9030132

I 9030209 9030228

I 9030320 9030355

I 9030437 9030548

I 9030642 9030718

I 9030920 9030935

I 9053912 9053950

I 9054035 9054076

I 9080711 9080750

I 9080861 9080874

I 9082267 9082304

I 9082400 9082417

I 9085230 9085272

I 9089159 9089183

I 9146863 9146890

I 9146962 9147025

I 9147115 9147244

I 9147328 9147354

I 9147436 9147444

I 9147448 9147521

I 9147616 9147634

I 9147719 9147754

I 9147845 9147968

I 9148056 9148155

I 9148226 9148249

I 9155002 9155003

I 9156832 9156856

I 9169869 9169911

I 9169996 9170139

I 9205311 9205312

I 9212860 9213046

I 9257433 9257438

I 9258248 9258256

I 9264904 9264939

I 9265209 9265450

I 9300614 9300923

I 9301007 9301144

I 9301234 9301529

I 9301620 9301810

I 9307251 9307385

I 9314813 9314818

I 9315794 9315829

I 9316630 9316760

I 9316843 9316859

I 9346251 9346335

I 9346427 9346833

I 9346969 9347134

I 9347227 9347246

I 9347340 9347417

I 9347513 9347555

I 9347758 9347932

I 9348277 9348721

I 9351258 9351327

I 9383027 9383127

I 9384542 9384937

I 9385054 9385076

I 9385302 9385349

I 9386654 9386675

I 9386856 9387454

I 9407508 9407523

I 9452243 9452353

I 9452443 9452866

I 9452973 9452974

I 9453069 9453673

I 9453761 9453786

I 9490401 9490418

I 9490525 9490576

I 9490769 9490794

I 9490988 9491081

I 9491230 9491244

I 9491338 9491428

I 9491596 9491790

I 9497600 9497665

I 9497735 9497737

I 9497917 9497920

I 9498015 9498103

I 9498153 9498239

I 9498335 9498452

I 9498547 9498551

I 9498632 9498812

I 9498867 9499017

I 9499083 9499098

I 9499262 9499304

I 9499459 9499649

I 9499864 9499888

I 9499980 9500185

I 9501765 9501790

I 9505913 9506095

I 9545912 9545937

I 9559295 9559344

I 9562347 9562398

I 9563875 9563912

I 9569153 9569291

I 9569382 9569515

I 9570000 9570014

I 9570205 9570326

I 9574950 9575486

I 9576959 9577522

I 9577624 9577679

I 9577839 9577862

I 9590545 9590642

I 9591189 9591286

I 9591396 9591416

I 9591520 9591732

I 9606703 9606810

I 9606903 9607326

I 9607404 9607540

I 9607632 9607862

I 9631015 9631024

I 9636909 9636915

I 9637053 9637694

I 9643074 9643102

I 9656156 9656460

I 9656575 9656780

I 9656962 9656971

I 9672717 9672734

I 9672817 9672855

I 9679447 9679840

I 9679925 9680240

I 9680334 9682866

I 9692728 9692962

I 9693126 9693151

I 9693238 9693295

I 9693764 9693856

I 9695785 9695838

I 9696221 9696230

I 9728452 9728714

I 9728834 9728932

I 9729094 9729267

I 9729586 9729641

I 9729922 9730000

I 9730113 9730114

I 9730115 9730119

I 9776484 9776577

I 9776663 9776751

I 9776845 9777023

I 9793435 9793491

I 9806845 9806914

I 9811551 9811574

I 9811663 9811707

I 9811798 9811816

I 9811886 9811973

I 9816874 9819684

I 9819873 9819901

I 9819947 9819986

I 9820055 9820066

I 9820140 9820190

I 9820416 9821000

I 9821233 9821243

I 9821380 9821383

I 9821476 9821967

I 9822057 9822107

I 9822187 9822310

I 9822396 9822451

I 9822543 9822564

I 9822652 9822968

I 9823308 9823603

I 9823691 9823763

I 9823857 9823877

I 9824183 9824226

I 9824320 9824356

I 9925281 9925335

I 9925511 9925542

I 9983583 9983791

I 9994209 9994271

I 9998526 9998547

I 9998743 9998749

I 10006045 10006234

I 10006312 10006353

I 10006526 10006583

I 10019752 10019778

I 10020051 10020100

I 10020239 10020260

I 10021921 10021929

I 10027450 10027496

I 10027583 10027613

I 10064524 10064543

I 10071186 10071333

I 10071546 10071683

I 10073631 10073803

I 10073897 10073969

I 10074079 10074103

I 10076156 10076217

I 10076322 10076329

I 10076525 10076554

I 10076725 10076991

I 10090791 10090871

I 10102265 10102298

I 10130631 10130693

I 10130789 10130894

I 10131026 10131549

I 10131602 10132279

I 10132365 10133074

I 10137111 10137265

I 10137393 10137745

I 10140963 10141651

I 10141713 10141845

I 10141931 10142112

I 10166857 10166873

I 10181738 10181795

I 10184192 10184205

I 10188810 10188819

I 10188968 10189009

I 10189336 10189469

I 10189525 10189606

I 10189710 10189792

I 10189871 10189890

I 10201013 10201039

I 10201150 10201223

I 10201318 10201488

I 10201957 10202050

I 10202153 10202168

I 10202449 10202516

I 10203227 10203242

I 10203344 10203410

I 10203494 10203809

I 10204169 10204229

I 10204325 10205378

I 10205540 10206072

I 10206197 10207393

I 10207520 10207527

I 10207629 10207878

I 10208057 10208169

I 10208249 10209391

I 10209467 10209775

I 10209863 10209947

I 10210034 10210626

I 10210674 10214948

I 10215041 10215087

I 10215173 10216203

I 10216274 10216318

I 10216403 10217984

I 10218058 10219183

I 10219326 10219389

I 10219475 10219477

I 10219741 10219816

I 10219906 10219924

I 10219977 10219982

I 10220130 10220269

I 10220363 10220391

I 10220509 10220646

I 10220857 10221002

I 10238809 10238830

I 10239172 10239216

I 10239368 10241624

I 10263471 10263652

I 10266381 10266498

I 10266611 10266726

I 10266912 10266978

I 10267075 10267338

I 10267419 10267566

I 10267662 10268090

I 10268144 10269317

I 10269368 10269434

I 10269500 10271046

I 10271147 10271652

I 10271733 10272791

I 10272869 10272902

I 10272997 10274192

I 10274254 10274389

I 10274468 10276316

I 10276436 10276842

I 10276925 10277017

I 10287558 10287592

I 10291682 10291700

I 10302563 10303219

I 10303305 10303362

I 10303523 10303558

I 10303862 10303894

I 10303996 10304063

I 10304639 10304733

I 10304813 10304820

I 10304917 10304980

I 10305079 10305163

I 10305307 10305332

I 10305465 10305732

I 10305943 10306090

I 10306411 10306539

I 10306735 10306794

I 10306954 10307087

I 10307428 10307558

I 10307661 10308089

I 10308181 10308711

I 10308885 10308929

I 10309009 10309108

I 10309546 10309579

I 10309691 10309724

I 10311703 10311996

I 10312150 10312296

I 10312980 10312995

I 10313219 10313302

I 10313620 10313675

I 10313900 10313933

I 10314097 10314801

I 10315376 10315544

I 10315850 10315954

I 10316049 10316055

I 10316148 10316298

I 10316801 10316814

I 10316905 10317198

I 10317888 10318066

I 10318389 10318440

I 10318537 10318622

I 10318955 10319609

I 10364158 10364180

I 10415010 10415470

I 10415716 10415724

I 10416006 10416476

I 10416572 10416635

I 10416714 10416889

I 10417022 10417041

I 10417170 10417201

I 10417287 10417364

I 10417530 10417596

I 10417650 10417824

I 10417882 10417911

I 10418474 10418554

I 10418646 10418710

I 10418766 10418981

I 10419411 10419586

I 10419873 10419964

I 10420054 10420069

I 10420168 10420201

I 10420510 10420530

I 10420685 10420741

I 10421182 10421251

I 10421508 10421553

I 10421796 10421865

I 10421977 10422075

I 10454328 10456118

I 10456488 10456511

I 10457343 10458679

I 10458766 10459132

I 10459849 10459919

I 10460082 10460141

I 10460343 10460364

I 10460453 10460670

I 10460787 10460907

I 10460996 10461009

I 10461097 10461154

I 10461538 10461604

I 10461736 10461833

I 10462185 10462209

I 10462304 10462429

I 10463079 10463129

I 10463190 10463195

I 10468993 10469037

I 10469124 10469295

I 10485615 10485794

I 10487615 10487645

I 10498803 10498816

I 10500255 10500267

I 10500438 10500485

I 10517113 10517127

I 10528280 10529451

I 10529566 10529809

I 10530579 10530767

I 10549857 10549938

I 10550409 10550425

I 10550661 10550671

I 10550872 10551009

I 10551115 10551196

I 10551267 10551361

I 10561767 10561790

I 10565296 10565309

I 10594090 10594091

I 10594299 10594355

I 10596530 10596546

I 10596672 10596702

I 10601158 10601269

I 10601336 10601347

I 10601719 10601728

I 10601876 10601914

I 10602028 10602041

I 10603946 10604267

I 10604452 10604654

I 10620130 10620142

I 10622525 10622568

I 10636219 10636223

I 10651404 10651458

I 10651641 10651758

I 10651853 10652230

I 10652483 10652849

I 10652946 10653569

I 10653660 10653682

I 10678146 10678164

I 10678218 10678315

I 10697790 10697842

I 10697997 10698080

I 10717311 10717493

I 10717840 10717895

I 10718054 10718068

I 10718185 10718332

I 10718423 10718510

I 10718767 10718789

I 10718921 10719056

I 10719192 10719313

I 10719958 10720107

I 10720240 10720320

I 10740856 10740857

I 10740859 10740860

I 10740936 10740993

I 10785200 10785277

I 10786438 10786444

I 10787224 10787234

I 10787377 10787400

I 10787602 10787610

I 10788340 10788410

I 10789288 10789293

I 10789493 10789521

I 10789665 10789673

I 10791198 10791204

I 10792634 10792711

I 10795922 10795964

I 10796035 10796041

I 10803248 10803321

I 10811166 10811220

I 10811392 10811439

I 10811747 10811768

I 10812185 10812198

I 10812295 10812309

I 10821323 10821548

I 10821639 10821696

I 10829508 10829551

I 10851743 10851788

I 10851838 10852604

I 10852652 10852968

I 10853058 10854421

I 10854471 10854853

I 10854903 10855343

I 10858064 10858071

I 10858139 10858166

I 10871424 10872956

I 10900081 10900179

I 10906086 10906949

I 10909825 10909865

I 10909958 10910035

I 10910388 10910392

I 10910393 10910394

I 10910488 10910517

I 10910607 10910619

I 10910710 10910718

I 10910767 10910815

I 10910906 10910923

I 10911082 10911087

I 10911177 10911275

I 10914162 10914385

I 10914451 10914506

I 10918953 10919821

I 10929821 10929825

I 10946059 10946077

I 10946143 10946200

I 10946247 10946433

I 10946498 10946664

I 10946720 10946781

I 10946831 10947121

I 10947199 10947307

I 10947356 10947371

I 10947444 10947639

I 10947786 10947961

I 10948078 10948126

I 10948240 10948310

I 10948477 10948627

I 10948805 10948822

I 10948931 10949132

I 10949262 10950026

I 10950075 10950191

I 10950337 10950407

I 10950486 10950898

I 10950959 10950967

I 10951047 10951322

I 10951372 10951387

I 10951474 10951543

I 10951600 10951749

I 10951796 10951928

I 10951991 10951997

I 10952052 10952883

I 10952963 10952980

I 10953616 10953663

I 10953826 10953871

I 10955796 10955799

I 10955886 10955900

I 10956828 10956875

I 10956964 10957129

I 10957306 10957473

I 10957523 10957634

I 10957696 10957699

I 10957850 10957889

I 10957971 10958018

I 10958108 10958142

I 10958334 10958618

I 10958711 10958843

I 10959047 10959091

I 10963009 10963035

I 10967257 10967260

I 10967851 10967855

I 10986868 10986900

I 10987004 10987037

I 10987392 10987436

I 10987685 10987715

I 10987816 10987851

I 10987989 10988036

I 10993606 10993615

I 10995689 10995796

I 11014281 11014319

I 11014914 11014969

I 11015056 11015119

I 11020551 11020797

I 11020858 11020931

I 11021115 11021123

I 11028542 11028547

I 11032697 11032759

I 11042284 11042540

I 11042730 11042994

I 11044286 11044287

I 11044892 11044895

I 11053845 11053862

I 11054242 11054255

I 11063218 11063262

I 11063700 11063781

I 11063975 11063989

I 11064166 11064172

I 11064306 11064320

I 11064415 11064541

I 11064933 11064968

I 11065061 11065096

I 11065290 11065315

I 11065504 11065583

I 11065747 11065836

I 11067397 11067434

I 11067562 11067589

I 11068014 11068023

I 11068529 11068787

I 11075862 11075953

I 11076117 11076192

I 11079714 11079779

I 11081722 11081765

I 11082336 11082369

I 11083132 11083141

I 11083681 11083941

I 11098847 11098859

I 11099240 11099258

I 11108901 11108972

I 11109036 11109178

I 11116525 11116657

I 11116916 11116984

I 11133926 11134098

I 11142321 11142467

I 11142547 11142613

I 11142700 11142747

I 11142900 11143195

I 11144944 11144957

I 11147144 11147225

I 11157784 11158146

I 11165572 11165587

I 11165776 11165845

I 11166106 11166124

I 11166199 11166230

I 11170001 11170026

I 11170347 11170372

I 11170478 11170485

I 11170626 11170638

I 11170729 11170764

I 11170908 11170920

I 11171052 11171061

I 11171147 11171178

I 11171372 11172091

I 11172142 11172166

I 11172478 11172522

I 11176828 11176834

I 11177233 11177235

I 11190720 11190758

I 11194057 11194061

I 11194160 11194163

I 11215431 11215538

I 11228140 11228166

I 11228256 11228366

I 11228490 11228585

I 11228713 11228948

I 11229027 11229770

I 11229887 11229934

I 11230007 11230066

I 11230119 11230158

I 11230325 11230391

I 11230509 11230578

I 11230687 11231520

I 11231586 11231745

I 11233876 11234145

I 11236871 11237081

I 11237398 11237406

I 11238019 11238226

I 11238795 11238863

I 11241529 11241546

I 11241676 11241678

I 11242989 11243042

I 11243174 11243178

I 11244670 11244730

I 11246938 11246960

I 11247039 11247061

I 11247285 11247458

I 11248885 11248892

I 11249181 11249230

I 11249290 11249353

I 11254078 11254140

I 11254201 11254262

I 11263569 11263575

I 11264725 11264969

I 11265123 11265365

I 11266994 11267103

I 11267199 11267384

I 11268957 11269351

I 11269499 11269508

I 11269662 11269694

I 11273035 11273040

I 11273786 11273848

I 11273917 11273993

I 11274151 11274239

I 11274280 11274536

I 11274615 11274620

I 11281581 11281712

I 11281776 11281879

I 11281929 11282058

I 11299689 11299758

I 11299838 11300739

I 11301293 11302195

I 11302276 11305710

I 11307629 11311063

I 11312870 11313077

I 11313694 11313836

I 11313924 11313937

I 11313988 11313999

I 11314064 11314162

I 11314360 11314463

I 11314676 11314688

I 11314786 11314854

I 11316113 11316184

I 11316263 11316336

I 11317673 11318369

I 11318460 11318473

I 11318569 11319055

I 11324986 11325119

I 11325233 11325323

I 11325414 11325556

I 11325607 11325687

I 11338253 11338291

I 11338341 11338369

I 11338502 11338532

I 11338872 11338953

I 11339203 11339274

I 11342658 11342716

I 11344814 11344840

I 11345236 11345263

I 11349515 11349936

I 11350006 11350437

I 11350528 11350645

I 11355220 11356393

I 11357497 11357554

I 11358138 11359319

I 11364047 11364052

I 11364129 11364140

I 11365876 11366045

I 11366126 11366557

I 11383745 11383781

I 11384416 11384603

I 11384675 11384688

I 11385106 11385245

I 11385325 11385404

I 11385612 11385627

I 11385696 11385779

I 11386000 11386101

I 11386294 11386321

I 11398042 11398043

I 11398839 11398842

I 11398974 11398990

I 11399463 11399464

I 11399594 11399609

I 11406976 11407222

I 11407290 11407534

I 11420326 11420331

I 11420532 11420564

I 11420814 11420820

I 11420901 11420932

I 11425264 11425373

I 11426837 11427073

I 11427163 11427215

I 11427293 11427399

I 11427859 11427941

I 11431410 11431441

I 11431535 11433537

I 11433679 11433916

I 11434040 11435570

I 11435907 11436173

I 11436349 11436568

I 11436707 11437518

I 11437912 11437928

I 11438135 11438395

I 11438963 11439078

I 11439726 11439812

I 11440000 11440018

I 11440189 11440197

I 11440634 11440711

I 11440815 11440832

I 11441263 11441298

I 11441950 11442037

I 11442133 11442148

I 11442245 11442327

I 11442422 11442459

I 11442586 11442587

I 11442724 11442802

I 11443163 11443223

I 11443351 11443389

I 11443597 11443627

I 11443861 11443955

I 11444160 11444166

I 11444482 11444486

I 11444582 11444626

I 11444841 11444905

I 11445033 11445190

I 11445286 11445300

I 11445908 11445917

I 11446002 11446022

I 11446114 11446208

I 11446375 11446486

I 11446626 11446704

I 11446906 11447081

I 11448933 11452295

I 11452389 11453585

I 11453637 11453862

I 11453959 11454698

I 11454801 11454829

I 11454922 11456919

I 11457958 11458031

I 11458197 11458207

I 11458485 11458552

I 11464375 11464446

I 11466737 11467475

I 11467569 11467791

I 11467853 11469045

I 11469138 11472510

I 11473166 11473977

I 11477194 11477411

I 11477584 11477851

I 11478463 11479991

I 11480082 11480200

I 11480265 11480695

I 11480747 11480868

I 11480990 11481084

I 11481154 11481186

I 11481241 11481369

I 11481550 11481623

I 11481704 11481937

I 11482061 11482296

I 11484997 11484998

I 11494821 11494845

I 11494992 11495026

I 11495281 11495374

I 11495506 11495548

I 11495642 11495689

I 11496126 11496220

I 11496438 11496452

I 11507986 11508012

I 11508118 11508124

I 11508418 11508438

I 11508525 11508535

I 11509175 11509272

I 11563056 11563123

I 11563227 11563241

I 11563318 11563346

I 11563482 11563552

I 11563656 11563667

I 11563780 11563808

I 11585402 11585412

I 11592873 11593209

I 11593333 11593377

I 11593524 11593528

I 11595736 11595738

I 11595883 11595932

I 11596055 11596390

I 11615768 11620907

I 11620986 11621338

I 11622038 11622389

I 11622475 11627607

I 11627743 11628112

I 11628187 11628220

I 11628312 11628322

I 11628714 11629086

I 11629210 11629226

I 11629291 11629295

I 11629706 11629746

I 11637184 11638007

I 11638092 11638385

I 11647419 11647430

I 11649534 11649567

I 11649658 11649670

I 11655791 11656023

I 11656079 11656259

I 11656353 11656363

I 11656997 11657318

I 11657410 11657622

I 11659750 11659818

I 11660112 11660177

I 11669217 11669235

I 11684723 11684885

I 11686426 11686457

I 11686554 11686635

I 11686716 11686799

I 11686887 11686904

I 11687217 11687230

I 11687361 11687466

I 11687565 11687767

I 11691778 11691799

I 11692041 11692055

I 11703637 11703656

I 11703763 11703812

I 11703935 11703950

I 11704207 11704244

I 11704723 11704791

I 11705279 11705311

I 11705830 11705851

I 11706093 11706137

I 11706871 11706884

I 11707924 11707972

I 11711067 11711081

I 11712340 11712378

I 11712489 11712561

I 11712657 11712669

I 11713782 11713818

I 11714675 11714679

I 11722946 11722973

I 11723116 11723175

I 11726271 11726304

I 11726441 11726469

I 11726911 11727340

I 11727440 11727532

I 11727622 11728051

I 11728313 11728347

I 11728684 11728771

I 11728872 11728993

I 11729063 11729255

I 11729349 11729393

I 11729567 11729752

I 11729909 11730132

I 11730204 11730226

I 11730396 11730595

I 11730649 11730699

I 11730952 11730955

I 11732984 11733189

I 11733523 11733725

I 11738200 11738205

I 11738698 11738740

I 11738835 11738984

I 11739141 11739255

I 11739427 11739436

I 11739820 11739937

I 11740205 11740242

I 11740480 11740490

I 11740691 11740758

I 11740871 11740913

I 11763086 11763122

I 11763213 11763225

I 11763335 11763403

I 11763477 11763516

I 11763667 11763870

I 11824028 11824103

I 11824220 11824370

I 11829629 11829655

I 11829785 11829965

I 11830660 11830747

I 11846813 11846933

I 11849316 11851575

I 11861224 11861274

I 11861338 11861347

I 11861421 11861470

I 11861923 11861924

I 11868587 11868590

I 11868591 11868593

I 11868891 11869026

I 11880827 11880864

I 11880922 11880988

I 11881163 11881164

I 11881165 11881166

I 11881218 11881395

I 11881478 11881672

I 11881832 11881870

I 11881957 11881986

I 11882399 11882473

I 11882568 11882720

I 11882800 11882877

I 11882941 11882963

I 11883117 11883231

I 11883286 11883356

I 11883535 11883554

I 11883647 11883772

I 11883866 11883912

I 11886572 11886581

I 11886753 11886756

I 11888289 11888356

I 11888662 11888676

I 11889574 11889591

I 11889735 11889736

I 11890136 11890184

I 11897027 11897168

I 11899481 11899485

I 11899848 11899868

I 11900226 11900281

I 11900376 11900594

I 11900690 11900808

I 11900950 11901007

I 11901164 11901191

I 11901557 11901575

I 11902068 11902210

I 11928160 11928172

I 11928564 11928577

I 11935981 11936007

I 11936094 11936121

I 11941634 11941910

I 11941981 11942254

I 11942339 11942416

I 11943543 11943550

I 11943907 11943921

I 11944150 11944171

I 11944300 11944336

I 11944902 11944918

I 11960563 11960628

I 11960846 11960937

I 11965770 11965912

I 11970669 11970677

I 11970769 11970799

I 11975784 11975829

I 11989964 11989968

I 11990090 11990091

I 11990204 11990241

I 11990663 11990753

I 11997087 11997318

I 11997471 11997497

I 11997589 11997642

I 11997733 11997761

I 12012605 12012636

I 12018703 12018774

I 12018834 12018854

I 12018948 12018993

I 12020170 12020202

I 12023596 12023660

I 12023803 12023823

I 12026673 12026727

I 12026824 12026896

I 12027033 12027106

I 12027203 12027223

I 12027381 12027540

I 12027697 12027699

I 12037310 12037340

I 12037433 12037673

I 12037806 12037953

I 12038045 12038225

I 12038368 12038490

I 12038588 12038614

I 12038705 12038913

I 12067057 12067066

I 12067135 12067144

I 12067229 12067353

I 12067567 12067676

I 12067735 12067771

I 12071901 12071910

I 12071965 12071975

I 12073302 12073336

I 12075201 12075240

I 12075289 12075343

I 12075435 12075500

I 12075605 12075632

I 12075727 12075960

I 12076431 12076487

I 12076583 12076916

I 12077138 12077240

I 12077345 12077430

I 12077722 12077892

I 12077986 12078025

I 12078133 12078246

I 12078339 12078940

I 12079155 12079170

I 12079258 12079423

I 12079589 12079756

I 12079844 12079862

I 12080071 12080125

I 12106174 12106237

I 12110059 12110190

I 12113138 12113224

I 12113382 12113530

I 12113627 12113671

I 12116763 12119018

I 12124025 12124048

I 12124874 12126820

I 12129580 12129741

I 12130195 12131092

I 12131184 12131303

I 12132630 12132747

I 12132842 12133735

I 12133827 12134058

I 12134172 12134878

I 12134971 12138201

I 12138824 12142053

I 12142146 12142852

I 12144044 12144120

I 12144215 12145568

I 12146757 12146986

I 12149181 12150532

I 12150629 12150702

I 12159001 12159009

I 12159380 12159392

I 12173753 12173949

I 12190649 12190790

I 12190983 12191013

I 12191275 12191280

I 12191337 12191347

I 12191503 12191550

I 12191704 12191718

I 12191832 12191877

I 12191964 12191973

I 12192101 12192105

I 12192224 12192296

I 12192420 12192518

I 12192580 12192971

I 12193405 12193594

I 12193674 12193717

I 12243931 12243936

I 12244140 12244174

I 12250558 12250575

I 12250665 12250702

I 12258131 12258144

I 12258267 12258419

I 12258533 12258545

I 12258618 12261157

I 12261277 12262458

I 12262551 12262877

I 12263377 12263573

I 12263668 12264902

I 12265022 12265085

I 12266955 12267348

I 12267617 12267618

I 12267777 12267789

I 12267963 12270498

I 12270619 12271798

I 12271894 12272220

I 12275562 12275572

I 12275724 12275727

I 12276534 12276612

I 12278440 12278837

I 12279047 12279112

I 12279226 12280466

I 12280563 12280753

I 12284128 12284148

I 12284339 12284340

I 12285750 12285906

I 12286098 12286104

I 12286604 12286746

I 12288262 12288443

I 12289597 12289650

I 12307695 12307758

I 12307870 12307894

I 12309025 12309052

I 12311172 12311214

I 12311307 12311319

I 12311378 12311459

I 12311508 12311597

I 12311666 12311752

I 12312076 12312093

I 12312300 12312365

I 12312475 12312501

I 12313500 12313519

I 12324783 12324902

I 12343643 12343680

I 12354501 12354515

I 12361294 12361312

I 12365354 12365366

I 12369249 12369294

I 12370294 12370304

I 12371412 12371457

I 12372344 12372348

I 12399720 12399726

I 12400054 12400079

I 12401519 12402681

I 12406318 12406326

I 12414979 12415005

I 12415347 12415350

I 12424620 12424624

I 12424736 12424834

I 12424922 12424963

I 12452072 12452083

I 12452211 12452351

I 12463115 12463168

I 12463274 12463283

I 12465601 12465768

I 12465920 12466041

I 12466132 12467154

I 12467224 12467311

I 12467375 12467414

I 12467628 12468779

I 12468857 12468938

I 12469134 12469287

I 12469344 12470394

I 12470513 12470638

I 12470729 12470799

I 12473371 12473434

I 12474016 12474180

I 12474345 12474474

I 12474573 12474640

I 12479602 12479641

I 12479747 12479759

I 12480989 12481209

I 12481366 12482124

I 12482206 12482622

I 12482710 12482860

I 12583614 12583733

I 12584278 12584399

I 12597750 12597777

I 12598082 12598213

I 12598908 12598959

I 12599073 12599074

I 12599077 12599078

I 12599656 12599660

I 12599762 12599875

I 12600721 12600836

I 12600938 12600949

I 12601078 12601156

I 12601247 12601264

I 12601512 12601527

I 12601642 12601658

I 12614020 12614038

I 12627391 12627812

I 12627906 12633504

I 12634223 12639820

I 12639915 12640335

I 12646723 12646734

I 12657336 12657384

I 12677121 12677132

I 12691061 12691194

I 12691359 12691420

I 12691512 12691575

I 12698634 12698652

I 12707096 12707124

I 12750833 12750844

I 12751215 12751223

I 12752106 12752198

I 12752379 12752611

I 12753251 12753310

I 12753365 12753519

I 12753611 12753830

I 12753886 12753932

I 12754017 12754025

I 12754190 12754288

I 12754404 12754445

I 12754539 12754634

I 12755097 12755249

I 12755358 12755363

I 12756077 12756207

I 12756526 12756527

I 12756616 12756804

I 12756869 12756896

I 12757016 12757075

I 12757167 12757317

I 12757411 12757434

I 12757984 12758018

I 12776129 12776205

I 12777610 12778107

I 12778204 12778304

I 12779530 12779681

I 12782387 12782467

I 12782519 12782592

I 12782654 12782723

I 12782810 12782944

I 12782995 12783119

I 12785244 12785259

I 12785417 12785481

I 12785647 12785657

I 12785959 12785979

I 12786075 12786109

I 12786481 12786517

I 12786607 12786640

I 12786760 12786794

I 12786984 12787059

I 12798739 12798829

I 12799617 12799706

I 12819578 12819584

I 12819682 12819688

I 12819775 12819800

I 12820011 12820060

I 12827699 12827739

I 12840139 12840177

I 12840311 12840380

I 12840473 12840716

I 12840812 12840834

I 12840925 12840996

I 12841175 12841320

I 12841414 12841506

I 12841639 12841709

I 12852227 12852329

I 12852386 12852432

I 12854654 12854663

I 12854907 12855079

I 12855134 12855347

I 12858609 12858809

I 12886962 12887000

I 12887092 12887141

I 12893116 12893268

I 12899264 12899319

I 12899458 12899522

I 12899638 12899786

I 12910085 12910142

I 12910515 12910547

I 12910621 12910682

I 12911130 12911146

I 12911238 12911241

I 12911242 12911246

I 12911433 12911448

I 12911550 12911563

I 12911655 12911692

I 12912017 12912090

I 12912181 12912218

I 12912523 12912603

I 12912943 12913086

I 12913176 12913189

I 12914470 12914484

I 12914578 12914720

I 12915063 12915139

I 12924702 12925680

I 12925888 12926864

I 12927260 12927275

I 12927510 12927525

I 12927622 12927773

I 12935538 12935736

I 12935986 12936060

I 12944670 12944683

I 12946163 12946189

I 12948778 12948900

I 12949214 12949229

I 12950589 12950916

I 12952425 12952548

I 12952862 12952876

I 12953523 12953856

I 12956051 12956109

I 12985331 12985428

I 12985529 12985530

I 13016340 13017094

I 13017143 13018830

I 13018904 13018966

I 13021238 13021252

I 13076840 13077676

I 13077762 13078599

I 13082525 13082549

I 13085138 13085694

I 13085753 13085877

I 13086243 13086300

I 13086399 13086450

I 13086637 13086818

I 13087506 13087795

I 13087890 13087901

I 13088454 13088599

I 13088693 13088705

I 13088798 13088902

I 13089208 13089374

I 13089948 13090652

I 13090819 13090853

I 13091076 13091134

I 13091454 13091531

I 13091759 13091773

I 13093237 13093240

I 13093455 13093470

I 13094614 13094640

I 13095272 13095308

I 13095478 13095614

I 13095709 13096011

I 13096087 13096226

I 13096398 13096609

I 13096780 13096783

I 13096978 13097046

I 13097112 13097134

I 13097398 13097520

I 13097682 13097787

I 13097880 13097927

I 13098011 13098066

I 13098121 13098142

I 13098387 13098529

I 13098744 13098979

I 13099153 13099170

I 13099344 13099410

I 13099489 13099642

I 13100925 13100936

I 13101992 13102098

I 13102223 13102755

I 13111594 13111606

I 13118306 13118309

I 13118577 13118587

I 13119459 13119472

I 13124414 13124441

I 13124823 13124834

I 13128710 13128741

I 13129117 13129128

I 13133139 13133213

I 13146843 13146909

I 13146982 13147111

I 13147207 13147242

I 13147389 13147428

I 13148101 13148102

I 13148587 13148593

I 13149023 13149061

I 13149401 13149430

I 13149579 13151647

I 13151794 13151803

I 13151929 13151962

I 13154962 13154986

I 13162737 13162922

I 13163009 13163088

I 13163187 13163225

I 13163326 13163345

I 13163540 13163575

I 13163901 13163954

I 13170528 13170731

I 13175887 13175985

I 13176074 13176303

I 13176406 13176491

I 13176639 13176864

I 13188172 13188228

I 13188372 13188669

I 13188855 13189120

I 13189325 13189326

I 13198632 13198672

I 13212351 13212613

I 13212775 13213148

I 13213239 13213424

I 13219896 13219919

I 13220010 13220017

I 13220237 13220280

I 13220350 13220495

I 13220566 13220791

I 13221131 13221252

I 13221427 13221439

I 13221618 13221685

I 13222060 13222130

I 13222312 13222627

I 13222854 13223041

I 13223135 13223170

I 13223494 13223602

I 13223697 13223704

I 13223806 13223950

I 13224096 13224160

I 13224254 13224369

I 13224466 13224592

I 13224838 13224990

I 13225090 13225106

I 13225200 13225269

I 13225338 13225344

I 13225521 13225548

I 13225725 13225754

I 13225803 13226060

I 13226145 13226274

I 13226364 13226399

I 13226470 13226523

I 13226626 13226731

I 13226787 13226837

I 13230475 13230584

I 13230674 13230738

I 13230793 13230858

I 13230974 13231132

I 13231187 13231231

I 13231306 13231387

I 13231450 13231552

I 13231621 13231652

I 13244878 13244920

I 13245050 13245434

I 13245573 13245825

I 13251127 13251225

I 13253216 13253222

I 13253278 13253279

I 13253395 13253444

I 13255377 13255470

I 13256518 13256523

I 13266230 13266299

I 13266522 13266617

I 13266826 13266944

I 13267146 13267194

I 13267348 13267362

I 13267420 13267710

I 13267966 13268671

I 13268760 13268781

I 13268964 13269482

I 13273346 13273347

I 13273455 13273757

I 13276992 13277118

I 13277207 13277267

I 13277446 13277473

I 13309418 13309419

I 13323295 13323307

I 13360279 13360402

I 13363035 13363109

I 13363202 13363205

I 13383145 13383169

I 13404373 13404386

I 13404442 13404622

I 13404686 13408440

I 13409015 13409105

I 13411620 13411702

I 13413536 13413541

I 13413784 13413840

I 13414421 13414991

I 13416156 13416737

I 13423561 13423622

I 13423673 13423926

I 13425348 13425354

I 13425426 13425439

I 13425489 13425616

I 13426926 13427132

I 13427333 13427542

I 13429432 13429442

I 13429962 13429974

I 13432922 13432924

I 13440250 13440262

I 13440475 13440488

I 13447372 13447580

I 13447820 13447895

I 13448191 13448423

I 13450712 13450733

I 13451937 13451973

I 13453094 13453195

I 13453378 13453470

I 13453626 13453694

I 13453783 13453937

I 13454028 13454047

I 13454138 13454458

I 13454585 13454635

I 13455923 13455928

I 13456016 13458061

I 13458146 13458269

I 13466990 13467019

I 13467098 13467104

I 13467196 13467274

I 13482610 13482695

I 13482782 13482869

I 13484920 13484924

I 13485395 13485426

I 13486159 13486218

I 13486839 13486899

I 13487686 13487755

I 13487832 13488117

I 13488206 13488532

I 13488625 13488720

I 13488777 13488937

I 13497088 13497100

I 13497340 13497440

I 13498035 13498065

I 13515810 13515820

I 13516157 13516177

I 13519429 13519444

I 13520691 13520839

I 13522494 13522504

I 13522627 13522744

I 13522852 13522945

I 13523050 13523106

I 13529707 13529734

I 13530075 13530091

I 13530215 13530250

I 13532456 13532933

I 13533028 13533104

I 13533196 13533865

I 13538740 13538741

I 13538742 13538745

I 13539047 13539048

I 13545670 13545952

I 13552189 13552231

I 13552302 13552543

I 13552651 13552710

I 13552814 13552896

I 13554742 13554767

I 13554880 13554906

I 13555042 13555077

I 13558799 13558806

I 13558939 13558969

I 13562427 13562459

I 13562559 13562560

I 13562561 13562566

I 13583386 13583413

I 13583508 13583573

I 13584145 13584174

I 13584287 13584296

I 13590316 13591867

I 13594758 13594898

I 13596097 13596128

I 13596823 13596846

I 13597344 13597418

I 13597733 13597807

I 13599817 13599854

I 13600798 13600807

I 13600913 13601111

I 13601225 13601250

I 13601331 13601335

I 13601517 13601532

I 13609694 13609711

I 13609795 13609960

I 13610065 13610181

I 13610244 13610561

I 13610665 13610855

I 13610928 13611031

I 13611127 13611864

I 13611958 13612197

I 13612273 13612591

I 13612641 13612738

I 13612809 13613525

I 13613623 13613654

I 13613711 13613797

I 13613874 13615025

I 13615070 13615181

I 13615231 13615309

I 13615405 13615450

I 13615524 13615574

I 13615723 13615727

I 13624133 13624134

I 13624232 13624240

I 13624339 13624373

I 13624557 13624613

I 13630307 13630609

I 13638436 13638544

I 13659714 13659752

I 13660468 13660604

I 13660777 13660787

I 13660880 13661043

I 13661127 13661135

I 13661302 13661316

I 13662948 13662949

I 13667966 13668027

I 13675503 13675514

I 13678165 13678233

I 13678353 13678706

I 13682230 13682231

I 13689910 13689929

I 13693812 13693841

I 13693903 13693976

I 13694057 13694071

I 13694167 13694177

I 13694292 13694300

I 13694452 13694566

I 13695018 13695309

I 13708661 13708682

I 13708763 13708788

I 13710880 13710883

I 13711379 13711481

I 13711656 13711657

I 13714099 13714235

I 13714348 13714432

I 13714526 13714575

I 13715532 13715547

I 13715642 13716128

I 13716223 13717005

I 13726655 13726695

I 13728178 13728208

I 13728724 13728762

I 13728852 13728887

I 13729226 13729352

I 13729452 13729469

I 13729768 13729848

I 13729971 13730036

I 13730172 13730238

I 13730327 13730391

I 13730765 13730839

I 13737623 13737662

I 13737848 13737872

I 13737941 13737997

I 13738106 13738123

I 13738216 13738250

I 13738334 13738342

I 13738770 13738802

I 13739847 13739958

I 13740101 13742325

I 13748242 13748297

I 13748648 13748773

I 13749049 13749067

I 13749167 13749220

I 13749365 13749366

I 13753134 13753163

I 13787928 13788017

I 13794421 13794532

I 13795323 13795398

I 13796174 13796401

I 13796996 13797059

I 13798635 13798666

I 13798921 13798945

I 13799069 13799103

I 13799256 13799648

I 13799781 13799926

I 13800661 13800752

I 13800970 13801037

I 13801202 13801204

I 13801292 13801440

I 13801813 13801854

I 13825489 13825685

I 13825931 13825963

I 13826051 13826103

I 13826192 13826208

I 13826666 13826681

I 13826774 13826809

I 13829982 13829998

I 13830175 13830189

I 13830246 13830260

I 13832460 13832523

I 13833107 13833171

I 13838875 13838938

I 13839915 13839934

I 13841325 13841341

I 13841894 13841897

I 13843157 13843175

I 13843247 13843300

I 13854297 13854417

I 13858472 13859141

I 13867443 13867455

I 13868265 13868268

I 13868323 13868334

I 13873351 13873386

I 13873440 13873477

I 13873559 13873575

I 13873846 13873852

I 13873861 13874034

I 13874094 13874178

I 13875218 13875222

I 13875308 13875388

I 13876358 13876441

I 13876556 13876648

I 13877660 13877676

I 13877849 13877861

I 13878014 13878094

I 13878189 13878255

I 13878373 13878415

I 13883940 13884981

I 13885039 13885079

I 13888670 13888827

I 13888981 13888995

I 13889084 13889106

I 13889201 13889396

I 13889488 13889532

I 13889665 13889891

I 13889991 13890112

I 13890253 13892225

I 13892363 13894341

I 13894620 13894624

I 13897453 13897505

I 13903421 13903498

I 13913159 13913183

I 13914699 13914941

I 13915096 13915272

I 13916070 13916084

I 13920237 13920246

I 13920906 13920920

I 13924450 13924616

I 13924711 13924809

I 13924927 13924942

I 13925059 13925089

I 13925258 13925347

I 13925442 13925560

I 13926022 13926068

I 13929643 13929671

I 13929762 13929785

I 13930132 13930265

I 13930355 13930387

I 13930479 13930945

I 13931034 13931070

I 13931288 13931823

I 13931921 13931963

I 13932055 13932077

I 13932245 13932307

I 13932440 13932453

I 13932611 13932668

I 13932717 13932914

I 13933003 13933056

I 13933147 13933224

I 13933317 13933469

I 13933566 13933706

I 13933790 13933950

I 13934070 13934349

I 13934451 13934561

I 13934759 13934769

I 13934823 13934921

I 13935004 13935060

I 13935161 13935187

I 13935352 13936004

I 13936059 13936464

I 13937619 13937678

I 13961065 13961087

I 13961926 13961987

I 13962987 13963081

I 13963209 13963324

I 13963511 13963519

I 13965588 13965601

I 13967308 13967475

I 13967634 13967743

I 13968443 13968469

I 13968662 13969057

I 13969506 13969519

I 13969574 13969619

I 13969837 13969859

I 13969915 13969965

I 13970061 13970164

I 13970770 13970819

I 13970937 13970941

I 13971164 13971225

I 13972392 13972400

I 13972509 13972589

I 13972710 13972928

I 13973363 13973383

I 13973473 13973632

I 13973724 13973814

I 13973921 13974391

I 13974449 13974471

I 13974631 13974740

I 13977485 13977496

I 13977626 13977714

I 13977806 13977841

I 13977931 13978047

I 13988790 13988961

I 13989017 13989046

I 13991977 13991979

I 13996535 13996548

I 13996782 13996801

I 14003212 14003314

I 14003408 14003576

I 14003663 14003908

I 14004002 14004069

I 14014408 14014475

I 14021807 14021822

I 14021911 14023054

I 14023146 14023444

I 14024411 14024834

I 14040041 14040060

I 14083519 14083533

I 14087068 14087083

I 14087171 14087194

I 14087288 14087318

I 14087453 14087562

I 14090113 14090120

I 14092435 14092625

I 14092796 14092816

I 14092943 14092960

I 14093048 14093065

I 14093326 14093330

I 14093438 14093679

I 14093760 14093958

I 14094196 14094267

I 14094428 14094454

I 14094511 14094569

I 14094787 14094990

I 14095057 14095111

I 14095160 14095173

I 14095279 14095359

I 14095465 14095485

I 14095545 14096000

I 14102682 14102795

I 14105517 14105659

I 14105741 14105956

I 14106052 14106097

I 14116243 14116368

I 14116436 14116561

I 14119455 14119491

I 14119732 14119762

I 14123663 14123693

I 14123791 14123835

I 14123936 14123970

I 14124036 14124086

I 14124195 14124235

I 14124317 14124348

I 14124429 14124471

I 14127529 14127706

I 14127796 14127934

I 14128208 14128445

I 14150766 14150816

I 14152076 14152188

I 14153327 14153721

I 14154026 14154052

I 14155515 14155539

I 14155841 14156236

I 14157861 14157975

I 14192350 14192383

I 14192470 14192824

I 14192915 14192975

I 14193087 14193301

I 14201751 14201754

I 14201991 14202022

I 14202113 14202252

I 14202323 14202334

I 14210958 14210975

I 14247731 14247735

I 14247826 14247900

I 14248248 14248280

I 14248368 14248603

I 14248760 14248833

I 14248929 14248964

I 14249019 14249204

I 14249300 14249320

I 14249496 14249744

I 14249933 14249945

I 14250173 14250254

I 14250350 14250361

I 14257542 14257680

I 14257789 14257885

I 14257950 14257989

I 14258170 14258302

I 14258378 14258478

I 14263937 14263944

I 14264036 14264047

I 14264407 14264480

I 14264550 14264577

I 14285505 14285719

I 14290006 14290134

I 14290349 14290521

I 14290613 14290654

I 14290746 14291265

I 14291384 14291506

I 14292024 14292205

I 14292314 14292380

I 14292548 14292549

I 14292639 14292997

I 14293261 14293361

I 14293679 14293929

I 14294025 14294034

I 14294114 14294142

I 14294482 14294543

I 14294662 14294714

I 14294810 14294811

I 14295073 14295107

I 14295321 14295422

I 14299405 14302777

I 14302871 14306240

I 14307383 14307559

I 14309019 14309186

I 14310668 14310695

I 14311762 14311814

I 14313386 14313416

I 14319594 14319599

I 14333969 14334052

I 14338396 14338483

I 14338595 14338677

I 14341292 14341414

I 14342639 14342641

I 14342893 14342896

I 14345873 14345874

I 14345875 14345876

I 14346391 14346401

I 14348335 14348398

I 14348623 14348632

I 14348916 14348917

I 14350818 14350848

I 14350951 14351324

I 14351868 14352008

I 14352164 14352175

I 14352406 14352547

I 14352590 14352603

I 14354221 14354283

I 14354397 14354539

I 14354602 14354812

I 14354902 14354937

I 14355715 14355749

I 14357211 14357247

I 14363614 14363644

I 14363979 14364019

I 14375238 14375351

I 14375416 14375526

I 14380366 14380387

I 14383533 14383574

I 14384012 14384056

I 14384242 14384292

I 14386178 14386651

I 14387681 14387709

I 14387790 14387978

I 14388168 14388227

I 14402921 14402927

I 14403442 14403450

I 14403608 14403658

I 14414674 14414678

I 14414734 14414765

I 14420027 14420038

I 14423812 14423839

I 14427788 14427814

I 14428292 14428342

I 14434241 14434292

I 14434400 14434584

I 14436211 14436844

I 14437410 14438038

I 14443096 14443253

I 14443312 14443473

I 14443528 14443557

I 14449301 14449414

I 14452435 14452761

I 14452824 14452983

I 14453058 14453693

I 14453803 14454612

I 14455326 14455343

I 14455389 14455401

I 14455490 14455567

I 14455664 14455911

I 14456055 14456289

I 14456345 14456390

I 14456444 14456571

I 14456674 14456692

I 14456781 14456880

I 14471162 14471165

I 14471340 14471347

I 14475951 14476258

I 14481568 14481673

I 14481722 14481810

I 14481905 14481918

I 14493548 14493551

I 14493648 14493700

I 14495661 14495664

I 14495760 14495813

I 14501051 14501245

I 14501371 14501564

I 14505179 14505214

I 14505613 14505649

I 14513163 14513177

I 14513556 14513576

I 14513766 14513780

I 14517819 14517887

I 14518088 14518147

I 14518883 14519070

I 14521278 14521290

I 14527330 14527334

I 14527565 14527580

I 14534097 14534108

I 14541132 14541153

I 14542633 14542670

I 14542933 14542992

I 14545314 14545800

I 14547909 14547934

I 14548027 14548132

I 14548462 14548473

I 14551034 14551053

I 14554252 14554328

I 14554419 14554449

I 14556868 14556950

I 14561711 14561796

I 14562007 14562056

I 14562229 14562259

I 14562938 14562976

I 14563025 14563221

I 14567613 14567846

I 14567941 14568045

I 14568225 14568267

I 14574123 14574190

I 14574280 14574300

I 14574510 14574603

I 14593662 14593975

I 14594077 14594085

I 14594683 14594687

I 14595659 14595696

I 14595788 14595810

I 14595921 14596056

I 14596464 14596626

I 14596934 14597129

I 14598079 14598127

I 14598499 14598504

I 14598622 14598657

I 14598762 14598817

I 14598910 14599040

I 14600399 14600465

I 14603256 14603276

I 14603836 14603856

I 14604718 14604727

I 14604870 14604900

I 14604994 14605252

I 14605394 14605429

I 14607876 14607917

I 14609652 14609944

I 14610478 14610497

I 14617714 14617722

I 14617963 14618000

I 14618077 14618092

I 14618152 14618212

I 14618298 14618385

I 14627032 14627048

I 14635946 14636035

I 14639654 14639672

I 14640627 14640649

I 14643405 14643603

I 14643697 14643878

I 14654736 14654983

I 14655086 14655188

I 14655261 14655321

I 14659962 14660061

I 14660132 14660310

I 14660470 14660670

I 14662049 14662078

I 14662130 14662146

I 14667349 14667356

I 14667443 14667524

I 14667662 14667805

I 14668095 14668114

I 14668209 14668248

I 14668413 14668467

I 14668612 14668626

I 14668851 14668917

I 14673224 14673292

I 14673443 14673504

I 14678275 14678338

I 14678445 14678487

I 14678638 14678675

I 14678766 14678809

I 14678977 14679029

I 14679114 14679138

I 14679227 14679323

I 14679580 14679997

I 14685205 14685362

I 14685452 14685673

I 14685921 14685963

I 14686263 14686380

I 14686497 14686541

I 14686602 14686662

I 14686761 14686782

I 14687057 14687181

I 14687246 14687297

I 14702281 14702388

I 14702550 14702659

I 14715995 14716009

I 14716101 14716118

I 14723821 14723899

I 14734082 14734184

I 14734261 14734288

I 14748276 14748288

I 14750296 14750299

I 14750721 14750799

I 14750958 14751182

I 14762375 14762394

I 14764615 14764650

I 14773008 14773248

I 14773324 14773496

I 14799013 14799020

I 14799230 14799435

I 14799576 14799646

I 14799736 14799859

I 14799943 14800021

I 14800192 14800227

I 14800277 14800279

I 14800340 14800393

I 14800448 14800608

I 14800673 14800702

I 14806376 14806427

I 14823237 14823259

I 14842072 14842158

I 14842344 14842388

I 14846471 14846541

I 14885087 14889946

I 14890011 14891052

I 14891197 14891478

I 14968755 14968848

I 14969841 14969852

I 14981095 14981108

I 14981236 14981243

I 14981423 14981442

I 14981497 14981577

I 14981671 14981683

I 14981808 14981913

I 14982075 14982122

I 14982216 14982238

I 14982382 14982474

I 14982648 14982654

I 14982766 14982778

I 14982925 14982948

I 14983073 14983106

I 14983396 14983462

I 14983551 14983935

I 14988102 14988125

I 14988235 14988330

I 14988381 14988528

I 14988679 14988780

I 14989040 14989044

I 15015593 15015637

I 15042014 15042170

I 15054265 15054274

I 15054367 15054368

I 15059808 15059841

I 15059996 15060030

I 15060155 15060208

I 15060343 15060765

I 15060861 15061172

I 15061255 15064116

I 15067543 15067963

I 15068057 15068368

I 15068448 15071313

I 15071729 15071751

I 15071841 15071916

I 15072004 15072224

I 15072309 15072434

II 0 128

II 16059 18321

II 37803 37894

II 40347 40450

II 40887 40891

II 99084 99109

II 99184 99515

II 99600 99612

II 99713 99781

II 140501 140662

II 140752 142087

II 142193 142278

II 142334 142406

II 142493 142556

II 142977 143051

II 143169 143185

II 143469 143488

II 143595 143796

II 144261 144288

II 144379 144515

II 144585 145812

II 145898 146240

II 146325 146351

II 171214 171294

II 212204 212254

II 212388 212397

II 212687 212708

II 212873 212909

II 225659 225673

II 225833 225878

II 225970 226128

II 226302 226385

II 228228 228237

II 228308 228346

II 228426 228937

II 233820 233937

II 234031 234247

II 234344 234352

II 234504 234621

II 234715 234939

II 235030 235041

II 238502 238508

II 238624 238885

II 238940 239150

II 239204 239243

II 244264 244290

II 244356 244852

II 255765 256036

II 270752 270770

II 274920 274982

II 275096 275153

II 275345 275462

II 275555 275613

II 275689 275697

II 279970 279993

II 291455 291466

II 300371 300401

II 300494 300502

II 308686 308728

II 308814 308857

II 308991 309006

II 309287 309375

II 309670 309781

II 310114 310156

II 310401 310409

II 310827 310999

II 311070 311074

II 312233 312405

II 315024 315066

II 315153 315187

II 315325 315343

II 315623 317350

II 317446 319112

II 406865 406892

II 407148 407185

II 417040 417058

II 431044 431105

II 431336 432118

II 432205 432311

II 432406 432762

II 433627 433980

II 434078 434184

II 434270 434788

II 434887 435051

II 435569 435576

II 435689 435733

II 436465 436484

II 436645 436662

II 437414 437430

II 438302 438512

II 439559 439912

II 441413 441670

II 443782 443824

II 443937 443942

II 445191 445200

II 448966 448981

II 451982 451998

II 452299 452411

II 452814 452824

II 452920 452947

II 453014 453550

II 454738 455255

II 455342 455373

II 455467 455481

II 464650 464697

II 468945 469030

II 469648 469729

II 478331 480593

II 500977 501193

II 501270 502048

II 502131 502149

II 502241 502532

II 504001 504593

II 533644 533657

II 535110 535133

II 535360 535373

II 536650 536719

II 536795 536800

II 539297 539456

II 539533 539749

II 539896 539901

II 539993 540015

II 540082 540137

II 540210 540244

II 541220 541243

II 541806 541840

II 583238 583323

II 586404 586444

II 586600 586671

II 586730 586775

II 586855 586881

II 587023 587059

II 587146 587262

II 588915 589028

II 591813 591977

II 592073 592081

II 593152 593162

II 593255 593422

II 593666 593699

II 593868 593910

II 594002 594178

II 610773 610895

II 611097 611162

II 612482 612606

II 612837 612900

II 629052 629149

II 629203 629215

II 629271 629390

II 629485 629708

II 632514 632538

II 632594 632607

II 632698 632816

II 632939 632973

II 633091 633121

II 633211 633304

II 633360 633396

II 633493 633510

II 633600 633802

II 633887 634040

II 635024 635050

II 635455 635471

II 635745 635760

II 636483 636495

II 640252 640793

II 653809 653843

II 654205 654220

II 654443 654459

II 655378 655383

II 655766 655778

II 655990 656007

II 656099 656132

II 657004 657010

II 657071 657077

II 657184 657309

II 657489 657496

II 657821 657851

II 657994 658089

II 658272 658302

II 664197 664220

II 665994 666019

II 673251 673276

II 677786 677799

II 680019 680033

II 682788 682796

II 683132 683152

II 690166 690179

II 690648 690656

II 690748 690783

II 690899 690916

II 691183 691583

II 693552 693564

II 693713 693921

II 694005 694165

II 694277 694493

II 694624 694646

II 694883 694979

II 695116 695214

II 695388 695439

II 695521 695600

II 695727 696034

II 696213 696432

II 696683 696686

II 696879 697090

II 697158 697177

II 697233 697435

II 697752 697776

II 697872 698042

II 698126 698146

II 698204 698235

II 698325 698341

II 698527 698547

II 698691 698979

II 699286 699315

II 699552 699600

II 699973 699994

II 705089 705345

II 707616 707633

II 708113 708128

II 710761 711079

II 718045 718106

II 720119 720176

II 720452 720512

II 724756 724772

II 725074 725090

II 725228 725291

II 727925 727970

II 728057 728084

II 728370 728375

II 728690 728693

II 728977 728988

II 733049 733145

II 734919 734993

II 777282 777447

II 777521 777692

II 777771 777838

II 777998 778015

II 778062 778064

II 778169 778218

II 778301 778422

II 778657 779036

II 784423 785512

II 785600 785957

II 790471 790474

II 790717 790722

II 802543 802562

II 812073 812107

II 812422 812495

II 814509 814629

II 815705 815836

II 815927 815944

II 816058 816229

II 816598 816629

II 816780 816841

II 816944 817061

II 822507 822628

II 822977 823009

II 850825 850950

II 851046 851115

II 851203 851337

II 851434 851522

II 851641 851654

II 851750 852800

II 852890 853254

II 853348 853878

II 853972 854124

II 854217 854316

II 854412 855385

II 855478 855932

II 856019 856121

II 856270 856674

II 856765 856774

II 856861 856886

II 856963 856989

II 857128 857205

II 857288 857509

II 857655 857850

II 863632 863866

II 864031 864081

II 864178 864644

II 864763 865572

II 865676 865718

II 865816 866007

II 866055 866102

II 872042 872127

II 872215 872297

II 872387 872519

II 872630 872899

II 879746 879831

II 883039 883113

II 883231 883303

II 884838 885366

II 891404 891988

II 893342 893357

II 894250 895780

II 909087 909104

II 909881 909899

II 924973 924993

II 930521 930559

II 930714 930733

II 931157 931191

II 949638 949725

II 949864 949907

II 953043 953076

II 954695 954699

II 956362 956366

II 958339 958351

II 967042 967255

II 967355 967428

II 967602 967894

II 967988 968085

II 968178 968367

II 968461 968504

II 968676 968736

II 973531 973576

II 977015 977058

II 977172 977218

II 977404 977643

II 977745 977955

II 978076 978079

II 978144 978183

II 978263 978316

II 978367 978516

II 984022 984117

II 984210 984899

II 984996 986077

II 986162 986560

II 986974 987239

II 987394 987396

II 989268 989275

II 989421 989449

II 989832 989833

II 993281 993476

II 993570 993886

II 993981 995275

II 995458 996764

II 996854 997135

II 997228 997582

II 997674 998395

II 998549 998842

II 998931 999248

II 999344 999535

II 1000258 1000269

II 1003020 1015853

II 1015938 1021291

II 1021367 1021398

II 1022496 1022507

II 1022870 1022964

II 1024951 1025105

II 1027266 1027385

II 1031727 1032890

II 1033806 1033863

II 1033934 1033986

II 1034103 1034121

II 1034556 1034693

II 1034784 1035195

II 1035255 1035519

II 1035607 1037248

II 1037856 1037893

II 1037941 1040086

II 1044236 1044303

II 1045087 1045233

II 1048703 1048848

II 1049234 1049275

II 1050777 1050820

II 1051006 1051213

II 1051374 1051449

II 1051622 1051914

II 1052006 1052104

II 1052199 1052388

II 1052485 1052521

II 1052695 1052754

II 1052849 1053661

II 1053760 1054446

II 1054542 1054640

II 1054737 1055082

II 1055166 1055565

II 1055661 1055663

II 1055977 1056237

II 1056348 1056356

II 1058166 1058179

II 1058666 1058758

II 1060922 1061074

II 1062750 1062868

II 1064325 1064469

II 1064662 1064669

II 1067862 1067888

II 1072741 1072878

II 1073037 1073057

II 1073351 1073378

II 1073608 1073661

II 1076071 1077116

II 1077212 1077297

II 1077392 1079267

II 1079346 1079729

II 1079823 1081100

II 1081198 1081335

II 1081419 1081598

II 1095244 1095363

II 1095453 1095574

II 1095718 1095838

II 1095924 1096036

II 1108888 1108929

II 1109482 1109489

II 1110898 1110947

II 1112281 1112323

II 1112763 1112765

II 1117939 1117990

II 1119596 1120027

II 1120306 1120419

II 1121246 1121275

II 1134547 1134590

II 1134665 1134675

II 1134802 1134854

II 1137268 1137274

II 1137580 1137589

II 1137871 1137876

II 1138176 1138190

II 1154057 1154161

II 1154211 1154256

II 1166733 1166740

II 1168556 1168715

II 1168821 1168862

II 1170142 1170181

II 1170290 1170445

II 1171822 1171835

II 1197703 1197719

II 1198368 1198715

II 1209180 1209384

II 1209651 1209857

II 1221970 1222212

II 1222305 1222975

II 1242027 1242028

II 1250650 1250814

II 1250864 1250994

II 1251054 1251263

II 1253155 1253169

II 1253253 1253376

II 1253445 1253473

II 1260912 1260964

II 1261056 1261160

II 1261249 1261261

II 1261358 1261439

II 1261531 1261610

II 1261699 1261803

II 1274978 1275041

II 1278419 1278468

II 1281568 1281680

II 1286945 1287002

II 1287106 1287264

II 1290661 1290669

II 1290837 1290846

II 1291073 1291148

II 1291761 1291827

II 1292048 1292058

II 1292200 1292237

II 1303263 1303273

II 1303324 1303468

II 1303563 1303917

II 1304025 1304102

II 1304231 1304302

II 1308188 1308217

II 1308316 1308337

II 1314762 1314812

II 1315292 1315311

II 1319373 1319431

II 1319519 1319578

II 1342484 1342501

II 1343109 1343131

II 1362803 1362904

II 1363000 1363017

II 1363149 1363301

II 1363351 1364537

II 1364628 1364638

II 1364731 1364892

II 1364985 1365266

II 1365388 1365435

II 1365517 1365548

II 1365699 1365717

II 1367381 1367397

II 1367547 1367566

II 1367662 1367765

II 1370162 1370289

II 1378680 1378738

II 1379214 1379426

II 1379516 1379609

II 1379744 1379756

II 1379808 1379855

II 1380290 1380372

II 1397160 1397175

II 1397347 1397361

II 1411795 1411796

II 1412025 1412138

II 1414713 1415028

II 1418940 1418959

II 1419009 1419278

II 1419478 1419629

II 1423017 1423142

II 1423199 1423416

II 1424103 1424878

II 1424969 1425056

II 1427962 1427971

II 1428239 1428308

II 1430652 1430843

II 1431999 1432314

II 1435811 1436091

II 1447116 1450553

II 1450633 1450821

II 1450900 1451065

II 1451134 1451143

II 1451201 1451221

II 1451315 1451361

II 1451443 1451521

II 1451587 1451638

II 1451700 1451740

II 1451788 1452275

II 1452338 1452372

II 1452524 1452914

II 1452970 1453375

II 1453466 1453581

II 1453677 1453691

II 1453763 1454398

II 1454473 1454588

II 1454656 1454716

II 1454831 1454856

II 1454935 1454959

II 1455130 1455160

II 1455746 1455749

II 1455917 1456965

II 1457122 1457314

II 1457391 1457723

II 1457846 1457976

II 1458025 1458221

II 1458311 1458741

II 1458819 1462184

II 1462757 1462761

II 1468019 1468226

II 1470879 1471086

II 1483038 1483148

II 1486456 1486459

II 1486602 1486673

II 1486893 1486903

II 1487528 1487593

II 1487708 1487808

II 1487898 1487916

II 1488115 1488388

II 1488474 1488549

II 1488644 1488649

II 1492095 1492142

II 1511281 1511462

II 1511548 1511695

II 1511789 1512131

II 1512330 1512393

II 1512503 1512553

II 1512803 1512957

II 1513069 1513414

II 1513623 1513728

II 1514194 1514409

II 1515573 1515583

II 1517021 1517027

II 1535509 1535629

II 1536825 1536918

II 1539468 1539559

II 1540383 1540472

II 1540987 1541129

II 1541382 1541600

II 1542206 1542247

II 1542527 1542530

II 1542626 1542696

II 1542825 1542922

II 1543015 1543025

II 1543120 1543136

II 1543254 1543314

II 1543557 1543612

II 1556193 1556253

II 1558983 1559044

II 1560252 1560298

II 1561336 1561405

II 1564405 1564414

II 1564536 1564694

II 1564780 1564815

II 1564906 1565046

II 1565146 1565318

II 1565574 1565591

II 1565762 1565807

II 1566357 1566565

II 1566657 1566879

II 1585213 1585259

II 1588809 1588850

II 1590459 1590486

II 1591443 1591459

II 1591725 1591816

II 1592064 1592065

II 1592194 1592233

II 1592603 1592628

II 1595847 1595856

II 1595998 1596323

II 1596421 1596434

II 1596521 1596573

II 1596818 1596882

II 1596984 1597168

II 1597416 1597450

II 1617938 1617966

II 1619666 1619673

II 1622426 1622502

II 1627352 1627451

II 1627547 1627669

II 1627764 1627813

II 1627907 1627987

II 1629704 1629772

II 1630458 1632152

II 1632254 1632303

II 1632380 1632423

II 1632580 1632588

II 1633118 1633206

II 1633539 1633560

II 1633760 1633863

II 1633962 1635660

II 1635868 1635899

II 1636425 1636453

II 1641803 1641822

II 1641941 1642079

II 1642169 1642222

II 1642666 1642678

II 1642757 1642899

II 1643049 1643146

II 1643326 1643328

II 1643714 1643851

II 1643926 1644038

II 1644133 1644247

II 1651555 1651558

II 1652183 1652190

II 1657685 1657734

II 1657833 1657893

II 1657985 1657992

II 1658158 1658169

II 1658279 1658288

II 1659591 1659673

II 1659750 1659756

II 1660006 1660035

II 1660122 1660128

II 1660245 1660260

II 1661042 1661058

II 1661488 1661656

II 1662301 1662316

II 1662727 1662772

II 1665949 1665963

II 1666050 1666066

II 1700791 1700861

II 1701348 1701428

II 1701640 1701753

II 1701814 1701847

II 1712322 1712419

II 1716930 1717050

II 1717440 1717454

II 1719613 1719616

II 1720211 1720267

II 1720658 1720795

II 1727810 1727831

II 1727882 1727925

II 1741856 1741892

II 1742187 1742207

II 1743370 1743404

II 1743701 1743718

II 1751603 1751611

II 1751720 1751820

II 1752001 1752053

II 1752446 1752633

II 1755786 1755801

II 1755903 1755917

II 1767777 1767814

II 1768778 1768884

II 1769392 1769407

II 1769502 1769602

II 1769696 1769748

II 1769887 1769968

II 1770334 1770381

II 1770777 1770845

II 1770939 1770959

II 1771292 1771375

II 1771470 1771617

II 1772167 1772354

II 1772593 1772696

II 1773217 1773236

II 1773327 1773429

II 1773522 1773575

II 1773709 1773791

II 1774000 1774015

II 1774112 1774207

II 1774667 1774736

II 1774828 1774849

II 1775185 1775266

II 1775364 1775548

II 1775957 1776142

II 1777401 1777433

II 1777677 1777693

II 1777788 1777855

II 1791377 1791517

II 1791806 1791930

II 1792977 1792989

II 1793236 1793247

II 1793340 1794845

II 1795332 1796805

II 1796899 1796913

II 1797296 1797559

II 1797642 1797816

II 1797963 1798103

II 1798190 1798414

II 1798581 1799192

II 1800011 1800133

II 1800428 1800559

II 1809104 1809258

II 1809336 1809359

II 1809529 1810188

II 1810275 1810547

II 1810640 1811089

II 1812834 1813588

II 1813708 1813860

II 1814035 1814170

II 1814340 1814526

II 1814703 1814797

II 1814884 1814946

II 1815040 1815902

II 1823670 1823715

II 1824480 1824558

II 1824649 1824687

II 1825279 1825354

II 1825466 1825479

II 1825927 1825941

II 1826520 1826618

II 1826707 1826835

II 1826927 1827039

II 1827155 1827203

II 1827824 1827908

II 1828256 1828342

II 1828484 1828535

II 1828698 1828706

II 1828830 1828845

II 1837420 1837534

II 1838416 1838528

II 1851245 1851277

II 1852183 1852218

II 1854133 1854144

II 1855449 1855600

II 1855687 1855833

II 1856020 1856027

II 1856725 1856733

II 1857827 1857846

II 1858042 1858075

II 1860533 1860550

II 1861611 1861652

II 1861997 1862259

II 1862351 1862471

II 1893256 1893322

II 1893392 1893457

II 1900669 1900671

II 1903821 1903897

II 1911344 1911409

II 1911702 1911774

II 1917034 1917045

II 1924977 1924984

II 1935521 1935580

II 1935667 1936443

II 1936517 1937053

II 1941035 1941061

II 1941151 1941211

II 1941340 1941363

II 1942485 1942494

II 1942786 1942787

II 1942967 1942969

II 1944289 1944310

II 1944406 1944467

II 1944590 1944617

II 1948690 1948728

II 1949591 1949627

II 1950085 1950102

II 1950184 1950250

II 1950479 1950663

II 1950730 1950767

II 1950857 1950966

II 1951132 1951167

II 1951300 1951408

II 1951650 1951699

II 1951885 1952027

II 1952113 1952135

II 1952301 1952322

II 1952564 1952601

II 1952714 1952878

II 1954527 1954537

II 1955619 1955620

II 1955852 1955860

II 1955945 1955967

II 1956388 1956480

II 1956628 1956687

II 1956816 1956848

II 1957065 1957072

II 1957174 1957212

II 1957543 1957647

II 1957823 1957842

II 1959237 1959250

II 1959498 1959555

II 1959652 1959752

II 1960129 1960592

II 1960723 1960959

II 1961311 1961366

II 1961458 1968632

II 1968711 1976398

II 1976482 1976623

II 1976680 1986050

II 1986141 1986457

II 1986635 1986696

II 1996821 1996914

II 1997200 1997231

II 1997481 1997502

II 1997561 1997599

II 1997833 1997893

II 1998038 1998132

II 1998556 1998573

II 1998665 1998669

II 1999088 1999136

II 1999290 1999296

II 2001023 2001090

II 2001160 2001168

II 2001302 2001306

II 2001936 2002456

II 2002556 2005459

II 2005549 2005935

II 2006023 2007485

II 2007578 2007582

II 2007677 2010029

II 2010312 2010481

II 2010678 2010686

II 2010819 2010846

II 2015375 2015453

II 2015548 2015690

II 2015822 2015896

II 2015986 2016024

II 2016120 2016240

II 2016500 2016598

II 2016721 2016898

II 2017035 2017091

II 2017289 2017393

II 2017520 2017603

II 2017691 2018027

II 2018113 2018203

II 2018519 2018530

II 2018580 2018605

II 2018697 2018809

II 2018977 2019136

II 2022984 2023135

II 2023296 2023347

II 2023482 2023520

II 2023685 2023704

II 2023797 2023851

II 2024089 2024102

II 2024368 2024765

II 2024854 2024938

II 2024989 2025086

II 2025185 2025237

II 2025493 2025581

II 2029554 2029565

II 2032451 2032500

II 2036644 2036738

II 2036831 2036847

II 2036898 2036917

II 2037014 2037072

II 2037134 2037141

II 2039768 2039842

II 2040528 2040544

II 2042076 2042170

II 2046600 2046612

II 2046821 2046954

II 2047575 2047598

II 2050445 2050477

II 2050527 2050878

II 2050935 2050976

II 2051044 2051126

II 2051205 2051238

II 2055959 2055966

II 2067909 2067942

II 2101168 2101196

II 2105865 2105896

II 2111712 2111778

II 2112216 2112234

II 2118189 2119126

II 2119203 2119256

II 2119426 2119551

II 2124425 2124514

II 2124793 2124993

II 2128906 2129109

II 2130467 2130478

II 2131443 2131531

II 2145195 2145275

II 2149918 2150004

II 2153837 2153903

II 2155290 2155450

II 2155602 2155729

II 2155964 2156090

II 2156184 2156188

II 2156330 2156472

II 2156650 2156715

II 2157241 2157248

II 2157519 2157526

II 2158531 2158545

II 2158704 2158750

II 2160624 2160653

II 2160743 2160814

II 2161066 2161093

II 2161768 2161859

II 2161948 2161975

II 2162478 2162527

II 2162778 2162781

II 2163317 2163330

II 2163520 2163564

II 2164312 2164490

II 2164582 2164727

II 2171245 2171845

II 2171935 2172054

II 2176164 2176236

II 2216355 2216360

II 2218574 2218582

II 2218674 2218746

II 2224929 2224993

II 2225354 2225412

II 2232462 2232474

II 2232946 2232972

II 2238563 2238661

II 2239412 2239512

II 2241464 2241497

II 2255121 2255144

II 2256043 2256068

II 2256521 2256585

II 2260145 2260159

II 2260257 2260304

II 2264510 2264573

II 2265020 2265047

II 2265754 2265778

II 2266230 2266380

II 2266977 2267043

II 2267265 2267453

II 2267575 2267653

II 2267833 2267911

II 2268003 2268407

II 2268501 2268512

II 2269973 2269990

II 2270091 2270099

II 2270490 2270498

II 2271410 2271515

II 2271743 2271789

II 2274002 2274028

II 2274462 2274464

II 2279678 2279695

II 2280691 2280701

II 2281591 2281652

II 2281712 2281775

II 2282845 2282874

II 2282969 2283005

II 2283667 2283675

II 2283990 2284009

II 2285723 2285758

II 2286112 2286165

II 2290477 2290523

II 2290771 2290874

II 2293102 2293172

II 2293299 2293320

II 2293833 2293988

II 2294411 2294564

II 2297473 2297508

II 2306043 2306092

II 2306234 2306238

II 2306441 2306461

II 2306575 2306620

II 2310056 2310062

II 2310245 2310359

II 2311389 2311407

II 2311574 2311597

II 2313544 2313566

II 2326359 2326416

II 2326776 2326827

II 2344587 2344609

II 2347728 2347828

II 2348099 2348141

II 2348477 2348522

II 2348579 2348592

II 2348801 2349005

II 2349057 2349080

II 2349155 2349224

II 2354263 2354298

II 2356650 2356683

II 2361820 2361844

II 2362298 2362367

II 2362766 2362892

II 2362951 2362978

II 2363091 2363153

II 2373300 2373304

II 2383030 2383067

II 2384960 2384976

II 2385082 2385122

II 2386210 2386366

II 2386430 2386941

II 2387025 2387154

II 2387248 2387305

II 2387395 2387454

II 2387707 2387747

II 2387960 2388022

II 2388559 2388565

II 2391992 2392017

II 2392281 2392342

II 2392452 2392568

II 2395656 2395658

II 2398714 2398742

II 2400376 2400380

II 2400382 2400387

II 2401763 2401795

II 2402729 2402968

II 2408708 2408711

II 2424041 2424047

II 2430143 2430295

II 2430453 2430487

II 2430929 2430961

II 2431119 2431275

II 2463554 2463560

II 2464466 2464515

II 2465988 2466018

II 2467148 2467169

II 2471314 2471398

II 2471921 2471998

II 2477475 2477485

II 2496696 2496889

II 2497062 2497095

II 2497198 2497253

II 2497346 2497545

II 2497639 2497657

II 2497720 2497761

II 2497853 2497953

II 2498053 2498074

II 2498166 2498851

II 2500621 2500637

II 2501027 2501036

II 2505485 2505527

II 2509276 2509278

II 2512120 2512122

II 2512619 2512645

II 2516358 2516603

II 2516684 2516704

II 2520372 2520575

II 2522780 2522872

II 2523045 2523133

II 2523204 2523257

II 2529696 2529762

II 2529852 2529901

II 2529979 2530040

II 2530139 2530183

II 2531613 2531654

II 2531705 2531966

II 2532607 2533519

II 2533612 2534498

II 2534607 2534618

II 2534784 2534816

II 2534900 2534956

II 2535076 2535086

II 2535196 2536081

II 2536177 2537083

II 2539443 2539444

II 2547087 2547089

II 2550466 2550576

II 2550667 2550848

II 2550931 2551092

II 2551313 2551968

II 2553814 2553832

II 2554236 2554322

II 2554485 2554553

II 2554642 2554656

II 2554933 2554996

II 2555086 2555236

II 2555424 2555504

II 2555676 2555887

II 2555976 2556093

II 2556258 2556403

II 2556716 2556737

II 2556881 2556900

II 2565890 2565998

II 2566063 2566105

II 2566453 2566492

II 2567827 2567850

II 2575350 2575371

II 2577086 2577503

II 2577580 2577998

II 2584838 2584947

II 2585036 2585143

II 2588058 2588080

II 2588495 2588546

II 2590844 2591083

II 2593125 2593305

II 2593374 2596911

II 2596977 2599659

II 2599714 2599802

II 2599860 2599915

II 2600120 2600241

II 2600352 2600389

II 2600484 2600622

II 2602939 2603082

II 2603171 2603182

II 2603310 2603335

II 2603443 2603453

II 2603897 2603912

II 2606379 2606416

II 2606534 2606597

II 2606743 2606751

II 2606870 2606900

II 2607014 2607017

II 2607471 2607483

II 2611422 2611439

II 2611858 2611909

II 2612531 2612564

II 2612680 2612725

II 2613770 2613964

II 2614696 2614787

II 2614857 2614994

II 2615147 2615267

II 2616887 2617014

II 2619398 2619529

II 2620592 2620624

II 2620742 2620786

II 2623508 2623530

II 2632199 2632271

II 2632367 2632555

II 2639838 2639968

II 2644824 2644875

II 2645689 2645691

II 2645901 2645913

II 2646119 2646153

II 2646624 2646668

II 2646761 2646835

II 2646932 2646996

II 2648926 2648950

II 2655816 2655837

II 2660989 2660997

II 2664199 2664221

II 2664357 2664616

II 2664820 2665033

II 2665381 2665403

II 2665979 2666120

II 2666215 2666276

II 2666738 2667109

II 2667200 2667319

II 2667578 2667876

II 2668047 2668146

II 2669746 2669795

II 2672008 2672017

II 2672567 2672629

II 2673027 2673105

II 2673198 2673219

II 2673498 2673514

II 2673633 2673702

II 2673798 2673915

II 2674140 2674200

II 2674441 2674516

II 2675542 2675653

II 2675826 2675890

II 2676098 2676145

II 2676832 2676866

II 2687592 2687775

II 2688881 2689033

II 2697355 2697359

II 2699271 2699274

II 2711686 2711721

II 2711841 2712086

II 2712223 2712282

II 2712369 2712469

II 2712670 2712716

II 2713152 2713577

II 2713727 2713745

II 2713860 2713884

II 2713997 2714024

II 2714224 2714237

II 2716940 2716942

II 2735824 2735842

II 2736305 2736316

II 2746312 2746324

II 2746370 2746380

II 2759774 2759854

II 2759931 2759936

II 2760000 2760020

II 2839354 2839398

II 2860377 2860386

II 2861581 2861595

II 2883582 2883700

II 2883791 2883952

II 2884045 2884065

II 2888074 2888333

II 2890604 2890862

II 2891678 2891871

II 2891965 2892030

II 2892208 2892290

II 2892375 2892518

II 2892602 2892748

II 2892799 2892835

II 2892912 2893062

II 2897181 2897242

II 2897419 2897777

II 2897920 2897938

II 2898115 2898468

II 2898829 2898845

II 2905943 2906026

II 2906115 2906238

II 2906329 2906477

II 2906562 2906794

II 2909045 2909241

II 2909338 2909401

II 2911175 2911191

II 2911326 2911465

II 2912417 2912455

II 2912570 2912709

II 2950140 2950241

II 2950297 2950749

II 2950844 2950901

II 2950996 2951081

II 2951153 2951236

II 2955470 2955550

II 2955625 2955703

II 2957455 2957459

II 2957460 2957463

II 2971664 2971740

II 2980325 2982577

II 2989125 2989308

II 2996360 2996425

II 3002084 3002228

II 3003548 3003691

II 3007275 3007386

II 3007482 3007511

II 3007608 3007624

II 3007721 3007764

II 3009578 3009693

II 3011266 3011374

II 3011704 3011748

II 3011844 3011861

II 3011958 3011985

II 3012085 3012197

II 3021561 3021565

II 3021628 3021630

II 3021631 3021633

II 3021737 3021818

II 3027294 3027360

II 3027442 3027590

II 3027677 3027818

II 3027874 3028099

II 3031190 3031285

II 3031569 3031579

II 3031705 3031715

II 3031892 3031959

II 3032096 3032106

II 3032288 3032434

II 3032553 3032658

II 3033815 3033821

II 3033886 3033920

II 3041518 3042403

II 3048391 3048426

II 3050847 3050866

II 3053413 3053431

II 3058515 3058517

II 3058518 3058521

II 3078259 3078260

II 3095035 3095090

II 3095185 3095333

II 3098875 3098916

II 3099173 3099266

II 3102512 3102587

II 3103804 3103863

II 3103938 3103989

II 3106181 3106256

II 3106347 3106478

II 3106574 3106762

II 3106844 3107104

II 3126857 3126874

II 3128076 3128099

II 3132547 3132683

II 3132774 3132804

II 3132890 3133182

II 3133322 3133331

II 3133411 3133833

II 3142196 3142293

II 3142370 3142466

II 3143098 3143128

II 3144794 3144827

II 3156020 3156083

II 3158397 3158459

II 3159566 3159577

II 3159647 3160481

II 3160574 3161638

II 3161695 3162744

II 3162807 3162852

II 3162946 3163384

II 3163480 3164908

II 3164947 3165679

II 3165762 3167591

II 3167673 3168076

II 3168137 3168501

II 3176968 3177190

II 3177280 3177317

II 3177401 3177439

II 3177524 3177562

II 3177746 3177753

II 3180044 3180125

II 3180191 3180262

II 3181605 3181640

II 3182327 3182381

II 3182587 3182650

II 3182822 3182937

II 3183395 3183468

II 3183557 3183591

II 3183682 3183754

II 3183871 3183885

II 3184136 3184155

II 3184251 3184330

II 3184648 3184708

II 3185259 3185268

II 3187607 3187657

II 3187849 3187850

II 3189352 3189451

II 3189622 3189917

II 3190180 3190300

II 3190388 3190761

II 3191221 3191277

II 3191374 3191510

II 3191624 3191646

II 3191995 3192211

II 3192418 3192684

II 3192815 3192837

II 3201839 3201859

II 3209674 3209694

II 3215927 3215991

II 3216085 3216159

II 3216260 3216302

II 3217808 3217863

II 3226580 3228836

II 3231922 3231988

II 3233050 3233092

II 3233283 3233433

II 3233565 3233628

II 3233967 3233973

II 3234276 3234285

II 3234337 3234357

II 3234411 3234421

II 3234494 3234589

II 3242774 3242830

II 3243403 3243427

II 3271783 3272217

II 3289252 3289270

II 3289438 3289444

II 3289887 3290005

II 3290695 3290749

II 3305690 3305742

II 3307539 3307553

II 3307738 3308874

II 3309941 3309994

II 3310060 3310073

II 3316535 3316604

II 3316663 3316732

II 3316827 3316829

II 3317107 3317186

II 3317281 3317404

II 3319383 3319419

II 3319565 3319571

II 3319844 3320171

II 3320320 3320673

II 3320779 3321128

II 3321281 3321604

II 3321864 3321871

II 3322017 3322054

II 3342500 3342872

II 3343006 3343070

II 3345348 3345370

II 3345739 3345757

II 3348288 3348323

II 3350622 3350629

II 3350874 3350889

II 3351090 3351186

II 3351273 3351417

II 3351467 3351563

II 3351677 3352310

II 3354329 3354428

II 3354603 3354605

II 3354961 3355019

II 3355112 3355197

II 3355704 3355707

II 3356127 3356156

II 3356206 3356939

II 3357029 3357077

II 3357336 3357432

II 3357520 3357565

II 3357714 3357735

II 3357826 3358047

II 3358137 3358181

II 3358255 3358267

II 3358356 3358441

II 3358526 3358569

II 3358825 3358885

II 3360678 3360738

II 3360877 3361243

II 3365423 3365500

II 3365761 3365845

II 3366132 3366146

II 3366449 3366461

II 3374538 3374557

II 3410258 3410293

II 3435893 3435932

II 3447085 3447100

II 3456198 3457137

II 3457229 3457335

II 3458039 3458042

II 3460493 3460496

II 3460580 3460587

II 3464867 3465249

II 3465373 3465663

II 3465845 3466225

II 3466288 3466419

II 3466530 3467145

II 3467220 3467757

II 3467819 3468248

II 3468332 3468602

II 3468760 3468992

II 3469180 3469187

II 3469451 3469548

II 3481006 3481018

II 3481903 3481920

II 3482005 3482049

II 3490542 3490548

II 3491907 3491911

II 3507819 3507972

II 3508083 3508252

II 3511359 3511516

II 3511651 3511810

II 3512567 3512639

II 3521672 3521713

II 3522170 3522200

II 3522391 3522422

II 3522534 3522642

II 3522881 3523047

II 3523409 3523479

II 3548085 3548100

II 3548167 3548298

II 3548444 3548575

II 3549030 3549186

II 3549288 3549437

II 3567640 3567641

II 3582976 3583020

II 3584506 3584521

II 3585112 3585229

II 3586549 3586565

II 3586651 3590811

II 3590960 3595123

II 3601700 3602861

II 3622517 3622600

II 3622869 3623050

II 3625899 3626048

II 3629473 3629558

II 3629683 3629688

II 3632554 3632701

II 3633127 3633132

II 3633265 3633445

II 3634697 3634731

II 3636852 3636854

II 3638424 3638431

II 3638649 3638702

II 3644771 3644801

II 3647793 3647849

II 3648066 3648074

II 3650675 3650708

II 3655922 3655939

II 3695224 3695229

II 3696083 3696135

II 3696248 3696566

II 3696818 3696851

II 3696947 3696992

II 3697089 3697093

II 3697191 3697295

II 3697500 3697554

II 3697616 3697714

II 3697805 3697815

II 3734491 3734557

II 3734622 3734710

II 3734767 3734881

II 3735062 3735095

II 3735182 3735234

II 3735340 3735358

II 3735493 3735565

II 3735682 3735697

II 3750074 3750082

II 3750176 3750214

II 3750426 3750458

II 3750554 3750562

II 3754376 3755192

II 3755340 3755428

II 3761801 3761810

II 3761876 3761955

II 3762184 3762246

II 3762341 3762371

II 3762659 3762681

II 3762786 3762793

II 3769149 3769855

II 3769964 3770202

II 3770292 3770676

II 3796703 3796705

II 3796706 3796707

II 3796955 3796970

II 3813667 3813736

II 3813853 3813959

II 3814052 3814065

II 3816540 3816621

II 3817880 3817896

II 3818624 3818696

II 3819059 3819091

II 3819177 3819196

II 3819282 3819369

II 3821780 3821986

II 3822079 3822121

II 3822675 3822679

II 3822742 3822806

II 3825023 3825148

II 3849501 3849512

II 3849706 3849722

II 3849851 3849899

II 3850252 3850311

II 3853242 3853391

II 3854001 3854248

II 3854407 3854506

II 3863301 3863393

II 3863556 3863805

II 3864347 3864501

II 3867033 3867092

II 3867447 3867490

II 3867624 3867639

II 3867838 3867846

II 3868655 3868670

II 3868721 3868728

II 3883643 3883713

II 3883971 3883993

II 3884084 3884111

II 3884263 3884277

II 3884372 3884395

II 3884463 3884505

II 3886699 3886790

II 3886983 3887028

II 3937608 3937727

II 3937891 3937919

II 3938074 3938086

II 3938177 3938189

II 3938273 3938451

II 3939362 3941618

II 3941817 3942930

II 3942999 3943092

II 3943184 3943368

II 3943422 3943473

II 3943566 3943584

II 3943679 3945187

II 3945405 3946633

II 3994012 3994060

II 3994432 3994510

II 3994751 3994763

II 4048272 4048377

II 4049166 4049188

II 4049504 4049507

II 4049859 4049878

II 4051290 4051524

II 4051617 4051652

II 4051734 4051835

II 4051920 4053060

II 4053155 4054188

II 4054280 4054401

II 4055633 4055692

II 4055883 4055893

II 4056374 4056406

II 4056653 4056667

II 4057924 4057993

II 4058732 4058762

II 4061475 4061589

II 4061792 4061836

II 4061901 4061909

II 4061975 4062144

II 4062280 4062296

II 4062379 4062384

II 4062477 4062493

II 4065417 4065441

II 4065509 4065522

II 4065931 4065943

II 4078273 4078451

II 4101719 4101927

II 4102051 4102113

II 4102237 4102239

II 4113613 4113721

II 4126417 4126448

II 4130310 4130420

II 4135174 4135188

II 4135239 4135371

II 4135473 4135488

II 4168718 4169490

II 4202904 4202921

II 4202996 4202998

II 4207682 4207808

II 4231932 4231936

II 4232110 4232184

II 4232362 4232570

II 4232788 4232792

II 4232983 4233007

II 4233315 4233485

II 4233700 4233706

II 4233956 4234064

II 4234651 4234677

II 4235657 4235670

II 4237255 4237264

II 4259501 4259596

II 4259748 4259822

II 4260061 4260073

II 4260163 4260519

II 4260813 4260849

II 4260916 4260964

II 4261625 4261631

II 4285797 4285919

II 4286230 4286349

II 4308496 4308612

II 4308662 4308776

II 4308850 4309065

II 4309111 4309126

II 4316769 4316788

II 4317988 4318187

II 4318331 4318688

II 4318775 4318789

II 4339151 4339258

II 4339367 4339452

II 4339602 4339700

II 4339711 4339718

II 4339780 4339863

II 4386719 4386774

II 4387002 4387054

II 4414878 4414981

II 4415110 4415139

II 4415232 4415639

II 4415732 4415741

II 4432881 4432912

II 4437684 4437693

II 4438118 4438123

II 4438219 4438224

II 4442572 4442710

II 4475690 4475695

II 4475819 4475925

II 4476018 4476275

II 4476364 4476705

II 4476801 4476845

II 4479107 4479120

II 4495291 4495463

II 4496565 4496819

II 4497421 4497483

II 4497587 4497789

II 4546136 4546203

II 4553954 4554083

II 4554221 4554608

II 4554664 4554992

II 4555066 4555290

II 4555384 4555499

II 4555589 4555905

II 4555977 4556177

II 4641359 4641615

II 4641719 4642203

II 4642587 4642609

II 4642702 4642786

II 4642859 4643007

II 4643057 4643592

II 4643731 4643761

II 4643904 4644263

II 4644329 4644415

II 4644502 4644680

II 4644740 4644782

II 4644839 4644958

II 4651305 4651361

II 4657133 4657145

II 4684265 4684305

II 4684356 4684366

II 4701443 4701751

II 4704429 4704455

II 4750506 4750600

II 4750752 4750902

II 4786123 4786204

II 4825461 4825628

II 4834253 4834286

II 4835575 4835608

II 4837029 4837059

II 4841422 4841594

II 4841829 4841880

II 4841999 4842235

II 4842510 4842555

II 4842760 4842802

II 4860166 4860203

II 4860412 4860460

II 4863687 4863927

II 4864085 4864185

II 4864235 4864282

II 4864363 4864370

II 4864518 4864597

II 4865063 4865076

II 4865190 4865201

II 4865289 4865343

II 4865576 4865748

II 4867105 4867131

II 4867395 4867464

II 4872652 4872675

II 4872942 4873009

II 4878721 4878734

II 4892389 4892439

II 4892534 4892580

II 4894345 4894361

II 4894592 4894604

II 4895125 4895265

II 4895572 4895617

II 4895784 4895788

II 4895884 4895912

II 4896025 4896067

II 4896218 4896238

II 4896334 4896420

II 4896514 4896570

II 4896717 4896807

II 4896931 4897319

II 4897411 4897435

II 4897563 4897904

II 4897999 4898104

II 4898238 4898382

II 4898471 4898521

II 4898761 4898785

II 4898921 4898946

II 4903995 4904033

II 4916375 4916527

II 4926971 4928065

II 4928236 4928357

II 4928651 4928678

II 4928772 4928787

II 4928839 4929022

II 4929128 4929197

II 4929302 4929345

II 4929596 4929623

II 4929688 4929723

II 4933076 4933218

II 4933279 4933332

II 4939846 4939919

II 4940094 4940112

II 4940170 4940181

II 4963383 4963499

II 4964219 4964336

II 5012937 5012940

II 5013099 5013136

II 5013193 5013309

II 5064367 5064558

II 5073409 5073572

II 5073669 5073848

II 5073952 5074342

II 5074401 5074640

II 5074730 5074982

II 5075035 5075404

II 5075483 5076059

II 5076113 5076382

II 5076478 5076721

II 5076956 5077015

II 5077107 5077286

II 5077339 5077378

II 5097244 5097327

II 5097411 5097487

II 5097576 5097577

II 5097650 5097692

II 5097760 5097862

II 5100263 5100279

II 5101200 5101221

II 5102491 5102517

II 5115344 5115350

II 5144639 5144648

II 5144733 5144836

II 5145041 5145088

II 5145186 5145215

II 5145317 5145361

II 5145410 5145567

II 5145624 5145759

II 5145808 5145909

II 5145981 5146693

II 5156910 5156966

II 5157042 5157047

II 5158186 5158214

II 5158409 5158472

II 5158847 5158907

II 5194625 5194682

II 5200053 5200113

II 5200333 5200372

II 5240036 5241042

II 5251474 5251719

II 5251885 5251991

II 5252080 5252128

II 5252219 5252228

II 5252324 5252359

II 5252452 5252705

II 5252758 5252844

II 5284541 5284695

II 5288179 5288181

II 5319210 5319217

II 5320981 5320986

II 5321081 5321096

II 5321186 5321263

II 5343460 5343477

II 5343590 5343610

II 5344065 5344186

II 5346591 5347236

II 5347325 5348071

II 5348167 5348217

II 5348311 5348776

II 5348869 5349789

II 5349913 5350558

II 5353574 5353659

II 5401237 5401313

II 5401439 5401467

II 5408405 5408670

II 5410796 5411061

II 5441869 5441912

II 5442449 5442495

II 5494063 5494133

II 5495083 5495088

II 5495221 5495414

II 5519457 5519975

II 5534877 5534914

II 5535009 5535035

II 5535359 5536083

II 5539363 5540088

II 5540417 5540444

II 5540540 5540575

II 5542805 5542810

II 5544240 5544243

II 5546177 5546358

II 5550488 5550498

II 5550557 5550558

II 5550640 5550660

II 5550773 5550804

II 5550873 5550997

II 5551085 5551191

II 5551259 5551504

II 5560987 5561137

II 5564903 5564936

II 5564992 5565007

II 5565207 5565230

II 5565809 5566958

II 5567052 5567091

II 5567361 5567465

II 5567522 5568668

II 5568809 5569030

II 5569117 5569156

II 5569248 5569371

II 5569510 5569568

II 5569656 5569818

II 5621608 5622854

II 5622951 5624232

II 5624327 5624464

II 5624839 5624867

II 5626056 5626064

II 5626448 5626450

II 5626521 5626537

II 5626659 5627176

II 5627735 5627800

II 5628098 5628119

II 5630070 5631316

II 5631412 5632697

II 5632789 5632927

II 5633259 5633285

II 5634410 5634413

II 5673634 5673723

II 5675149 5675150

II 5676171 5676194

II 5693102 5693208

II 5709203 5709383

II 5709433 5709448

II 5709539 5709687

II 5732835 5732839

II 5746426 5746592

II 5753474 5753718

II 5754696 5754751

II 5755605 5755661

II 5756030 5756271

II 5757648 5757718

II 5758091 5758161

II 5778517 5778610

II 5778938 5778951

II 5797143 5797156

II 5798217 5798233

II 5810166 5810172

II 5813313 5813315

II 5815501 5815610

II 5815704 5815881

II 5815934 5816262

II 5817725 5817729

II 5844887 5844898

II 5865195 5865232

II 5866626 5866655

II 5876656 5876663

II 5877012 5877014

II 5979624 5982168

II 6008338 6008445

II 6008567 6008578

II 6008863 6009012

II 6009253 6009422

II 6009568 6009576

II 6013029 6013066

II 6013154 6013306

II 6013546 6013677

II 6013755 6013778

II 6013868 6013880

II 6013969 6014042

II 6014166 6014241

II 6054490 6054542

II 6055078 6055126

II 6056278 6056311

II 6058102 6058190

II 6059000 6059008

II 6059983 6060005

II 6064169 6064176

II 6064307 6064400

II 6064535 6064581

II 6064674 6064692

II 6065065 6065175

II 6065350 6065378

II 6065539 6065604

II 6065825 6065896

II 6068260 6069501

II 6069594 6069724

II 6069802 6070789

II 6074143 6074150

II 6074215 6075794

II 6075872 6076518

II 6076594 6077414

II 6079295 6079302

II 6079587 6079700

II 6079896 6079903

II 6080047 6080072

II 6080318 6080335

II 6081321 6081385

II 6081541 6081545

II 6081728 6081785

II 6082058 6082204

II 6082324 6082338

II 6082430 6082583

II 6083349 6083377

II 6083988 6084032

II 6084383 6084536

II 6084809 6084842

II 6085102 6085340

II 6085536 6085557

II 6085742 6085752

II 6086118 6086176

II 6086232 6086316

II 6086412 6086443

II 6086541 6086633

II 6086710 6086713

II 6086862 6086964

II 6087062 6087087

II 6087171 6087184

II 6087285 6087366

II 6087529 6087573

II 6089943 6089945

II 6090226 6090346

II 6090539 6090550

II 6090691 6090714

II 6090968 6090984

II 6129326 6129414

II 6163567 6163597

II 6163669 6163717

II 6166826 6166872

II 6167146 6167163

II 6185326 6186437

II 6239906 6239953

II 6240493 6240582

II 6268656 6268691

II 6269087 6269152

II 6269384 6269395

II 6269700 6269701

II 6269789 6269824

II 6269911 6269952

II 6296864 6296921

II 6297056 6297125

II 6298079 6298211

II 6298336 6298366

II 6298461 6298735

II 6299442 6299713

II 6299808 6299833

II 6299964 6300096

II 6301707 6302125

II 6302220 6302315

II 6302529 6302605

II 6302669 6302676

II 6304800 6304821

II 6311068 6311115

II 6420673 6420686

II 6421048 6421074

II 6421550 6421597

II 6425175 6425248

II 6425322 6425341

II 6433337 6433366

II 6434674 6434680

II 6434848 6434891

II 6437361 6437398

II 6437612 6437721

II 6438453 6438560

II 6438770 6438810

II 6462236 6462356

II 6504957 6505139

II 6505220 6505264

II 6505319 6505427

II 6505736 6505873

II 6505986 6506009

II 6506229 6506874

II 6506968 6507257

II 6507381 6507790

II 6507885 6507888

II 6507945 6508031

II 6508278 6508313

II 6508390 6508420

II 6508927 6508986

II 6509086 6509107

II 6509248 6509285

II 6548071 6548125

II 6548271 6548413

II 6548550 6548617

II 6548733 6548762

II 6549060 6549175

II 6549275 6549429

II 6571262 6572790

II 6576040 6576053

II 6576124 6576153

II 6576770 6576780

II 6579764 6579767

II 6580234 6580254

II 6580580 6580653

II 6580922 6581258

II 6627461 6627799

II 6628071 6628138

II 6628717 6628743

II 6629191 6629194

II 6657521 6657545

II 6661983 6662037

II 6662131 6662546

II 6662711 6662719

II 6662835 6662914

II 6663010 6663165

II 6663445 6663530

II 6670350 6670403

II 6670497 6670914

II 6671072 6671083

II 6671291 6671365

II 6671462 6671616

II 6671898 6671978

II 6673178 6673204

II 6673340 6673368

II 6674742 6674787

II 6675004 6675011

II 6679115 6679148

II 6679279 6679307

II 6697649 6697660

II 6731416 6731420

II 6774004 6774007

II 6775223 6775426

II 6775513 6775551

II 6775775 6775793

II 6776149 6776217

II 6776384 6776451

II 6778781 6778799

II 6778853 6778864

II 6795112 6795499

II 6795556 6795658

II 6795743 6797144

II 6797237 6797906

II 6797988 6798221

II 6848462 6848570

II 6880841 6880859

II 6884183 6884203

II 6905879 6905886

II 6906387 6906404

II 6906492 6906515

II 6908191 6908212

II 6913958 6913964

II 6938195 6938424

II 6938684 6938707

II 6938801 6938909

II 6938998 6939141

II 6968434 6968470

II 6977295 6977369

II 6977776 6977826

II 6981326 6981368

II 6981822 6981827

II 6982160 6982658

II 6982751 6982933

II 6983026 6983097

II 6983203 6983426

II 6983519 6983562

II 6983611 6983678

II 6983823 6983837

II 6983923 6984170

II 6984260 6984270

II 6984436 6985212

II 6985351 6985430

II 6985486 6985567

II 6997376 6997470

II 6997627 6997654

II 6997985 6998051

II 6998181 6998188

II 6998234 6998317

II 6998693 6998766

II 7000581 7000643

II 7000809 7000892

II 7001080 7001109

II 7001550 7001568

II 7020759 7020774

II 7020855 7020868

II 7020984 7021042

II 7021243 7021252

II 7023165 7023277

II 7025361 7025365

II 7039602 7039604

II 7039798 7039813

II 7039933 7039970

II 7040263 7040295

II 7041763 7041798

II 7041877 7041929

II 7042083 7042119

II 7042226 7042269

II 7042451 7042745

II 7043162 7044104

II 7044198 7044426

II 7045712 7045726

II 7045813 7045834

II 7045985 7046211

II 7046303 7047244

II 7049378 7049404

II 7066101 7066410

II 7066609 7066636

II 7066733 7066738

II 7067180 7067326

II 7068952 7068980

II 7077360 7077379

II 7077569 7077662

II 7078083 7078232

II 7078672 7078677

II 7078772 7078803

II 7078996 7081253

II 7081335 7081653

II 7084247 7084260

II 7084388 7084399

II 7085254 7085316

II 7085393 7085452

II 7086029 7086048

II 7086202 7086382

II 7086481 7086553

II 7086711 7086720

II 7088428 7088500

II 7120508 7120738

II 7120829 7121503

II 7123958 7124002

II 7124825 7124889

II 7125911 7125923

II 7129302 7129341

II 7130075 7130116

II 7130513 7130746

II 7130840 7131510

II 7153189 7153226

II 7156188 7156191

II 7162537 7162548

II 7163924 7163984

II 7165197 7165200

II 7171926 7171944

II 7203953 7204410

II 7206394 7206853

II 7262601 7262618

II 7349343 7349348

II 7364695 7364747

II 7365090 7365115

II 7365496 7365523

II 7365950 7365952

II 7366891 7367454

II 7370231 7370360

II 7370759 7370873

II 7371880 7372440

II 7372588 7372597

II 7378519 7378547

II 7378602 7378671

II 7378824 7378869

II 7378915 7379040

II 7379741 7379801

II 7379911 7379942

II 7380130 7380243

II 7391034 7391870

II 7435264 7435394

II 7435574 7435645

II 7435867 7436069

II 7437752 7437874

II 7438020 7439279

II 7439442 7439583

II 7439897 7439929

II 7440131 7440194

II 7440263 7440320

II 7440415 7440504

II 7440598 7440674

II 7440793 7440818

II 7441298 7441387

II 7441493 7441584

II 7441858 7441919

II 7442246 7442263

II 7442356 7442423

II 7442544 7442551

II 7442654 7442730

II 7443077 7443125

II 7443287 7443346

II 7443441 7443852

II 7443962 7444029

II 7444295 7446728

II 7447083 7447167

II 7447329 7447483

II 7447553 7447691

II 7447759 7448021

II 7448117 7448794

II 7448854 7448886

II 7450240 7450760

II 7450839 7453271

II 7453331 7454596

II 7454741 7454865

II 7454958 7455161

II 7455380 7455452

II 7455632 7455766

II 7460362 7460511

II 7460641 7460721

II 7471053 7471071

II 7471255 7471299

II 7471390 7471448

II 7471565 7471581

II 7475147 7475198

II 7475294 7475315

II 7475398 7475465

II 7479001 7479014

II 7479099 7479137

II 7486650 7486688

II 7486777 7486811

II 7502336 7504588

II 7516712 7518243

II 7546235 7546266

II 7572853 7573264

II 7573841 7574213

II 7584219 7584255

II 7584461 7584490

II 7584869 7584872

II 7584996 7585007

II 7626229 7626250

II 7784871 7784930

II 7785016 7785040

II 7801555 7801559

II 7801653 7801666

II 7803866 7803909

II 7804129 7804137

II 7804204 7804249

II 7804634 7804689

II 7804845 7804967

II 7805183 7805229

II 7818724 7818744

II 7819013 7819044

II 7826976 7826995

II 7827241 7827273

II 7837634 7837756

II 7894767 7895064

II 7896972 7896983

II 7897275 7897301

II 7897496 7897734

II 7898015 7898044

II 7898321 7898471

II 7898880 7898924

II 7922588 7922680

II 7922767 7923285

II 7923354 7924113

II 7953661 7953696

II 8049718 8049758

II 8097390 8097592

II 8097717 8097777

II 8103493 8103537

II 8108272 8108297

II 8108701 8108714

II 8145737 8145910

II 8215099 8215168

II 8229420 8229620

II 8230010 8230091

II 8230180 8230374

II 8230464 8230511

II 8230571 8230668

II 8230762 8230784

II 8240466 8240905

II 8240995 8241465

II 8241547 8241728

II 8241824 8241840

II 8241921 8242091

II 8244274 8244308

II 8244471 8244502

II 8244895 8244913

II 8245100 8245205

II 8245442 8245760

II 8245854 8245896

II 8246171 8246313

II 8246795 8246809

II 8246942 8247130

II 8247227 8247833

II 8247926 8247972

II 8248079 8248131

II 8262569 8262612

II 8262706 8262762

II 8262996 8263050

II 8263144 8263191

II 8286038 8286149

II 8287692 8287703

II 8288204 8288385

II 8288513 8288667

II 8288735 8288804

II 8288868 8289156

II 8289235 8289506

II 8289641 8289869

II 8289973 8290937

II 8291032 8291045

II 8291098 8291145

II 8291281 8291684

II 8291754 8291982

II 8292107 8292217

II 8292311 8292514

II 8292696 8292700

II 8335121 8335165

II 8335536 8335546

II 8335764 8335940

II 8336033 8336093

II 8347473 8347520

II 8347894 8347898

II 8348118 8348298

II 8348390 8348450

II 8420004 8420035

II 8422331 8422359

II 8422533 8422555

II 8422933 8423007

II 8423410 8423539

II 8423710 8423845

II 8424703 8424730

II 8433085 8433192

II 8459644 8459727

II 8459821 8459883

II 8460146 8460217

II 8460265 8460503

II 8460879 8460922

II 8474051 8474104

II 8474214 8474255

II 8474353 8474954

II 8475051 8475238

II 8475374 8475386

II 8475877 8476018

II 8476289 8476336

II 8476429 8476747

II 8476980 8477092

II 8477276 8477292

II 8477687 8477713

II 8477881 8477911

II 8595105 8595108

II 8668229 8668392

II 8689667 8689684

II 8815364 8815384

II 8815540 8815545

II 8815990 8815996

II 8816129 8816135

II 8824174 8824183

II 8824502 8824530

II 8824748 8824767

II 8837633 8837637

II 8837791 8837835

II 8860139 8860256

II 8860425 8860595

II 8860689 8860812

II 8862023 8862454

II 8862547 8862608

II 8862879 8862990

II 8863085 8863505

II 8867379 8867413

II 8867481 8867540

II 8878334 8878404

II 8975525 8976279

II 8976365 8976979

II 8977030 8977437

II 9041821 9041826

II 9058338 9058373

II 9084855 9084920

II 9085008 9085096

II 9085174 9085191

II 9101475 9101555

II 9101674 9101837

II 9101976 9102039

II 9102114 9102133

II 9102308 9102469

II 9102560 9102687

II 9102875 9102953

II 9103040 9103053

II 9106253 9106440

II 9125786 9125852

II 9150684 9150694

II 9150977 9150990

II 9151312 9151423

II 9151732 9151738

II 9202037 9202241

II 9202294 9202351

II 9202628 9202653

II 9202864 9203434

II 9203494 9203795

II 9203863 9203910

II 9301804 9301830

II 9301916 9301943

II 9302036 9302166

II 9348487 9348521

II 9348571 9348593

II 9348973 9349061

II 9349114 9349133

II 9363451 9363499

II 9374207 9374518

II 9376415 9376419

II 9377593 9377903

II 9381456 9381463

II 9386181 9386238

II 9393455 9394200

II 9394803 9394853

II 9395213 9395266

II 9403723 9403748

II 9404904 9404912

II 9405125 9405211

II 9405300 9405368

II 9452385 9453541

II 9453635 9453792

II 9457250 9457618

II 9457790 9457792

II 9457989 9457998

II 9458240 9458253

II 9458507 9458971

II 9459068 9459189

II 9459391 9459721

II 9459817 9461590

II 9462045 9462506

II 9508340 9508352

II 9508562 9508700

II 9508965 9509332

II 9509440 9509509

II 9513204 9513217

II 9513421 9513560

II 9513839 9514204

II 9514315 9514383

II 9522285 9522379

II 9522448 9522482

II 9534026 9534537

II 9534630 9534718

II 9534869 9534992

II 9535089 9535106

II 9535865 9535970

II 9536194 9536338

II 9536442 9536474

II 9536596 9536620

II 9537031 9537047

II 9562921 9563053

II 9563167 9563173

II 9563514 9563557

II 9566963 9567592

II 9567676 9568501

II 9568595 9568800

II 9568871 9569042

II 9569139 9569256

II 9629724 9629726

II 9630037 9630061

II 9630366 9630404

II 9631834 9631946

II 9632012 9632546

II 9632612 9632944

II 9633010 9633116

II 9633195 9633261

II 9633417 9633503

II 9633577 9633873

II 9633926 9633968

II 9634157 9634170

II 9634332 9634365

II 9634523 9634710

II 9636030 9636042

II 9636155 9636222

II 9636312 9636313

II 9673413 9673529

II 9673622 9673672

II 9673766 9673792

II 9688267 9688473

II 9688547 9688640

II 9688722 9689339

II 9697331 9697498

II 9697591 9697712

II 9697801 9697825

II 9698002 9698225

II 9699679 9699763

II 9699859 9699983

II 9718841 9719013

II 9719126 9719133

II 9719269 9719297

II 9719406 9719422

II 9719618 9719641

II 9719874 9719942

II 9720042 9720111

II 9725981 9726140

II 9726236 9726367

II 9731882 9731909

II 9734609 9734711

II 9752635 9752766

II 9752915 9753051

II 9790439 9790465

II 9790575 9790589

II 9792001 9792010

II 9804814 9804845

II 9808068 9810589

II 9810660 9812242

II 9812549 9812641

II 9812725 9813858

II 9813911 9814356

II 9814544 9816813

II 9816925 9817169

II 9817228 9817554

II 9817641 9819126

II 9819197 9819743

II 9819834 9821849

II 9822026 9822186

II 9822261 9823293

II 9823376 9823908

II 9824019 9824773

II 9824983 9825007

II 9825107 9825333

II 9825651 9825669

II 9825783 9825786

II 9829800 9829854

II 9843465 9843520

II 9843628 9843679

II 9845655 9845692

II 9847494 9847717

II 9847961 9847992

II 9854080 9854105

II 9854237 9854292

II 9856005 9856059

II 9858208 9858251

II 9860252 9860369

II 9864052 9864184

II 9864711 9864743

II 9865023 9865140

II 9866007 9866135

II 9866662 9866884

II 9867121 9867158

II 9912869 9913301

II 9913393 9913654

II 9923342 9923348

II 9923576 9923677

II 9923768 9923795

II 9923887 9924006

II 9924275 9924318

II 9929172 9929186

II 9929262 9929347

II 9929612 9929710

II 9929888 9929916

II 9984465 9984482

II 10004861 10004882

II 10005017 10005029

II 10005120 10005138

II 10005459 10005465

II 10006213 10006215

II 10006288 10006308

II 10006403 10006493

II 10019037 10019063

II 10020514 10020560

II 10020647 10020721

II 10022655 10022662

II 10023197 10023227

II 10023350 10023996

II 10024221 10024270

II 10024392 10024566

II 10024783 10024790

II 10027497 10027546

II 10033711 10034135

II 10034224 10034524

II 10034613 10034959

II 10088186 10088433

II 10117428 10117487

II 10117648 10117709

II 10123112 10123565

II 10123947 10124397

II 10129288 10129292

II 10130634 10130639

II 10131047 10131062

II 10135339 10135341

II 10135437 10135471

II 10171335 10171573

II 10171672 10171686

II 10171782 10171983

II 10205608 10205682

II 10206136 10206196

II 10241167 10241331

II 10247844 10247976

II 10248123 10248192

II 10248277 10248284

II 10248398 10248493

II 10302303 10302339

II 10304902 10304935

II 10328908 10328911

II 10336079 10336221

II 10336289 10336296

II 10336483 10337230

II 10337284 10339307

II 10341700 10341744

II 10360862 10360864

II 10360961 10361047

II 10361190 10361196

II 10361655 10361657

II 10361751 10361770

II 10361866 10361981

II 10362044 10362107

II 10362288 10362328

II 10362472 10362514

II 10362656 10362662

II 10362800 10362841

II 10380421 10380978

II 10381068 10381072

II 10381163 10381443

II 10381495 10381697

II 10381757 10381911

II 10394192 10394426

II 10394530 10394545

II 10394640 10394841

II 10409457 10409477

II 10439807 10439901

II 10439998 10440000

II 10442868 10442962

II 10443058 10443064

II 10465060 10465071

II 10465541 10465550

II 10465709 10465773

II 10465930 10466140

II 10466234 10466264

II 10466452 10466461

II 10477453 10477464

II 10483256 10483328

II 10483477 10483614

II 10493307 10493499

II 10493584 10493964

II 10522492 10522555

II 10522644 10522718

II 10541630 10541669

II 10541719 10541817

II 10557773 10557783

II 10564312 10564365

II 10564460 10564527

II 10565050 10565112

II 10565207 10565264

II 10578524 10578614

II 10578707 10578790

II 10635890 10636037

II 10637157 10637206

II 10637521 10637671

II 10637764 10637766

II 10637857 10637914

II 10776742 10776810

II 10831122 10831251

II 10832529 10832747

II 10832798 10832987

II 10833081 10833216

II 10833353 10833369

II 10833452 10833517

II 10833620 10833685

II 10833769 10833981

II 10839212 10839372

II 10848029 10849643

II 10853460 10855071

II 10886601 10886668

II 10886785 10886848

II 10904525 10905686

II 10911048 10911146

II 10911236 10911308

II 10911651 10911674

II 10911773 10911859

II 10912030 10912040

II 10919950 10920772

II 10920864 10920956

II 10921177 10921390

II 10921488 10921660

II 10921877 10921977

II 10922058 10922887

II 10923067 10923070

II 10932643 10932700

II 10932795 10932862

II 10932976 10933197

II 10941848 10941902

II 10941999 10942081

II 10942179 10942398

II 11050657 11050741

II 11122086 11122400

II 11122607 11122706

II 11122934 11122995

II 11123087 11123128

II 11123236 11123244

II 11123333 11123391

II 11123543 11123592

II 11123688 11124045

II 11124138 11124181

II 11124265 11124372

II 11124500 11124515

II 11137394 11137457

II 11137723 11137790

II 11137843 11137978

II 11138046 11138261

II 11138374 11138382

II 11166090 11166093

II 11169100 11169132

II 11169365 11169408

II 11169494 11169677

II 11169765 11169933

II 11170026 11170042

II 11170109 11170179

II 11170461 11170500

II 11170566 11170667

II 11179592 11179642

II 11190364 11191552

II 11191618 11191979

II 11192118 11192127

II 11197160 11197760

II 11197939 11198590

II 11211330 11211407

II 11211561 11211639

II 11211767 11211778

II 11212004 11212081

II 11212958 11212959

II 11213467 11213485

II 11213587 11213606

II 11214365 11214386

II 11214516 11214518

II 11214614 11215084

II 11215165 11215513

II 11215568 11215892

II 11215953 11215984

II 11216067 11216282

II 11216332 11216534

II 11229570 11229934

II 11230115 11230135

II 11230343 11230388

II 11230458 11230495

II 11230605 11231761

II 11231847 11232010

II 11295736 11295789

II 11296175 11296202

II 11296505 11296534

II 11304485 11304537

II 11377378 11377406

II 11440992 11442519

II 11458366 11458428

II 11505199 11505429

II 11505521 11505538

II 11505794 11505800

II 11505939 11505999

II 11506137 11506146

II 11506233 11506366

II 11506486 11506736

II 11506790 11506832

II 11506915 11506933

II 11507038 11507088

II 11507203 11507209

II 11507395 11507530

II 11507609 11507738

II 11507825 11507876

II 11507983 11508147

II 11508197 11508484

II 11518969 11519206

II 11519298 11519366

II 11527621 11527932

II 11528032 11528124

II 11528187 11529128

II 11529284 11529323

II 11529417 11529457

II 11529595 11530713

II 11532736 11532791

II 11532894 11532898

II 11533000 11533044

II 11534663 11534726

II 11534810 11534947

II 11548938 11548960

II 11549091 11549141

II 11550869 11550878

II 11551737 11551761

II 11552118 11552227

II 11552320 11552352

II 11552588 11552629

II 11552685 11552706

II 11552822 11552826

II 11553036 11553081

II 11553213 11553317

II 11553416 11553500

II 11553574 11553878

II 11554014 11554043

II 11554158 11554159

II 11554426 11554518

II 11554650 11554667

II 11554834 11554845

II 11554942 11555186

II 11558407 11558427

II 11558558 11558609

II 11638386 11638408

II 11679978 11680051

II 11695651 11695853

II 11757191 11759307

II 11759653 11761770

II 11800247 11800335

II 11800401 11800695

II 11800789 11800878

II 11800970 11801522

II 11801842 11801869

II 11801962 11802169

II 11803414 11803448

II 11803540 11803743

II 11803958 11803959

II 11820610 11820617

II 11821510 11821515

II 11824285 11824319

II 11824393 11824497

II 11824575 11824697

II 11824779 11824814

II 11824910 11824928

II 11825081 11825150

II 11825275 11825545

II 11825708 11825721

II 11825813 11825989

II 11826074 11826161

II 11826232 11826249

II 11826361 11826766

II 11826914 11826917

II 11827059 11827280

II 11827354 11827451

II 11832305 11832318

II 11840565 11840661

II 11840755 11840855

II 11875331 11875373

II 11875461 11875467

II 11878236 11878252

II 11878370 11878403

II 11878455 11878463

II 11878698 11878734

II 11879156 11879221

II 11888502 11888539

II 11888992 11889005

II 11906706 11907536

II 11918813 11918838

II 11951380 11951395

II 11955256 11955587

II 11955678 11956338

II 11956407 11956520

II 11956629 11956799

II 11956943 11957262

II 11957335 11957547

II 11957665 11957739

II 11957868 11958084

II 11973351 11973365

II 11984068 11984076

II 11984270 11984282

II 11995743 11995964

II 11996044 11996265

II 11996384 11996402

II 11996491 11996536

II 11996777 11996793

II 12000212 12000298

II 12017020 12017090

II 12031522 12031649

II 12044843 12044988

II 12056844 12056870

II 12056979 12057007

II 12057073 12057087

II 12076263 12076298

II 12078289 12078318

II 12078502 12078524

II 12078642 12078657

II 12082053 12082149

II 12082331 12082341

II 12084849 12084853

II 12091165 12091547

II 12091605 12091610

II 12091709 12091768

II 12091939 12091941

II 12092115 12092142

II 12096583 12096592

II 12096926 12096932

II 12097153 12097161

II 12113101 12114062

II 12114156 12114190

II 12114268 12114311

II 12116715 12116739

II 12119209 12119223

II 12119318 12119352

II 12119403 12119438

II 12120190 12121152

II 12121526 12121637

II 12121745 12121874

II 12121967 12122036

II 12122152 12122248

II 12122347 12122354

II 12122508 12122509

II 12122597 12122607

II 12122729 12122757

II 12122898 12122909

II 12123066 12123107

II 12128242 12128246

II 12129031 12129104

II 12129203 12129214

II 12129369 12129380

II 12133300 12133349

II 12133649 12133731

II 12133859 12133879

II 12134089 12134107

II 12134269 12134296

II 12135173 12135194

II 12135686 12135712

II 12142299 12142304

II 12142532 12142589

II 12148895 12148975

II 12151478 12151599

II 12151956 12151998

II 12152101 12152301

II 12152532 12152627

II 12153988 12154109

II 12154500 12154560

II 12155198 12155201

II 12155296 12155424

II 12156973 12156992

II 12157072 12157273

II 12157492 12157588

II 12158725 12158730

II 12159003 12159026

II 12159120 12159175

II 12162182 12162191

II 12162458 12162480

II 12162576 12162629

II 12163942 12163975

II 12164068 12164171

II 12164307 12164383

II 12164566 12164611

II 12164702 12164775

II 12167254 12167266

II 12168875 12168878

II 12168973 12168980

II 12171392 12171552

II 12171628 12171710

II 12171934 12172202

II 12182030 12182044

II 12182128 12182159

II 12192288 12192329

II 12192430 12192440

II 12194982 12195280

II 12196497 12196581

II 12196638 12196727

II 12200307 12200313

II 12233426 12233465

II 12233577 12233616

II 12238865 12238881

II 12249551 12249577

II 12249666 12249689

II 12249764 12249823

II 12249920 12249928

II 12250344 12250426

II 12263422 12263448

II 12263676 12263684

II 12267970 12268049

II 12268107 12268194

II 12282031 12282105

II 12282317 12282381

II 12283102 12283131

II 12285399 12285556

II 12285641 12286201

II 12286354 12286680

II 12286765 12286924

II 12290779 12290781

II 12307450 12307598

II 12307685 12307695

II 12307968 12308059

II 12308115 12308230

II 12308320 12308337

II 12308421 12308515

II 12310183 12310262

II 12315024 12315043

II 12315098 12315104

II 12315190 12315301

II 12315355 12315414

II 12315464 12315493

II 12315575 12315619

II 12315833 12315878

II 12316032 12316166

II 12316426 12316438

II 12316533 12316575

II 12316651 12316714

II 12316902 12316906

II 12316909 12316922

II 12317178 12317239

II 12317382 12317851

II 12318156 12318294

II 12318357 12318440

II 12318534 12318650

II 12326609 12326737

II 12326825 12326934

II 12353587 12353678

II 12355421 12355448

II 12357725 12357758

II 12359695 12359709

II 12361295 12361324

II 12365107 12365282

II 12395217 12395306

II 12395447 12395640

II 12402941 12403265

II 12403331 12403479

II 12403560 12403606

II 12403673 12403908

II 12403972 12403993

II 12406069 12406070

II 12408396 12408399

II 12426949 12427514

II 12441484 12441545

II 12441824 12442165

II 12442425 12442546

II 12450235 12450265

II 12452736 12452768

II 12520790 12522320

II 12536006 12536145

II 12536226 12536247

II 12536373 12536393

II 12536517 12536636

II 12536960 12536963

II 12537198 12537272

II 12537388 12537392

II 12537488 12537492

II 12537686 12538109

II 12538189 12538261

II 12538356 12538420

II 12538518 12538730

II 12538826 12538829

II 12541965 12541967

II 12545141 12545199

II 12545510 12545551

II 12569585 12570236

II 12582573 12582642

II 12582738 12582771

II 12582945 12583082

II 12583178 12583283

II 12623235 12623385

II 12623757 12623759

II 12623903 12623922

II 12624043 12624172

II 12624446 12624462

II 12624554 12624689

II 12626928 12626948

II 12629461 12629497

II 12649179 12649401

II 12649484 12649502

II 12649583 12649607

II 12649728 12649745

II 12649855 12649908

II 12650039 12650084

II 12650192 12650429

II 12650686 12650711

II 12650955 12651107

II 12651203 12651229

II 12651284 12651386

II 12651455 12651469

II 12651681 12651706

II 12651804 12651921

II 12656608 12656661

II 12656797 12656947

II 12657094 12657114

II 12657199 12657481

II 12663545 12663599

II 12670577 12670619

II 12680931 12680943

II 12700024 12700125

II 12710713 12710786

II 12713211 12713214

II 12713324 12713353

II 12714017 12714050

II 12719393 12719426

II 12719761 12719865

II 12725714 12725718

II 12725919 12725941

II 12726380 12726383

II 12734837 12734908

II 12735247 12735256

II 12740014 12740023

II 12740168 12740191

II 12740463 12740565

II 12741100 12741106

II 12741494 12741597

II 12741748 12741774

II 12745694 12745768

II 12746098 12746112

II 12747657 12747697

II 12747871 12748034

II 12748128 12748297

II 12748566 12749054

II 12749482 12749534

II 12749638 12749683

II 12750367 12750371

II 12750463 12750565

II 12750747 12750764

II 12750898 12750969

II 12751018 12752382

II 12752469 12752503

II 12752611 12752667

II 12753792 12753859

II 12756139 12756202

II 12759773 12759794

II 12775712 12775765

II 12775849 12775921

II 12776398 12776405

II 12777209 12777294

II 12777466 12777723

II 12777840 12777946

II 12778148 12778165

II 12778361 12778404

II 12778885 12778889

II 12779106 12779131

II 12779193 12779245

II 12779672 12779707

II 12779911 12779980

II 12780554 12780574

II 12785739 12785759

II 12786875 12786935

II 12791630 12791639

II 12791760 12791767

II 12806373 12806382

II 12806604 12806607

II 12808146 12808161

II 12810494 12810517

II 12814711 12814735

II 12822275 12822350

II 12822430 12822481

II 12822935 12822975

II 12823245 12823265

II 12829856 12829872

II 12843865 12844844

II 12844951 12845385

II 12845450 12845465

II 12845526 12846013

II 12860408 12860517

II 12860767 12860822

II 12860876 12860945

II 12861097 12861200

II 12872438 12872499

II 12875232 12875240

II 12882084 12882126

II 12892295 12892352

II 12916538 12916579

II 12917934 12917937

II 12926622 12926932

II 12927896 12927943

II 12944846 12944848

II 12946366 12946429

II 12946479 12946510

II 12953916 12954012

II 12974746 12974877

II 12975018 12975244

II 12975296 12975799

II 12975994 12976124

II 13016472 13016531

II 13016657 13016730

II 13020817 13020818

II 13027869 13028029

II 13028175 13028329

II 13031471 13031549

II 13031868 13031943

II 13033633 13033722

II 13036009 13036035

II 13038463 13038474

II 13038794 13038888

II 13039299 13039312

II 13039399 13039417

II 13039512 13039860

II 13040085 13040089

II 13040178 13040384

II 13040520 13040650

II 13040738 13040776

II 13041089 13041119

II 13041277 13041281

II 13041283 13041284

II 13041596 13041603

II 13041775 13041936

II 13042028 13042098

II 13042241 13042251

II 13042565 13042583

II 13042672 13042780

II 13042867 13042904

II 13042994 13043029

II 13043216 13043244

II 13043319 13043368

II 13043464 13043480

II 13043788 13043823

II 13043989 13044010

II 13044177 13044446

II 13044507 13044817

II 13044986 13045029

II 13045161 13045195

II 13045278 13045293

II 13045584 13045631

II 13045796 13045926

II 13046135 13046153

II 13046304 13046409

II 13046465 13046484

II 13046792 13047039

II 13047089 13047118

II 13047209 13047241

II 13047832 13047928

II 13057592 13057595

II 13058222 13058320

II 13058483 13058571

II 13061819 13061861

II 13066423 13066438

II 13079353 13080428

II 13080604 13080756

II 13082418 13082573

II 13082663 13083735

II 13094983 13094989

II 13095242 13095367

II 13095519 13095643

II 13095896 13095905

II 13097307 13097380

II 13097467 13097548

II 13097643 13097644

II 13097708 13097792

II 13120188 13120470

II 13120581 13120664

II 13120738 13120793

II 13125084 13125195

II 13125247 13125461

II 13125581 13125664

II 13125956 13125976

II 13127488 13127507

II 13127597 13127637

II 13127926 13127943

II 13128558 13128566

II 13131710 13131748

II 13134609 13134630

II 13134819 13134858

II 13135227 13135261

II 13135380 13135620

II 13135759 13135972

II 13136065 13136245

II 13136337 13136406

II 13136548 13136590

II 13136658 13136728

II 13136921 13136930

II 13137210 13137228

II 13137566 13137585

II 13137871 13137931

II 13138010 13138098

II 13138212 13138278

II 13138335 13138346

II 13138697 13138751

II 13157800 13157837

II 13161096 13161103

II 13161245 13161315

II 13161398 13161407

II 13182329 13182331

II 13182332 13182334

II 13182514 13182566

II 13182627 13182641

II 13183795 13183844

II 13184559 13184599

II 13188775 13188922

II 13188968 13188994

II 13189084 13189115

II 13191619 13191675

II 13191743 13193028

II 13193140 13193172

II 13202527 13202546

II 13243732 13243900

II 13243950 13244197

II 13244281 13244474

II 13244618 13244637

II 13244983 13245089

II 13251122 13251128

II 13251693 13251725

II 13259302 13259540

II 13259926 13259988

II 13261551 13261607

II 13262599 13262779

II 13263398 13263410

II 13263507 13263609

II 13264163 13264193

II 13264332 13264346

II 13264702 13264759

II 13265327 13265338

II 13284766 13284845

II 13284912 13284983

II 13307640 13307733

II 13307818 13308269

II 13308349 13309038

II 13309128 13309147

II 13312118 13312168

II 13317475 13317854

II 13318374 13318757

II 13319304 13319334

II 13319480 13319775

II 13321594 13321673

II 13321752 13321805

II 13323868 13323951

II 13324787 13324876

II 13334519 13334605

II 13334757 13334841

II 13339077 13339092

II 13339266 13339285

II 13343851 13343968

II 13344062 13344068

II 13344186 13344395

II 13344397 13344398

II 13344532 13344706

II 13348550 13348572

II 13348622 13349286

II 13349451 13349473

II 13352634 13352685

II 13354369 13354508

II 13354611 13355274

II 13355389 13355526

II 13355628 13355659

II 13357322 13357339

II 13358492 13358501

II 13358886 13358894

II 13361100 13361113

II 13361336 13361346

II 13375914 13375981

II 13377162 13377229

II 13377708 13377720

II 13378956 13378965

II 13379913 13380050

II 13381265 13381320

II 13381385 13381563

II 13381659 13381756

II 13381842 13381877

II 13383715 13383833

II 13383902 13384044

II 13384130 13384236

II 13384543 13384578

II 13384660 13384665

II 13384951 13384971

II 13385065 13385142

II 13385524 13385562

II 13386572 13386768

II 13386908 13386943

II 13387112 13387217

II 13392773 13392896

II 13392955 13393092

II 13393177 13393206

II 13393262 13393272

II 13395370 13395429

II 13397911 13397965

II 13398098 13398109

II 13398257 13398310

II 13399537 13399538

II 13406887 13406984

II 13413328 13413338

II 13413463 13413473

II 13421322 13421613

II 13421743 13422016

II 13422066 13422079

II 13422953 13423292

II 13423373 13423713

II 13429592 13429615

II 13429701 13430940

II 13431026 13433228

II 13433290 13433410

II 13441460 13441538

II 13441703 13442074

II 13443843 13443853

II 13444094 13444100

II 13452080 13452173

II 13452358 13452463

II 13452734 13452869

II 13452961 13453010

II 13459309 13459323

II 13460108 13460116

II 13462997 13463588

II 13463677 13464347

II 13474979 13474986

II 13486675 13486749

II 13486870 13487326

II 13487381 13487390

II 13488861 13488867

II 13489253 13489258

II 13493681 13493744

II 13494056 13494073

II 13502957 13503091

II 13503172 13503304

II 13521658 13521692

II 13523846 13524042

II 13524103 13524393

II 13524482 13524670

II 13524754 13524781

II 13524873 13525078

II 13525167 13525363

II 13534737 13534845

II 13534905 13535011

II 13544144 13544164

II 13544727 13544744

II 13544924 13545309

II 13551405 13551439

II 13555944 13555999

II 13557060 13557119

II 13558778 13558791

II 13558922 13558930

II 13559168 13559176

II 13562477 13562491

II 13563709 13563726

II 13579134 13579149

II 13579581 13579596

II 13580171 13580186

II 13583886 13584171

II 13591235 13591247

II 13591363 13591366

II 13594511 13594547

II 13598599 13598633

II 13598731 13599160

II 13599221 13599328

II 13599403 13599591

II 13599670 13599806

II 13599865 13599866

II 13599867 13599871

II 13599961 13599979

II 13600300 13600315

II 13600406 13600425

II 13605352 13605375

II 13605467 13605484

II 13605600 13605682

II 13605798 13606019

II 13606221 13606299

II 13606571 13606580

II 13606672 13606780

II 13606887 13606947

II 13607126 13607135

II 13607262 13607342

II 13607580 13607589

II 13607705 13607748

II 13618425 13618603

II 13619248 13619283

II 13619398 13619473

II 13619622 13619684

II 13619741 13619808

II 13619898 13620015

II 13620198 13620240

II 13620400 13620467

II 13620564 13620594

II 13620679 13620799

II 13620926 13621014

II 13621157 13621398

II 13621571 13621705

II 13621900 13622339

II 13631194 13631267

II 13639318 13639322

II 13639418 13639424

II 13646646 13646685

II 13647700 13647738

II 13647941 13648029

II 13650272 13650457

II 13650660 13651130

II 13651195 13651553

II 13651646 13651832

II 13651909 13651954

II 13652039 13652151

II 13652262 13652379

II 13652460 13652560

II 13654121 13654131

II 13654352 13654412

II 13654786 13654798

II 13654893 13654916

II 13655089 13655175

II 13655228 13655229

II 13655346 13655374

II 13655537 13655601

II 13656117 13656268

II 13656459 13656545

II 13656641 13656648

II 13657074 13657091

II 13657203 13657301

II 13657446 13657914

II 13658000 13658278

II 13658392 13658412

II 13658506 13658563

II 13658922 13658951

II 13659568 13659627

II 13659700 13659784

II 13659880 13659944

II 13660040 13660107

II 13660200 13660211

II 13660305 13660397

II 13660510 13660550

II 13660645 13660792

II 13661774 13661857

II 13662257 13662434

II 13662732 13662742

II 13662964 13663020

II 13668534 13668537

II 13668639 13668701

II 13670050 13670068

II 13670237 13670285

II 13670459 13670489

II 13682771 13682850

II 13683105 13683112

II 13683194 13683212

II 13683297 13683319

II 13683491 13683537

II 13684142 13684152

II 13687023 13687026

II 13687483 13687486

II 13687571 13687576

II 13701747 13701790

II 13701870 13701931

II 13702010 13702071

II 13702153 13702531

II 13702585 13702877

II 13705483 13705489

II 13705607 13705689

II 13717608 13717718

II 13726515 13726533

II 13726912 13726931

II 13727003 13727029

II 13727369 13727386

II 13740959 13740989

II 13741063 13741101

II 13759453 13759455

II 13761372 13761448

II 13761511 13761546

II 13761618 13761748

II 13761832 13761868

II 13761963 13762039

II 13762109 13762253

II 13762349 13762404

II 13762474 13762579

II 13762822 13762842

II 13764174 13764180

II 13769911 13769920

II 13770084 13770113

II 13790930 13791040

II 13800098 13800173

II 13800232 13800653

II 13804543 13804566

II 13804865 13804868

II 13817806 13822201

II 13822286 13824161

II 13824815 13828347

II 13828553 13828650

II 13829330 13829480

II 13835956 13836127

II 13842921 13842946

II 13849275 13849368

II 13850774 13850779

II 13851352 13851368

II 13852783 13852950

II 13853336 13853358

II 13853485 13853501

II 13853620 13853647

II 13853868 13853915

II 13859429 13859642

II 13868767 13868803

II 13869096 13869128

II 13875767 13875769

II 13891599 13891626

II 13891691 13891832

II 13892016 13892053

II 13892144 13892180

II 13892230 13892305

II 13899313 13899321

II 13899579 13899586

II 13904337 13904344

II 13904434 13904444

II 13918443 13918453

II 13923216 13923341

II 13926139 13926174

II 13926252 13926724

II 13926780 13926839

II 13929037 13929169

II 13939983 13940015

II 13940110 13940117

II 13940193 13941422

II 13944398 13944431

II 13944516 13944701

II 13944797 13945157

II 13946322 13946391

II 13946608 13946613

II 13946771 13946775

II 13947111 13947182

II 13947999 13948016

II 13948277 13948640

II 13948731 13948918

II 13949000 13949129

II 13951460 13951578

II 13960248 13960271

II 13960337 13960759

II 13962737 13964150

II 13969933 13970060

II 13985040 13985171

II 13985237 13985432

II 13985611 13985876

II 13985931 13986188

II 13986334 13986539

II 13986587 13986590

II 13986885 13986912

II 13989560 13989565

II 13989789 13989797

II 13990162 13990212

II 13990308 13990432

II 13990649 13990657

II 13990730 13990780

II 13990829 13990852

II 13990920 13990924

II 14000359 14000492

II 14000831 14000844

II 14000850 14000853

II 14000948 14001190

II 14001284 14001314

II 14012075 14012085

II 14021263 14021285

II 14021375 14021395

II 14022909 14022970

II 14023126 14023140

II 14023228 14023251

II 14023732 14023751

II 14024134 14024178

II 14024230 14024305

II 14024513 14024522

II 14024638 14025028

II 14025119 14025249

II 14025446 14025555

II 14025664 14025731

II 14025815 14026027

II 14026113 14026151

II 14026244 14026305

II 14026462 14026476

II 14026651 14026697

II 14026856 14027122

II 14027915 14028213

II 14028305 14028717

II 14028805 14029017

II 14029113 14029157

II 14029243 14029319

II 14029407 14029682

II 14029858 14029972

II 14030060 14030064

II 14047123 14047128

II 14049518 14049574

II 14049664 14049718

II 14059586 14059621

II 14064734 14064770

II 14064966 14065207

II 14066114 14066134

II 14074101 14074441

II 14075780 14075792

II 14076694 14076711

II 14082274 14082299

II 14083910 14083913

II 14084497 14084583

II 14084675 14084721

II 14085756 14085801

II 14087502 14087547

II 14090969 14090984

II 14091868 14091885

II 14092301 14092359

II 14092417 14092460

II 14092529 14092586

II 14099099 14099286

II 14099386 14099573

II 14123497 14123540

II 14135468 14135586

II 14135649 14136040

II 14136136 14136154

II 14139455 14139500

II 14142327 14142526

II 14144330 14144722

II 14144818 14145018

II 14145115 14145135

II 14145239 14145265

II 14145355 14145404

II 14145498 14145506

II 14145648 14145962

II 14146037 14146406

II 14146482 14146568

II 14147270 14147304

II 14158542 14158551

II 14158797 14158805

II 14159824 14159865

II 14159997 14160011

II 14160128 14161654

II 14161784 14161822

II 14163566 14163655

II 14163753 14163874

II 14163936 14163952

II 14164021 14164022

II 14164119 14164152

II 14167146 14167192

II 14167550 14167653

II 14167929 14167940

II 14176137 14176170

II 14176318 14176367

II 14181934 14181968

II 14185750 14185809

II 14204106 14204135

II 14220453 14220487

II 14220683 14220750

II 14222677 14222689

II 14222884 14223292

II 14224320 14224390

II 14224505 14224518

II 14225045 14225115

II 14227065 14227143

II 14227232 14227315

II 14230606 14230673

II 14230769 14230847

II 14258936 14258986

II 14259419 14259434

II 14259582 14259643

II 14259737 14259786

II 14259999 14260014

II 14260118 14260126

II 14260685 14260701

II 14260962 14260963

II 14266610 14266634

II 14267157 14267176

II 14267534 14267542

II 14271246 14271396

II 14275200 14275212

II 14275275 14275404

II 14275529 14275640

II 14277803 14277840

II 14277963 14277985

II 14278245 14278260

II 14278742 14278890

II 14279088 14279091

II 14279166 14279170

II 14282567 14282586

II 14283233 14283254

II 14284770 14284783

II 14284951 14284963

II 14285176 14285203

II 14291330 14291336

II 14291567 14291638

II 14291761 14291884

II 14291968 14291970

II 14292860 14292897

II 14293981 14294199

II 14297343 14297381

II 14300996 14301038

II 14301731 14305903

II 14305966 14310138

II 14312782 14312838

II 14313919 14314095

II 14314427 14314466

II 14314743 14314752

II 14314850 14314950

II 14315504 14315534

II 14317454 14317489

II 14321941 14321948

II 14322063 14322092

II 14322483 14322582

II 14322742 14322836

II 14323964 14325142

II 14325236 14326239

II 14326324 14326688

II 14326782 14330073

II 14330123 14330669

II 14330750 14331821

II 14331931 14331942

II 14332302 14332357

II 14332924 14332935

II 14333510 14333860

II 14333935 14334201

II 14334260 14334667

II 14334756 14336820

II 14336894 14337003

II 14337083 14337111

II 14337203 14337614

II 14337732 14337774

II 14337907 14337981

II 14338152 14338251

II 14338455 14338485

II 14338577 14338903

II 14339153 14339190

II 14340869 14340979

II 14341643 14341699

II 14341772 14341849

II 14342498 14342523

II 14343270 14343476

II 14343611 14343614

II 14344008 14344112

II 14344615 14344900

II 14345024 14345035

II 14345170 14345197

II 14346306 14346340

II 14346408 14346562

II 14369035 14369107

II 14369194 14369424

II 14369511 14369616

II 14369748 14369801

II 14369919 14369951

II 14370047 14370289

II 14370389 14370427

II 14370518 14370601

II 14370692 14371173

II 14371269 14371448

II 14371783 14372022

II 14372115 14372353

II 14372438 14372503

II 14414370 14414517

II 14414829 14414867

II 14415022 14415041

II 14415133 14415264

II 14482221 14482262

II 14486453 14486467

II 14499540 14499566

II 14499659 14499857

II 14499969 14500172

II 14504784 14504811

II 14520093 14520130

II 14520833 14520859

II 14538860 14538980

II 14539069 14539184

II 14539279 14539477

II 14539624 14539671

II 14539829 14539915

II 14561867 14561913

II 14561976 14561977

II 14562063 14562233

II 14562553 14562620

II 14562725 14562736

II 14562813 14562908

II 14563132 14563198

II 14563314 14563364

II 14563546 14563617

II 14563785 14563839

II 14563939 14563972

II 14578404 14578465

II 14588111 14588147

II 14635853 14636001

II 14641375 14641524

II 14687870 14688218

II 14688288 14691093

II 14691185 14693132

II 14693220 14693402

II 14711205 14718179

II 14718273 14725253

II 14726555 14726561

II 14726667 14726860

II 14736738 14736897

II 14736951 14737010

II 14741474 14741589

II 14741672 14741716

II 14741809 14742001

II 14742075 14742518

II 14742574 14743100

II 14759531 14759604

II 14759702 14759725

II 14761111 14761244

II 14761319 14761600

II 14761672 14761736

II 14761936 14761938

II 14762026 14762250

II 14762409 14762592

II 14762688 14762782

II 14763012 14763563

II 14763764 14763833

II 14763919 14763969

II 14764060 14764403

II 14764555 14764565

II 14764624 14764669

II 14764759 14764769

II 14765051 14765480

II 14765554 14765681

II 14765943 14766069

II 14766172 14766519

II 14766602 14766773

II 14766932 14767317

II 14767542 14767662

II 14767781 14767802

II 14773407 14773489

II 14793859 14793866

II 14794912 14794913

II 14801581 14801715

II 14801955 14801984

II 14802106 14802107

II 14802250 14802261

II 14802608 14802680

II 14803056 14803132

II 14815563 14815588

II 14815683 14815694

II 14835671 14835685

II 14835798 14835921

II 14835990 14836533

II 14836599 14836753

II 14836800 14836902

II 14836965 14837087

II 14837187 14837263

II 14837342 14837857

II 14837947 14838003

II 14838093 14838289

II 14863827 14864090

II 14864176 14864469

II 14864569 14864584

II 14864676 14864943

II 14865026 14865321

II 14865384 14865403

II 14872151 14872162

II 14872216 14872239

II 14874053 14874057

II 14876281 14876315

II 14876684 14876730

II 14877688 14878029

II 14878084 14878109

II 14878244 14878541

II 14878621 14878643

II 14878699 14879541

II 14879631 14879635

II 14879769 14879773

II 14912948 14912951

II 14913045 14913132

II 14913233 14913246

II 14913337 14913426

II 14917906 14917922

II 14967982 14967989

II 14977137 14977578

II 14977695 14977710

II 14977799 14978900

II 14978957 14978969

II 14979124 14979141

II 14979255 14979325

II 14979468 14979749

II 14979929 14979988

II 14980083 14980222

II 14985182 14985183

II 14987782 14988224

II 14988338 14988353

II 14989223 14989232

II 14989342 14989392

II 14989736 14989773

II 14989904 14989940

II 14990871 14990878

II 14991570 14991785

II 14991853 14991920

II 14991978 14993276

II 14993366 14993388

II 14993491 14994280

II 14994380 14994694

II 15000044 15000119

II 15000208 15000334

II 15000418 15000532

II 15000630 15000651

II 15001078 15001158

II 15006675 15006701

II 15006751 15006781

II 15011412 15011425

II 15011830 15011924

II 15012019 15012129

II 15024835 15024836

II 15024838 15024844

II 15031269 15031318

II 15031439 15031458

II 15032351 15032400

II 15032519 15032535

II 15033330 15033376

II 15041072 15041086

II 15041337 15041438

II 15042849 15042934

II 15043943 15044030

II 15045971 15046070

II 15046287 15046307

II 15046405 15046502

II 15046753 15046777

II 15046865 15047039

II 15049274 15049355

II 15049703 15049818

II 15049920 15049956

II 15050025 15050129

II 15050186 15050215

II 15050307 15050682

II 15050821 15050923

II 15052909 15053026

II 15053110 15053377

II 15053455 15054636

II 15054731 15054958

II 15055072 15055131

II 15055272 15055371

II 15055459 15055668

II 15059837 15060044

II 15060129 15060224

II 15060370 15060429

II 15060544 15060772

II 15060866 15062395

II 15062446 15062477

II 15065773 15065774

II 15065919 15065954

II 15067405 15067440

II 15067585 15067588

II 15067682 15067684

II 15069298 15069434

II 15072950 15072951

II 15073044 15073066

II 15074252 15074280

II 15080117 15080122

II 15082319 15082342

II 15082436 15082467

II 15082552 15082618

II 15095443 15095663

II 15095793 15095836

II 15095933 15095967

II 15096063 15096421

II 15096586 15096773

II 15096830 15096915

II 15105926 15106005

II 15106082 15106159

II 15106444 15106488

II 15106575 15106626

II 15106722 15107096

II 15111215 15111240

II 15111325 15111341

II 15112780 15112801

II 15114615 15114736

II 15114965 15115064

II 15116503 15116580

II 15116695 15117050

II 15120973 15120978

II 15123664 15123671

II 15123787 15123796

II 15123939 15123941

II 15124089 15124099

II 15124817 15124861

II 15126550 15126560

II 15126692 15126693

II 15126934 15126962

II 15127120 15127155

II 15127290 15127302

II 15127390 15127428

II 15131577 15131581

II 15131956 15131988

II 15132120 15132157

II 15137616 15137733

II 15137824 15138489

II 15138583 15138866

II 15138941 15139368

II 15139446 15139784

II 15139880 15139897

II 15139982 15140061

II 15140117 15140384

II 15140478 15142015

II 15142134 15142467

II 15142526 15142750

II 15142847 15142855

II 15156124 15156137

II 15156542 15156556

II 15167754 15167896

II 15167991 15168231

II 15170715 15170726

II 15172012 15173177

II 15179432 15179433

II 15179522 15179557

II 15179681 15179824

II 15180822 15180825

II 15181659 15182259

II 15182335 15182934

II 15184872 15184976

II 15186221 15186222

II 15186377 15186750

II 15186850 15187260

II 15187352 15187373

II 15188707 15188717

II 15188784 15188785

II 15189047 15189063

II 15189180 15189206

II 15191609 15191917

II 15192009 15192091

II 15194792 15194816

II 15199414 15199484

II 15199545 15199612

II 15199985 15200012

II 15200124 15200279

II 15209787 15209795

II 15210076 15210147

II 15210816 15210817

II 15210904 15210949

II 15211046 15211090

II 15213390 15213399

II 15213516 15213526

II 15214723 15214754

II 15215020 15215049

II 15215199 15215234

II 15222211 15222227

II 15222339 15222342

II 15223058 15223083

II 15225142 15225205

II 15225448 15225510

II 15225568 15225569

II 15225750 15225772

II 15230677 15230701

II 15233777 15233789

II 15235059 15235112

II 15235298 15235350

II 15245402 15245513

II 15245605 15245631

II 15245711 15245817

II 15245914 15245940

II 15249646 15249729

II 15249823 15249869

II 15249960 15250011

II 15250377 15250457

II 15274757 15275068

II 15275161 15276451

II 15276881 15276892

II 15277126 15278415

II 15278506 15278819

II 15278941 15279111

II 15279274 15279345

II 15279417 15279421

III 0 88

III 54180 54188

III 54284 54375

III 54432 54619

III 54743 54792

III 54902 54907

III 54981 55089

III 55241 55264

III 55410 55445

III 55511 55628

III 55801 55815

III 56270 56297

III 56605 56641

III 56780 56818

III 56939 56979

III 57097 57125

III 57555 57556

III 57944 57982

III 58801 58886

III 59040 59047

III 59117 59118

III 59205 59266

III 78645 79300

III 79390 79439

III 83614 83759

III 83909 83925

III 85484 85509

III 85562 85569

III 97021 98124

III 98219 99325

III 99485 99545

III 99810 99842

III 99985 100220

III 100340 100858

III 100926 101394

III 101487 102018

III 102096 103703

III 103792 104008

III 104101 104132

III 104224 104248

III 104308 104579

III 104642 104963

III 105013 105194

III 105275 105569

III 105707 106388

III 106481 107160

III 108063 108075

III 108208 108220

III 108357 108364

III 110321 110331

III 110419 111116

III 111275 111383

III 111498 111505

III 111609 111654

III 111809 111924

III 112078 112160

III 112211 112240

III 112331 112337

III 112630 112640

III 112776 112817

III 112890 112913

III 112967 112995

III 113088 113145

III 113301 113314

III 113482 113484

III 113539 113579

III 113674 113812

III 121344 121374

III 121470 121485

III 121724 121735

III 121947 121965

III 122256 122334

III 128819 128849

III 129030 129196

III 129286 129421

III 133675 133683

III 133742 133751

III 134743 135013

III 135104 135211

III 135296 135298

III 135533 135540

III 135660 135705

III 135768 135814

III 142392 142411

III 145771 145788

III 146789 146888

III 155449 155521

III 155606 155696

III 160930 161204

III 161295 161879

III 162831 162875

III 168464 168520

III 168686 168732

III 168815 168859

III 179109 179166

III 185978 186088

III 193372 193436

III 200370 200422

III 200517 200600

III 200739 200979

III 201063 201076

III 210904 210960

III 211169 211225

III 211738 211750

III 211856 211909

III 215018 215048

III 215105 215327

III 216926 216968

III 217155 217190

III 232991 233045

III 233136 233404

III 233642 233831

III 234001 234121

III 234209 234259

III 234351 234408

III 234489 234509

III 248703 248749

III 260003 260036

III 260278 260325

III 262336 262375

III 262431 262433

III 262526 262624

III 262712 262815

III 264708 264734

III 267215 267227

III 267349 267470

III 267550 267669

III 267762 268030

III 268118 268306

III 269809 269847

III 270002 270023

III 271279 271360

III 272649 272681

III 273364 273401

III 273478 273782

III 273876 276502

III 277387 277392

III 280175 280182

III 280917 281050

III 281146 281270

III 282037 282039

III 283055 283159

III 283293 283398

III 285677 285690

III 287080 287094

III 288687 288785

III 288863 288958

III 289968 290510

III 290605 290639

III 290691 292219

III 297027 297030

III 301548 301976

III 302184 302341

III 302424 302652

III 302756 302803

III 302908 302981

III 303030 303048

III 303601 303607

III 304111 304150

III 304243 304272

III 305053 305063

III 305381 305437

III 311933 311963

III 312606 312631

III 316103 316398

III 316576 316619

III 316695 316721

III 316812 316819

III 317744 318486

III 318550 319324

III 319471 319613

III 320567 320752

III 320846 320879

III 321042 321227

III 321827 324187

III 324271 324449

III 324507 324590

III 324679 324823

III 324879 326350

III 326411 328173

III 328261 329141

III 329194 329377

III 329435 330088

III 330181 330569

III 330637 332185

III 332273 332314

III 332374 332836

III 332918 333072

III 337134 337144

III 337333 337338

III 337395 337399

III 337554 337564

III 338859 338867

III 339315 339387

III 339457 339526

III 340789 340935

III 341041 341060

III 341196 341213

III 345254 345288

III 360935 360952

III 374185 374210

III 374532 374553

III 374634 374721

III 374949 374999

III 375091 375102

III 375339 375890

III 380143 380184

III 380273 380370

III 380448 380454

III 384049 384054

III 388370 388427

III 388518 388550

III 388605 388670

III 388785 388842

III 388936 389140

III 389195 389705

III 389783 390928

III 391012 391021

III 391081 391136

III 391248 391417

III 391581 391814

III 391961 392035

III 392234 392381

III 392438 392452

III 392536 392701

III 392782 393119

III 393256 393960

III 394055 396457

III 396651 396781

III 396870 397053

III 397161 397226

III 397308 397375

III 397465 397610

III 403197 403198

III 406174 406247

III 406345 406350

III 406420 406424

III 406490 406504

III 406970 407005

III 407090 407134

III 407197 407209

III 407334 407339

III 407429 407471

III 407604 407634

III 413121 413124

III 413248 413433

III 413503 413519

III 413570 413665

III 414414 415320

III 415546 415636

III 415754 416253

III 416327 416560

III 422471 422503

III 424384 424911

III 425080 425150

III 425277 425355

III 425466 425512

III 425628 425639

III 425728 425734

III 425803 425811

III 425887 426102

III 426157 426228

III 426314 426523

III 426575 426641

III 426731 426958

III 427009 427149

III 427200 427605

III 427698 428004

III 445695 445729

III 446398 446780

III 449883 449903

III 450043 450278

III 450344 450463

III 450559 450577

III 450670 450766

III 450826 452075

III 452198 452240

III 452306 452443

III 452557 452571

III 452768 452794

III 452867 452909

III 453031 453112

III 453286 453346

III 462910 462923

III 464682 464695

III 467410 467736

III 478773 478883

III 479131 479234

III 479289 479317

III 479390 479409

III 483461 483502

III 487837 487863

III 487980 488018

III 488246 488285

III 490746 490752

III 492702 492757

III 492884 492908

III 493142 493190

III 493268 493339

III 498796 498823

III 498980 499003

III 501383 501540

III 503198 504229

III 527783 527918

III 528121 528133

III 528502 528550

III 533248 533262

III 535424 535426

III 536414 536477

III 538872 538954

III 540671 540754

III 554095 554135

III 564642 564703

III 566189 566193

III 566277 566419

III 566503 566585

III 570123 570134

III 570429 570452

III 578438 578462

III 578648 578691

III 581112 581181

III 581339 581381

III 581489 581527

III 583124 583248

III 583325 583541

III 583591 583711

III 583807 583830

III 584931 585031

III 585084 585191

III 588035 588126

III 588468 589051

III 589147 590917

III 592447 592465

III 592654 592706

III 592919 592954

III 594308 594315

III 625417 625508

III 628913 628921

III 629221 629256

III 629311 629352

III 629446 629658

III 629748 629876

III 630007 630299

III 631669 631683

III 687613 687733

III 688942 689500

III 689602 689764

III 700484 700508

III 700608 700627

III 700841 700902

III 701029 701031

III 701125 701206

III 701630 701661

III 701863 701890

III 701971 702036

III 702271 702524

III 702618 702998

III 703173 703189

III 703284 703340

III 703388 703772

III 703865 704120

III 704208 704285

III 704353 704419

III 704515 704517

III 704518 704520

III 704601 704647

III 704734 704796

III 704946 704947

III 704949 704952

III 705010 705028

III 705151 705154

III 716757 716764

III 742479 742480

III 750712 750715

III 750871 750876

III 753073 753231

III 782033 782040

III 782091 782101

III 786992 787099

III 810806 810841

III 810930 810966

III 817967 817979

III 819797 820007

III 827158 827315

III 827422 827983

III 831170 831300

III 850703 850752

III 858632 858642

III 863773 863844

III 864246 864331

III 864414 864424

III 864538 864595

III 886015 886025

III 910746 910803

III 912978 913087

III 916076 916114

III 916202 916234

III 931294 931382

III 931449 931474

III 931771 931782

III 932067 932068

III 932631 932675

III 934515 935676

III 935846 935855

III 940596 940612

III 940665 940754

III 943916 943960

III 944659 944741

III 946979 947007

III 949627 949647

III 958735 958784

III 959236 959246

III 959512 959584

III 975126 975143

III 975220 975513

III 975602 975648

III 977976 978012

III 978747 978876

III 986398 986424

III 986776 986802

III 1006369 1006397

III 1009278 1009304

III 1011652 1011768

III 1011999 1012031

III 1017408 1017597

III 1017647 1018036

III 1018111 1019643

III 1019727 1020065

III 1020143 1020586

III 1022162 1022188

III 1022274 1022294

III 1022464 1022858

III 1029078 1029539

III 1035270 1035279

III 1035367 1035506

III 1039566 1039703

III 1039793 1039798

III 1065978 1066009

III 1066784 1067330

III 1068377 1068395

III 1068619 1068632

III 1073055 1073696

III 1073868 1073873

III 1073965 1074078

III 1076669 1076996

III 1087451 1087466

III 1087693 1087712

III 1088726 1090983

III 1095528 1095560

III 1095727 1096014

III 1096107 1096183

III 1096266 1096389

III 1118192 1118420

III 1118690 1118740

III 1118832 1118857

III 1119066 1119082

III 1119378 1119385

III 1119769 1119817

III 1119952 1120020

III 1120098 1120241

III 1120365 1120398

III 1120671 1120675

III 1120901 1120918

III 1121042 1121067

III 1121159 1121306

III 1121399 1121705

III 1121796 1122157

III 1122934 1122952

III 1123040 1124091

III 1145913 1145925

III 1146475 1146610

III 1147488 1147499

III 1148456 1148675

III 1148815 1149032

III 1150221 1150247

III 1174564 1174618

III 1174708 1174739

III 1192613 1192636

III 1192961 1192987

III 1198791 1198815

III 1211687 1211699

III 1239854 1239856

III 1245636 1245792

III 1245856 1246086

III 1246136 1246233

III 1246428 1246579

III 1246652 1246772

III 1250284 1251168

III 1251263 1252938

III 1253026 1253105

III 1253194 1254956

III 1255051 1257330

III 1257426 1258545

III 1258645 1259526

III 1259622 1261177

III 1261233 1261250

III 1261307 1261381

III 1261465 1261571

III 1261636 1263398

III 1263493 1265772

III 1265868 1266986

III 1270846 1270857

III 1283526 1283550

III 1283905 1283910

III 1285877 1285894

III 1293429 1293437

III 1293498 1294582

III 1299793 1299796

III 1299847 1300063

III 1300122 1300139

III 1300200 1300272

III 1300343 1301113

III 1301204 1301330

III 1301418 1301681

III 1301767 1302024

III 1302115 1303108

III 1304739 1304744

III 1314919 1314968

III 1317056 1317112

III 1319569 1319642

III 1320807 1320812

III 1322708 1322779

III 1324848 1325859

III 1325966 1326014

III 1326262 1326321

III 1326364 1326472

III 1326534 1326537

III 1337757 1337759

III 1353547 1353549

III 1353660 1353675

III 1353993 1353999

III 1356222 1356281

III 1356341 1356418

III 1356564 1356700

III 1356823 1356898

III 1356995 1357021

III 1358145 1358190

III 1358283 1358304

III 1358465 1358714

III 1358802 1358849

III 1358944 1359304

III 1359430 1359476

III 1362837 1363777

III 1363875 1364931

III 1364979 1365898

III 1365979 1366599

III 1366655 1366912

III 1366974 1367922

III 1368034 1368068

III 1368131 1368214

III 1368377 1368423

III 1368473 1368540

III 1368619 1368747

III 1368801 1368826

III 1368910 1370050

III 1375937 1375952

III 1394159 1394219

III 1394639 1394700

III 1399152 1399206

III 1403700 1403718

III 1403918 1403931

III 1407149 1407733

III 1407898 1408477

III 1414852 1415085

III 1415257 1415495

III 1417736 1417912

III 1418009 1418123

III 1418324 1418354

III 1418628 1418658

III 1420649 1420717

III 1421958 1421983

III 1422032 1422232

III 1422676 1422937

III 1423026 1423285

III 1430563 1430564

III 1432861 1432862

III 1441706 1441712

III 1443911 1443973

III 1444069 1444119

III 1444201 1444226

III 1444378 1444515

III 1444678 1444722

III 1444922 1445798

III 1445886 1445917

III 1447144 1447145

III 1447148 1447149

III 1463795 1463811

III 1467656 1467691

III 1469348 1469367

III 1472468 1472505

III 1473197 1473259

III 1476574 1476598

III 1480380 1481439

III 1481525 1481668

III 1481822 1483237

III 1487932 1487949

III 1488144 1488434

III 1488549 1488650

III 1488744 1488798

III 1490174 1490180

III 1490660 1490661

III 1491886 1491898

III 1494320 1494356

III 1497133 1497199

III 1497291 1497389

III 1497504 1497796

III 1497950 1498004

III 1501251 1501298

III 1502083 1502115

III 1503289 1503331

III 1505096 1505132

III 1507601 1507621

III 1511338 1511387

III 1513260 1513267

III 1522597 1522663

III 1526920 1526930

III 1529859 1529870

III 1530678 1530726

III 1531737 1531793

III 1535099 1535104

III 1536179 1536419

III 1541555 1541583

III 1544963 1544971

III 1545112 1545176

III 1547481 1547496

III 1547600 1547623

III 1548333 1548359

III 1550594 1550665

III 1550800 1550866

III 1551415 1551422

III 1552288 1552295

III 1553402 1553429

III 1556317 1556331

III 1556946 1556955

III 1560828 1560837

III 1560994 1561005

III 1562131 1562170

III 1566302 1566345

III 1566350 1566365

III 1566640 1566783

III 1566851 1566889

III 1566943 1566947

III 1567099 1567332

III 1567451 1567471

III 1568017 1568071

III 1568213 1568279

III 1568341 1568365

III 1568455 1568537

III 1568932 1568949

III 1569634 1569688

III 1574141 1574150

III 1579421 1579441

III 1579512 1579520

III 1579684 1579888

III 1579966 1579988

III 1580074 1580180

III 1581845 1582221

III 1582316 1583036

III 1608439 1608686

III 1608835 1608987

III 1609036 1609146

III 1609195 1609211

III 1615199 1615209

III 1615265 1615324

III 1615387 1615421

III 1615471 1615624

III 1615719 1615727

III 1631209 1631217

III 1633714 1633719

III 1634290 1634299

III 1639866 1639969

III 1640607 1640662

III 1641012 1641019

III 1641158 1641179

III 1641886 1641892

III 1643072 1643138

III 1643195 1643414

III 1645295 1645296

III 1645841 1645951

III 1646005 1646109

III 1646185 1646716

III 1648109 1648110

III 1648112 1648114

III 1650988 1651066

III 1651244 1651274

III 1651574 1651652

III 1656524 1656578

III 1656639 1657041

III 1663664 1663698

III 1671490 1671532

III 1689326 1689388

III 1689462 1689527

III 1690678 1690969

III 1695476 1695509

III 1695607 1695656

III 1695747 1695770

III 1696139 1696147

III 1696257 1696276

III 1699337 1699384

III 1699491 1699579

III 1699871 1699874

III 1700007 1700030

III 1700126 1700127

III 1700220 1700552

III 1700620 1701100

III 1701158 1701176

III 1701270 1701473

III 1701528 1701598

III 1703422 1703445

III 1703552 1703567

III 1707236 1707291

III 1707392 1707502

III 1707600 1707844

III 1708020 1708064

III 1708299 1708547

III 1710767 1710799

III 1715773 1715788

III 1715997 1716008

III 1726989 1727115

III 1727457 1727463

III 1727680 1727705

III 1729522 1730074

III 1730152 1730710

III 1757424 1757434

III 1757575 1757693

III 1760450 1760566

III 1760708 1760714

III 1766165 1766177

III 1766548 1766559

III 1766691 1766760

III 1771115 1771227

III 1776258 1776282

III 1778897 1778993

III 1782094 1782188

III 1784164 1784168

III 1785254 1785375

III 1785579 1785605

III 1807531 1807652

III 1810064 1810071

III 1810168 1810169

III 1817553 1817679

III 1817766 1817821

III 1817878 1817908

III 1818145 1818195

III 1820067 1820204

III 1820382 1820520

III 1820812 1820834

III 1825770 1825792

III 1825997 1826005

III 1826234 1826315

III 1826373 1826441

III 1826530 1826553

III 1828454 1828459

III 1828556 1828588

III 1828640 1828747

III 1828836 1828947

III 1829206 1829261

III 1829396 1829402

III 1829588 1829621

III 1831514 1831522

III 1831664 1831682

III 1833057 1833068

III 1837752 1837805

III 1837890 1837988

III 1839319 1839375

III 1842383 1842427

III 1843890 1843952

III 1845508 1845646

III 1845701 1846209

III 1847462 1847506

III 1848316 1848371

III 1851728 1851749

III 1851842 1851894

III 1851998 1852004

III 1853073 1853076

III 1853181 1853347

III 1862493 1862564

III 1862681 1862716

III 1863308 1863321

III 1863716 1863766

III 1863913 1863967

III 1867966 1868036

III 1868135 1868160

III 1870021 1870040

III 1870133 1870156

III 1872805 1872810

III 1873029 1873036

III 1873262 1873342

III 1873474 1873485

III 1874173 1874192

III 1874292 1874320

III 1874370 1874389

III 1875619 1875831

III 1881015 1881094

III 1892446 1892532

III 1892637 1893005

III 1903374 1903413

III 1917959 1917987

III 1918049 1918078

III 1933722 1933775

III 1934683 1934916

III 1934972 1935216

III 1935337 1935367

III 1935429 1935452

III 1935654 1935696

III 1935968 1935970

III 1942313 1942363

III 1942531 1942538

III 1944359 1944367

III 1944787 1944835

III 1945558 1945613

III 1947160 1947178

III 1948448 1948466

III 1958212 1958265

III 1965473 1965495

III 1965852 1965904

III 1985925 1985992

III 2009723 2009804

III 2009888 2009924

III 2010003 2010084

III 2010136 2010170

III 2011883 2011906

III 2012194 2012214

III 2014188 2014262

III 2022282 2022324

III 2023550 2023613

III 2023697 2023759

III 2028386 2028391

III 2028823 2028827

III 2055935 2055936

III 2056301 2056323

III 2056414 2056422

III 2056652 2056664

III 2063967 2064627

III 2064690 2064699

III 2065269 2065423

III 2067169 2067178

III 2072491 2072526

III 2072725 2072775

III 2072866 2073295

III 2074430 2074466

III 2074664 2074711

III 2074808 2075235

III 2077672 2079822

III 2079915 2080267

III 2080362 2082401

III 2082496 2082600

III 2113459 2113477

III 2113572 2113728

III 2113807 2113999

III 2114056 2114061

III 2114211 2114256

III 2114311 2114330

III 2114404 2114437

III 2119248 2120766

III 2121037 2121050

III 2121431 2121603

III 2122843 2122849

III 2123408 2123416

III 2125554 2125555

III 2135491 2135541

III 2135608 2135673

III 2135994 2136007

III 2159609 2159829

III 2178177 2178230

III 2180315 2180406

III 2186716 2186770

III 2186954 2187003

III 2187738 2187840

III 2189934 2190013

III 2190089 2190167

III 2195815 2195838

III 2203686 2203687

III 2203845 2203846

III 2205638 2205666

III 2207671 2207700

III 2208583 2208733

III 2208809 2209013

III 2209427 2209496

III 2209603 2209638

III 2214832 2215327

III 2215406 2215417

III 2215479 2215717

III 2215780 2216202

III 2216282 2216515

III 2218573 2219043

III 2219130 2219179

III 2223580 2223589

III 2223705 2223733

III 2224399 2224406

III 2226348 2226377

III 2237674 2237684

III 2261621 2261711

III 2261903 2261915

III 2262069 2262071

III 2262072 2262073

III 2268421 2268495

III 2271217 2271349

III 2271445 2271509

III 2271728 2271855

III 2274675 2274689

III 2277869 2277986

III 2278081 2278185

III 2278426 2278509

III 2290139 2290372

III 2290439 2290664

III 2297231 2297257

III 2297548 2297661

III 2300796 2300854

III 2300974 2300979

III 2312213 2312635

III 2312787 2312806

III 2313105 2313125

III 2313288 2313713

III 2322813 2322822

III 2324140 2324147

III 2338962 2339015

III 2339374 2339424

III 2340700 2340787

III 2340886 2341121

III 2344872 2344883

III 2345000 2345258

III 2349505 2349768

III 2351776 2351810

III 2354189 2354201

III 2354255 2354269

III 2354332 2354498

III 2354727 2354741

III 2355199 2355223

III 2357797 2357813

III 2358008 2358126

III 2358298 2358312

III 2358441 2358455

III 2362611 2362617

III 2364582 2364609

III 2365229 2365263

III 2365726 2365731

III 2373589 2373695

III 2373776 2373881

III 2377939 2377955

III 2378467 2378539

III 2378607 2378774

III 2379006 2379051

III 2383364 2383380

III 2394637 2394683

III 2396171 2396271

III 2396352 2396409

III 2396489 2396584

III 2398702 2398720

III 2398830 2398855

III 2398935 2398959

III 2399338 2399361

III 2412881 2412895

III 2413689 2413709

III 2414010 2414176

III 2414282 2414404

III 2414585 2414600

III 2414774 2414791

III 2414887 2414892

III 2415531 2415545

III 2415664 2415681

III 2415777 2415979

III 2416168 2416176

III 2416475 2416542

III 2416616 2416658

III 2429272 2429328

III 2429473 2429474

III 2432044 2432249

III 2432339 2432365

III 2433458 2435377

III 2435464 2435510

III 2435823 2435870

III 2435951 2437871

III 2454667 2454670

III 2455503 2455585

III 2455740 2455818

III 2457822 2457830

III 2458339 2458392

III 2458617 2458685

III 2459086 2459119

III 2459192 2459269

III 2459361 2459529

III 2459689 2459704

III 2459822 2459877

III 2459960 2460030

III 2460122 2460140

III 2460303 2460442

III 2460533 2460562

III 2460729 2460780

III 2460877 2460898

III 2461363 2461377

III 2461599 2461674

III 2461960 2462052

III 2462155 2462162

III 2462242 2462271

III 2462365 2462872

III 2463064 2463080

III 2463446 2463465

III 2463546 2463567

III 2464059 2464127

III 2464670 2464701

III 2466029 2466034

III 2466129 2466234

III 2467643 2467675

III 2469718 2469800

III 2470770 2470932

III 2471021 2471209

III 2471435 2471447

III 2471710 2471736

III 2494824 2494897

III 2494996 2495197

III 2495250 2495319

III 2495402 2495660

III 2495755 2496093

III 2496436 2496729

III 2497072 2498458

III 2498548 2498668

III 2498759 2499450

III 2499501 2500093

III 2500255 2501694

III 2501783 2501929

III 2501930 2501933

III 2502338 2502384

III 2502458 2502593

III 2502659 2502930

III 2503054 2503068

III 2503116 2503230

III 2503659 2503677

III 2511217 2511227

III 2521441 2521539

III 2521612 2521774

III 2521848 2522152

III 2563903 2564192

III 2564470 2564557

III 2564673 2564969

III 2565065 2565145

III 2583523 2583599

III 2583780 2583947

III 2584041 2584178

III 2584336 2584469

III 2601280 2601478

III 2601554 2601756

III 2604139 2604157

III 2604282 2604304

III 2607678 2607684

III 2607826 2607829

III 2607919 2607938

III 2608060 2608081

III 2611653 2611760

III 2611854 2611947

III 2612041 2612068

III 2612161 2612202

III 2612465 2612592

III 2612648 2612817

III 2633995 2634031

III 2661527 2661774

III 2661854 2662265

III 2664074 2664075

III 2664230 2664236

III 2664452 2664459

III 2696186 2696193

III 2696243 2696278

III 2696342 2696705

III 2696788 2696798

III 2696863 2696874

III 2699104 2699261

III 2699407 2699465

III 2699823 2699825

III 2699912 2699923

III 2700056 2700090

III 2703418 2703496

III 2709834 2709878

III 2717404 2717494

III 2722680 2723258

III 2723324 2723384

III 2729658 2729667

III 2729762 2729935

III 2730051 2730086

III 2730176 2730333

III 2736818 2736838

III 2740288 2740367

III 2740455 2740492

III 2740643 2740863

III 2740936 2740983

III 2741079 2741084

III 2741175 2741197

III 2741328 2741440

III 2741585 2741660

III 2741732 2741998

III 2742142 2742260

III 2742528 2742756

III 2742805 2742864

III 2743249 2743388

III 2759946 2759979

III 2761597 2761652

III 2761743 2761782

III 2761829 2761892

III 2761976 2761987

III 2762088 2762265

III 2762394 2762496

III 2762707 2762735

III 2762827 2762899

III 2763175 2763189

III 2763280 2763339

III 2763450 2763486

III 2775557 2775572

III 2775879 2775916

III 2776237 2776442

III 2783073 2783142

III 2783358 2783429

III 2788901 2788944

III 2789037 2789209

III 2789299 2789458

III 2789513 2789640

III 2789734 2789749

III 2789847 2789950

III 2790011 2790032

III 2790113 2790289

III 2796041 2796113

III 2796277 2796285

III 2796359 2796442

III 2797100 2797155

III 2797296 2797453

III 2800031 2800035

III 2800117 2800119

III 2803490 2803503

III 2803598 2803603

III 2803842 2803843

III 2803933 2803947

III 2804105 2804144

III 2817001 2817049

III 2817117 2817136

III 2817223 2817343

III 2817406 2817415

III 2817509 2817574

III 2817658 2817700

III 2818499 2818566

III 2818776 2818856

III 2818912 2818926

III 2819652 2819667

III 2820724 2820764

III 2820817 2820915

III 2821008 2821023

III 2821082 2821308

III 2828118 2828161

III 2828220 2828230

III 2828598 2828627

III 2837321 2837349

III 2837417 2837444

III 2839235 2839238

III 2839333 2839340

III 2841413 2841469

III 2845692 2845946

III 2845995 2846015

III 2846430 2846538

III 2846719 2846831

III 2856282 2856539

III 2857055 2857070

III 2857160 2857240

III 2857328 2857432

III 2857505 2857558

III 2857648 2857700

III 2857955 2858031

III 2859194 2859201

III 2859566 2859584

III 2859761 2859796

III 2859890 2859954

III 2860172 2860207

III 2868276 2868294

III 2881873 2881881

III 2882053 2882057

III 2894442 2894473

III 2895147 2895156

III 2895324 2895333

III 2895585 2895609

III 2896188 2896201

III 2954469 2954501

III 2960783 2960791

III 2960927 2960940

III 2961047 2961056

III 2961129 2961141

III 2963240 2963315

III 2967061 2967090

III 2967196 2967209

III 2967398 2967419

III 2967769 2967900

III 2968107 2968274

III 2968385 2968397

III 2968568 2968577

III 2968684 2968856

III 2968949 2969095

III 2969362 2969404

III 2969760 2969780

III 2969933 2969948

III 2970057 2970068

III 3001566 3001620

III 3001713 3001764

III 3003820 3004228

III 3004420 3004495

III 3008969 3008986

III 3009171 3009181

III 3009363 3009670

III 3009825 3009873

III 3011279 3011589

III 3011647 3011792

III 3011853 3011865

III 3011962 3011972

III 3012035 3012130

III 3014937 3014947

III 3015026 3015034

III 3018278 3018398

III 3018724 3018854

III 3023941 3024451

III 3024510 3024543

III 3024596 3024615

III 3024770 3024810

III 3026306 3026348

III 3026402 3026490

III 3030724 3030725

III 3032308 3032312

III 3050279 3050518

III 3050591 3050830

III 3053234 3053268

III 3053961 3053969

III 3054150 3054158

III 3054408 3054433

III 3055019 3055031

III 3078612 3078659

III 3078754 3078800

III 3078905 3078918

III 3079140 3079191

III 3079286 3079405

III 3080598 3080602

III 3080731 3080777

III 3080963 3080986

III 3081083 3081102

III 3083318 3083337

III 3085499 3085544

III 3085639 3085685

III 3085796 3085804

III 3086029 3086078

III 3086174 3086294

III 3119122 3119166

III 3119240 3119274

III 3119486 3119533

III 3119732 3119781

III 3119816 3119914

III 3121460 3121577

III 3121734 3121819

III 3121994 3122024

III 3123435 3123491

III 3123572 3123897

III 3123946 3124053

III 3157770 3157771

III 3173305 3173537

III 3173619 3173852

III 3180572 3181112

III 3181366 3181600

III 3181664 3181697

III 3181790 3181801

III 3182085 3182093

III 3182238 3182762

III 3187745 3187794

III 3187884 3188088

III 3188180 3188300

III 3192521 3192649

III 3193420 3193427

III 3193849 3193865

III 3194312 3194341

III 3194601 3194706

III 3194998 3195072

III 3195209 3195315

III 3210678 3210815

III 3210876 3210903

III 3211016 3211071

III 3211300 3211372

III 3216604 3216676

III 3218419 3218499

III 3229190 3229197

III 3230623 3230646

III 3230744 3230868

III 3230920 3231157

III 3231251 3231313

III 3231467 3231575

III 3231669 3231747

III 3245325 3245338

III 3246243 3246261

III 3259876 3259886

III 3260176 3260234

III 3260454 3260523

III 3260576 3260598

III 3262383 3263537

III 3264973 3265097

III 3265334 3265480

III 3265558 3265581

III 3265764 3265817

III 3275660 3275685

III 3275772 3275922

III 3277479 3277630

III 3277720 3277739

III 3279988 3280018

III 3280131 3280173

III 3280252 3280697

III 3292970 3293105

III 3293497 3293506

III 3297707 3297718

III 3297914 3297917

III 3298023 3298155

III 3324837 3324849

III 3325879 3325894

III 3332484 3332867

III 3332960 3332980

III 3334139 3334271

III 3334385 3334801

III 3334895 3336379

III 3336455 3336906

III 3337364 3337389

III 3346877 3346882

III 3362028 3362156

III 3362275 3362294

III 3362385 3362401

III 3362631 3362654

III 3385227 3385362

III 3385453 3385556

III 3385760 3385842

III 3385936 3386216

III 3386305 3386522

III 3386609 3386648

III 3386718 3386720

III 3412275 3412370

III 3412458 3412496

III 3412582 3412700

III 3412804 3413055

III 3413105 3413107

III 3413291 3413440

III 3433401 3433613

III 3433743 3433775

III 3439887 3439998

III 3444847 3444867

III 3445264 3445345

III 3445410 3445483

III 3446280 3446305

III 3448081 3449034

III 3449104 3450545

III 3450635 3450668

III 3450763 3451352

III 3451580 3451585

III 3454334 3454336

III 3454431 3454588

III 3454681 3454710

III 3454800 3454863

III 3455011 3455127

III 3455222 3455443

III 3455538 3455590

III 3455711 3455728

III 3456089 3456096

III 3489887 3489951

III 3517180 3517236

III 3517289 3517387

III 3517500 3517618

III 3517712 3517728

III 3517812 3517825

III 3517935 3518077

III 3518245 3518276

III 3518331 3518341

III 3528687 3528715

III 3528808 3528817

III 3540881 3540927

III 3543936 3543956

III 3544044 3544062

III 3544150 3544433

III 3544483 3544724

III 3544810 3545102

III 3566958 3566965

III 3666064 3666067

III 3666472 3666477

III 3666642 3666690

III 3666770 3666818

III 3693130 3693137

III 3693384 3693401

III 3695633 3695710

III 3695805 3696013

III 3696439 3696444

III 3696953 3696989

III 3697309 3697354

III 3711880 3711935

III 3740316 3740333

III 3740416 3740465

III 3747006 3747039

III 3758747 3758872

III 3758970 3758973

III 3770522 3770598

III 3770727 3770762

III 3833826 3834006

III 3834287 3834384

III 3836488 3836677

III 3836915 3837012

III 3837747 3837829

III 3838068 3838300

III 3838851 3838859

III 3839039 3839136

III 3839350 3839419

III 3839537 3839543

III 3840092 3840130

III 3840239 3840256

III 3840439 3840503

III 3840597 3840770

III 3840923 3841051

III 3841253 3841314

III 3841508 3841526

III 3841672 3841831

III 3841956 3841970

III 3842094 3842124

III 3842221 3842346

III 3842924 3842931

III 3843024 3843122

III 3843377 3843398

III 3843510 3843557

III 3843769 3843801

III 3844547 3844844

III 3845062 3845244

III 3845471 3845662

III 3845989 3846104

III 3846223 3846243

III 3846356 3846401

III 3846463 3846471

III 3846647 3846704

III 3846798 3846879

III 3846971 3847050

III 3847167 3847346

III 3847644 3847834

III 3848039 3848282

III 3848432 3848473

III 3848673 3848696

III 3849391 3849535

III 3849890 3849892

III 3849994 3850155

III 3850349 3850353

III 3850486 3850542

III 3850633 3850643

III 3850848 3850936

III 3851562 3851578

III 3851700 3851845

III 3852510 3852517

III 3852744 3852756

III 3852852 3852877

III 3852991 3853078

III 3853172 3853226

III 3853348 3853365

III 3853454 3853478

III 3854000 3854053

III 3854733 3854743

III 3855013 3855022

III 3855112 3855257

III 3855315 3855771

III 3855868 3855888

III 3855980 3856391

III 3856470 3856486

III 3856575 3856990

III 3857078 3857225

III 3857431 3857464

III 3857769 3857906

III 3859368 3860537

III 3860849 3860908

III 3861056 3861155

III 3894898 3895035

III 3895079 3895222

III 3895293 3895418

III 3895555 3895694

III 3895860 3896003

III 3896109 3896152

III 3896330 3896583

III 3896668 3896837

III 3896999 3897489

III 3897595 3897738

III 3906710 3906755

III 3932040 3932063

III 3932229 3932283

III 3932335 3932550

III 3950330 3950333

III 3950546 3950547

III 3950548 3950553

III 3950609 3950620

III 3950773 3950795

III 3987122 3987656

III 4001567 4001601

III 4041264 4041399

III 4041495 4041500

III 4042459 4042493

III 4042878 4043014

III 4043206 4043337

III 4043480 4043655

III 4043750 4044345

III 4044438 4044857

III 4044949 4045637

III 4045732 4045846

III 4045937 4046019

III 4046117 4046274

III 4046401 4046414

III 4046507 4046633

III 4046728 4046777

III 4047770 4047899

III 4048633 4048639

III 4052981 4053113

III 4053206 4053211

III 4054105 4054137

III 4060962 4060976

III 4061052 4061135

III 4062896 4063007

III 4075542 4075543

III 4075544 4075546

III 4148924 4149041

III 4240113 4240124

III 4254188 4254745

III 4254829 4255412

III 4255504 4255539

III 4255602 4255705

III 4255815 4255819

III 4255920 4256504

III 4256567 4257138

III 4298129 4298943

III 4299015 4299120

III 4299172 4299293

III 4350890 4350919

III 4351946 4351994

III 4369562 4369660

III 4379013 4379109

III 4379160 4379177

III 4381197 4381212

III 4389320 4389375

III 4389664 4389672

III 4402076 4402102

III 4406763 4406897

III 4414227 4414250

III 4425149 4425155

III 4426831 4426919

III 4428557 4428567

III 4431411 4434255

III 4436610 4439455

III 4479405 4479462

III 4479677 4479735

III 4479824 4479832

III 4479920 4479994

III 4480046 4480208

III 4484530 4484545

III 4484694 4484774

III 4484859 4484883

III 4484977 4485180

III 4485301 4485338

III 4543690 4543714

III 4544757 4544804

III 4626345 4626353

III 4629280 4629283

III 4629284 4629287

III 4629357 4629365

III 4629841 4629861

III 4632863 4632871

III 4632919 4632947

III 4633740 4633751

III 4635881 4636027

III 4636784 4636794

III 4637932 4638113

III 4638908 4638924

III 4639033 4639059

III 4639298 4639321

III 4648469 4648538

III 4654388 4654467

III 4670605 4670611

III 4675729 4675755

III 4678214 4678316

III 4683768 4683785

III 4684117 4684260

III 4685190 4685221

III 4736842 4736867

III 4737096 4737173

III 4741470 4741473

III 4742424 4742449

III 4742547 4742762

III 4744604 4745105

III 4745353 4745629

III 4745873 4745957

III 4749371 4750997

III 4751160 4752720

III 4760275 4760355

III 4760436 4760524

III 4760613 4760627

III 4782527 4782783

III 4782972 4782988

III 4783225 4783276

III 4783469 4783521

III 4783686 4783687

III 4783772 4783814

III 4783907 4784272

III 4784375 4784552

III 4784682 4784903

III 4785028 4785062

III 4816651 4816904

III 4821249 4821506

III 4830809 4830880

III 4830976 4830980

III 4838669 4838767

III 4839243 4839331

III 4839455 4839470

III 4842837 4842868

III 4873898 4873921

III 4922109 4922183

III 4922274 4922308

III 4957738 4957799

III 4978443 4978527

III 4978770 4979043

III 4979342 4979840

III 4980616 4980832

III 4980933 4980955

III 4981116 4981120

III 4984704 4984751

III 5002758 5002853

III 5002949 5003121

III 5025781 5025850

III 5025973 5025977

III 5030616 5030721

III 5031508 5031573

III 5031665 5031673

III 5034760 5034927

III 5048759 5048767

III 5048858 5048928

III 5054752 5055272

III 5055481 5055826

III 5056385 5056484

III 5062639 5062979

III 5063188 5063712

III 5080838 5080857

III 5084706 5084866

III 5091046 5091084

III 5091172 5091181

III 5091298 5091478

III 5091568 5091644

III 5092203 5092206

III 5092960 5092963

III 5098494 5098517

III 5099866 5099870

III 5099933 5100119

III 5100382 5100667

III 5100802 5100858

III 5100944 5101094

III 5145492 5145512

III 5145610 5145615

III 5145708 5145714

III 5156402 5156411

III 5168919 5169006

III 5169193 5169262

III 5172363 5172420

III 5178354 5178360

III 5211253 5211264

III 5211356 5211422

III 5281567 5281592

III 5282481 5282816

III 5282912 5283445

III 5283536 5283624

III 5283715 5283721

III 5285066 5285432

III 5285512 5285517

III 5324265 5324406

III 5324646 5324696

III 5324812 5324815

III 5337227 5337304

III 5337427 5337524

III 5337930 5337977

III 5340095 5340161

III 5340284 5340296

III 5352088 5352233

III 5352437 5352608

III 5352696 5352835

III 5352992 5353120

III 5353267 5353854

III 5353968 5353979

III 5354049 5354228

III 5354279 5354776

III 5354868 5355072

III 5355131 5355406

III 5355477 5355679

III 5355790 5355902

III 5356017 5356192

III 5356269 5356456

III 5356514 5356667

III 5356785 5356838

III 5356893 5356942

III 5357002 5357100

III 5357170 5357308

III 5357497 5357890

III 5357977 5358142

III 5358229 5358234

III 5358437 5358484

III 5358939 5359089

III 5360352 5360376

III 5360531 5360786

III 5365069 5365291

III 5365383 5365407

III 5365544 5365761

III 5365847 5366341

III 5366414 5366472

III 5374769 5374801

III 5409871 5409898

III 5409992 5410098

III 5410260 5410300

III 5410473 5410476

III 5410568 5410616

III 5410709 5410951

III 5437362 5437397

III 5456272 5456281

III 5456338 5456379

III 5456512 5456644

III 5459510 5459563

III 5459691 5460254

III 5460323 5460428

III 5460486 5460631

III 5460721 5460725

III 5460816 5460841

III 5460964 5461026

III 5481055 5481120

III 5488492 5488591

III 5491888 5491986

III 5495645 5495710

III 5495980 5495983

III 5518091 5518092

III 5570418 5570483

III 5627645 5627712

III 5636036 5636118

III 5636178 5636266

III 5636317 5636396

III 5636582 5636690

III 5636785 5636799

III 5637087 5637106

III 5637435 5637438

III 5637535 5637547

III 5647008 5647116

III 5750667 5750676

III 5755374 5755379

III 5778874 5779194

III 5779285 5779926

III 5780127 5780692

III 5780901 5781481

III 5782532 5782540

III 5782993 5783006

III 5783458 5783472

III 5783715 5783748

III 5783981 5783986

III 5784484 5784502

III 5784683 5784763

III 5784876 5785091

III 5786387 5786426

III 5786565 5786583

III 5786796 5786818

III 5786869 5786975

III 5787191 5787246

III 5787469 5787527

III 5787699 5787762

III 5787929 5788090

III 5788261 5788731

III 5788813 5788912

III 5789089 5789772

III 5848069 5848096

III 5850400 5850450

III 5850602 5850651

III 5850748 5850761

III 5929816 5929845

III 5929897 5929901

III 5930367 5930398

III 5941760 5941812

III 5943230 5943279

III 5987705 5987733

III 6013107 6013315

III 6038122 6038144

III 6041166 6041188

III 6054818 6054837

III 6062884 6062954

III 6063050 6063185

III 6063322 6063395

III 6063697 6063723

III 6063795 6063834

III 6063932 6064050

III 6064299 6064411

III 6064534 6064700

III 6064999 6065005

III 6065132 6065209

III 6065333 6065445

III 6065503 6065799

III 6065921 6066021

III 6066283 6066330

III 6066538 6066591

III 6109775 6109777

III 6126343 6126372

III 6133507 6133576

III 6150508 6150545

III 6150630 6150670

III 6150818 6151004

III 6151096 6151105

III 6151152 6151301

III 6151494 6151750

III 6151843 6151858

III 6152143 6152154

III 6152241 6152282

III 6152457 6152547

III 6152636 6152692

III 6152782 6152799

III 6152950 6153064

III 6153167 6153275

III 6184127 6184266

III 6184316 6184353

III 6184441 6184575

III 6184665 6184746

III 6185132 6185153

III 6185250 6185257

III 6185316 6185900

III 6186024 6186062

III 6186918 6186963

III 6187054 6187062

III 6187137 6187292

III 6187347 6187381

III 6187454 6187608

III 6187718 6187754

III 6187818 6187890

III 6188127 6188179

III 6301754 6301878

III 6337095 6337112

III 6337599 6337625

III 6400070 6400074

III 6501736 6501757

III 6502115 6502258

III 6502408 6502424

III 6502597 6502680

III 6502782 6502845

III 6502952 6503011

III 6503105 6503121

III 6503524 6503648

III 6503746 6503795

III 6503912 6503924

III 6503937 6503959

III 6504179 6504273

III 6504461 6504505

III 6504573 6504787

III 6504900 6504955

III 6505473 6505475

III 6511260 6511300

III 6511437 6511670

III 6514111 6514176

III 6516161 6516187

III 6541948 6541999

III 6542441 6542623

III 6543192 6543418

III 6546998 6549093

III 6549187 6549218

III 6551417 6551426

III 6551564 6551576

III 6552194 6552213

III 6552306 6552336

III 6552452 6552481

III 6552569 6552579

III 6552664 6552669

III 6552733 6552764

III 6552819 6552832

III 6553037 6553142

III 6553325 6553381

III 6553473 6553510

III 6554011 6554060

III 6554504 6554684

III 6555235 6555456

III 6559518 6561614

III 6561710 6561739

III 6564475 6564476

III 6564940 6565266

III 6565457 6565535

III 6565790 6565855

III 6566874 6567658

III 6567740 6568402

III 6568461 6568921

III 6568995 6569351

III 6569412 6570908

III 6570957 6571437

III 6571503 6572386

III 6572454 6572784

III 6572869 6573999

III 6574057 6574439

III 6574514 6574727

III 6574911 6574950

III 6575028 6575179

III 6575314 6575323

III 6575864 6576994

III 6590303 6590328

III 6590419 6590425

III 6617340 6617384

III 6617598 6617661

III 6617770 6617946

III 6618174 6618193

III 6618384 6618411

III 6618551 6618552

III 6622256 6622579

III 6625945 6626046

III 6626545 6626657

III 6626936 6626991

III 6627083 6627086

III 6627208 6627311

III 6627535 6627572

III 6627786 6627818

III 6627882 6627885

III 6627888 6627891

III 6628198 6628408

III 6628503 6628522

III 6628703 6628921

III 6629086 6629590

III 6629751 6629838

III 6629895 6630049

III 6630195 6630316

III 6630410 6630554

III 6630777 6631161

III 6631257 6631264

III 6631369 6631380

III 6631559 6631646

III 6648419 6648480

III 6648573 6648579

III 6648793 6648952

III 6659613 6660849

III 6660940 6661092

III 6661179 6661188

III 6661637 6661681

III 6661772 6661832

III 6661947 6662043

III 6662213 6662270

III 6662572 6662590

III 6662751 6662815

III 6662991 6663015

III 6663150 6663157

III 6663303 6663576

III 6663743 6664236

III 6664436 6664683

III 6664770 6664804

III 6685323 6685572

III 6685641 6685718

III 6685800 6686049

III 6740715 6740822

III 6768265 6768266

III 6771346 6771358

III 6771360 6771361

III 6771465 6771495

III 6772522 6774374

III 6774467 6774643

III 6774737 6775704

III 6775798 6776426

III 6777788 6777907

III 6778115 6778144

III 6778282 6779543

III 6779658 6779695

III 6780814 6781846

III 6781919 6783846

III 6783941 6784120

III 6784215 6785672

III 6785747 6785901

III 6788121 6788135

III 6858055 6858100

III 6877887 6877948

III 6878230 6878248

III 6884658 6884664

III 6884852 6884873

III 6914309 6914407

III 6916021 6916120

III 6917113 6917322

III 6917465 6917574

III 6917668 6917730

III 6917823 6918013

III 6918113 6918774

III 6918841 6919450

III 6919538 6919661

III 6919715 6919724

III 6919801 6920167

III 6930883 6930897

III 6931004 6931009

III 6940720 6940733

III 6940839 6940843

III 6961025 6961040

III 6968223 6968229

III 6974723 6974757

III 6982141 6982357

III 6982536 6982664

III 6982755 6983080

III 6983145 6983165

III 6983258 6983449

III 6983658 6983785

III 6983967 6983997

III 6984089 6984267

III 6984446 6984506

III 6984588 6984602

III 6984690 6984742

III 6984831 6984992

III 6985056 6985395

III 6997940 6998047

III 6998145 6998294

III 6998419 7000140

III 7000243 7000749

III 7000838 7001316

III 7001439 7001543

III 7010622 7010649

III 7052213 7052217

III 7089195 7089202

III 7089278 7089317

III 7105407 7105912

III 7106008 7106567

III 7125280 7125717

III 7125811 7125834

III 7125998 7126076

III 7126144 7126162

III 7126366 7126412

III 7126868 7126971

III 7127079 7127097

III 7127363 7127364

III 7127518 7127529

III 7127619 7127706

III 7127801 7127802

III 7127803 7127805

III 7127899 7128052

III 7128537 7128713

III 7129134 7129215

III 7129483 7129923

III 7130014 7130044

III 7130201 7130275

III 7130472 7130572

III 7130759 7130801

III 7130911 7131005

III 7131097 7131112

III 7131201 7131271

III 7131367 7131430

III 7131527 7131609

III 7131695 7131755

III 7140364 7140371

III 7142475 7142479

III 7151462 7151580

III 7151672 7151776

III 7151867 7152129

III 7152379 7152443

III 7152538 7152648

III 7171180 7171661

III 7171755 7173601

III 7174063 7175908

III 7176000 7176482

III 7204385 7204449

III 7234674 7234708

III 7234910 7235077

III 7235205 7235224

III 7235364 7235509

III 7235890 7236028

III 7237963 7237965

III 7260645 7261795

III 7303231 7303240

III 7306480 7307130

III 7307204 7307987

III 7326516 7326526

III 7327159 7327180

III 7328160 7328172

III 7328669 7328683

III 7328774 7328810

III 7333994 7334017

III 7334101 7334105

III 7362499 7363727

III 7364122 7365353

III 7386651 7386688

III 7386773 7386844

III 7388475 7388561

III 7404272 7404597

III 7404727 7405332

III 7405445 7405806

III 7405854 7406027

III 7406075 7406711

III 7406800 7408905

III 7409002 7409569

III 7409625 7410681

III 7410771 7411473

III 7411545 7412593

III 7412667 7414316

III 7414572 7414912

III 7415002 7415207

III 7415441 7415489

III 7415573 7416463

III 7416520 7416833

III 7416930 7417041

III 7417096 7417375

III 7417450 7419188

III 7419249 7419914

III 7419987 7422633

III 7422683 7423245

III 7423352 7424888

III 7424964 7425151

III 7425294 7425401

III 7425492 7425504

III 7425599 7425822

III 7425881 7426017

III 7426099 7426186

III 7426262 7426268

III 7426352 7426618

III 7426678 7426682

III 7426897 7427107

III 7427173 7433573

III 7433659 7434248

III 7434374 7436058

III 7436136 7436508

III 7436559 7437184

III 7437303 7438224

III 7438291 7438372

III 7438541 7438749

III 7438815 7439413

III 7439544 7440111

III 7440203 7443097

III 7443191 7443243

III 7443333 7443655

III 7443786 7444390

III 7444503 7444905

III 7445000 7445414

III 7445502 7445634

III 7445773 7445813

III 7445862 7446101

III 7446154 7446512

III 7446745 7446892

III 7447092 7447104

III 7447278 7447313

III 7447460 7447726

III 7447809 7447816

III 7447876 7448054

III 7448288 7448798

III 7448863 7449266

III 7449354 7449383

III 7449476 7452228

III 7452736 7452751

III 7456102 7456145

III 7469149 7469232

III 7469318 7469451

III 7469598 7469698

III 7469812 7469991

III 7470082 7470217

III 7470439 7470465

III 7470544 7470560

III 7470647 7470648

III 7470743 7470916

III 7471008 7471070

III 7471221 7471986

III 7472079 7472350

III 7472441 7472564

III 7472656 7472721

III 7472767 7473037

III 7473124 7473477

III 7473581 7473838

III 7473935 7474018

III 7474108 7474156

III 7474241 7474363

III 7474456 7474535

III 7474682 7474987

III 7475162 7475197

III 7475300 7475540

III 7475628 7475950

III 7476043 7476056

III 7476145 7476610

III 7476699 7476806

III 7477108 7477197

III 7477758 7477759

III 7548218 7548226

III 7548335 7548359

III 7549845 7550157

III 7550213 7550251

III 7550324 7550655

III 7550809 7551003

III 7563491 7563497

III 7563567 7563613

III 7576462 7576535

III 7591805 7591847

III 7592078 7592095

III 7592221 7592223

III 7592411 7592420

III 7592523 7592532

III 7593753 7593795

III 7594902 7594938

III 7595031 7595110

III 7595302 7595984

III 7596075 7596518

III 7596629 7596762

III 7596833 7596922

III 7597005 7597253

III 7597899 7597927

III 7598012 7598026

III 7598140 7598143

III 7598241 7598327

III 7598765 7598916

III 7599005 7599053

III 7599356 7599377

III 7599595 7599775

III 7599873 7599917

III 7600025 7600064

III 7600167 7600182

III 7600597 7600679

III 7600743 7600777

III 7600876 7600985

III 7601149 7601198

III 7601293 7601325

III 7601549 7601578

III 7602031 7602791

III 7602871 7603192

III 7603254 7603528

III 7603611 7603655

III 7604018 7604031

III 7604593 7604652

III 7642701 7642710

III 7642788 7642803

III 7646418 7646506

III 7646637 7646653

III 7646757 7646995

III 7647104 7647156

III 7668698 7668860

III 7669032 7669172

III 7669294 7669376

III 7670609 7670691

III 7671702 7671805

III 7672005 7672010

III 7672182 7672192

III 7673141 7673244

III 7673905 7674232

III 7674345 7674367

III 7674422 7674424

III 7674644 7675262

III 7677093 7677118

III 7678212 7678310

III 7678360 7678410

III 7678712 7678980

III 7679262 7679616

III 7679754 7679878

III 7680034 7680055

III 7680243 7680317

III 7680563 7680688

III 7680779 7680822

III 7680976 7681053

III 7681147 7681204

III 7681327 7681651

III 7681749 7682273

III 7682400 7683014

III 7683107 7683363

III 7683440 7683705

III 7708687 7708717

III 7708837 7708847

III 7708920 7708972

III 7713078 7713096

III 7713167 7713224

III 7713375 7713511

III 7713622 7713742

III 7713899 7713975

III 7714118 7714142

III 7714217 7714257

III 7714605 7714613

III 7714866 7715001

III 7715053 7715058

III 7715318 7715363

III 7715457 7715469

III 7715636 7715726

III 7715846 7715914

III 7716035 7716080

III 7716255 7716274

III 7716469 7716595

III 7770300 7770355

III 7770451 7770697

III 7770788 7771091

III 7774414 7774583

III 7820818 7820900

III 7821016 7821143

III 7821253 7821470

III 7821560 7821711

III 7881696 7881830

III 7943325 7943330

III 7943411 7943501

III 7944646 7944661

III 7953571 7953682

III 7953770 7953797

III 7954013 7954125

III 7954224 7954238

III 7954463 7954567

III 7954727 7954749

III 7959398 7959424

III 7959519 7959638

III 7959732 7959748

III 7960008 7960014

III 7960078 7960278

III 7960369 7960374

III 7960485 7960513

III 7960772 7960814

III 7961008 7961137

III 7961423 7961447

III 7961789 7961798

III 7962276 7962316

III 7962588 7962714

III 7962972 7962984

III 7963213 7963240

III 7963382 7963406

III 7963633 7963672

III 7963921 7963967

III 7964219 7964221

III 7964448 7964515

III 7964614 7964645

III 7965905 7965914

III 7966098 7966108

III 7966205 7966324

III 7966419 7966442

III 7997670 7997779

III 8032163 8032218

III 8032373 8032383

III 8074233 8074250

III 8104628 8104760

III 8129332 8129339

III 8158992 8159084

III 8181159 8181163

III 8189322 8189363

III 8189461 8189512

III 8189990 8189999

III 8203566 8204736

III 8205056 8205084

III 8222865 8222876

III 8222978 8222987

III 8223290 8223308

III 8223542 8223585

III 8254546 8254555

III 8299566 8299568

III 8304731 8304776

III 8304844 8304856

III 8304969 8305044

III 8306588 8307253

III 8335216 8335217

III 8335628 8335651

III 8335735 8335969

III 8336152 8336280

III 8336373 8343712

III 8343810 8344438

III 8344561 8344811

III 8344870 8351909

III 8352005 8352501

III 8355936 8355948

III 8356045 8356129

III 8384706 8384707

III 8384829 8384986

III 8401429 8401467

III 8415069 8415076

III 8415116 8415123

III 8415321 8415400

III 8415526 8415651

III 8424432 8424498

III 8438218 8438307

III 8441357 8441374

III 8441663 8441709

III 8441819 8442495

III 8442644 8443251

III 8464024 8464229

III 8464317 8464491

III 8464582 8464670

III 8480001 8480004

III 8486959 8487100

III 8487157 8487199

III 8523105 8523141

III 8523227 8523244

III 8531983 8531993

III 8540041 8540309

III 8540362 8540729

III 8540826 8540970

III 8541041 8541173

III 8541283 8541292

III 8541342 8541422

III 8541516 8541541

III 8565479 8566626

III 8629379 8629524

III 8629649 8629666

III 8629850 8629920

III 8630561 8630592

III 8656859 8656876

III 8694401 8696660

III 8712277 8712284

III 8735339 8735801

III 8735875 8736249

III 8736405 8736507

III 8755980 8756042

III 8756560 8756667

III 8772286 8772401

III 8792627 8792741

III 8810240 8810285

III 8810545 8810592

III 8813271 8813390

III 8813509 8814221

III 8814301 8814328

III 8814464 8814524

III 8851213 8851284

III 8852643 8853038

III 8861016 8861414

III 8861838 8861855

III 8861939 8861991

III 8862042 8862401

III 8862491 8862492

III 8862588 8862590

III 8862714 8863088

III 8863242 8863750

III 8863999 8864029

III 8864168 8864201

III 8868929 8868948

III 8869032 8869049

III 8898244 8898309

III 8900998 8901009

III 8920130 8920198

III 8920505 8920588

III 8920878 8920915

III 8921033 8921094

III 8924309 8924376

III 8924609 8924687

III 8924974 8925013

III 8925130 8925190

III 8935652 8935876

III 8959312 8959334

III 8959407 8960435

III 8989930 8989991

III 8990115 8990122

III 9006209 9006259

III 9006354 9006410

III 9013714 9013750

III 9013868 9013895

III 9013948 9014214

III 9077037 9077047

III 9099392 9099408

III 9100180 9100472

III 9100562 9100565

III 9100660 9100785

III 9101981 9102104

III 9102198 9102202

III 9102294 9102583

III 9103275 9103287

III 9110703 9110772

III 9110858 9110889

III 9110977 9110999

III 9111001 9111002

III 9161073 9161200

III 9161279 9161409

III 9236780 9236833

III 9236916 9237009

III 9237098 9237136

III 9237233 9237355

III 9249818 9249831

III 9253699 9253757

III 9291803 9291845

III 9318252 9318267

III 9320667 9320681

III 9361825 9361892

III 9371558 9371562

III 9384444 9384771

III 9391504 9391532

III 9392177 9392247

III 9392431 9392445

III 9392571 9392715

III 9404520 9404525

III 9406894 9406896

III 9407082 9407212

III 9407302 9407352

III 9407438 9407498

III 9407550 9407650

III 9407902 9407926

III 9408437 9408465

III 9408914 9408976

III 9409324 9409351

III 9409458 9409617

III 9409711 9409773

III 9410165 9410269

III 9410374 9410452

III 9410599 9410708

III 9410839 9410894

III 9410990 9411248

III 9411336 9411351

III 9411567 9411982

III 9412037 9412129

III 9412235 9412256

III 9412350 9412426

III 9412521 9412793

III 9413012 9413082

III 9414603 9414607

III 9415983 9416015

III 9416233 9416411

III 9416475 9416755

III 9416875 9417335

III 9417444 9418374

III 9418575 9418576

III 9418579 9418581

III 9418696 9419296

III 9419388 9419614

III 9419690 9420096

III 9420190 9420320

III 9423029 9423034

III 9445800 9445837

III 9445931 9445980

III 9447784 9447840

III 9447928 9447969

III 9449624 9449643

III 9487982 9488003

III 9504264 9504409

III 9526026 9526044

III 9533600 9533620

III 9533710 9533905

III 9534095 9534521

III 9534941 9534946

III 9535040 9535206

III 9544464 9544546

III 9566462 9566605

III 9574276 9574760

III 9574848 9575093

III 9575140 9575559

III 9575594 9575619

III 9596618 9596663

III 9596845 9596909

III 9601591 9601922

III 9604199 9604524

III 9604724 9605080

III 9605162 9605196

III 9605292 9605307

III 9605403 9605433

III 9638039 9638042

III 9651262 9651291

III 9652614 9652622

III 9674814 9675058

III 9675206 9675543

III 9681322 9681371

III 9681472 9681518

III 9682935 9682947

III 9683086 9683181

III 9683267 9683401

III 9683493 9683548

III 9683635 9683683

III 9683772 9683796

III 9683900 9683925

III 9684031 9684068

III 9684158 9684172

III 9684349 9684375

III 9684519 9684565

III 9684621 9684641

III 9684778 9684805

III 9684896 9684936

III 9711133 9711192

III 9716850 9716906

III 9835804 9835811

III 9836105 9836109

III 9836259 9836690

III 9849477 9849498

III 9849592 9849647

III 9852015 9852238

III 9852346 9852430

III 9852606 9852624

III 9893689 9893752

III 9893824 9893878

III 9894181 9894198

III 9894342 9894362

III 9897658 9897720

III 9897815 9898012

III 9898092 9898109

III 9907908 9908007

III 9908118 9908223

III 9914892 9914951

III 9915548 9915627

III 9915722 9915754

III 9916076 9916788

III 9916999 9917070

III 9917327 9917350

III 9917430 9917448

III 9917750 9917788

III 9917883 9917924

III 9918073 9918197

III 9918303 9918310

III 9923480 9923541

III 9924510 9924589

III 9924686 9924714

III 9925511 9926230

III 9935841 9935853

III 9935949 9935985

III 9940619 9940649

III 9959561 9959590

III 9962410 9962415

III 9962535 9962545

III 10050349 10050475

III 10050555 10050577

III 10050666 10050703

III 10050800 10050876

III 10051551 10051616

III 10051716 10051837

III 10052267 10052275

III 10052442 10052449

III 10052788 10052851

III 10052944 10052969

III 10053263 10053294

III 10053384 10053423

III 10053573 10053592

III 10053758 10053832

III 10053924 10054135

III 10054217 10054233

III 10054328 10054424

III 10102226 10102295

III 10102378 10102399

III 10123564 10123570

III 10123651 10123682

III 10139850 10139885

III 10140068 10140078

III 10140227 10140235

III 10140293 10140604

III 10142902 10142968

III 10143094 10143099

III 10143541 10143658

III 10144276 10144314

III 10144678 10144712

III 10144804 10145045

III 10145185 10145400

III 10145491 10145670

III 10145764 10145830

III 10145973 10145989

III 10146077 10146152

III 10146349 10146354

III 10147075 10147093

III 10147341 10147588

III 10147684 10147693

III 10147884 10147893

III 10148184 10148236

III 10148591 10148945

III 10149381 10149399

III 10219168 10220574

III 10220688 10221741

III 10221830 10222098

III 10224390 10224411

III 10224474 10224477

III 10224722 10224759

III 10224850 10225199

III 10225297 10225583

III 10225759 10225862

III 10225939 10226004

III 10226215 10227760

III 10230170 10230185

III 10242660 10242819

III 10261043 10261177

III 10267048 10267098

III 10267496 10267513

III 10267604 10267668

III 10267996 10267997

III 10268217 10268227

III 10268309 10268333

III 10268446 10268459

III 10268835 10269262

III 10269351 10269371

III 10269623 10269661

III 10269767 10269823

III 10269984 10270092

III 10270292 10270301

III 10270409 10270477

III 10270569 10270796

III 10316974 10317038

III 10317090 10317182

III 10317278 10317841

III 10320457 10320547

III 10326854 10326881

III 10327129 10327189

III 10361652 10361697

III 10375078 10375168

III 10375402 10375665

III 10394995 10395024

III 10395133 10395165

III 10436385 10436549

III 10436728 10436784

III 10436875 10436953

III 10437205 10437368

III 10437671 10437732

III 10438079 10438095

III 10438314 10438638

III 10438798 10438942

III 10443109 10443201

III 10443499 10444340

III 10444474 10444803

III 10453035 10453371

III 10453508 10454341

III 10454574 10454667

III 10468297 10468503

III 10476487 10476509

III 10481198 10481325

III 10542203 10542380

III 10554921 10554942

III 10555031 10555128

III 10555747 10555763

III 10555991 10556003

III 10556304 10557474

III 10576437 10576507

III 10586713 10586720

III 10587008 10587094

III 10587187 10587226

III 10587336 10587656

III 10587754 10587774

III 10587852 10587973

III 10588063 10588143

III 10588236 10588265

III 10588360 10588464

III 10588562 10588645

III 10602266 10602280

III 10602417 10602431

III 10608937 10609131

III 10637560 10637588

III 10637737 10637757

III 10654414 10654437

III 10654646 10654668

III 10661997 10662046

III 10674805 10674896

III 10675004 10675034

III 10678121 10678155

III 10678388 10678400

III 10678462 10678506

III 10678581 10678655

III 10683407 10683542

III 10683656 10684282

III 10684407 10684450

III 10684648 10684685

III 10684828 10684859

III 10684973 10685063

III 10687826 10687838

III 10687943 10687956

III 10688177 10688193

III 10733587 10733645

III 10733704 10733746

III 10733832 10733928

III 10750826 10751384

III 10751475 10751498

III 10751586 10751610

III 10752059 10752067

III 10752126 10752140

III 10752215 10752428

III 10752520 10752969

III 10753056 10753088

III 10753393 10753408

III 10753731 10753766

III 10753933 10753950

III 10754276 10754304

III 10792055 10792434

III 10792519 10793416

III 10796513 10796538

III 10804641 10804644

III 10805663 10805672

III 10811101 10811103

III 10811219 10811316

III 10811383 10811390

III 10845217 10845261

III 10846130 10846505

III 10846691 10847028

III 10847121 10847123

III 10847222 10847329

III 10847379 10847390

III 10847443 10847545

III 10863991 10863995

III 10893950 10893973

III 10894088 10894128

III 10919210 10919365

III 10919452 10919481

III 10940208 10940251

III 10941199 10941223

III 10943816 10943917

III 10944005 10944040

III 10944158 10944307

III 10944382 10944415

III 10944928 10945033

III 10948601 10948652

III 10948739 10948749

III 10948828 10948871

III 10948958 10948968

III 10958106 10958279

III 10958363 10958585

III 10967969 10968172

III 10968553 10968558

III 10968617 10968624

III 10982065 10982407

III 10982502 10982947

III 10984688 10984764

III 10984859 10984875

III 10985046 10985063

III 10985153 10985230

III 10985327 10985400

III 10986731 10987660

III 10989720 10989736

III 11004719 11005433

III 11005483 11006343

III 11027105 11027123

III 11027202 11027431

III 11027683 11027748

III 11027882 11028252

III 11028445 11028453

III 11028547 11028550

III 11028664 11028738

III 11028974 11028975

III 11029302 11029425

III 11029545 11029564

III 11029648 11029682

III 11029804 11029949

III 11040066 11040374

III 11071396 11071400

III 11085818 11086008

III 11088195 11088209

III 11089624 11089647

III 11103839 11104064

III 11104952 11105006

III 11105192 11105755

III 11105919 11106378

III 11112514 11112533

III 11113147 11113188

III 11113290 11113318

III 11113810 11113857

III 11115066 11115141

III 11117360 11117922

III 11118112 11118128

III 11118371 11118384

III 11118618 11119080

III 11120435 11120487

III 11121324 11121545

III 11124622 11124649

III 11127422 11127432

III 11129143 11129155

III 11131585 11131613

III 11131692 11131706

III 11134652 11134699

III 11148501 11148572

III 11148813 11148886

III 11150137 11150264

III 11150383 11150510

III 11150856 11151113

III 11151172 11151364

III 11151529 11151566

III 11158867 11158904

III 11160128 11160391

III 11160469 11160916

III 11161046 11161182

III 11161260 11161439

III 11161495 11161644

III 11161839 11161898

III 11162803 11162948

III 11163254 11163345

III 11166012 11166168

III 11166217 11166558

III 11166700 11166726

III 11169266 11169387

III 11177281 11177283

III 11177333 11177371

III 11189332 11189363

III 11189456 11189485

III 11190058 11190093

III 11192484 11192485

III 11195081 11195124

III 11223902 11223906

III 11224347 11224444

III 11226156 11226216

III 11242882 11243028

III 11243116 11243216

III 11252750 11252757

III 11252852 11252914

III 11253012 11253021

III 11253079 11253106

III 11253201 11253464

III 11253542 11253634

III 11253704 11253744

III 11270493 11270554

III 11271571 11271644

III 11271865 11271908

III 11293172 11293186

III 11293343 11293354

III 11293426 11293882

III 11294893 11295317

III 11298218 11298639

III 11303808 11304131

III 11307235 11307249

III 11307531 11307549

III 11309004 11309306

III 11309353 11309427

III 11309522 11309562

III 11309681 11309736

III 11320507 11320837

III 11320997 11321218

III 11329277 11329444

III 11329511 11329723

III 11329812 11329851

III 11330400 11330406

III 11373609 11373613

III 11373708 11373752

III 11373835 11373839

III 11373933 11373980

III 11376656 11377617

III 11381232 11381264

III 11381398 11381434

III 11399898 11400147

III 11400201 11400227

III 11426522 11426573

III 11429153 11429875

III 11430040 11430474

III 11430547 11430767

III 11430841 11431362

III 11431434 11431849

III 11431938 11432248

III 11432329 11432461

III 11432539 11433285

III 11433365 11433804

III 11433878 11434922

III 11435016 11435025

III 11435076 11435092

III 11435164 11435176

III 11435259 11435280

III 11435341 11435932

III 11436028 11436079

III 11436175 11436229

III 11436300 11436422

III 11436559 11436726

III 11494327 11494367

III 11497241 11497263

III 11498607 11498653

III 11514502 11514504

III 11514692 11514829

III 11515058 11515185

III 11518451 11518524

III 11518620 11518668

III 11518755 11518776

III 11518903 11519129

III 11519184 11519198

III 11519352 11519406

III 11519572 11519664

III 11520599 11520692

III 11520857 11520908

III 11525003 11525477

III 11525654 11526120

III 11532602 11532634

III 11546828 11547139

III 11547217 11547531

III 11549067 11549078

III 11549181 11549186

III 11553313 11553395

III 11553508 11553640

III 11553712 11553730

III 11570373 11570660

III 11570745 11571085

III 11575739 11575921

III 11575976 11576025

III 11576111 11576455

III 11578156 11578168

III 11578287 11578302

III 11579809 11579838

III 11580077 11580108

III 11585437 11585443

III 11585529 11585608

III 11585727 11585797

III 11585939 11585999

III 11590674 11590692

III 11593537 11596690

III 11596830 11596876

III 11596933 11601184

III 11601247 11601387

III 11601469 11602308

III 11602478 11602586

III 11603275 11603288

III 11603459 11603460

III 11603786 11603796

III 11603973 11603982

III 11604149 11604198

III 11605004 11605063

III 11605319 11607575

III 11609071 11609158

III 11609280 11609490

III 11613928 11613930

III 11613931 11613934

III 11619886 11619952

III 11622009 11622010

III 11642183 11642501

III 11642590 11642795

III 11642853 11642979

III 11643054 11645601

III 11645680 11646198

III 11646301 11647567

III 11653282 11653437

III 11653525 11653619

III 11653783 11653792

III 11653893 11654016

III 11654102 11654158

III 11654245 11654356

III 11654423 11654439

III 11654526 11654561

III 11655136 11655171

III 11661436 11661453

III 11662568 11662592

III 11669853 11669961

III 11670058 11670171

III 11671821 11671873

III 11672327 11672374

III 11682495 11682505

III 11687382 11687397

III 11707322 11707562

III 11710438 11710444

III 11723809 11723817

III 11723879 11723886

III 11734049 11734087

III 11734182 11734508

III 11734645 11734706

III 11734771 11735075

III 11735170 11735193

III 11735399 11735673

III 11735746 11736293

III 11736422 11736580

III 11736632 11736843

III 11739283 11739297

III 11739420 11739476

III 11739590 11739764

III 11739821 11739852

III 11739924 11739959

III 11741626 11741651

III 11744284 11744298

III 11744438 11744495

III 11746375 11746387

III 11773837 11774401

III 11774463 11774480

III 11787534 11787714

III 11787810 11789453

III 11790535 11792180

III 11792275 11792456

III 11807481 11807499

III 11821490 11821496

III 11822172 11822191

III 11827031 11827439

III 11827622 11827747

III 11835428 11835793

III 11839974 11839987

III 11840133 11840358

III 11840580 11840584

III 11840586 11840595

III 11851943 11851982

III 11852065 11852085

III 11852150 11852347

III 11852423 11852487

III 11852629 11852643

III 11857431 11857441

III 11857528 11857565

III 11857745 11857760

III 11857816 11857853

III 11857936 11857940

III 11857992 11858133

III 11859686 11859761

III 11859890 11859907

III 11860059 11860132

III 11860277 11860301

III 11860486 11860516

III 11860591 11860596

III 11860905 11860911

III 11864665 11864710

III 11864765 11864830

III 11866544 11866571

III 11866723 11866762

III 11867017 11867019

III 11867534 11867536

III 11868043 11868058

III 11868244 11868273

III 11873056 11873072

III 11873216 11873225

III 11873310 11873362

III 11883364 11883475

III 11883870 11883921

III 11884014 11884017

III 11884018 11884021

III 11884112 11884260

III 11884575 11884626

III 11885030 11885183

III 11890714 11890722

III 11892147 11892189

III 11894083 11894127

III 11897957 11897990

III 11903450 11903468

III 11903701 11903719

III 11905166 11905208

III 11905295 11905391

III 11905450 11905498

III 11907617 11907704

III 11909029 11909040

III 11925803 11925818

III 11926361 11926477

III 11926537 11926666

III 11927457 11927475

III 11933279 11933307

III 11939387 11939507

III 11939583 11939686

III 11940018 11940105

III 11940200 11940320

III 11949167 11949212

III 11950687 11950713

III 11950808 11950823

III 11953038 11953054

III 11953903 11953925

III 11954717 11954722

III 11955075 11955096

III 11955229 11955286

III 11956365 11956378

III 11956539 11956597

III 11958834 11958999

III 11959147 11959779

III 11964940 11965325

III 11965420 11965929

III 11966294 11966452

III 11966522 11966543

III 11966672 11966806

III 11967094 11967120

III 11967208 11967243

III 11967845 11968025

III 11968119 11968176

III 11975001 11975102

III 11975191 11975218

III 11975312 11975418

III 11982993 11982999

III 11983277 11983280

III 11990634 11990789

III 11993480 11993525

III 11993847 11993926

III 12004311 12004503

III 12004738 12004778

III 12005276 12005353

III 12005583 12005642

III 12006083 12006104

III 12007210 12007630

III 12007719 12007910

III 12008248 12008249

III 12008337 12008429

III 12008484 12008502

III 12008584 12008653

III 12008736 12008824

III 12010788 12010808

III 12010960 12011057

III 12011169 12011197

III 12011269 12011289

III 12011382 12011456

III 12011522 12011546

III 12011687 12011800

III 12011910 12011961

III 12012100 12012142

III 12012412 12012414

III 12024561 12024570

III 12025979 12026370

III 12026584 12026641

III 12027982 12027990

III 12042163 12042188

III 12049502 12049607

III 12049744 12049851

III 12056074 12056087

III 12056208 12056286

III 12056367 12056384

III 12056497 12056572

III 12056639 12056641

III 12056686 12056786

III 12057390 12057430

III 12057697 12057705

III 12057783 12057827

III 12057916 12057934

III 12059923 12059973

III 12066587 12066598

III 12066899 12066907

III 12068621 12068635

III 12069179 12069203

III 12070276 12070301

III 12070586 12070607

III 12072462 12072542

III 12072672 12072917

III 12072967 12073117

III 12073204 12073205

III 12073262 12073498

III 12073572 12073608

III 12074511 12074514

III 12076022 12076046

III 12099397 12099526

III 12099605 12099647

III 12099812 12099876

III 12099957 12099982

III 12100034 12100253

III 12112318 12112338

III 12143969 12143974

III 12161573 12161596

III 12161708 12161781

III 12161897 12162055

III 12162146 12162171

III 12162227 12162240

III 12162991 12162992

III 12164336 12164338

III 12169846 12170363

III 12170418 12170546

III 12172517 12172538

III 12172712 12172879

III 12172996 12173158

III 12173212 12173213

III 12173270 12173289

III 12175430 12175438

III 12177423 12177457

III 12177808 12177827

III 12180169 12180200

III 12180279 12180397

III 12180693 12180813

III 12190808 12190822

III 12192759 12192867

III 12192930 12192935

III 12193846 12193847

III 12196398 12196462

III 12200294 12200313

III 12202190 12202194

III 12204329 12204593

III 12209461 12209640

III 12209730 12209829

III 12209902 12209931

III 12210087 12210118

III 12210253 12210515

III 12210657 12210777

III 12211058 12211128

III 12211327 12211555

III 12211624 12211696

III 12211834 12211874

III 12212021 12212033

III 12212259 12212285

III 12212336 12212510

III 12220145 12220151

III 12222524 12222563

III 12222703 12222713

III 12227055 12227072

III 12231282 12231306

III 12239426 12239629

III 12239758 12239794

III 12242273 12242296

III 12242526 12242547

III 12242737 12242795

III 12242879 12242916

III 12246203 12246221

III 12247750 12247762

III 12252130 12252198

III 12254738 12254891

III 12254963 12255121

III 12255195 12255449

III 12262713 12262727

III 12265544 12265737

III 12265888 12265993

III 12266084 12266176

III 12266293 12266378

III 12266471 12266551

III 12266649 12266671

III 12266964 12267003

III 12267090 12267124

III 12267630 12267762

III 12267812 12268208

III 12268295 12268338

III 12272470 12272480

III 12279108 12279146

III 12279275 12279300

III 12279381 12279450

III 12279586 12279611

III 12279840 12279854

III 12280000 12280001

III 12281731 12281745

III 12288235 12288270

III 12289792 12289813

III 12289906 12289948

III 12290126 12290193

III 12290271 12290371

III 12290467 12290512

III 12290653 12290693

III 12290765 12290797

III 12290998 12291003

III 12295423 12295521

III 12296828 12296881

III 12298560 12298777

III 12315848 12316049

III 12316238 12316252

III 12316532 12316535

III 12316818 12316839

III 12317027 12317068

III 12317337 12317344

III 12320471 12320475

III 12321819 12321863

III 12321999 12322042

III 12323240 12323320

III 12323444 12323459

III 12327387 12327389

III 12328431 12328454

III 12328620 12328639

III 12328736 12328754

III 12328842 12328856

III 12336354 12336358

III 12339101 12339165

III 12339242 12339307

III 12339374 12339383

III 12339524 12339588

III 12339653 12339664

III 12343978 12344033

III 12344507 12344514

III 12346071 12346172

III 12346270 12346538

III 12346612 12346929

III 12347015 12347171

III 12347256 12347420

III 12347508 12347678

III 12347761 12347882

III 12347974 12348136

III 12349887 12349891

III 12354271 12354316

III 12362986 12363120

III 12363780 12363946

III 12366469 12366493

III 12378603 12378631

III 12380868 12380874

III 12381042 12381065

III 12381133 12381409

III 12381536 12381681

III 12381814 12381888

III 12381954 12381992

III 12382104 12382178

III 12382352 12382412

III 12382845 12382914

III 12383006 12383029

III 12383260 12383268

III 12383357 12383384

III 12383703 12383709

III 12385562 12385569

III 12386685 12387046

III 12387345 12387369

III 12387572 12387581

III 12389638 12389776

III 12390506 12390556

III 12390677 12390720

III 12390856 12390870

III 12391160 12391290

III 12391443 12391501

III 12391784 12391844

III 12396430 12396507

III 12397234 12397243

III 12403840 12403873

III 12405462 12405501

III 12405617 12405653

III 12408751 12408767

III 12415875 12415957

III 12416016 12416582

III 12423308 12423738

III 12423834 12423849

III 12423938 12424021

III 12432823 12432857

III 12433315 12433317

III 12433413 12433432

III 12437718 12437804

III 12438125 12438195

III 12438318 12438332

III 12441423 12441449

III 12441546 12441615

III 12441686 12441709

III 12441800 12441860

III 12442111 12442166

III 12442249 12442303

III 12444734 12444739

III 12447730 12447748

III 12451421 12451425

III 12451498 12451575

III 12451936 12451985

III 12458784 12458816

III 12460921 12460941

III 12461030 12461042

III 12462221 12462231

III 12463663 12463693

III 12466196 12466200

III 12467239 12467278

III 12471345 12471364

III 12476392 12476409

III 12476475 12476611

III 12476725 12476833

III 12476887 12476918

III 12476994 12477280

III 12477379 12477512

III 12477622 12477760

III 12477841 12478140

III 12478236 12478261

III 12478318 12478521

III 12478706 12478811

III 12478876 12478899

III 12479010 12479087

III 12486256 12486269

III 12491703 12491715

III 12493405 12493425

III 12493481 12493539

III 12494057 12494117

III 12494296 12494305

III 12502035 12502180

III 12502356 12502376

III 12502469 12502536

III 12502689 12502807

III 12502857 12502864

III 12503057 12503061

III 12504049 12504563

III 12504629 12504761

III 12507128 12507171

III 12508867 12508872

III 12508997 12509016

III 12512876 12512877

III 12512997 12513010

III 12513718 12514538

III 12514591 12515208

III 12518447 12519608

III 12520413 12520453

III 12520666 12520689

III 12520840 12520871

III 12523566 12523599

III 12525052 12525187

III 12525500 12525622

III 12529605 12529643

III 12530905 12530952

III 12531317 12531410

III 12531484 12531512

III 12531616 12531704

III 12531864 12531890

III 12538574 12538637

III 12541250 12541692

III 12541775 12542212

III 12542322 12542328

III 12544999 12545166

III 12545265 12545508

III 12545592 12545593

III 12545644 12545691

III 12546002 12546032

III 12546151 12546166

III 12546241 12546249

III 12546838 12546841

III 12546842 12546843

III 12550584 12550705

III 12550801 12550926

III 12551798 12551863

III 12551957 12552113

III 12552568 12552923

III 12571924 12571927

III 12572029 12572042

III 12578661 12578703

III 12579103 12579125

III 12580049 12580106

III 12580287 12580353

III 12586325 12586343

III 12586441 12586463

III 12586536 12586557

III 12586646 12586685

III 12586762 12586783

III 12590329 12590350

III 12591314 12591352

III 12594247 12594534

III 12594588 12594613

III 12594802 12595157

III 12595316 12595346

III 12595398 12595423

III 12595488 12595529

III 12595579 12595655

III 12596641 12596649

III 12596947 12596955

III 12610311 12610353

III 12610556 12610558

III 12620959 12620989

III 12621099 12621388

III 12621666 12621701

III 12621764 12621815

III 12623102 12623141

III 12623342 12623423

III 12627198 12627222

III 12627412 12627422

III 12627580 12627595

III 12627835 12627970

III 12628037 12628109

III 12628231 12628340

III 12628487 12628546

III 12631567 12631568

III 12632518 12632597

III 12633342 12633362

III 12634612 12634634

III 12636602 12636665

III 12636714 12636772

III 12636854 12636981

III 12637120 12637122

III 12637195 12637254

III 12643951 12643958

III 12646104 12646131

III 12646269 12646357

III 12646501 12646612

III 12646819 12646928

III 12646985 12647005

III 12647224 12647308

III 12647381 12647558

III 12647654 12647693

III 12647788 12647828

III 12648157 12648158

III 12648250 12648282

III 12648428 12648443

III 12648680 12648732

III 12649167 12649302

III 12649379 12649399

III 12652911 12652948

III 12656908 12657141

III 12657236 12657301

III 12657429 12657460

III 12661003 12661071

III 12661223 12661251

III 12661384 12661389

III 12661510 12661512

III 12665741 12665745

III 12665827 12665858

III 12665932 12665954

III 12666343 12666370

III 12667181 12667206

III 12672355 12672406

III 12672552 12672562

III 12672701 12672720

III 12679357 12679395

III 12679549 12679624

III 12679707 12679799

III 12682207 12682252

III 12684218 12684227

III 12684842 12684947

III 12685183 12685187

III 12691981 12691996

III 12694653 12694665

III 12694756 12694862

III 12694998 12695111

III 12695251 12695320

III 12695415 12695454

III 12696801 12696828

III 12697059 12697077

III 12698125 12698126

III 12698260 12698488

III 12698648 12698723

III 12698817 12698832

III 12699945 12700037

III 12700114 12701010

III 12701594 12701638

III 12701790 12701835

III 12702780 12702782

III 12702865 12703010

III 12703660 12703672

III 12703745 12703972

III 12704032 12704270

III 12704352 12704537

III 12704614 12704721

III 12705167 12705185

III 12705474 12705491

III 12705786 12705815

III 12705863 12705920

III 12706061 12706080

III 12706938 12707058

III 12707310 12707620

III 12708407 12708441

III 12708535 12708629

III 12715991 12716337

III 12716470 12716691

III 12720891 12720895

III 12724723 12724728

III 12730405 12730422

III 12730609 12730623

III 12734612 12734699

III 12735934 12735959

III 12736095 12736254

III 12736423 12736531

III 12739643 12739645

III 12741155 12741335

III 12741400 12741580

III 12743203 12743204

III 12751667 12751689

III 12760899 12760900

III 12760973 12761124

III 12762154 12762175

III 12762505 12762526

III 12767001 12767341

III 12767732 12767756

III 12767918 12767926

III 12768501 12768965

III 12769111 12769213

III 12783792 12783830

III 12786548 12786612

III 12790550 12790568

III 12790764 12790773

III 12790940 12790943

III 12791035 12791128

III 12791238 12791299

III 12791392 12791462

III 12804276 12804287

III 12804441 12804450

III 12805886 12805912

III 12811588 12811639

III 12812783 12812803

III 12820414 12820437

III 12820675 12820697

III 12822296 12822355

III 12835811 12835906

III 12835968 12836068

III 12843335 12843389

III 12843670 12843728

III 12848204 12848244

III 12848305 12848344

III 12851966 12852046

III 12852139 12852233

III 12862703 12862710

III 12864394 12864406

III 12865205 12866736

III 12868375 12868379

III 12868562 12868563

III 12878096 12878106

III 12878167 12878331

III 12878480 12878798

III 12878888 12878957

III 12879047 12879463

III 12879549 12879866

III 12879958 12880236

III 12880329 12880423

III 12880572 12880577

III 12880710 12880754

III 12882091 12882125

III 12885939 12885990

III 12886122 12886154

III 12890184 12890207

III 12890311 12890320

III 12890943 12890952

III 12891051 12891076

III 12895180 12895207

III 12903260 12903475

III 12903564 12903675

III 12903759 12903955

III 12908848 12908890

III 12918954 12919036

III 12922186 12922198

III 12923019 12923020

III 12923300 12923531

III 12923582 12923615

III 12923703 12924043

III 12927020 12927025

III 12930065 12930083

III 12933263 12933422

III 12933513 12933679

III 12938799 12938880

III 12956281 12956283

III 12963706 12963746

III 12965760 12965779

III 12970256 12970318

III 12970417 12970460

III 12972792 12972830

III 12973014 12973072

III 12973301 12973367

III 12973612 12973643

III 12974190 12974293

III 12974357 12974402

III 12985499 12985682

III 12985772 12985831

III 13010740 13010851

III 13012512 13012540

III 13013007 13013122

III 13023293 13023342

III 13023767 13023906

III 13026531 13026578

III 13027750 13027892

III 13032335 13032359

III 13066654 13066660

III 13072665 13072671

III 13074795 13074825

III 13074916 13074920

III 13074970 13075586

III 13075637 13076352

III 13076405 13076826

III 13076919 13077464

III 13077514 13077887

III 13077936 13079723

III 13079802 13080032

III 13080121 13080192

III 13080272 13082261

III 13082337 13082372

III 13082771 13086729

III 13087123 13087149

III 13088121 13088131

III 13088310 13088344

III 13088527 13088528

III 13094375 13094425

III 13095213 13095265

III 13100243 13100290

III 13101527 13101592

III 13105115 13105146

III 13109873 13109879

III 13110066 13110079

III 13110180 13110247

III 13114924 13115007

III 13115199 13115282

III 13117746 13117800

III 13117936 13118008

III 13123930 13123991

III 13126718 13126719

III 13129598 13129699

III 13134862 13134922

III 13141347 13141375

III 13141785 13141811

III 13142478 13142491

III 13142745 13142807

III 13143129 13143152

III 13143201 13143302

III 13144555 13144564

III 13145388 13145409

III 13147860 13147979

III 13148069 13148564

III 13148660 13148667

III 13149149 13149190

III 13149492 13149547

III 13158092 13158133

III 13158296 13158378

III 13161750 13161801

III 13161864 13161897

III 13162311 13162334

III 13172305 13172316

III 13176690 13176708

III 13186677 13186685

III 13189044 13189053

III 13189201 13189350

III 13191031 13191145

III 13193820 13193860

III 13193956 13194264

III 13194315 13194353

III 13194411 13194465

III 13195606 13195718

III 13196541 13196546

III 13199054 13199136

III 13199508 13199554

III 13199691 13199792

III 13200256 13200311

III 13203049 13203051

III 13203052 13203053

III 13206758 13206792

III 13216347 13216361

III 13229658 13230020

III 13230070 13230082

III 13230388 13230391

III 13230725 13230749

III 13231737 13231852

III 13233409 13233425

III 13233515 13233620

III 13233797 13233841

III 13234553 13234557

III 13235137 13235266

III 13235802 13235839

III 13236486 13236565

III 13236622 13236792

III 13237035 13237104

III 13239726 13239742

III 13242275 13242388

III 13242704 13243091

III 13243145 13243155

III 13243689 13245214

III 13270514 13270527

III 13290195 13290282

III 13290451 13290453

III 13293180 13293318

III 13293410 13294150

III 13294217 13295262

III 13295358 13295493

III 13295576 13296214

III 13296265 13296499

III 13315812 13315818

III 13315918 13315952

III 13316084 13316111

III 13327075 13327545

III 13327639 13327942

III 13327995 13328417

III 13337580 13337767

III 13337897 13338082

III 13369626 13369826

III 13369929 13370120

III 13379066 13379068

III 13398182 13398243

III 13398344 13398395

III 13398488 13398630

III 13398815 13398836

III 13399092 13399179

III 13402017 13402158

III 13402247 13402333

III 13414826 13414928

III 13415010 13415541

III 13415677 13415791

III 13415876 13416686

III 13416780 13416791

III 13416868 13417041

III 13417156 13417249

III 13417422 13417668

III 13428734 13428798

III 13446631 13446662

III 13446834 13447357

III 13447447 13447474

III 13447612 13447758

III 13447841 13447872

III 13447925 13448024

III 13448308 13448622

III 13449427 13449490

III 13451127 13451205

III 13451546 13451574

III 13451702 13451707

III 13453498 13453549

III 13453788 13454001

III 13454167 13454193

III 13468882 13468930

III 13476297 13476298

III 13497225 13497335

III 13497437 13497765

III 13498038 13498316

III 13498386 13498417

III 13499045 13499096

III 13508709 13508735

III 13517166 13517210

III 13517301 13517373

III 13517436 13517522

III 13522082 13522393

III 13522477 13522696

III 13522746 13523377

III 13523465 13523609

III 13531672 13532301

III 13532383 13533093

III 13533166 13533189

III 13533247 13533824

III 13533910 13534429

III 13535189 13535256

III 13584330 13584372

III 13584444 13584580

III 13584877 13585227

III 13585276 13585312

III 13585407 13585723

III 13585789 13585834

III 13585928 13585947

III 13586041 13586086

III 13586195 13586475

III 13586544 13586689

III 13586781 13587066

III 13587121 13587159

III 13587242 13587987

III 13588051 13588361

III 13588610 13588691

III 13588791 13589320

III 13589384 13589706

III 13589829 13590064

III 13590156 13590340

III 13590421 13590467

III 13590561 13590567

III 13590682 13591131

III 13591207 13591504

III 13591557 13592172

III 13592330 13592388

III 13592514 13593355

III 13593514 13593756

III 13593823 13594706

III 13594865 13595488

III 13595579 13595587

III 13595668 13596165

III 13596296 13597023

III 13597106 13597395

III 13597580 13597661

III 13597710 13597876

III 13597925 13598182

III 13598312 13598500

III 13598568 13598947

III 13599007 13599132

III 13599211 13599216

III 13599217 13599221

III 13599484 13599665

III 13599948 13600008

III 13600059 13600201

III 13600627 13600709

III 13600967 13600969

III 13601132 13601151

III 13601221 13601319

III 13601412 13601490

III 13604260 13604265

III 13609950 13610212

III 13610302 13610520

III 13610623 13610739

III 13610831 13610953

III 13611061 13611159

III 13611229 13611397

III 13611489 13611619

III 13611670 13611671

III 13611749 13611806

III 13611900 13611927

III 13612027 13612286

III 13612404 13612517

III 13612607 13613070

III 13613148 13613432

III 13613555 13613626

III 13613720 13613760

III 13625077 13625141

III 13627317 13627322

III 13631157 13631531

III 13631618 13631665

III 13631792 13631878

III 13631953 13632186

III 13632270 13632288

III 13632436 13632514

III 13681054 13681057

III 13725482 13725603

III 13725724 13725738

III 13725847 13726020

III 13726173 13726192

III 13739173 13739213

III 13740916 13740927

III 13741385 13741420

III 13741535 13741547

III 13748856 13749000

III 13749104 13749245

III 13749318 13749449

III 13749505 13750137

III 13750201 13750342

III 13750423 13750499

III 13750679 13750824

III 13750949 13751348

III 13751904 13751929

III 13752897 13753526

III 13753615 13753681

III 13753735 13753849

III 13753905 13753955

III 13754006 13754429

III 13754489 13754521

III 13754547 13754645

III 13754726 13754970

III 13774415 13774437

III 13775443 13776210

III 13776293 13776739

III 13776822 13777267

III 13777362 13777566

III 13777662 13777865

III 13777953 13777967

III 13778058 13778201

III 13779298 13779447

III 13779549 13779554

III 13780136 13780154

III 13781388 13782467

III 13782563 13782769

III 13782865 13783066

III 13783243 13783386

III 13783496 13783511

III 13783637 13783801

IV 0 105

IV 195 1121

IV 1216 2290

IV 2386 4639

IV 4735 5450

IV 5545 8695

IV 8792 12936

IV 13035 17474

IV 17535 18987

IV 19077 23607

IV 24272 28804

IV 28898 30344

IV 30430 34850

IV 34939 39088

IV 39184 42332

IV 42428 43145

IV 43236 45495

IV 45588 46661

IV 46757 47685

IV 52412 52467

IV 58521 58591

IV 60867 61603

IV 61717 61755

IV 61846 62037

IV 62121 62379

IV 62492 62511

IV 62684 62815

IV 62904 62990

IV 63092 63128

IV 63264 63521

IV 63616 63689

IV 63793 63942

IV 64041 64065

IV 64158 64229

IV 64319 64569

IV 64632 64890

IV 64978 65078

IV 65128 65246

IV 65336 65622

IV 65704 66116

IV 66251 66389

IV 66479 66509

IV 66616 66947

IV 82974 83050

IV 83191 83215

IV 83317 83327

IV 83463 83525

IV 83595 83617

IV 83705 83716

IV 84314 84319

IV 84413 84544

IV 85529 86438

IV 86487 86548

IV 111297 111331

IV 111424 111445

IV 111506 111617

IV 114402 114410

IV 114567 114628

IV 116682 116718

IV 116812 116857

IV 116950 117001

IV 117088 117099

IV 117358 117549

IV 117641 118121

IV 118210 118337

IV 118559 118569

IV 118726 118784

IV 123073 123254

IV 125083 125117

IV 125210 125245

IV 125341 125374

IV 130886 130892

IV 160324 160330

IV 160420 160574

IV 160761 160872

IV 181474 181477

IV 185496 185532

IV 191559 191575

IV 195750 195756

IV 195952 196047

IV 196331 196553

IV 200436 200447

IV 200518 200534

IV 206500 206525

IV 209059 209077

IV 212283 212438

IV 212530 212586

IV 215865 215866

IV 215867 215887

IV 216006 216007

IV 216225 216274

IV 221232 221512

IV 221608 222017

IV 222144 223362

IV 223417 224142

IV 224219 224431

IV 224483 224500

IV 224658 224808

IV 227794 227865

IV 227954 227968

IV 228190 228251

IV 229942 230108

IV 230204 230282

IV 230359 230520

IV 230607 230857

IV 238679 238739

IV 239088 239209

IV 260787 260853

IV 266749 266764

IV 267236 267249

IV 280800 280946

IV 281119 281158

IV 281337 281456

IV 281537 281593

IV 281643 281711

IV 281889 281957

IV 284585 284636

IV 301254 301266

IV 332830 332842

IV 333133 333239

IV 335383 335654

IV 335812 335876

IV 349008 350496

IV 353409 353416

IV 353523 353567

IV 353634 353674

IV 353729 353834

IV 357016 357054

IV 384244 384309

IV 385411 385504

IV 397319 397350

IV 397614 397652

IV 430083 430220

IV 432202 432468

IV 440901 441242

IV 446648 446660

IV 446710 446711

IV 446799 446973

IV 447203 447336

IV 449579 449996

IV 450094 450751

IV 450914 450986

IV 451117 451149

IV 451511 451575

IV 452482 452536

IV 452628 452668

IV 452778 453114

IV 453172 453387

IV 453467 453520

IV 453608 453752

IV 457668 457674

IV 458152 458204

IV 501845 501968

IV 502044 502252

IV 502455 502688

IV 502921 503262

IV 503527 503649

IV 505506 505542

IV 505653 505689

IV 507730 510925

IV 511005 513263

IV 520068 520100

IV 528652 528679

IV 528774 528839

IV 529018 529197

IV 529325 529354

IV 541424 541500

IV 574004 574060

IV 574154 574170

IV 574260 574380

IV 574472 574551

IV 574701 574709

IV 574822 574884

IV 574973 574989

IV 624205 624253

IV 628230 628445

IV 632675 632685

IV 632772 632887

IV 633151 633165

IV 633234 633235

IV 633241 633244

IV 633295 633297

IV 651986 651999

IV 660168 660182

IV 665101 665240

IV 688782 692543

IV 692680 693388

IV 693565 693630

IV 693718 695657

IV 695887 698186

IV 701949 705147

IV 705223 707483

IV 721310 721322

IV 721544 721579

IV 721646 721791

IV 722044 722400

IV 722534 722555

IV 722667 722685

IV 722796 722804

IV 723476 723602

IV 723874 724004

IV 724111 724168

IV 770156 770275

IV 770492 770537

IV 770631 770726

IV 770901 770987

IV 771199 771214

IV 771309 771407

IV 771502 772149

IV 785074 785314

IV 785395 785471

IV 785548 785567

IV 785935 786051

IV 788471 788720

IV 788815 788885

IV 788953 789034

IV 789106 789178

IV 808968 809025

IV 809117 809125

IV 810698 810923

IV 811034 811387

IV 812804 813147

IV 813249 813348

IV 813448 813470

IV 813903 813920

IV 814420 814606

IV 814667 814717

IV 814828 814941

IV 815114 815135

IV 815210 815285

IV 816071 816189

IV 816299 816307

IV 816358 816461

IV 817738 817753

IV 818436 818709

IV 818877 818890

IV 819016 819246

IV 819347 819610

IV 820380 820474

IV 820583 820592

IV 820685 820719

IV 822428 822801

IV 822853 823103

IV 826039 826042

IV 826571 826572

IV 826877 826948

IV 827108 827200

IV 827304 827310

IV 849059 849117

IV 852541 852821

IV 867671 867708

IV 868264 868457

IV 869893 869897

IV 869956 870039

IV 890425 893674

IV 893754 906130

IV 906209 906732

IV 906789 911492

IV 911555 911565

IV 912996 913324

IV 921291 921621

IV 948758 948817

IV 948948 948955

IV 949042 949147

IV 950647 950676

IV 950756 951137

IV 951192 951384

IV 956743 956783

IV 957018 957151

IV 977965 977966

IV 978496 978503

IV 979672 979807

IV 979811 980012

IV 980107 980140

IV 980245 980378

IV 985013 985307

IV 985357 985436

IV 985521 985607

IV 986255 986549

IV 986743 986813

IV 986910 986983

IV 987051 987354

IV 987421 987471

IV 987594 987665

IV 988523 988635

IV 988691 988741

IV 988793 988816

IV 988896 989227

IV 991429 991435

IV 992652 992677

IV 1000381 1000414

IV 1020380 1020476

IV 1020555 1020597

IV 1020878 1021066

IV 1022226 1022530

IV 1025445 1025645

IV 1025777 1025809

IV 1025903 1026148

IV 1028547 1028559

IV 1028716 1028759

IV 1030652 1030663

IV 1030725 1030744

IV 1041909 1041984

IV 1051202 1051222

IV 1051671 1051814

IV 1051906 1051945

IV 1061875 1061977

IV 1072333 1072376

IV 1076713 1076856

IV 1078251 1078719

IV 1078796 1078824

IV 1078900 1078927

IV 1083859 1083918

IV 1083986 1084266

IV 1084316 1084366

IV 1084448 1084549

IV 1099637 1099991

IV 1100949 1101283

IV 1103920 1103925

IV 1125828 1125882

IV 1126064 1126067

IV 1134605 1134622

IV 1134805 1134920

IV 1135653 1135920

IV 1137548 1137579

IV 1137743 1137757

IV 1137990 1137998

IV 1138270 1138353

IV 1138448 1138655

IV 1149389 1149526

IV 1153713 1153746

IV 1153794 1153817

IV 1153913 1153936

IV 1153986 1154048

IV 1154155 1154253

IV 1154368 1154385

IV 1154464 1154557

IV 1154628 1154692

IV 1154880 1154931

IV 1154986 1155124

IV 1165494 1165511

IV 1165567 1165594

IV 1165754 1165808

IV 1165897 1165980

IV 1167005 1167062

IV 1167159 1167171

IV 1169619 1169624

IV 1170008 1170079

IV 1174435 1174446

IV 1175730 1175748

IV 1180062 1180169

IV 1180353 1180408

IV 1180500 1180518

IV 1180660 1180690

IV 1180823 1180877

IV 1181046 1181171

IV 1182477 1182562

IV 1184284 1184311

IV 1184504 1184578

IV 1185701 1185805

IV 1186186 1186262

IV 1186714 1186787

IV 1187461 1187471

IV 1188129 1188151

IV 1188212 1188415

IV 1188504 1188643

IV 1188738 1188830

IV 1190088 1190097

IV 1190191 1190231

IV 1190327 1190482

IV 1190591 1190738

IV 1191472 1191486

IV 1192143 1192284

IV 1192397 1192556

IV 1192654 1192691

IV 1192786 1192795

IV 1196321 1196551

IV 1196604 1197005

IV 1213743 1213749

IV 1217375 1217597

IV 1226927 1227145

IV 1244789 1244801

IV 1254159 1254255

IV 1258009 1258104

IV 1262547 1262626

IV 1262751 1262814

IV 1270496 1272463

IV 1272529 1272754

IV 1279530 1279818

IV 1279994 1280058

IV 1280145 1281516

IV 1281577 1282066

IV 1288672 1288693

IV 1288931 1289340

IV 1291829 1291849

IV 1294509 1294540

IV 1294635 1294693

IV 1295601 1295684

IV 1295873 1295875

IV 1296176 1296222

IV 1296994 1297099

IV 1298178 1298271

IV 1299430 1299538

IV 1300497 1300570

IV 1300973 1301002

IV 1301125 1301183

IV 1301270 1301427

IV 1301505 1301551

IV 1301990 1302080

IV 1302973 1302989

IV 1303278 1303298

IV 1304492 1304544

IV 1304743 1304858

IV 1306588 1306748

IV 1307072 1307423

IV 1308033 1308055

IV 1308413 1308439

IV 1315108 1315119

IV 1315960 1315969

IV 1319269 1319406

IV 1319602 1319745

IV 1324225 1324359

IV 1324552 1324623

IV 1327190 1327217

IV 1330235 1330367

IV 1330562 1330595

IV 1330647 1330827

IV 1330999 1331053

IV 1331193 1331341

IV 1334179 1334257

IV 1336013 1336038

IV 1336164 1336188

IV 1336330 1336360

IV 1336868 1336916

IV 1337011 1337108

IV 1338154 1338250

IV 1339645 1339649

IV 1339755 1339777

IV 1339869 1339930

IV 1340124 1340278

IV 1340373 1340518

IV 1340614 1340799

IV 1341072 1341258

IV 1341346 1341358

IV 1341452 1341499

IV 1341596 1341643

IV 1341775 1341940

IV 1342033 1342063

IV 1342151 1342165

IV 1342258 1342894

IV 1342990 1343147

IV 1343234 1343849

IV 1343945 1344250

IV 1344337 1344527

IV 1344624 1344733

IV 1344854 1345337

IV 1345409 1345497

IV 1345600 1345970

IV 1346075 1346206

IV 1346378 1346401

IV 1350458 1350541

IV 1350891 1351011

IV 1351239 1351314

IV 1352598 1352671

IV 1352999 1353115

IV 1353382 1353420

IV 1370191 1370304

IV 1370366 1370536

IV 1370762 1370770

IV 1371916 1372077

IV 1402071 1402108

IV 1402209 1402210

IV 1402408 1402519

IV 1403000 1403008

IV 1412621 1412663

IV 1413481 1413557

IV 1413993 1414012

IV 1422481 1422567

IV 1422658 1422663

IV 1423194 1423618

IV 1424479 1424500

IV 1424768 1424834

IV 1425565 1425988

IV 1426858 1426884

IV 1433611 1433612

IV 1433935 1433967

IV 1436079 1436210

IV 1436291 1436538

IV 1436624 1436793

IV 1439381 1439645

IV 1439767 1439792

IV 1439919 1440064

IV 1440197 1440203

IV 1440311 1440359

IV 1440451 1440694

IV 1440931 1441014

IV 1441141 1441144

IV 1441236 1441354

IV 1441647 1441648

IV 1441732 1441781

IV 1441875 1441925

IV 1442013 1442138

IV 1443307 1443349

IV 1443404 1443450

IV 1447072 1447153

IV 1447221 1447267

IV 1448569 1448584

IV 1448673 1448762

IV 1448855 1449208

IV 1449301 1449376

IV 1449467 1449552

IV 1449635 1449667

IV 1449758 1449813

IV 1449956 1449990

IV 1462992 1463020

IV 1463495 1463509

IV 1463598 1463681

IV 1473114 1473127

IV 1476189 1477116

IV 1477633 1477638

IV 1492428 1492560

IV 1492637 1492711

IV 1492782 1492788

IV 1492883 1492968

IV 1493026 1493143

IV 1498056 1498061

IV 1498140 1498411

IV 1498504 1498647

IV 1501079 1501357

IV 1501446 1501461

IV 1501555 1501570

IV 1501660 1501670

IV 1501741 1501787

IV 1502787 1502883

IV 1502974 1503004

IV 1503639 1504066

IV 1504142 1504257

IV 1504344 1504664

IV 1504757 1505082

IV 1505146 1505323

IV 1506784 1506856

IV 1507557 1507708

IV 1508001 1508144

IV 1511235 1512436

IV 1520896 1520990

IV 1528827 1528961

IV 1529519 1529776

IV 1530872 1530928

IV 1531013 1531020

IV 1531264 1531292

IV 1540085 1540234

IV 1541891 1541906

IV 1541982 1542031

IV 1543920 1543992

IV 1544113 1544151

IV 1557696 1557888

IV 1566964 1567070

IV 1567166 1567271

IV 1570194 1570345

IV 1570395 1570547

IV 1570596 1570603

IV 1575123 1575270

IV 1575404 1575726

IV 1576300 1576327

IV 1577820 1577847

IV 1579534 1579565

IV 1579932 1579944

IV 1582246 1582291

IV 1582502 1582525

IV 1582714 1582783

IV 1582842 1582845

IV 1583364 1583476

IV 1583570 1583664

IV 1583823 1584047

IV 1586082 1586135

IV 1586278 1586287

IV 1586382 1586648

IV 1586742 1587016

IV 1587127 1587246

IV 1587367 1587444

IV 1587517 1587709

IV 1587810 1588469

IV 1588559 1589360

IV 1589450 1589864

IV 1591541 1591567

IV 1592419 1592474

IV 1593161 1593239

IV 1597921 1598008

IV 1598812 1598829

IV 1599218 1599308

IV 1603658 1603848

IV 1603959 1603979

IV 1604060 1604364

IV 1607753 1607798

IV 1607842 1608250

IV 1608346 1608467

IV 1609523 1610236

IV 1611412 1611423

IV 1611508 1611606

IV 1611681 1611852

IV 1611918 1611964

IV 1612083 1612084

IV 1617306 1617323

IV 1617539 1619284

IV 1621902 1623648

IV 1623871 1623884

IV 1632771 1632807

IV 1640033 1640050

IV 1640114 1640530

IV 1641217 1641289

IV 1642165 1642443

IV 1642506 1642729

IV 1642822 1642840

IV 1645406 1645503

IV 1645786 1645787

IV 1645789 1645791

IV 1646032 1646033

IV 1646094 1646157

IV 1647869 1647886

IV 1648067 1648086

IV 1653094 1653272

IV 1653368 1653458

IV 1653554 1653798

IV 1653920 1653942

IV 1656537 1656612

IV 1656689 1656855

IV 1665735 1666446

IV 1668535 1669052

IV 1669106 1669107

IV 1669194 1669238

IV 1670704 1670878

IV 1671023 1671263

IV 1671410 1671447

IV 1677946 1678092

IV 1681328 1681331

IV 1692019 1692243

IV 1692313 1692416

IV 1692492 1692582

IV 1693536 1693542

IV 1693831 1693968

IV 1696537 1696544

IV 1696836 1696970

IV 1697101 1697139

IV 1702978 1703053

IV 1703138 1703150

IV 1703223 1703238

IV 1703429 1703733

IV 1703783 1703787

IV 1703937 1704317

IV 1704393 1704394

IV 1704396 1704443

IV 1704549 1704861

IV 1704945 1705089

IV 1705151 1705522

IV 1705621 1705787

IV 1705837 1705941

IV 1715157 1715246

IV 1719803 1719908

IV 1720003 1720237

IV 1721191 1721469

IV 1722172 1722451

IV 1725485 1725502

IV 1730271 1730318

IV 1730536 1730982

IV 1732314 1732372

IV 1732453 1732509

IV 1732616 1732882

IV 1733695 1733717

IV 1733806 1733839

IV 1734800 1734830

IV 1734920 1734956

IV 1735165 1735179

IV 1736336 1736437

IV 1740530 1740536

IV 1740689 1740714

IV 1742482 1742498

IV 1742588 1742619

IV 1743273 1743427

IV 1743510 1743717

IV 1754974 1755249

IV 1755298 1755455

IV 1756080 1756186

IV 1756250 1756297

IV 1756393 1756500

IV 1756898 1756948

IV 1757209 1757401

IV 1757640 1757657

IV 1760201 1760225

IV 1763456 1763460

IV 1765255 1765296

IV 1765457 1765480

IV 1765581 1765805

IV 1765807 1765808

IV 1767013 1767085

IV 1770599 1771209

IV 1771349 1771724

IV 1776653 1776673

IV 1776830 1777064

IV 1777146 1777232

IV 1777560 1777579

IV 1777667 1777844

IV 1777933 1778062

IV 1787966 1788173

IV 1792934 1792978

IV 1793071 1793191

IV 1793460 1793489

IV 1793586 1793714

IV 1793804 1793805

IV 1794501 1794555

IV 1794663 1794666

IV 1800905 1800939

IV 1808887 1808936

IV 1809096 1809288

IV 1809463 1809691

IV 1809772 1809864

IV 1809959 1810070

IV 1810120 1810233

IV 1810423 1810424

IV 1810474 1810808

IV 1811007 1811070

IV 1811276 1811335

IV 1811576 1811621

IV 1811717 1811749

IV 1811980 1812019

IV 1812406 1812431

IV 1812597 1812663

IV 1813069 1813082

IV 1813391 1813410

IV 1813497 1813556

IV 1828002 1828075

IV 1828365 1828377

IV 1828736 1828844

IV 1829001 1829302

IV 1829692 1829785

IV 1831132 1831148

IV 1831265 1831343

IV 1831487 1831513

IV 1848549 1848639

IV 1853319 1853323

IV 1854677 1854714

IV 1854845 1855119

IV 1855169 1855210

IV 1855294 1855677

IV 1855733 1855837

IV 1855915 1856110

IV 1856204 1856343

IV 1861892 1862036

IV 1862198 1862321

IV 1862403 1862591

IV 1866373 1866711

IV 1874357 1874381

IV 1874563 1874566

IV 1875191 1875212

IV 1878052 1878172

IV 1883028 1883084

IV 1884768 1885469

IV 1885569 1885602

IV 1888710 1888712

IV 1888713 1888714

IV 1891029 1891147

IV 1897115 1897131

IV 1897263 1897323

IV 1899033 1899071

IV 1899134 1899205

IV 1922084 1922118

IV 1925144 1925174

IV 1928668 1928684

IV 1931179 1931548

IV 1931601 1932158

IV 1932250 1932462

IV 1932609 1932987

IV 1933077 1933361

IV 1935686 1935810

IV 1935888 1935981

IV 1936073 1936259

IV 1938018 1938024

IV 1941201 1941221

IV 1949871 1949993

IV 1950530 1950631

IV 1950796 1950810

IV 1950927 1951178

IV 1951326 1951459

IV 1951768 1951963

IV 1952117 1952304

IV 1964883 1965107

IV 1966671 1966757

IV 1966851 1967347

IV 1967597 1967698

IV 1977052 1977064

IV 1977202 1977322

IV 1977405 1977519

IV 1980702 1980738

IV 1981988 1982000

IV 1982088 1982201

IV 1982365 1982386

IV 1982435 1982542

IV 1982718 1982784

IV 1982975 1982989

IV 1983162 1983175

IV 1983985 1983992

IV 1985024 1985196

IV 1987157 1987325

IV 1987743 1987779

IV 1987930 1987939

IV 1988731 1988739

IV 1988888 1988924

IV 1989865 1989997

IV 1992356 1992456

IV 1992566 1992612

IV 1997056 1997103

IV 1997288 1997336

IV 1997509 1997607

IV 1997719 1997764

IV 1998601 1999020

IV 1999169 1999291

IV 2000060 2000062

IV 2001769 2001774

IV 2005342 2005384

IV 2005471 2005529

IV 2007448 2007466

IV 2011373 2011390

IV 2011559 2011577

IV 2012922 2012941

IV 2013904 2013994

IV 2014106 2014430

IV 2014513 2014523

IV 2014588 2014677

IV 2014764 2014778

IV 2014997 2015091

IV 2015852 2015853

IV 2016080 2016086

IV 2016557 2017104

IV 2017162 2017170

IV 2017435 2017447

IV 2018228 2018257

IV 2018530 2018560

IV 2021949 2021952

IV 2028056 2028090

IV 2029693 2029726

IV 2034062 2034065

IV 2034557 2034622

IV 2034851 2034867

IV 2036090 2036109

IV 2046229 2046279

IV 2046359 2046505

IV 2046653 2046939

IV 2050582 2050604

IV 2050756 2050824

IV 2050943 2050984

IV 2051078 2051200

IV 2064349 2064352

IV 2064551 2064574

IV 2066856 2066960

IV 2067850 2067851

IV 2067913 2068468

IV 2068522 2068547

IV 2069736 2069800

IV 2069869 2069957

IV 2070100 2070305

IV 2070399 2070459

IV 2082730 2082782

IV 2092175 2092325

IV 2092504 2092692

IV 2092782 2092794

IV 2092951 2093009

IV 2094862 2094883

IV 2095067 2095096

IV 2095337 2095389

IV 2096309 2096312

IV 2096475 2096531

IV 2096856 2096881

IV 2097002 2097065

IV 2097722 2097970

IV 2098046 2098257

IV 2098331 2098693

IV 2098788 2098950

IV 2099014 2099253

IV 2101116 2101182

IV 2101323 2101331

IV 2101509 2101528

IV 2101686 2101745

IV 2110388 2110396

IV 2110476 2110504

IV 2113910 2113918

IV 2114059 2114129

IV 2114227 2114278

IV 2114382 2114409

IV 2117656 2117688

IV 2117774 2117886

IV 2118017 2118036

IV 2118099 2118101

IV 2118102 2118103

IV 2118309 2118357

IV 2118454 2118656

IV 2124799 2124887

IV 2127913 2128031

IV 2128319 2128329

IV 2135800 2135806

IV 2136185 2136223

IV 2136337 2136344

IV 2136558 2136567

IV 2139043 2139101

IV 2139230 2139279

IV 2139349 2139420

IV 2139637 2139662

IV 2139741 2139770

IV 2139887 2139977

IV 2140027 2140034

IV 2141270 2141276

IV 2142891 2142920

IV 2145492 2145499

IV 2145640 2145693

IV 2156602 2156640

IV 2156707 2156745

IV 2174051 2174158

IV 2183106 2183241

IV 2183331 2183454

IV 2183848 2183996

IV 2184090 2184219

IV 2187356 2187479

IV 2187562 2187732

IV 2187846 2187894

IV 2189277 2189285

IV 2190457 2190465

IV 2191037 2191048

IV 2194659 2194683

IV 2194795 2194802

IV 2196202 2196411

IV 2196550 2196760

IV 2200750 2200760

IV 2200974 2200984

IV 2203052 2203236

IV 2203321 2203331

IV 2203689 2203699

IV 2203785 2203970

IV 2206998 2207391

IV 2207565 2207577

IV 2207638 2207698

IV 2210565 2210585

IV 2210634 2210719

IV 2213542 2214082

IV 2215273 2215280

IV 2216515 2216575

IV 2216670 2216675

IV 2216976 2216981

IV 2222383 2222409

IV 2225493 2225576

IV 2226693 2226721

IV 2227601 2227681

IV 2230316 2230344

IV 2230439 2230467

IV 2231987 2232024

IV 2232116 2232395

IV 2232474 2232476

IV 2232720 2232763

IV 2232846 2232883

IV 2232978 2233258

IV 2234139 2234161

IV 2238797 2238815

IV 2239275 2239549

IV 2239826 2239864

IV 2240065 2240588

IV 2241545 2241617

IV 2242376 2242449

IV 2244677 2244693

IV 2244880 2244887

IV 2244970 2245095

IV 2245158 2245187

IV 2245267 2245333

IV 2245413 2245421

IV 2245513 2245582

IV 2245635 2245682

IV 2246855 2247376

IV 2247581 2247616

IV 2247890 2248164

IV 2248628 2248642

IV 2255294 2255326

IV 2255641 2255700

IV 2255940 2256832

IV 2257164 2257291

IV 2257569 2257587

IV 2258818 2258843

IV 2259083 2259088

IV 2259346 2259417

IV 2259636 2259652

IV 2259821 2259852

IV 2260117 2260130

IV 2260961 2260965

IV 2261177 2261187

IV 2261561 2261585

IV 2262258 2262309

IV 2264985 2265093

IV 2266589 2266599

IV 2266738 2266741

IV 2267224 2267274

IV 2268411 2268439

IV 2269014 2269021

IV 2272061 2272072

IV 2274650 2274665

IV 2275170 2275186

IV 2276443 2276467

IV 2277298 2277467

IV 2277660 2277687

IV 2277847 2277851

IV 2278186 2278272

IV 2278357 2278388

IV 2283037 2283088

IV 2285781 2285823

IV 2286593 2286596

IV 2288736 2288757

IV 2295043 2295078

IV 2295524 2295562

IV 2297362 2297534

IV 2297604 2297631

IV 2301694 2301853

IV 2301966 2302066

IV 2311368 2311391

IV 2311529 2311552

IV 2330005 2330008

IV 2330300 2330335

IV 2330571 2330884

IV 2331041 2331088

IV 2331215 2331354

IV 2331512 2331528

IV 2331608 2331876

IV 2331969 2332025

IV 2332112 2332176

IV 2332261 2332267

IV 2332680 2332756

IV 2332849 2333076

IV 2333140 2333271

IV 2333407 2333442

IV 2333535 2333609

IV 2334721 2334741

IV 2336416 2336456

IV 2338221 2338288

IV 2338966 2338996

IV 2339086 2339127

IV 2339197 2339234

IV 2339753 2339831

IV 2340346 2340355

IV 2340435 2340666

IV 2341910 2341940

IV 2342035 2342054

IV 2342185 2342195

IV 2342387 2342394

IV 2343442 2343468

IV 2343559 2343783

IV 2343877 2343880

IV 2345166 2345217

IV 2346228 2346262

IV 2346430 2346459

IV 2346554 2346716

IV 2346946 2346956

IV 2348540 2348552

IV 2350504 2350540

IV 2351790 2351810

IV 2352138 2352172

IV 2353364 2353372

IV 2353527 2353918

IV 2354363 2354725

IV 2359899 2359957

IV 2360060 2360111

IV 2360685 2360732

IV 2360828 2360887

IV 2360979 2361099

IV 2361150 2361206

IV 2361258 2361392

IV 2361530 2361633

IV 2362500 2362801

IV 2363956 2364197

IV 2364246 2364366

IV 2364460 2364667

IV 2365423 2365558

IV 2365764 2365981

IV 2366376 2366593

IV 2366828 2366964

IV 2367279 2367581

IV 2367880 2367954

IV 2368041 2368183

IV 2368371 2370151

IV 2370237 2371481

IV 2372465 2372492

IV 2372801 2372803

IV 2374343 2374398

IV 2375944 2375966

IV 2378527 2378579

IV 2379436 2379526

IV 2379595 2379662

IV 2381068 2381104

IV 2381861 2381909

IV 2394405 2395011

IV 2397338 2397348

IV 2397506 2398059

IV 2404711 2404809

IV 2404871 2405046

IV 2405137 2405239

IV 2405332 2405403

IV 2405500 2405573

IV 2405648 2405751

IV 2405846 2405939

IV 2406033 2406041

IV 2406107 2406843

IV 2406982 2407361

IV 2407411 2407494

IV 2407544 2407627

IV 2407678 2407715

IV 2411397 2411521

IV 2413079 2413087

IV 2418847 2418858

IV 2419121 2419151

IV 2419359 2419372

IV 2420271 2420322

IV 2421090 2421442

IV 2422854 2422944

IV 2423143 2423156

IV 2424170 2424180

IV 2424246 2424299

IV 2424390 2424476

IV 2427269 2427416

IV 2427720 2428103

IV 2431214 2431262

IV 2431356 2431423

IV 2431483 2431787

IV 2433896 2434140

IV 2434306 2434347

IV 2465861 2465888

IV 2467135 2467164

IV 2473744 2473859

IV 2473953 2474017

IV 2484263 2484374

IV 2484434 2484613

IV 2484791 2484944

IV 2485158 2486651

IV 2503162 2503334

IV 2508402 2508408

IV 2509657 2509677

IV 2511949 2511976

IV 2512038 2512062

IV 2513243 2513489

IV 2513564 2513570

IV 2513927 2514152

IV 2515477 2515519

IV 2515612 2516146

IV 2516766 2516830

IV 2516880 2516941

IV 2517072 2517150

IV 2517500 2517579

IV 2518508 2518525

IV 2519599 2519653

IV 2520024 2520088

IV 2526151 2526352

IV 2526402 2526494

IV 2558445 2558451

IV 2559545 2559552

IV 2561030 2561041

IV 2561223 2561281

IV 2564363 2564367

IV 2566181 2568264

IV 2568674 2570753

IV 2571798 2571814

IV 2573783 2573804

IV 2574070 2574346

IV 2574438 2574675

IV 2574727 2576016

IV 2578691 2578698

IV 2580382 2580450

IV 2584196 2584211

IV 2585255 2585298

IV 2585546 2585565

IV 2586088 2586120

IV 2586615 2586681

IV 2587161 2587194

IV 2587455 2587474

IV 2587750 2587769

IV 2588817 2588833

IV 2588922 2588972

IV 2589112 2589131

IV 2589225 2589293

IV 2589380 2589428

IV 2589520 2589526

IV 2589887 2589913

IV 2590006 2590021

IV 2590225 2590397

IV 2590491 2590546

IV 2590670 2590780

IV 2590871 2591154

IV 2591237 2591376

IV 2596346 2596459

IV 2601659 2601724

IV 2609025 2609183

IV 2609545 2609628

IV 2609702 2609728

IV 2617478 2617480

IV 2619448 2619452

IV 2625108 2625144

IV 2628646 2628686

IV 2633227 2633265

IV 2633479 2633497

IV 2633595 2633620

IV 2633722 2633797

IV 2633888 2633908

IV 2634065 2634407

IV 2634660 2634702

IV 2634786 2634791

IV 2634932 2634948

IV 2637748 2637900

IV 2638132 2638144

IV 2638208 2638266

IV 2638430 2638456

IV 2645854 2645913

IV 2646090 2646255

IV 2646347 2646419

IV 2647816 2648204

IV 2648269 2648309

IV 2648374 2648383

IV 2655913 2655985

IV 2661955 2661967

IV 2662385 2662396

IV 2671532 2671581

IV 2675018 2675109

IV 2675376 2675405

IV 2676106 2676206

IV 2682293 2682348

IV 2682529 2682576

IV 2683341 2683359

IV 2683729 2683782

IV 2684274 2684309

IV 2689707 2689708

IV 2690511 2690513

IV 2693992 2694025

IV 2694732 2694769

IV 2695045 2695092

IV 2695594 2695600

IV 2699085 2699161

IV 2699988 2700094

IV 2700181 2700445

IV 2700490 2700531

IV 2701121 2701178

IV 2701610 2701663

IV 2702074 2702081

IV 2702157 2702162

IV 2702237 2702288

IV 2702363 2702416

IV 2713814 2713862

IV 2713978 2713979

IV 2716303 2716339

IV 2716447 2716550

IV 2719506 2719556

IV 2719747 2719819

IV 2721392 2721499

IV 2721609 2721643

IV 2722341 2722415

IV 2722554 2722564

IV 2724053 2724070

IV 2725031 2725052

IV 2727042 2727156

IV 2727248 2727268

IV 2727427 2729245

IV 2732256 2734078

IV 2739042 2739270

IV 2739366 2739392

IV 2739612 2739961

IV 2740115 2740179

IV 2740334 2740551

IV 2744985 2745000

IV 2754868 2754989

IV 2755070 2755148

IV 2755234 2755361

IV 2755507 2755616

IV 2755778 2755939

IV 2756021 2756178

IV 2756260 2756300

IV 2756754 2756786

IV 2757002 2757072

IV 2759502 2759514

IV 2759660 2759681

IV 2760529 2760543

IV 2762718 2762733

IV 2762877 2762895

IV 2763414 2763423

IV 2767251 2767921

IV 2777584 2777644

IV 2777766 2777829

IV 2777917 2778155

IV 2780474 2780503

IV 2780674 2780714

IV 2780881 2781116

IV 2781207 2781220

IV 2784415 2784537

IV 2784599 2784614

IV 2784705 2784706

IV 2784760 2785101

IV 2787706 2787715

IV 2787765 2787775

IV 2787908 2787928

IV 2787990 2787999

IV 2788403 2788474

IV 2792548 2792660

IV 2792720 2792880

IV 2792948 2793252

IV 2795440 2795458

IV 2795516 2795545

IV 2801572 2801847

IV 2808129 2808137

IV 2810448 2810567

IV 2814895 2815006

IV 2820201 2820202

IV 2820204 2820212

IV 2820311 2820322

IV 2820408 2820416

IV 2823397 2823440

IV 2827908 2827952

IV 2828570 2828598

IV 2828800 2828816

IV 2828912 2828965

IV 2829090 2829914

IV 2829983 2830078

IV 2830142 2830439

IV 2830760 2830804

IV 2830946 2831069

IV 2831278 2831321

IV 2865576 2865588

IV 2871268 2871284

IV 2873488 2873673

IV 2873758 2873774

IV 2873857 2873905

IV 2874065 2874196

IV 2875547 2875683

IV 2875794 2875814

IV 2876074 2876095

IV 2876155 2876280

IV 2878289 2878451

IV 2878527 2878764

IV 2878837 2878875

IV 2878967 2878990

IV 2881088 2881091

IV 2883180 2883554

IV 2884195 2884216

IV 2884985 2884990

IV 2885048 2885068

IV 2894936 2895054

IV 2895222 2895300

IV 2895422 2895465

IV 2895563 2895715

IV 2895798 2895811

IV 2895909 2896014

IV 2896311 2896372

IV 2896990 2897018

IV 2897108 2897123

IV 2897313 2897337

IV 2899187 2899316

IV 2899513 2899683

IV 2899790 2899798

IV 2899920 2900088

IV 2900184 2900328

IV 2900386 2900543

IV 2900593 2900634

IV 2901042 2901051

IV 2901256 2901479

IV 2901599 2901637

IV 2901786 2901798

IV 2902273 2902577

IV 2902669 2902724

IV 2902816 2902936

IV 2903029 2903187

IV 2903284 2903474

IV 2903532 2903533

IV 2903714 2903735

IV 2903902 2904036

IV 2904123 2904761

IV 2904856 2905005

IV 2905172 2905457

IV 2905545 2906267

IV 2906364 2906574

IV 2906641 2906695

IV 2906789 2906866

IV 2906958 2907333

IV 2907421 2907434

IV 2907527 2907580

IV 2907672 2907887

IV 2908041 2908147

IV 2908243 2908253

IV 2908342 2908488

IV 2908792 2908807

IV 2908908 2909328

IV 2909478 2909630

IV 2909787 2909887

IV 2909980 2910019

IV 2910112 2910122

IV 2910281 2910892

IV 2910981 2911308

IV 2911449 2911492

IV 2911583 2911665

IV 2911769 2911850

IV 2911935 2911981

IV 2912053 2912171

IV 2912299 2912360

IV 2912542 2912610

IV 2912766 2913271

IV 2913417 2913456

IV 2913548 2913568

IV 2913830 2913946

IV 2916481 2916535

IV 2916711 2916804

IV 2916861 2916886

IV 2916972 2916984

IV 2917094 2917122

IV 2917363 2917387

IV 2917603 2917725

IV 2917809 2917878

IV 2917990 2917997

IV 2918080 2918142

IV 2930010 2930146

IV 2930234 2930410

IV 2930461 2930554

IV 2930633 2930658

IV 2934626 2934650

IV 2937343 2937407

IV 2940166 2940175

IV 2947701 2947708

IV 2947994 2948003

IV 2949567 2949590

IV 2949791 2949878

IV 2953293 2953333

IV 2953801 2953909

IV 2954000 2954013

IV 2954159 2954175

IV 2954276 2954296

IV 2954544 2954556

IV 2962147 2962153

IV 2975204 2975259

IV 2975568 2975608

IV 2989591 2989596

IV 2991431 2991434

IV 2995727 2996318

IV 2996412 2997008

IV 2999530 2999634

IV 3000820 3000952

IV 3001040 3001175

IV 3001225 3001332

IV 3001411 3001532

IV 3005120 3005121

IV 3005261 3005262

IV 3005265 3005275

IV 3006302 3006349

IV 3006700 3006720

IV 3012359 3012385

IV 3022852 3022876

IV 3030456 3030490

IV 3031148 3031303

IV 3050536 3050586

IV 3050654 3050840

IV 3053351 3053374

IV 3057453 3057671

IV 3057765 3057771

IV 3057937 3057975

IV 3058443 3058448

IV 3058542 3059061

IV 3059783 3060053

IV 3060151 3060293

IV 3060391 3060448

IV 3060545 3060624

IV 3064990 3065229

IV 3068848 3069088

IV 3069163 3069427

IV 3069545 3069551

IV 3070111 3070178

IV 3070850 3070915

IV 3073558 3073584

IV 3074287 3074403

IV 3074597 3074624

IV 3079729 3079769

IV 3079861 3079885

IV 3082263 3082297

IV 3091856 3092318

IV 3094522 3094713

IV 3094826 3094935

IV 3095144 3095286

IV 3096918 3096956

IV 3097806 3097836

IV 3097951 3097963

IV 3100992 3101008

IV 3101692 3101741

IV 3101819 3102136

IV 3102186 3102401

IV 3105498 3105706

IV 3105794 3105933

IV 3106050 3106099

IV 3109109 3109126

IV 3120160 3120171

IV 3125418 3125471

IV 3125543 3125634

IV 3125749 3125922

IV 3125999 3126057

IV 3126624 3126789

IV 3127146 3127322

IV 3130123 3130162

IV 3130718 3130754

IV 3135546 3135653

IV 3137194 3137563

IV 3137657 3140313

IV 3141647 3141868

IV 3141959 3141961

IV 3142127 3142168

IV 3142614 3142618

IV 3142714 3143232

IV 3143962 3144232

IV 3144329 3144473

IV 3144569 3144628

IV 3144722 3144804

IV 3144945 3145286

IV 3145390 3145430

IV 3147216 3147425

IV 3147542 3148153

IV 3149237 3149507

IV 3149604 3149666

IV 3149826 3149903

IV 3149992 3150003

IV 3150063 3150085

IV 3150142 3150393

IV 3150633 3150899

IV 3153662 3153836

IV 3153906 3153970

IV 3154021 3154062

IV 3154119 3154217

IV 3154337 3154372

IV 3157454 3157513

IV 3157964 3157982

IV 3158066 3158094

IV 3165867 3165873

IV 3166285 3166401

IV 3172061 3174720

IV 3174811 3175185

IV 3182202 3182219

IV 3183438 3183458

IV 3185406 3185450

IV 3193267 3193873

IV 3194102 3194151

IV 3206427 3206747

IV 3206879 3207381

IV 3207463 3208188

IV 3208282 3208548

IV 3208618 3208928

IV 3209060 3209404

IV 3209481 3209505

IV 3212620 3212657

IV 3212851 3213137

IV 3213200 3213309

IV 3218683 3218693

IV 3221563 3221605

IV 3221695 3221849

IV 3221943 3221982

IV 3222218 3222258

IV 3223965 3223982

IV 3224935 3224959

IV 3227009 3227032

IV 3227110 3227341

IV 3228607 3228659

IV 3235362 3235364

IV 3238276 3238314

IV 3238382 3238419

IV 3238514 3238562

IV 3238655 3238734

IV 3238825 3238884

IV 3238962 3238979

IV 3240682 3240688

IV 3241380 3241418

IV 3242218 3242256

IV 3242627 3242690

IV 3243595 3243773

IV 3243959 3244010

IV 3244080 3244107

IV 3244200 3244374

IV 3244543 3244554

IV 3248144 3248181

IV 3248325 3248606

IV 3248658 3248674

IV 3248780 3248846

IV 3250725 3250741

IV 3250845 3250983

IV 3252059 3252071

IV 3252122 3252134

IV 3254771 3254776

IV 3259692 3259767

IV 3262849 3262870

IV 3279404 3279413

IV 3279499 3279523

IV 3279608 3279609

IV 3281686 3281737

IV 3286896 3286987

IV 3287038 3287121

IV 3287181 3287204

IV 3289749 3289769

IV 3293136 3293144

IV 3293526 3293679

IV 3298154 3298185

IV 3300870 3302403

IV 3302461 3303997

IV 3304090 3304185

IV 3304291 3304317

IV 3306026 3306052

IV 3309699 3309700

IV 3309765 3309794

IV 3310154 3310157

IV 3310467 3310469

IV 3310566 3310593

IV 3313180 3313189

IV 3313475 3313528

IV 3313893 3314130

IV 3314220 3314376

IV 3314485 3314523

IV 3314612 3314617

IV 3314716 3314758

IV 3314927 3315298

IV 3315388 3315790

IV 3315864 3316253

IV 3316350 3316515

IV 3316690 3316743

IV 3316921 3317129

IV 3317379 3317557

IV 3317645 3318375

IV 3318463 3318493

IV 3318661 3318706

IV 3318795 3319000

IV 3319137 3319261

IV 3319436 3319902

IV 3320196 3320363

IV 3320647 3320788

IV 3321178 3321194

IV 3321316 3321331

IV 3321424 3321491

IV 3321651 3321669

IV 3321730 3321784

IV 3322054 3322105

IV 3322539 3322657

IV 3322742 3322864

IV 3322951 3323466

IV 3323539 3323859

IV 3323947 3324191

IV 3324244 3324444

IV 3324531 3324692

IV 3324784 3325006

IV 3325143 3325188

IV 3325398 3325420

IV 3325765 3325779

IV 3326038 3326189

IV 3326270 3326439

IV 3326974 3327122

IV 3327236 3327251

IV 3327462 3327466

IV 3327567 3327730

IV 3327919 3327921

IV 3328061 3328112

IV 3328207 3328211

IV 3328425 3328509

IV 3329134 3329149

IV 3329268 3329414

IV 3330061 3330066

IV 3330294 3330306

IV 3330401 3330427

IV 3330541 3330627

IV 3330728 3330782

IV 3330891 3330908

IV 3330997 3331022

IV 3331477 3331533

IV 3332116 3332122

IV 3332860 3332879

IV 3332940 3332983

IV 3333140 3333178

IV 3333257 3333506

IV 3333836 3333841

IV 3344249 3344268

IV 3352994 3353018

IV 3353139 3353173

IV 3353297 3353462

IV 3353649 3353724

IV 3353789 3353800

IV 3353852 3353919

IV 3354047 3354077

IV 3354301 3354348

IV 3356044 3356564

IV 3356837 3356890

IV 3357413 3357515

IV 3364929 3365444

IV 3365940 3366044

IV 3366277 3366281

IV 3366381 3366436

IV 3366529 3366604

IV 3366768 3366991

IV 3367047 3367105

IV 3367541 3367562

IV 3367764 3367789

IV 3367914 3368146

IV 3368472 3368490

IV 3380299 3380367

IV 3380443 3380446

IV 3387676 3387703

IV 3388022 3388091

IV 3388186 3388228

IV 3388462 3388578

IV 3388817 3388823

IV 3388933 3388980

IV 3389128 3389258

IV 3389356 3389388

IV 3389531 3389657

IV 3390015 3390084

IV 3390162 3390240

IV 3391313 3391402

IV 3391678 3391707

IV 3391961 3392013

IV 3392485 3392511

IV 3392695 3392736

IV 3394224 3394297

IV 3394389 3394522

IV 3431297 3431316

IV 3431366 3431376

IV 3431526 3431623

IV 3431776 3431781

IV 3432702 3432736

IV 3432940 3432964

IV 3433120 3433128

IV 3433220 3433235

IV 3433354 3433382

IV 3433627 3433703

IV 3433916 3433920

IV 3434017 3434116

IV 3434312 3434320

IV 3434411 3434416

IV 3434512 3434550

IV 3434667 3434715

IV 3434844 3434987

IV 3435352 3435375

IV 3449691 3449740

IV 3455454 3455544

IV 3457657 3457872

IV 3457957 3458174

IV 3485877 3485931

IV 3486012 3486317

IV 3489474 3489551

IV 3489600 3489866

IV 3490878 3490942

IV 3498522 3499737

IV 3499827 3499992

IV 3500122 3500280

IV 3500371 3500412

IV 3500508 3500926

IV 3501019 3501020

IV 3501106 3501232

IV 3501356 3501916

IV 3504645 3504657

IV 3529374 3529444

IV 3529533 3529596

IV 3529681 3529831

IV 3529890 3529956

IV 3530050 3530134

IV 3532384 3532387

IV 3534102 3534239

IV 3537850 3537990

IV 3538389 3538566

IV 3542339 3542479

IV 3542903 3543079

IV 3568089 3568210

IV 3568335 3568370

IV 3583136 3583294

IV 3583408 3583446

IV 3583532 3583707

IV 3589077 3589572

IV 3589771 3589786

IV 3589908 3590053

IV 3590131 3590551

IV 3619414 3619805

IV 3670664 3670772

IV 3670878 3670912

IV 3671014 3671049

IV 3671109 3671123

IV 3671281 3671318

IV 3671377 3671608

IV 3671663 3671972

IV 3672054 3672293

IV 3678545 3678569

IV 3696473 3696486

IV 3696645 3696686

IV 3718484 3718516

IV 3720363 3720375

IV 3720442 3720525

IV 3721122 3721416

IV 3721465 3721576

IV 3721623 3721797

IV 3727577 3727801

IV 3729394 3729747

IV 3729977 3730104

IV 3737477 3737562

IV 3740437 3740516

IV 3771246 3771323

IV 3771493 3771510

IV 3789134 3789174

IV 3789452 3789653

IV 3789864 3789904

IV 3790554 3790751

IV 3801292 3801426

IV 3817851 3817875

IV 3818188 3818198

IV 3818445 3818457

IV 3818644 3818696

IV 3825869 3825945

IV 3834116 3834125

IV 3834216 3834320

IV 3879457 3879534

IV 3879816 3879852

IV 3882077 3882090

IV 3885939 3885953

IV 3897718 3897752

IV 3901371 3901495

IV 3913319 3913394

IV 3913661 3913702

IV 3923733 3923760

IV 3929395 3929613

IV 3930958 3930983

IV 3932446 3932665

IV 3933151 3933185

IV 3940408 3940426

IV 3940478 3941089

IV 3950738 3950756

IV 3952636 3952651

IV 3962639 3962656

IV 3963097 3963098

IV 3963099 3963101

IV 3963303 3963311

IV 3970446 3970480

IV 4014429 4014456

IV 4023529 4023657

IV 4041916 4042046

IV 4058709 4058739

IV 4059486 4059515

IV 4061436 4061449

IV 4064827 4064837

IV 4069960 4069997

IV 4076121 4076140

IV 4076396 4076418

IV 4081739 4081791

IV 4087091 4087139

IV 4087246 4087611

IV 4087946 4088307

IV 4088416 4088464

IV 4092515 4092549

IV 4092632 4092924

IV 4093065 4093104

IV 4099317 4099469

IV 4114882 4114932

IV 4115991 4116045

IV 4117007 4117124

IV 4120653 4120657

IV 4124113 4124147

IV 4125919 4126450

IV 4126726 4126784

IV 4127052 4127055

IV 4127119 4127595

IV 4127653 4127856

IV 4127946 4128140

IV 4128222 4128302

IV 4129942 4130045

IV 4130138 4130280

IV 4135340 4135441

IV 4135535 4135680

IV 4142646 4142706

IV 4143287 4143399

IV 4143710 4143921

IV 4144103 4144137

IV 4144191 4144223

IV 4144426 4144452

IV 4145574 4145611

IV 4150827 4150877

IV 4151358 4151376

IV 4157879 4157931

IV 4158423 4158453

IV 4167110 4167177

IV 4170162 4170691

IV 4170809 4170925

IV 4180270 4184931

IV 4185022 4185124

IV 4185221 4185252

IV 4185337 4185382

IV 4185473 4185637

IV 4186002 4186004

IV 4186091 4186235

IV 4186324 4186527

IV 4186700 4187390

IV 4187473 4187535

IV 4187625 4187926

IV 4188482 4188521

IV 4188726 4188771

IV 4188884 4188905

IV 4189161 4189257

IV 4189353 4189625

IV 4189782 4189792

IV 4189909 4190260

IV 4190400 4190529

IV 4190683 4190708

IV 4190890 4190967

IV 4191198 4191329

IV 4191424 4191461

IV 4191615 4191693

IV 4191787 4191843

IV 4191968 4192291

IV 4192384 4192912

IV 4193032 4193652

IV 4193746 4194003

IV 4194095 4194169

IV 4194253 4194357

IV 4194453 4194457

IV 4195040 4195164

IV 4195259 4195294

IV 4195415 4195424

IV 4195551 4195714

IV 4195859 4195881

IV 4196072 4196129

IV 4196332 4196458

IV 4196613 4196781

IV 4196890 4196951

IV 4197135 4197155

IV 4197264 4197300

IV 4197398 4197403

IV 4197521 4197589

IV 4197804 4197904

IV 4198083 4198090

IV 4198473 4198518

IV 4198584 4198599

IV 4200354 4200402

IV 4220487 4221643

IV 4224061 4224072

IV 4225218 4225238

IV 4225328 4225330

IV 4225421 4225499

IV 4225591 4225655

IV 4227071 4227082

IV 4228869 4228892

IV 4239808 4239820

IV 4240233 4240383

IV 4240570 4240586

IV 4259222 4259299

IV 4278635 4278703

IV 4304345 4304472

IV 4304565 4304717

IV 4304800 4305081

IV 4305186 4305445

IV 4305743 4306010

IV 4307865 4307971

IV 4308134 4308159

IV 4308255 4308287

IV 4308559 4308582

IV 4308674 4308685

IV 4309198 4309249

IV 4309467 4309512

IV 4309564 4309591

IV 4309683 4309691

IV 4310408 4310492

IV 4311271 4311277

IV 4312138 4312139

IV 4312233 4312374

IV 4312765 4312794

IV 4313376 4313381

IV 4314120 4314192

IV 4314294 4314320

IV 4314490 4314529

IV 4315082 4315106

IV 4316135 4316235

IV 4317477 4317530

IV 4318753 4318775

IV 4318945 4318964

IV 4319216 4319246

IV 4319880 4319994

IV 4320112 4320131

IV 4320332 4320469

IV 4320650 4320702

IV 4320847 4320867

IV 4321722 4321780

IV 4322150 4322176

IV 4322702 4322743

IV 4322854 4322890

IV 4324788 4325370

IV 4325542 4325692

IV 4325786 4325913

IV 4332151 4332184

IV 4335469 4335629

IV 4336752 4336789

IV 4348232 4352004

IV 4352461 4356234

IV 4358361 4358447

IV 4366903 4366911

IV 4367053 4367060

IV 4376885 4376921

IV 4377217 4377229

IV 4377394 4377596

IV 4377746 4377825

IV 4377927 4377942

IV 4378799 4378817

IV 4378913 4378925

IV 4379119 4379153

IV 4379850 4379882

IV 4380128 4380426

IV 4380565 4380574

IV 4380774 4380791

IV 4380997 4381072

IV 4381241 4381248

IV 4381403 4381494

IV 4381783 4381818

IV 4382070 4382269

IV 4382367 4382373

IV 4383304 4383325

IV 4386837 4386840

IV 4387316 4387320

IV 4387658 4387724

IV 4387938 4387999

IV 4388271 4388280

IV 4388390 4388478

IV 4388718 4388756

IV 4389244 4389262

IV 4389501 4389511

IV 4390119 4390175

IV 4390416 4390453

IV 4391228 4391267

IV 4391660 4391682

IV 4401698 4401773

IV 4401862 4401968

IV 4416262 4416361

IV 4416514 4416639

IV 4416700 4416814

IV 4416934 4417349

IV 4417454 4417476

IV 4417532 4417687

IV 4417740 4417818

IV 4417871 4418078

IV 4418156 4418427

IV 4418485 4418525

IV 4418627 4419150

IV 4419211 4419930

IV 4420001 4420714

IV 4420809 4421221

IV 4421291 4421508

IV 4421560 4421865

IV 4421953 4421984

IV 4433734 4433746

IV 4437668 4437687

IV 4465823 4465971

IV 4474507 4474675

IV 4475205 4475375

IV 4500457 4500619

IV 4500679 4501128

IV 4501216 4501564

IV 4501630 4501972

IV 4507314 4507345

IV 4507455 4507545

IV 4507599 4507746

IV 4507945 4507973

IV 4508032 4508181

IV 4554347 4554849

IV 4555040 4555108

IV 4555165 4555168

IV 4559825 4562241

IV 4591787 4591815

IV 4592284 4592466

IV 4594789 4594827

IV 4598015 4598043

IV 4598534 4598714

IV 4600820 4600836

IV 4617597 4617696

IV 4617786 4617806

IV 4658812 4658927

IV 4674989 4674995

IV 4675139 4675145

IV 4702125 4702411

IV 4713091 4713136

IV 4713623 4713806

IV 4715081 4715218

IV 4716027 4720688

IV 4721476 4721597

IV 4721790 4721863

IV 4721962 4722237

IV 4722362 4722624

IV 4722760 4722829

IV 4722890 4722964

IV 4725595 4725660

IV 4725754 4725777

IV 4726128 4726143

IV 4726304 4726343

IV 4726611 4726624

IV 4726979 4726995

IV 4727074 4727231

IV 4727354 4727382

IV 4727440 4727448

IV 4727878 4727893

IV 4728069 4728165

IV 4729294 4729340

IV 4753486 4753529

IV 4753779 4753817

IV 4753872 4753953

IV 4754020 4754174

IV 4754264 4754303

IV 4763260 4763414

IV 4770024 4770060

IV 4771320 4771374

IV 4783042 4783117

IV 4783199 4783407

IV 4784882 4784943

IV 4804734 4804773

IV 4816243 4816272

IV 4816348 4816351

IV 4822642 4822768

IV 4823253 4823933

IV 4824272 4825101

IV 4843612 4843882

IV 4857783 4857789

IV 4860817 4860829

IV 4864665 4864696

IV 4866851 4866879

IV 4868657 4868684

IV 4868924 4868961

IV 4869089 4869242

IV 4870227 4870364

IV 4870474 4870521

IV 4872438 4872473

IV 4872603 4872758

IV 4874284 4874421

IV 4874530 4874577

IV 4881209 4881414

IV 4881501 4881665

IV 4881834 4882303

IV 4882399 4882505

IV 4882597 4882728

IV 4885583 4885609

IV 4897687 4897794

IV 4897928 4897987

IV 4898083 4898419

IV 4898504 4898750

IV 4899563 4899651

IV 4899723 4899824

IV 4899914 4899966

IV 4900255 4900697

IV 4914427 4914625

IV 4917129 4917323

IV 4920790 4920835

IV 4920929 4921009

IV 4921091 4925879

IV 4925977 4928067

IV 4930994 4931019

IV 4943823 4943890

IV 4947993 4948058

IV 4948872 4949031

IV 4949244 4949339

IV 4949407 4949653

IV 4949772 4949792

IV 4949862 4949871

IV 4950038 4950050

IV 4976512 4976527

IV 4994412 4994706

IV 4999527 4999819

IV 5003530 5003531

IV 5003817 5003877

IV 5011188 5011292

IV 5015872 5015894

IV 5016548 5016558

IV 5016940 5016984

IV 5017501 5017529

IV 5022776 5022875

IV 5023280 5023602

IV 5035413 5035736

IV 5055696 5055727

IV 5055814 5055886

IV 5055985 5056032

IV 5060117 5060197

IV 5062577 5062639

IV 5062697 5062776

IV 5062874 5062956

IV 5064655 5064910

IV 5065004 5065060

IV 5065153 5065177

IV 5065438 5065454

IV 5065594 5065783

IV 5065898 5065966

IV 5066060 5066095

IV 5066192 5066323

IV 5066418 5068307

IV 5068404 5069333

IV 5069449 5069878

IV 5069974 5070313

IV 5070409 5070489

IV 5070857 5071306

IV 5071465 5072320

IV 5072420 5072477

IV 5072569 5072603

IV 5072790 5072791

IV 5072933 5073137

IV 5073375 5073499

IV 5073746 5073792

IV 5073942 5073955

IV 5074283 5074299

IV 5089933 5090135

IV 5162922 5163060

IV 5165607 5165744

IV 5182593 5182637

IV 5191705 5191708

IV 5217648 5219921

IV 5222746 5222837

IV 5225704 5227982

IV 5231405 5235062

IV 5235331 5238988

IV 5239080 5239672

IV 5239760 5241182

IV 5241267 5245477

IV 5245747 5249952

IV 5250041 5251462

IV 5251548 5252279

IV 5252518 5252725

IV 5253050 5253082

IV 5253186 5253245

IV 5253348 5254204

IV 5254363 5254809

IV 5255177 5255255

IV 5255348 5255692

IV 5255782 5256217

IV 5256344 5257268

IV 5257363 5259254

IV 5259349 5259479

IV 5259574 5259612

IV 5259707 5259841

IV 5259890 5260076

IV 5260192 5260207

IV 5260471 5260600

IV 5260712 5260964

IV 5261166 5261214

IV 5265438 5265491

IV 5265643 5265842

IV 5265916 5266019

IV 5294857 5295077

IV 5295138 5295429

IV 5297977 5298306

IV 5298357 5298391

IV 5299244 5299321

IV 5299371 5299394

IV 5299659 5299691

IV 5299766 5299819

IV 5299890 5299997

IV 5300087 5300130

IV 5300222 5300284

IV 5300374 5301147

IV 5301402 5301420

IV 5304158 5304168

IV 5304276 5304279

IV 5306731 5306757

IV 5306949 5307024

IV 5307163 5307202

IV 5307476 5307605

IV 5308536 5308641

IV 5308703 5308894

IV 5309196 5309431

IV 5310038 5310043

IV 5312769 5313012

IV 5313084 5313191

IV 5316296 5316310

IV 5316450 5316555

IV 5316675 5316869

IV 5317751 5317773

IV 5338704 5338776

IV 5339017 5339053

IV 5349161 5349750

IV 5349991 5349997

IV 5350085 5350342

IV 5350445 5350663

IV 5383360 5383368

IV 5383660 5383662

IV 5383754 5383802

IV 5384198 5384252

IV 5384951 5385078

IV 5386318 5386441

IV 5399045 5399049

IV 5408513 5408564

IV 5431100 5431159

IV 5435101 5435248

IV 5454922 5454987

IV 5455119 5455176

IV 5455415 5455492

IV 5455587 5455706

IV 5456079 5456200

IV 5456294 5456368

IV 5457329 5457449

IV 5457573 5457594

IV 5457686 5457705

IV 5461632 5461633

IV 5461804 5461927

IV 5464699 5464747

IV 5465091 5466121

IV 5466190 5466576

IV 5466667 5466760

IV 5466824 5466895

IV 5466951 5467117

IV 5483088 5483093

IV 5483270 5483387

IV 5483542 5483572

IV 5483748 5483807

IV 5483928 5483967

IV 5484244 5484364

IV 5484556 5484575

IV 5484862 5484996

IV 5485162 5485164

IV 5485342 5485511

IV 5485687 5486067

IV 5486335 5486343

IV 5486434 5486435

IV 5486437 5486444

IV 5486781 5486823

IV 5486913 5486982

IV 5487089 5487145

IV 5487246 5487409

IV 5487570 5487595

IV 5487763 5487789

IV 5487991 5488556

IV 5489459 5489530

IV 5489625 5489637

IV 5489786 5489847

IV 5489936 5489973

IV 5490061 5490133

IV 5490225 5490237

IV 5490505 5490799

IV 5490887 5490954

IV 5491301 5491307

IV 5491399 5491486

IV 5509966 5510334

IV 5510421 5510524

IV 5510625 5511357

IV 5511437 5511566

IV 5522579 5522641

IV 5555031 5556437

IV 5556533 5557937

IV 5571168 5571217

IV 5571291 5571292

IV 5571386 5571555

IV 5571828 5571888

IV 5571979 5572159

IV 5572244 5572248

IV 5579640 5579777

IV 5579880 5579931

IV 5580021 5580031

IV 5580168 5580237

IV 5611485 5611489

IV 5611732 5611763

IV 5611944 5611973

IV 5676682 5676734

IV 5677060 5677078

IV 5677464 5677477

IV 5677572 5677591

IV 5691324 5691337

IV 5691399 5691419

IV 5693741 5693742

IV 5693743 5693745

IV 5695157 5695194

IV 5733438 5733491

IV 5733651 5733672

IV 5745652 5745666

IV 5772003 5772036

IV 5772134 5772289

IV 5772472 5772875

IV 5772972 5773061

IV 5773142 5773291

IV 5773377 5774068

IV 5774149 5774631

IV 5774704 5775091

IV 5793650 5793680

IV 5793981 5794325

IV 5837628 5837635

IV 5844782 5845338

IV 5845433 5845675

IV 5849241 5849489

IV 5849580 5850117

IV 5850445 5850478

IV 5868537 5868756

IV 5868808 5868887

IV 5884148 5884205

IV 5884290 5884448

IV 5901682 5901690

IV 5901827 5901920

IV 5965033 5965116

IV 5994107 5994175

IV 5995938 5996009

IV 5997416 5997481

IV 5999878 5999943

IV 6000486 6000497

IV 6005407 6005589

IV 6005726 6005747

IV 6009171 6009347

IV 6012579 6012757

IV 6012895 6012924

IV 6033719 6033791

IV 6033882 6033922

IV 6039511 6039728

IV 6039780 6039862

IV 6077347 6077365

IV 6077940 6078009

IV 6081537 6081556

IV 6082108 6082174

IV 6084599 6084655

IV 6085410 6085466

IV 6086926 6086959

IV 6087994 6088023

IV 6090651 6090663

IV 6094213 6094328

IV 6094722 6094751

IV 6107022 6107090

IV 6140345 6140459

IV 6141396 6141681

IV 6141831 6142399

IV 6142461 6142560

IV 6192017 6192119

IV 6192196 6192216

IV 6192269 6192483

IV 6192721 6192848

IV 6192914 6192988

IV 6193147 6193249

IV 6210136 6210138

IV 6218349 6218415

IV 6218959 6218989

IV 6220444 6220550

IV 6230048 6230058

IV 6234015 6234269

IV 6234352 6234518

IV 6234588 6234635

IV 6234685 6234889

IV 6250293 6250403

IV 6250499 6250525

IV 6250704 6251267

IV 6251554 6251679

IV 6251774 6251782

IV 6251881 6251976

IV 6252135 6252246

IV 6252479 6252583

IV 6252676 6252691

IV 6252869 6252985

IV 6253255 6253482

IV 6253723 6253950

IV 6254227 6254324

IV 6254552 6254607

IV 6257274 6257447

IV 6257533 6258014

IV 6258097 6258294

IV 6258384 6258547

IV 6269097 6269142

IV 6285354 6285377

IV 6285474 6285696

IV 6285783 6285960

IV 6286269 6286558

IV 6286706 6286990

IV 6287081 6287155

IV 6287300 6287477

IV 6287563 6287632

IV 6287721 6287789

IV 6287898 6287937

IV 6307270 6307389

IV 6308004 6308115

IV 6311366 6311410

IV 6311762 6311769

IV 6311910 6311934

IV 6312029 6312084

IV 6312181 6312257

IV 6312404 6312460

IV 6312543 6312643

IV 6312797 6312888

IV 6312980 6313145

IV 6313230 6313911

IV 6313999 6314174

IV 6314264 6314350

IV 6315549 6315560

IV 6315832 6315900

IV 6317437 6317440

IV 6317602 6317613

IV 6317852 6318120

IV 6318215 6318301

IV 6318394 6318404

IV 6318576 6319013

IV 6319170 6319366

IV 6319485 6319562

IV 6319679 6319705

IV 6319807 6320112

IV 6320531 6320549

IV 6320613 6320749

IV 6340521 6340538

IV 6343298 6343300

IV 6343701 6344123

IV 6344242 6344281

IV 6344371 6344445

IV 6345183 6345229

IV 6345638 6345661

IV 6345801 6345824

IV 6345961 6345972

IV 6346197 6346265

IV 6346421 6346452

IV 6346960 6346972

IV 6347088 6347144

IV 6347408 6347500

IV 6348043 6348106

IV 6348218 6348371

IV 6348461 6348482

IV 6348800 6349000

IV 6349091 6349158

IV 6349246 6349323

IV 6349393 6349452

IV 6349699 6349711

IV 6349805 6349993

IV 6350243 6350274

IV 6350536 6350635

IV 6350824 6350889

IV 6350940 6351212

IV 6351335 6351372

IV 6351490 6351665

IV 6351747 6351881

IV 6352121 6352206

IV 6352279 6352332

IV 6352415 6352540

IV 6352669 6352677

IV 6355236 6355310

IV 6355395 6355441

IV 6355579 6355773

IV 6355859 6355876

IV 6355927 6355947

IV 6356041 6356271

IV 6356364 6356380

IV 6357890 6358242

IV 6358383 6358526

IV 6358649 6358918

IV 6358975 6359021

IV 6359119 6359368

IV 6359572 6359805

IV 6359874 6360119

IV 6360174 6360226

IV 6360280 6360298

IV 6360348 6360361

IV 6360551 6360618

IV 6360675 6360862

IV 6360953 6361069

IV 6406987 6407070

IV 6419041 6419042

IV 6423674 6423851

IV 6423934 6424238

IV 6461400 6461711

IV 6462422 6462737

IV 6468714 6468785

IV 6469045 6469177

IV 6469234 6469745

IV 6482833 6483180

IV 6483248 6483566

IV 6483696 6483793

IV 6501338 6501609

IV 6515538 6516098

IV 6516158 6516225

IV 6516296 6516518

IV 6516609 6516620

IV 6519156 6519167

IV 6526914 6527086

IV 6538208 6538235

IV 6539148 6539326

IV 6551592 6551768

IV 6590321 6590328

IV 6590438 6590485

IV 6590648 6591080

IV 6591503 6591863

IV 6591956 6591975

IV 6594698 6594714

IV 6594808 6595170

IV 6598293 6598351

IV 6598447 6598678

IV 6598841 6598885

IV 6598995 6599004

IV 6599186 6599226

IV 6599317 6599372

IV 6609280 6609353

IV 6609541 6609561

IV 6609647 6609650

IV 6609742 6609766

IV 6609860 6609877

IV 6610063 6610133

IV 6610229 6610250

IV 6610409 6610431

IV 6611462 6611503

IV 6611750 6611844

IV 6616582 6616624

IV 6616864 6616959

IV 6665850 6665860

IV 6665922 6665957

IV 6666049 6666072

IV 6668374 6668516

IV 6668618 6669159

IV 6669209 6669428

IV 6669486 6669748

IV 6669838 6669934

IV 6669999 6670002

IV 6670074 6670352

IV 6670408 6670787

IV 6670856 6671044

IV 6671109 6671216

IV 6671297 6671829

IV 6672011 6672089

IV 6672182 6672249

IV 6672338 6672628

IV 6672705 6673062

IV 6673122 6673237

IV 6673325 6673578

IV 6673653 6674545

IV 6674630 6674781

IV 6674847 6675660

IV 6675752 6675826

IV 6676078 6676209

IV 6676300 6676349

IV 6676517 6676520

IV 6682810 6683452

IV 6683516 6684224

IV 6684317 6684510

IV 6684560 6685360

IV 6685418 6685518

IV 6685577 6685703

IV 6685759 6685876

IV 6685974 6686063

IV 6686156 6686174

IV 6686323 6686378

IV 6686569 6686667

IV 6686744 6686853

IV 6686937 6687132

IV 6687264 6687281

IV 6687356 6687488

IV 6687629 6687667

IV 6687817 6687853

IV 6688010 6688023

IV 6688093 6688719

IV 6688827 6688845

IV 6688933 6689635

IV 6689727 6690752

IV 6690816 6691112

IV 6691193 6691217

IV 6691278 6691537

IV 6691621 6691829

IV 6691912 6692642

IV 6692730 6693936

IV 6694030 6696389

IV 6696454 6697663

IV 6697751 6697976

IV 6698067 6699165

IV 6699296 6699754

IV 6699934 6700721

IV 6700804 6701363

IV 6701415 6701501

IV 6701588 6703920

IV 6703982 6704144

IV 6704242 6704265

IV 6704347 6704445

IV 6704528 6704681

IV 6707831 6707907

IV 6708243 6708250

IV 6708442 6708444

IV 6708710 6709128

IV 6709173 6709202

IV 6709280 6709294

IV 6709380 6709599

IV 6709748 6709774

IV 6709853 6710102

IV 6710218 6710449

IV 6710525 6710695

IV 6710804 6711037

IV 6711129 6711333

IV 6711422 6712454

IV 6712533 6713553

IV 6713632 6714160

IV 6714261 6715248

IV 6715319 6716165

IV 6716237 6716399

IV 6716495 6716507

IV 6716580 6716601

IV 6716692 6716752

IV 6716829 6717413

IV 6717483 6719369

IV 6719449 6719494

IV 6719553 6721236

IV 6721300 6721360

IV 6721445 6721892

IV 6721941 6722182

IV 6722245 6722528

IV 6722735 6722964

IV 6723027 6723790

IV 6723914 6724681

IV 6724741 6724864

IV 6724929 6725110

IV 6725207 6725277

IV 6725338 6725829

IV 6725949 6726040

IV 6726123 6727033

IV 6727079 6728904

IV 6729080 6729211

IV 6729264 6729351

IV 6729477 6729549

IV 6729615 6729701

IV 6729826 6729901

IV 6729951 6730167

IV 6730257 6730470

IV 6730600 6730639

IV 6730733 6730925

IV 6731066 6731173

IV 6731315 6731384

IV 6731681 6731882

IV 6732584 6732591

IV 6732680 6732690

IV 6737870 6737919

IV 6774893 6775036

IV 6786331 6786396

IV 6822992 6823037

IV 6823109 6824091

IV 6824941 6824971

IV 6827308 6827367

IV 6827524 6827565

IV 6827823 6827856

IV 6861323 6861483

IV 6865633 6865651

IV 6865742 6865774

IV 6865870 6865987

IV 6866172 6866196

IV 6866280 6866325

IV 6866532 6866542

IV 6884390 6884404

IV 6884488 6884546

IV 6884599 6884747

IV 6884797 6885255

IV 6898326 6898378

IV 6901482 6901673

IV 6901740 6901742

IV 6901946 6901984

IV 6902116 6902119

IV 6914364 6914399

IV 6914597 6914634

IV 6922027 6922077

IV 6922254 6922477

IV 6922676 6922789

IV 6939735 6939792

IV 6947658 6947788

IV 6947880 6948065

IV 6948157 6948158

IV 6948250 6948610

IV 6948722 6948920

IV 6949131 6949399

IV 6949484 6950132

IV 6950246 6950258

IV 6951966 6951985

IV 6973830 6973832

IV 6974011 6974100

IV 6974147 6974180

IV 6981018 6981124

IV 6981213 6981226

IV 6981375 6981591

IV 6981720 6981828

IV 6981922 6982245

IV 6982341 6982451

IV 7041199 7041216

IV 7041796 7041835

IV 7042481 7042602

IV 7056923 7056956

IV 7057074 7057251

IV 7068217 7068260

IV 7068373 7068692

IV 7068797 7068863

IV 7069007 7069098

IV 7069245 7069297

IV 7069438 7069540

IV 7069633 7069729

IV 7070036 7070374

IV 7071010 7071109

IV 7071621 7071628

IV 7072517 7072523

IV 7073371 7073470

IV 7111049 7111076

IV 7111674 7111685

IV 7112066 7112107

IV 7112625 7112655

IV 7125204 7125241

IV 7125365 7125406

IV 7127110 7127137

IV 7127284 7127352

IV 7128178 7128185

IV 7128379 7128560

IV 7128739 7128939

IV 7129029 7129303

IV 7129425 7129735

IV 7129849 7129851

IV 7130124 7130201

IV 7130296 7130315

IV 7145560 7145574

IV 7146858 7146923

IV 7146984 7147007

IV 7147188 7147216

IV 7239965 7240098

IV 7247327 7247383

IV 7248477 7248561

IV 7251338 7251444

IV 7251492 7251501

IV 7252085 7252151

IV 7252324 7252406

IV 7252535 7252770

IV 7254390 7254556

IV 7254645 7254926

IV 7255770 7255774

IV 7256117 7256121

IV 7277124 7277234

IV 7277325 7277341

IV 7277409 7277457

IV 7277575 7277628

IV 7277725 7277848

IV 7278089 7278200

IV 7280193 7280276

IV 7344391 7344761

IV 7344852 7345238

IV 7345380 7345541

IV 7345603 7345784

IV 7356870 7359924

IV 7360821 7363880

IV 7363967 7366677

IV 7366771 7369743

IV 7370511 7373487

IV 7373581 7376289

IV 7380025 7380062

IV 7380149 7380197

IV 7380317 7380355

IV 7380586 7380652

IV 7380855 7380901

IV 7381168 7381210

IV 7381375 7381497

IV 7389434 7389533

IV 7389658 7389663

IV 7441085 7441523

IV 7441597 7441637

IV 7441867 7441988

IV 7461065 7461107

IV 7464727 7464732

IV 7464830 7464859

IV 7486858 7486861

IV 7524390 7525550

IV 7527851 7527890

IV 7528027 7528047

IV 7548217 7548361

IV 7563197 7563234

IV 7564118 7564193

IV 7564262 7564318

IV 7564396 7564568

IV 7564659 7564782

IV 7564851 7564857

IV 7564954 7565456

IV 7565506 7566510

IV 7566574 7566705

IV 7566800 7566927

IV 7567001 7567060

IV 7567141 7567439

IV 7591477 7591560

IV 7591684 7591700

IV 7591814 7591853

IV 7591987 7592018

IV 7592068 7592254

IV 7592347 7592379

IV 7592433 7592695

IV 7592765 7592831

IV 7592956 7593042

IV 7593196 7593263

IV 7593363 7593836

IV 7593915 7594122

IV 7594174 7594460

IV 7594535 7594565

IV 7594691 7594849

IV 7594940 7595041

IV 7595109 7595451

IV 7595545 7595640

IV 7595751 7595790

IV 7595981 7596098

IV 7596195 7596218

IV 7596317 7596378

IV 7596686 7596845

IV 7596938 7597161

IV 7597243 7597347

IV 7597397 7598248

IV 7598329 7598576

IV 7598785 7598891

IV 7599045 7599259

IV 7599332 7599724

IV 7601156 7601158

IV 7601426 7601807

IV 7602139 7602220

IV 7603638 7603754

IV 7603904 7604027

IV 7607070 7607082

IV 7607538 7607544

IV 7609607 7609617

IV 7609877 7610257

IV 7625750 7625766

IV 7625862 7625891

IV 7626140 7626327

IV 7634115 7634172

IV 7634327 7634392

IV 7634583 7634587

IV 7634747 7634778

IV 7635410 7635418

IV 7635537 7635540

IV 7635750 7635751

IV 7649498 7649543

IV 7683407 7683608

IV 7683804 7683910

IV 7683990 7684043

IV 7684095 7684101

IV 7684257 7684335

IV 7684727 7684953

IV 7685036 7685041

IV 7685206 7685210

IV 7685427 7685508

IV 7699188 7699365

IV 7699459 7699953

IV 7702342 7702429

IV 7702648 7702994

IV 7703081 7703525

IV 7735233 7735376

IV 7735431 7735461

IV 7735522 7736079

IV 7798172 7798186

IV 7798759 7798800

IV 7799437 7799552

IV 7803588 7803752

IV 7803834 7804024

IV 7804119 7804185

IV 7804241 7804282

IV 7810921 7811059

IV 7872195 7872199

IV 7874261 7874291

IV 7874401 7874885

IV 7874969 7875279

IV 7875487 7875511

IV 7920689 7920730

IV 7920802 7920958

IV 7922408 7922473

IV 7947156 7947260

IV 7947357 7947374

IV 7947584 7947665

IV 7947885 7947923

IV 7948012 7948061

IV 7948307 7948326

IV 7948453 7948492

IV 7967546 7967633

IV 7967781 7967783

IV 7968264 7968269

IV 7968361 7968382

IV 7968458 7968555

IV 7968654 7968941

IV 7969043 7969125

IV 7969185 7969280

IV 7969423 7969452

IV 7974432 7974580

IV 7974648 7974679

IV 7974767 7974991

IV 7975080 7975081

IV 7985039 7985178

IV 7998604 7998755

IV 8004537 8004541

IV 8004646 8004747

IV 8015885 8015937

IV 8015988 8016192

IV 8039005 8039013

IV 8072032 8072225

IV 8072302 8072318

IV 8072403 8072844

IV 8072908 8072997

IV 8073107 8073135

IV 8073239 8073350

IV 8073500 8073556

IV 8073650 8073662

IV 8073755 8073800

IV 8073897 8073948

IV 8074046 8074066

IV 8089633 8089754

IV 8089850 8090001

IV 8090091 8090373

IV 8090443 8091129

IV 8097268 8097348

IV 8097435 8097575

IV 8097697 8098085

IV 8098180 8098198

IV 8122564 8123065

IV 8123118 8123127

IV 8123302 8123651

IV 8123776 8123790

IV 8123956 8123976

IV 8223604 8224238

IV 8224299 8224958

IV 8225086 8225184

IV 8225266 8225289

IV 8246116 8246129

IV 8255681 8256086

IV 8256150 8256304

IV 8256384 8256872

IV 8256962 8256985

IV 8257174 8257326

IV 8257458 8257902

IV 8257993 8258060

IV 8258172 8258327

IV 8258416 8258886

IV 8269480 8269492

IV 8269570 8269672

IV 8269759 8269931

IV 8269991 8270072

IV 8270164 8270299

IV 8324978 8325044

IV 8325184 8325186

IV 8325520 8325526

IV 8330712 8332244

IV 8335144 8336670

IV 8337104 8337109

IV 8337862 8337880

IV 8338008 8338086

IV 8338435 8338494

IV 8339406 8339422

IV 8339626 8339631

IV 8349529 8349592

IV 8349674 8350353

IV 8350698 8350853

IV 8351157 8351889

IV 8351938 8352085

IV 8373758 8373767

IV 8373816 8373842

IV 8373936 8374152

IV 8374631 8374797

IV 8374966 8374981

IV 8389023 8389123

IV 8389262 8389322

IV 8389416 8389418

IV 8389606 8389625

IV 8389763 8392078

IV 8392173 8392283

IV 8392501 8393262

IV 8400679 8400983

IV 8401043 8403788

IV 8404753 8404782

IV 8404874 8407877

IV 8407999 8408054

IV 8409056 8409160

IV 8409208 8412316

IV 8412403 8412584

IV 8412834 8412843

IV 8412916 8413067

IV 8413186 8413199

IV 8413284 8413369

IV 8413601 8413658

IV 8413754 8413850

IV 8413999 8414027

IV 8414797 8414904

IV 8415044 8415100

IV 8415196 8415198

IV 8415388 8415405

IV 8415546 8417864

IV 8417959 8418068

IV 8418242 8419031

IV 8424531 8424717

IV 8424777 8424885

IV 8434803 8434847

IV 8434948 8434978

IV 8441421 8441990

IV 8442071 8442106

IV 8442230 8442826

IV 8442919 8442938

IV 8455341 8455372

IV 8455927 8455943

IV 8456016 8456157

IV 8466638 8466740

IV 8466950 8467228

IV 8467371 8467408

IV 8467581 8467627

IV 8467682 8468258

IV 8468320 8468396

IV 8468601 8468649

IV 8468745 8468837

IV 8468949 8468959

IV 8469204 8469434

IV 8469510 8469601

IV 8469674 8469907

IV 8469998 8470253

IV 8470442 8470540

IV 8470606 8470746

IV 8470831 8470890

IV 8471160 8471170

IV 8471289 8471400

IV 8484132 8484180

IV 8484458 8484506

IV 8500372 8500564

IV 8500798 8500966

IV 8501049 8501060

IV 8508173 8508203

IV 8521302 8521323

IV 8521423 8521497

IV 8563047 8564899

IV 8565319 8565352

IV 8565442 8565483

IV 8565649 8565711

IV 8565809 8566028

IV 8566099 8566141

IV 8566232 8566249

IV 8566461 8566573

IV 8566733 8566740

IV 8566880 8568731

IV 8571459 8571566

IV 8571725 8571777

IV 8572699 8572869

IV 8572954 8573015

IV 8573149 8573151

IV 8573206 8573216

IV 8573287 8573369

IV 8573482 8573587

IV 8573637 8573818

IV 8573881 8573915

IV 8573988 8574018

IV 8574308 8574365

IV 8574452 8574498

IV 8574577 8574748

IV 8574831 8574870

IV 8575395 8575460

IV 8575518 8575579

IV 8575704 8575846

IV 8576056 8576529

IV 8576599 8576726

IV 8576778 8576863

IV 8576977 8576985

IV 8577057 8577160

IV 8577310 8577997

IV 8578057 8578077

IV 8578320 8578365

IV 8578447 8578487

IV 8578543 8578733

IV 8578871 8578988

IV 8579169 8579193

IV 8579242 8579348

IV 8579508 8579515

IV 8579641 8579682

IV 8579825 8579836

IV 8580086 8580406

IV 8580543 8580593

IV 8580700 8580786

IV 8581083 8581125

IV 8581182 8581191

IV 8581247 8581292

IV 8581309 8581366

IV 8581413 8581426

IV 8581487 8581517

IV 8581608 8581626

IV 8607483 8607509

IV 8607618 8607714

IV 8646171 8646222

IV 8663303 8663395

IV 8663557 8663611

IV 8664107 8664119

IV 8664279 8664360

IV 8665208 8665286

IV 8667055 8667350

IV 8667503 8667664

IV 8667755 8667788

IV 8667877 8668149

IV 8715093 8715100

IV 8715785 8715801

IV 8734463 8734559

IV 8734652 8734659

IV 8734779 8734803

IV 8748017 8748143

IV 8749774 8749797

IV 8787795 8788131

IV 8789580 8789803

IV 8823048 8823855

IV 8823950 8824198

IV 8824777 8825024

IV 8825121 8825928

IV 8894827 8894903

IV 8894971 8895008

IV 8895119 8895333

IV 8895586 8895704

IV 8895802 8895908

IV 8896067 8896076

IV 8896183 8896208

IV 8903489 8903651

IV 8948031 8948322

IV 8958249 8958253

IV 8958302 8958377

IV 8958458 8958459

IV 8958526 8958558

IV 8958608 8958828

IV 8958920 8958962

IV 8959103 8959111

IV 8959195 8959385

IV 8959555 8959558

IV 8959719 8959804

IV 8959942 8960190

IV 8960547 8960645

IV 8960742 8960774

IV 8961006 8961099

IV 8961239 8961242

IV 8961715 8961721

IV 8961974 8962122

IV 8962271 8962360

IV 8962526 8962540

IV 8962959 8963012

IV 8963143 8963298

IV 8963492 8963501

IV 8963599 8963660

IV 8963755 8963848

IV 8964020 8964119

IV 8964297 8964578

IV 8997279 8997296

IV 8997409 8997620

IV 8997769 8997806

IV 8997941 8998001

IV 8998081 8998199

IV 8998535 8998739

IV 8998743 8998747

IV 8998830 8998927

IV 8999039 8999102

IV 8999250 8999416

IV 9014005 9014145

IV 9014230 9014288

IV 9014377 9014403

IV 9014498 9014672

IV 9014806 9014896

IV 9014974 9015076

IV 9028594 9028779

IV 9028830 9028834

IV 9028928 9029056

IV 9029226 9029289

IV 9029356 9029540

IV 9029632 9029769

IV 9029830 9029865

IV 9029966 9030041

IV 9038796 9038798

IV 9045903 9045925

IV 9046038 9046048

IV 9046166 9046438

IV 9046567 9046899

IV 9047006 9047141

IV 9047215 9047305

IV 9047369 9047565

IV 9047746 9047752

IV 9047842 9047936

IV 9048027 9048048

IV 9048192 9048245

IV 9048344 9048628

IV 9049129 9049285

IV 9049382 9049402

IV 9049607 9049733

IV 9049807 9049897

IV 9050220 9050287

IV 9050381 9050407

IV 9057000 9058748

IV 9058834 9058842

IV 9058921 9059647

IV 9059697 9059801

IV 9059890 9060610

IV 9060700 9060713

IV 9060803 9062547

IV 9066170 9066505

IV 9066604 9066638

IV 9066734 9067158

IV 9067254 9067292

IV 9067417 9067486

IV 9067926 9067937

IV 9139377 9139442

IV 9149039 9149180

IV 9149425 9149505

IV 9152198 9152335

IV 9155536 9155558

IV 9161173 9161194

IV 9172319 9172324

IV 9175803 9175906

IV 9175954 9176034

IV 9176208 9176521

IV 9176573 9176738

IV 9193241 9193282

IV 9194714 9194733

IV 9194829 9195092

IV 9195271 9195302

IV 9195396 9195440

IV 9195530 9195569

IV 9196068 9196080

IV 9196178 9196294

IV 9196367 9196411

IV 9221445 9221486

IV 9221540 9221958

IV 9222050 9222109

IV 9222212 9222310

IV 9223458 9223614

IV 9223762 9223798

IV 9223968 9224037

IV 9224182 9224228

IV 9226409 9226579

IV 9228646 9228666

IV 9228732 9229101

IV 9229195 9229236

IV 9229327 9229337

IV 9234781 9234791

IV 9235255 9235261

IV 9240162 9240169

IV 9246315 9246540

IV 9246653 9246773

IV 9246863 9246883

IV 9247000 9247148

IV 9249203 9249334

IV 9265185 9265201

IV 9284651 9285214

IV 9285312 9285772

IV 9285866 9286174

IV 9286411 9286464

IV 9286888 9286894

IV 9286944 9287010

IV 9292145 9292708

IV 9292804 9293267

IV 9293363 9293667

IV 9293928 9293988

IV 9297111 9297117

IV 9297207 9297304

IV 9313036 9313104

IV 9331529 9331572

IV 9331803 9331969

IV 9332082 9332109

IV 9332191 9332260

IV 9332452 9332484

IV 9334753 9334754

IV 9347799 9347805

IV 9350740 9351044

IV 9351094 9351143

IV 9368987 9369032

IV 9369323 9369367

IV 9375157 9375551

IV 9379062 9379115

IV 9381909 9382290

IV 9402676 9402704

IV 9402794 9402883

IV 9403478 9403505

IV 9405925 9406013

IV 9406205 9406212

IV 9409573 9409913

IV 9410191 9410217

IV 9410277 9410278

IV 9410374 9410431

IV 9410489 9410552

IV 9410616 9410681

IV 9410772 9410886

IV 9410966 9411200

IV 9411264 9411270

IV 9411324 9411691

IV 9411774 9411848

IV 9411909 9412113

IV 9412651 9413814

IV 9416748 9418284

IV 9418549 9420085

IV 9434002 9434012

IV 9444685 9444719

IV 9457946 9457948

IV 9457949 9457951

IV 9457995 9458139

IV 9458341 9458342

IV 9458429 9458446

IV 9458527 9458531

IV 9458633 9458738

IV 9464560 9464594

IV 9502763 9503922

IV 9524200 9524291

IV 9595603 9595861

IV 9596013 9596145

IV 9596857 9596988

IV 9597139 9597396

IV 9606348 9606495

IV 9620354 9621397

IV 9621480 9621529

IV 9652924 9652948

IV 9653036 9653124

IV 9653212 9653267

IV 9681390 9681510

IV 9685306 9686833

IV 9687169 9687180

IV 9687288 9687293

IV 9689019 9689102

IV 9736290 9736300

IV 9736385 9736437

IV 9741007 9741163

IV 9742053 9742263

IV 9744108 9744288

IV 9744655 9744695

IV 9744786 9744803

IV 9744896 9744904

IV 9745766 9746094

IV 9746478 9746512

IV 9746608 9746626

IV 9746720 9746727

IV 9747460 9747726

IV 9750366 9750380

IV 9750660 9750766

IV 9750938 9750966

IV 9751135 9751158

IV 9764821 9764876

IV 9765173 9765438

IV 9765842 9765890

IV 9766247 9766296

IV 9766758 9767200

IV 9767372 9767392

IV 9768080 9768103

IV 9768198 9768220

IV 9768421 9768433

IV 9768808 9768839

IV 9769488 9769613

IV 9770028 9770272

IV 9808469 9808521

IV 9808815 9808869

IV 9809170 9809175

IV 9809263 9809314

IV 9830926 9831251

IV 9831323 9831427

IV 9831497 9832081

IV 9832508 9832575

IV 9836064 9836070

IV 9836119 9836133

IV 9836222 9836250

IV 9836311 9836360

IV 9837706 9837999

IV 9841568 9841579

IV 9841630 9841836

IV 9843464 9843468

IV 9843780 9843819

IV 9850866 9850871

IV 9852089 9852092

IV 9856621 9856638

IV 9856743 9856821

IV 9867379 9867384

IV 9876867 9876971

IV 9877062 9877585

IV 9877738 9877742

IV 9892915 9892944

IV 9893108 9893114

IV 9893315 9893329

IV 9893533 9893557

IV 9893650 9893676

IV 9894392 9894412

IV 9894584 9894623

IV 9894714 9894984

IV 9895461 9895508

IV 9895865 9895913

IV 10022274 10022672

IV 10022762 10022801

IV 10025287 10025322

IV 10025621 10025624

IV 10028863 10028995

IV 10029277 10029480

IV 10035437 10035559

IV 10035645 10035950

IV 10042519 10042921

IV 10042991 10043036

IV 10061973 10062096

IV 10071097 10071112

IV 10071281 10071300

IV 10073420 10073426

IV 10082970 10082974

IV 10093055 10093074

IV 10093120 10093202

IV 10093312 10093348

IV 10093426 10093441

IV 10095430 10095448

IV 10095546 10095623

IV 10095673 10095756

IV 10100444 10100483

IV 10116511 10116517

IV 10142909 10142930

IV 10143447 10143509

IV 10143558 10143626

IV 10144366 10144384

IV 10160611 10160905

IV 10161023 10161189

IV 10161342 10161355

IV 10161408 10161429

IV 10161516 10161548

IV 10212367 10212512

IV 10212566 10212728

IV 10214370 10214650

IV 10261776 10261886

IV 10261972 10262228

IV 10262324 10262504

IV 10262578 10262629

IV 10262716 10262757

IV 10262832 10262880

IV 10262949 10262998

IV 10263050 10263168

IV 10263403 10263465

IV 10263632 10264144

IV 10264246 10264290

IV 10264400 10264471

IV 10264543 10264563

IV 10264625 10264674

IV 10264791 10264796

IV 10264885 10264912

IV 10264995 10265012

IV 10265095 10265163

IV 10265259 10265363

IV 10305517 10305572

IV 10305628 10305648

IV 10305737 10305773

IV 10306093 10306100

IV 10308051 10308100

IV 10308171 10308376

IV 10308477 10308492

IV 10308677 10308888

IV 10308984 10309364

IV 10309439 10309534

IV 10322381 10322452

IV 10322611 10322745

IV 10322886 10323002

IV 10323085 10323098

IV 10323258 10323395

IV 10323478 10323747

IV 10532188 10532754

IV 10536879 10537445

IV 10540412 10540556

IV 10540615 10540674

IV 10548564 10548655

IV 10548808 10548930

IV 10549176 10549427

IV 10549570 10549634

IV 10549774 10549919

IV 10550057 10550166

IV 10550291 10550470

IV 10550564 10550585

IV 10550680 10551044

IV 10551234 10551347

IV 10551574 10551584

IV 10551905 10551927

IV 10552024 10552038

IV 10552513 10552533

IV 10552741 10552915

IV 10553039 10553128

IV 10553211 10553223

IV 10553303 10553326

IV 10554256 10554350

IV 10555980 10556148

IV 10556240 10556254

IV 10559049 10559218

IV 10559314 10559325

IV 10569440 10569567

IV 10654437 10654536

IV 10657556 10657636

IV 10657867 10657875

IV 10657963 10658036

IV 10658223 10658412

IV 10658464 10658566

IV 10658679 10658727

IV 10681811 10681934

IV 10681992 10682011

IV 10682078 10682133

IV 10686230 10686246

IV 10686473 10686630

IV 10686728 10686759

IV 10686911 10687025

IV 10687235 10687249

IV 10687928 10687957

IV 10707577 10707714

IV 10707868 10707945

IV 10708036 10708119

IV 10708435 10708545

IV 10708613 10708620

IV 10708895 10708898

IV 10709118 10709135

IV 10709232 10709253

IV 10750671 10750672

IV 10750794 10751162

IV 10751257 10751316

IV 10751481 10751690

IV 10751818 10751848

IV 10752019 10752028

IV 10752443 10752628

IV 10752723 10752827

IV 10753220 10753308

IV 10754202 10754266

IV 10756324 10756562

IV 10756659 10757219

IV 10757395 10757467

IV 10757616 10757651

IV 10835200 10835227

IV 10836434 10836444

IV 10843858 10843906

IV 10848955 10848967

IV 10884653 10884970

IV 10940981 10941012

IV 10941163 10941234

IV 10941407 10941967

IV 10942062 10942300

IV 10943171 10943262

IV 10943322 10943398

IV 10943568 10943590

IV 10943707 10943730

IV 10943875 10943938

IV 10944031 10944495

IV 10944669 10944680

IV 10945162 10945173

IV 10945348 10945845

IV 10945937 10945996

IV 10946067 10946231

IV 10946319 10946363

IV 10946445 10946537

IV 10952795 10952861

IV 10953752 10953843

IV 10954232 10954336

IV 10954432 10954616

IV 10955064 10955074

IV 10955249 10955281

IV 10955411 10955618

IV 10955787 10955843

IV 10955938 10956308

IV 10956426 10956429

IV 10956575 10956578

IV 10966243 10966274

IV 10986260 10986309

IV 10986395 10986434

IV 10986817 10986822

IV 10987138 10987206

IV 10987553 10987570

IV 10987733 10987846

IV 10987981 10988015

IV 10988143 10988175

IV 10988289 10988316

IV 10988625 10988669

IV 10997279 10997307

IV 10997419 10997433

IV 10997528 10997620

IV 10997833 10997841

IV 10998004 10998019

IV 10998168 10998212

IV 10998378 10998483

IV 10998534 10998537

IV 10998703 10998775

IV 10998885 10998901

IV 11001173 11001235

IV 11002630 11002684

IV 11002825 11002851

IV 11033651 11034275

IV 11034500 11035230

IV 11035378 11035476

IV 11035611 11035630

IV 11035725 11036046

IV 11071064 11071067

IV 11071236 11071248

IV 11071362 11072341

IV 11072508 11072793

IV 11072857 11072997

IV 11073122 11073281

IV 11073396 11074481

IV 11074558 11074737

IV 11074818 11075238

IV 11075330 11075896

IV 11106328 11106341

IV 11106428 11106501

IV 11107399 11107403

IV 11107517 11107587

IV 11108279 11108593

IV 11154469 11154618

IV 11179099 11179280

IV 11190035 11190082

IV 11190174 11190265

IV 11190363 11190386

IV 11190479 11190654

IV 11190783 11190888

IV 11191036 11191136

IV 11191243 11191409

IV 11191581 11191715

IV 11191800 11191822

IV 11191931 11192021

IV 11192150 11192643

IV 11192731 11193890

IV 11194086 11194393

IV 11194549 11194566

IV 11194685 11194709

IV 11194804 11194847

IV 11194985 11195250

IV 11195355 11195601

IV 11195686 11195787

IV 11195875 11196021

IV 11196164 11196223

IV 11196372 11196618

IV 11196832 11196987

IV 11197178 11197211

IV 11197326 11197430

IV 11197628 11197703

IV 11197764 11197837

IV 11197967 11198120

IV 11198295 11198367

IV 11198579 11198658

IV 11198771 11198832

IV 11199016 11199034

IV 11199127 11199164

IV 11199308 11199426

IV 11199518 11199816

IV 11199888 11199890

IV 11200238 11200244

IV 11200451 11200546

IV 11200678 11200708

IV 11200903 11201028

IV 11201159 11201191

IV 11201402 11201458

IV 11201580 11201642

IV 11201706 11201823

IV 11201945 11202007

IV 11202079 11202086

IV 11202174 11202210

IV 11202477 11202564

IV 11202719 11202720

IV 11202816 11202856

IV 11203054 11203086

IV 11203167 11203221

IV 11203314 11203321

IV 11203456 11203556

IV 11203646 11203710

IV 11203817 11203871

IV 11204150 11204441

IV 11204527 11204568

IV 11204864 11204996

IV 11205395 11205444

IV 11206059 11206089

IV 11206205 11206235

IV 11218884 11218904

IV 11219045 11219050

IV 11221401 11221402

IV 11221403 11221406

IV 11226317 11226354

IV 11238914 11240078

IV 11253784 11253842

IV 11254608 11254692

IV 11263275 11264340

IV 11264936 11266002

IV 11282762 11282898

IV 11282983 11283442

IV 11302145 11302722

IV 11302811 11302921

IV 11302982 11303099

IV 11303201 11303759

IV 11312761 11312770

IV 11313036 11313083

IV 11324061 11324894

IV 11324986 11325158

IV 11325250 11326490

IV 11326580 11327031

IV 11337821 11338271

IV 11338362 11339600

IV 11339691 11340807

IV 11361926 11362179

IV 11362255 11362525

IV 11362675 11362712

IV 11362804 11363463

IV 11368810 11368829

IV 11368923 11369081

IV 11369215 11369258

IV 11369354 11369377

IV 11369543 11369575

IV 11370162 11370204

IV 11382744 11384295

IV 11398641 11400060

IV 11400185 11400198

IV 11400401 11401486

IV 11402973 11402974

IV 11406418 11406636

IV 11406736 11406781

IV 11407232 11407314

IV 11407818 11407830

IV 11407925 11407995

IV 11408237 11408245

IV 11408434 11408682

IV 11408859 11408882

IV 11408974 11409076

IV 11409219 11409252

IV 11409440 11409506

IV 11409595 11409643

IV 11409939 11409968

IV 11416865 11417018

IV 11418852 11419139

IV 11419223 11419532

IV 11419628 11420273

IV 11420361 11420622

IV 11420679 11420800

IV 11420862 11421019

IV 11468068 11468235

IV 11468306 11468739

IV 11468829 11469061

IV 11497231 11497291

IV 11497588 11497589

IV 11497681 11497707

IV 11497912 11497947

IV 11514406 11514583

IV 11514670 11514681

IV 11514731 11514762

IV 11558560 11558561

IV 11580416 11580447

IV 11607740 11607886

IV 11607959 11609951

IV 11610126 11610494

IV 11610579 11610589

IV 11610682 11611744

IV 11611814 11612362

IV 11612414 11615488

IV 11615896 11615916

IV 11616265 11616462

IV 11616541 11616686

IV 11616934 11618922

IV 11618998 11619136

IV 11624476 11624822

IV 11627138 11627484

IV 11642218 11642246

IV 11642751 11643143

IV 11643238 11643636

IV 11660465 11660868

IV 11660961 11661414

IV 11661473 11661799

IV 11673915 11673930

IV 11674416 11674464

IV 11676425 11676443

IV 11677156 11677201

IV 11681122 11681187

IV 11681386 11681408

IV 11728751 11728758

IV 11762748 11763165

IV 11763245 11763394

IV 11763490 11763500

IV 11763601 11763729

IV 11763815 11763822

IV 11763910 11763961

IV 11764041 11764058

IV 11764450 11764521

IV 11802479 11802599

IV 11806211 11806223

IV 11825449 11825653

IV 11825725 11825756

IV 11825829 11825974

IV 11826063 11826460

IV 11826965 11826988

IV 11827380 11827497

IV 11827668 11827672

IV 11827820 11827898

IV 11828352 11828353

IV 11828354 11828357

IV 11829606 11829672

IV 11829765 11829778

IV 11830018 11830124

IV 11834778 11834800

IV 11834886 11834908

IV 11835028 11835084

IV 11835180 11835242

IV 11835336 11835349

IV 11835590 11835698

IV 11836761 11836795

IV 11836880 11836932

IV 11837000 11837054

IV 11837146 11837350

IV 11837448 11837491

IV 11837553 11837697

IV 11837760 11837928

IV 11838109 11838265

IV 11869505 11870663

IV 11876245 11876640

IV 11876736 11877130

IV 11877643 11877665

IV 11943172 11943291

IV 12022118 12022382

IV 12022450 12022588

IV 12022676 12022791

IV 12022845 12022936

IV 12023028 12023736

IV 12023812 12023928

IV 12024003 12024052

IV 12024102 12024810

IV 12024861 12025073

IV 12025124 12025267

IV 12025328 12025431

IV 12025495 12025949

IV 12033013 12033190

IV 12033298 12033304

IV 12037670 12037730

IV 12038431 12038506

IV 12138136 12138162

IV 12155053 12155063

IV 12155151 12155277

IV 12155416 12155583

IV 12155671 12155765

IV 12155863 12155875

IV 12155994 12156015

IV 12156105 12156132

IV 12156218 12156288

IV 12156355 12156380

IV 12169394 12169971

IV 12170041 12170444

IV 12170539 12170627

IV 12174926 12183070

IV 12183159 12191303

IV 12224622 12224675

IV 12224827 12224995

IV 12225089 12225108

IV 12225297 12225334

IV 12225679 12225774

IV 12225832 12226035

IV 12247761 12248102

IV 12248177 12248367

IV 12248428 12248486

IV 12248722 12249238

IV 12249626 12249657

IV 12249710 12249974

IV 12250059 12250066

IV 12250187 12250213

IV 12250275 12250328

IV 12250417 12250423

IV 12250522 12250709

IV 12253972 12254831

IV 12254929 12255145

IV 12255327 12255393

IV 12255513 12255523

IV 12255693 12256183

IV 12256265 12257260

IV 12293925 12293978

IV 12294404 12294477

IV 12294945 12295009

IV 12295123 12295153

IV 12295296 12295308

IV 12295917 12295935

IV 12296027 12296036

IV 12296095 12296178

IV 12296245 12296273

IV 12296323 12296399

IV 12296748 12297080

IV 12297173 12297204

IV 12297301 12297312

IV 12297517 12297541

IV 12307338 12307761

IV 12311117 12311543

IV 12314209 12314270

IV 12314513 12315062

IV 12315163 12316325

IV 12316410 12316428

IV 12316508 12316528

IV 12316580 12318961

IV 12319046 12319056

IV 12319137 12319152

IV 12319235 12319247

IV 12321215 12321248

IV 12321336 12321585

IV 12321649 12322003

IV 12322110 12322241

IV 12322305 12322663

IV 12322794 12323053

IV 12323342 12323482

IV 12323563 12323649

IV 12323742 12323852

IV 12324086 12325606

IV 12344795 12344953

IV 12380324 12380334

IV 12383388 12383409

IV 12396009 12396243

IV 12396293 12396356

IV 12420987 12421037

IV 12421128 12421868

IV 12421951 12421970

IV 12422139 12422245

IV 12422341 12422396

IV 12422867 12423607

IV 12430610 12430719

IV 12460962 12461162

IV 12461236 12461251

IV 12461338 12461370

IV 12461567 12461619

IV 12461783 12462057

IV 12462133 12462200

IV 12462272 12462447

IV 12471512 12478961

IV 12479053 12486496

IV 12490283 12490370

IV 12500556 12500622

IV 12504929 12504984

IV 12505469 12505481

IV 12509867 12512126

IV 12518294 12518295

IV 12519529 12519581

IV 12520864 12520904

IV 12533224 12533385

IV 12554514 12554526

IV 12554628 12554768

IV 12554877 12554996

IV 12558544 12558558

IV 12561120 12561148

IV 12561344 12561351

IV 12564053 12564059

IV 12585020 12585037

IV 12603052 12603359

IV 12603558 12603592

IV 12607724 12608470

IV 12608564 12609194

IV 12621513 12621565

IV 12621640 12621695

IV 12621786 12621817

IV 12638085 12638322

IV 12638377 12638428

IV 12638490 12638516

IV 12638928 12639467

IV 12676149 12676413

IV 12676722 12677256

IV 12679979 12680003

IV 12680789 12680847

IV 12681063 12681157

IV 12681285 12681297

IV 12681570 12681866

IV 12697938 12697985

IV 12698073 12698240

IV 12707265 12707270

IV 12726874 12726915

IV 12727192 12727232

IV 12730600 12730988

IV 12731055 12731358

IV 12731408 12731784

IV 12731839 12731841

IV 12731888 12732658

IV 12733045 12733052

IV 12733369 12733452

IV 12733583 12733636

IV 12733710 12733756

IV 12733989 12734015

IV 12747074 12747078

IV 12782085 12782144

IV 12787157 12787202

IV 12792107 12792131

IV 12792228 12792382

IV 12795628 12795662

IV 12847030 12847038

IV 12856056 12856081

IV 12856176 12856218

IV 12875417 12875881

IV 12875955 12876502

IV 12884622 12884734

IV 12891411 12891594

IV 12891687 12891769

IV 12895719 12895764

IV 12925009 12925020

IV 12926055 12926093

IV 12926264 12926273

IV 12928460 12928462

IV 12928596 12928650

IV 12928796 12928867

IV 12929212 12929514

IV 12929673 12929694

IV 12929787 12930015

IV 12930108 12930116

IV 12930195 12930470

IV 12930563 12930572

IV 12930714 12930736

IV 12930868 12930878

IV 12930966 12931026

IV 12931136 12931342

IV 12931411 12931427

IV 12931521 12931571

IV 12931722 12931814

IV 12931890 12931940

IV 12932067 12932108

IV 12932221 12932490

IV 12932553 12932715

IV 12932935 12933293

IV 12933383 12935630

IV 12935726 12936200

IV 12936797 12937269

IV 12937367 12939610

IV 12939707 12940061

IV 12973279 12973327

IV 12980623 12980637

IV 12983533 12983585

IV 12988249 12988304

IV 13040275 13040288

IV 13040376 13040413

IV 13040478 13040640

IV 13043501 13044249

IV 13044338 13044723

IV 13050168 13050225

IV 13050321 13050383

IV 13050448 13050465

IV 13050628 13050629

IV 13050723 13050921

IV 13051081 13051111

IV 13108931 13109057

IV 13112261 13112382

IV 13113917 13114236

IV 13114288 13115081

IV 13115421 13115466

IV 13115518 13115973

IV 13116066 13116262

IV 13128185 13133689

IV 13133779 13139284

IV 13139928 13140262

IV 13140356 13140379

IV 13140461 13140560

IV 13140632 13140710

IV 13140826 13140938

IV 13141113 13141329

IV 13164358 13164393

IV 13179951 13179957

IV 13180186 13180349

IV 13180572 13180616

IV 13186544 13186591

IV 13186811 13186974

IV 13187112 13187165

IV 13187417 13187420

IV 13189428 13189465

IV 13193046 13193116

IV 13204867 13204901

IV 13223586 13223610

IV 13223757 13225287

IV 13225387 13225627

IV 13225856 13226001

IV 13226126 13226175

IV 13226271 13226290

IV 13226412 13226426

IV 13226573 13226879

IV 13227081 13227575

IV 13227707 13227791

IV 13227899 13227923

IV 13228007 13228127

IV 13229818 13229833

IV 13229959 13230052

IV 13230313 13230373

IV 13230800 13230824

IV 13251852 13251853

IV 13262969 13262977

IV 13263143 13263163

IV 13264522 13264553

IV 13267991 13268020

IV 13291942 13292008

IV 13292084 13292108

IV 13292198 13292207

IV 13292302 13292303

IV 13292417 13292467

IV 13292552 13292558

IV 13292751 13292896

IV 13293796 13294158

IV 13294243 13295502

IV 13295592 13296777

IV 13305166 13305195

IV 13305451 13305467

IV 13311098 13311106

IV 13311186 13311264

IV 13339690 13339746

IV 13340153 13340169

IV 13340794 13341020

IV 13341137 13341208

IV 13341342 13341459

IV 13341565 13341576

IV 13341673 13341691

IV 13342250 13342302

IV 13360592 13360798

IV 13360944 13361035

IV 13361094 13361131

IV 13361322 13361325

IV 13361404 13361547

IV 13361598 13361651

IV 13361776 13361845

IV 13361893 13361894

IV 13363664 13363671

IV 13363998 13364120

IV 13397529 13398342

IV 13407710 13407725

IV 13429534 13429552

IV 13429682 13429734

IV 13431318 13431399

IV 13431960 13431979

IV 13432073 13432087

IV 13432189 13432308

IV 13432444 13432514

IV 13432630 13432858

IV 13433505 13433524

IV 13433980 13434039

IV 13435829 13435883

IV 13435987 13436037

IV 13436144 13436257

IV 13436351 13436456

IV 13436614 13436823

IV 13436915 13438937

IV 13439101 13439163

IV 13440587 13440644

IV 13440817 13440847

IV 13440941 13440969

IV 13441849 13441926

IV 13442895 13442906

IV 13443432 13443489

IV 13443596 13443690

IV 13443788 13443802

IV 13450961 13450973

IV 13455172 13455176

IV 13455238 13455421

IV 13455514 13455523

IV 13456001 13456112

IV 13456342 13456358

IV 13456480 13456556

IV 13458890 13459828

IV 13459922 13460049

IV 13461826 13461828

IV 13462006 13462085

IV 13462184 13462213

IV 13462376 13462389

IV 13462554 13462564

IV 13462638 13462661

IV 13462755 13462759

IV 13462980 13462984

IV 13463145 13463432

IV 13463501 13463542

IV 13463771 13463838

IV 13463888 13464130

IV 13464428 13464482

IV 13464628 13464634

IV 13464712 13464725

IV 13464871 13464937

IV 13464995 13465015

IV 13465381 13465394

IV 13465707 13465719

IV 13477294 13477297

IV 13480006 13480033

IV 13487394 13487404

IV 13487544 13487561

IV 13500092 13500177

IV 13500267 13500352

IV 13503554 13503809

IV 13504126 13504278

IV 13504335 13504424

IV 13504481 13504508

IV 13504869 13504880

IV 13507259 13507267

IV 13507341 13507408

IV 13523709 13523732

IV 13523788 13523807

IV 13526401 13526409

IV 13526615 13526821

IV 13526901 13526907

IV 13526996 13527048

IV 13527292 13527311

IV 13527399 13527408

IV 13527657 13527687

IV 13527737 13527762

IV 13528114 13528151

IV 13528258 13528269

IV 13528359 13528370

IV 13528500 13528645

IV 13528722 13529038

IV 13535569 13535762

IV 13535850 13535912

IV 13535977 13536041

IV 13536156 13536157

IV 13536323 13536771

IV 13548725 13549378

IV 13551420 13551444

IV 13577220 13577253

IV 13577405 13577454

IV 13577546 13577868

IV 13578017 13578307

IV 13578675 13578687

IV 13578778 13578800

IV 13578894 13578939

IV 13578995 13579030

IV 13579180 13579436

IV 13579554 13579611

IV 13579747 13579997

IV 13580090 13580100

IV 13586209 13586235

IV 13586379 13586431

IV 13586529 13586598

IV 13586648 13586802

IV 13588707 13588767

IV 13589539 13589545

IV 13590970 13591021

IV 13592360 13592398

IV 13596857 13596898

IV 13597044 13597094

IV 13597187 13597509

IV 13597657 13597945

IV 13598388 13598397

IV 13599634 13599677

IV 13599769 13599780

IV 13611620 13611629

IV 13611968 13611975

IV 13617872 13617874

IV 13618689 13618697

IV 13627272 13627328

IV 13627383 13627749

IV 13627932 13627966

IV 13628661 13629186

IV 13635809 13635825

IV 13636023 13636159

IV 13636298 13636311

IV 13636579 13636597

IV 13636852 13636932

IV 13640782 13640791

IV 13641547 13641653

IV 13641819 13641846

IV 13651159 13651803

IV 13652021 13652066

IV 13652165 13652236

IV 13654662 13654707

IV 13655112 13655171

IV 13655464 13655521

IV 13655675 13655798

IV 13655882 13656008

IV 13656102 13656157

IV 13656247 13656356

IV 13656416 13656461

IV 13656544 13656722

IV 13656818 13657288

IV 13657383 13657628

IV 13657735 13657946

IV 13658103 13658397

IV 13658594 13659541

IV 13662317 13662963

IV 13663178 13663223

IV 13663322 13663392

IV 13667716 13667861

IV 13668096 13668119

IV 13668261 13668347

IV 13668860 13668887

IV 13669478 13669495

IV 13669574 13669690

IV 13670887 13671535

IV 13671629 13672047

IV 13689091 13689129

IV 13690014 13690054

IV 13697695 13697719

IV 13715952 13715962

IV 13716180 13716233

IV 13733644 13733670

IV 13733949 13734040

IV 13734435 13735567

IV 13735622 13735655

IV 13735712 13735733

IV 13735906 13736849

IV 13736903 13736986

IV 13737111 13737124

IV 13737496 13737551

IV 13737651 13737764

IV 13737943 13738424

IV 13738567 13738702

IV 13738961 13738968

IV 13739050 13739139

IV 13739272 13739456

IV 13739549 13739818

IV 13739901 13739974

IV 13740044 13740055

IV 13740222 13740572

IV 13743541 13743590

IV 13772256 13772275

IV 13802580 13802706

IV 13802770 13803159

IV 13803231 13803376

IV 13803495 13803646

IV 13814615 13814787

IV 13815090 13817350

IV 13832719 13832823

IV 13845843 13845871

IV 13845943 13846050

IV 13846293 13846622

IV 13846730 13846945

IV 13847170 13847191

IV 13847409 13847575

IV 13848457 13848564

IV 13849548 13849570

IV 13849856 13851019

IV 13851546 13851754

IV 13851890 13851894

IV 13852285 13852384

IV 13852926 13853234

IV 13853325 13853382

IV 13853500 13853784

IV 13853903 13853920

IV 13854053 13854081

IV 13854359 13855308

IV 13855528 13855669

IV 13855842 13856043

IV 13856134 13856385

IV 13856477 13856946

IV 13857039 13857220

IV 13857312 13857320

IV 13857496 13857972

IV 13858172 13858217

IV 13858439 13858534

IV 13858620 13858656

IV 13858748 13858876

IV 13858958 13858959

IV 13861565 13862121

IV 13862238 13862316

IV 13862444 13862459

IV 13862545 13862559

IV 13862693 13862704

IV 13862789 13862978

IV 13875118 13875295

IV 13875478 13875594

IV 13875670 13875974

IV 13876048 13876055

IV 13876130 13876456

IV 13879240 13879248

IV 13884326 13884345

IV 13884446 13884509

IV 13884742 13884869

IV 13885057 13885135

IV 13909826 13909885

IV 13909967 13910509

IV 13910661 13910824

IV 13915344 13915369

IV 13915555 13916718

IV 13921193 13921275

IV 13921929 13922223

IV 13922279 13922410

IV 13922585 13922667

IV 13922738 13923121

IV 13923207 13923994

IV 13924090 13924250

IV 13929315 13929433

IV 13929566 13929652

IV 13929729 13929760

IV 13929850 13930766

IV 13930853 13931453

IV 13931544 13931686

IV 13931781 13932240

IV 13932335 13932336

IV 13932384 13932404

IV 13932479 13932614

IV 13932664 13933252

IV 13933358 13933412

IV 13933634 13934013

IV 13942183 13942252

IV 13947082 13947663

IV 13947743 13948281

IV 13964118 13964133

IV 13965245 13965319

IV 13965502 13965549

IV 13965881 13965890

IV 13966046 13966075

IV 13966309 13966324

IV 13966420 13966437

IV 13966672 13966728

IV 13966930 13966934

IV 13967119 13967141

IV 13967787 13967806

IV 13968540 13968570

IV 13969197 13969229

IV 13969383 13969442

IV 13969552 13969668

IV 13969912 13969987

IV 13970120 13970190

IV 13970263 13970391

IV 13970479 13970579

IV 13970699 13970804

IV 13971078 13971092

IV 13971428 13971637

IV 13971711 13971765

IV 13982870 13982888

IV 13984702 13984720

IV 13990129 13990134

IV 14012425 14013587

IV 14017868 14017930

IV 14017981 14018002

IV 14028026 14028068

IV 14028155 14028327

IV 14037067 14037093

IV 14037952 14037976

IV 14048965 14049145

IV 14050796 14050831

IV 14057062 14057096

IV 14057209 14058055

IV 14058205 14058254

IV 14058414 14058572

IV 14058657 14059199

IV 14065887 14066113

IV 14079544 14079548

IV 14079699 14079717

IV 14085004 14085021

IV 14098888 14098898

IV 14109534 14109548

IV 14109638 14109655

IV 14109748 14109751

IV 14109842 14109859

IV 14111635 14111657

IV 14116935 14118099

IV 14118324 14118386

IV 14125469 14125524

IV 14134874 14135119

IV 14135215 14135365

IV 14135454 14136313

IV 14137586 14137606

IV 14137828 14137913

IV 14138023 14138045

IV 14138118 14138193

IV 14138282 14138637

IV 14151636 14151742

IV 14151834 14151941

IV 14152035 14152063

IV 14153699 14153801

IV 14153898 14153925

IV 14154051 14154161

IV 14158668 14158737

IV 14159047 14159116

IV 14184941 14185015

IV 14185124 14185132

IV 14185333 14185429

IV 14185516 14185575

IV 14185666 14185679

IV 14185767 14185959

IV 14186019 14186058

IV 14186189 14186193

IV 14189502 14189516

IV 14191103 14191150

IV 14191483 14191496

IV 14205822 14206415

IV 14206463 14207053

IV 14207140 14207240

IV 14207335 14207368

IV 14208096 14208495

IV 14208560 14208653

IV 14208746 14209080

IV 14209333 14209350

IV 14209445 14209580

IV 14209684 14209691

IV 14209855 14209972

IV 14210085 14210114

IV 14210211 14210243

IV 14210332 14210436

IV 14210521 14211414

IV 14211465 14211855

IV 14211922 14212777

IV 14212871 14215401

IV 14218004 14218123

IV 14218626 14218636

IV 14228036 14228051

IV 14230767 14230779

IV 14233380 14233404

IV 14234447 14234874

IV 14234966 14235576

IV 14235663 14235886

IV 14236061 14236616

IV 14236697 14236901

IV 14238980 14239029

IV 14240200 14240323

IV 14240373 14240436

IV 14245748 14245753

IV 14247528 14247653

IV 14247745 14247883

IV 14248114 14248130

IV 14248189 14248310

IV 14248421 14248520

IV 14251181 14251191

IV 14251329 14251403

IV 14251611 14251894

IV 14252015 14252057

IV 14252143 14252459

IV 14252583 14252620

IV 14252704 14252738

IV 14252938 14253844

IV 14255580 14255682

IV 14256106 14256195

IV 14256305 14257468

IV 14266743 14266800

IV 14281576 14281577

IV 14300432 14300479

IV 14300569 14300772

IV 14301890 14301961

IV 14307647 14307921

IV 14308331 14308579

IV 14308648 14308896

IV 14317224 14317234

IV 14321237 14321507

IV 14322529 14322942

IV 14328267 14328356

IV 14328463 14328538

IV 14336955 14336961

IV 14337013 14337018

IV 14373888 14373918

IV 14374338 14374347

IV 14377425 14377436

IV 14377487 14377504

IV 14383292 14383443

IV 14399421 14399529

IV 14399599 14399697

IV 14400649 14400788

IV 14417747 14418862

IV 14418948 14419119

IV 14425429 14425542

IV 14425657 14425794

IV 14426456 14426604

IV 14426830 14427533

IV 14428322 14428434

IV 14442591 14443027

IV 14443113 14443232

IV 14443347 14443453

IV 14446632 14446776

IV 14447063 14447094

IV 14451382 14451551

IV 14452351 14452416

IV 14452564 14452593

IV 14452689 14452758

IV 14452845 14452959

IV 14453052 14453085

IV 14453140 14453179

IV 14453435 14453451

IV 14453657 14453766

IV 14453908 14453910

IV 14454000 14454073

IV 14454165 14454485

IV 14454539 14454638

IV 14454811 14454873

IV 14454946 14455143

IV 14455291 14455322

IV 14461062 14461086

IV 14461704 14461712

IV 14461829 14461841

IV 14476250 14476262

IV 14476353 14476376

IV 14476537 14476564

IV 14476617 14476785

IV 14476850 14476901

IV 14476977 14477710

IV 14477772 14479553

IV 14479607 14479716

IV 14479766 14479808

IV 14479858 14479982

IV 14480034 14480253

IV 14480309 14480431

IV 14480518 14480548

IV 14480646 14480704

IV 14480791 14481077

IV 14481164 14481203

IV 14481251 14481369

IV 14497406 14497473

IV 14510415 14510460

IV 14510550 14510690

IV 14525944 14525963

IV 14545580 14546739

IV 14548203 14548213

IV 14552151 14552347

IV 14575184 14575193

IV 14575340 14575553

IV 14575602 14575747

IV 14579356 14579375

IV 14580337 14580574

IV 14580670 14580678

IV 14588337 14588448

IV 14607572 14607577

IV 14608141 14608147

IV 14633478 14633490

IV 14633580 14633822

IV 14634263 14634284

IV 14648391 14648394

IV 14654979 14655008

IV 14655103 14655260

IV 14655353 14655363

IV 14655679 14655702

IV 14655834 14655835

IV 14655836 14655837

IV 14655931 14655938

IV 14656033 14656069

IV 14659088 14659092

IV 14662481 14662516

IV 14699236 14699296

IV 14711807 14711808

IV 14712702 14712832

IV 14713736 14713930

IV 14714609 14714610

IV 14718586 14718715

IV 14719393 14719585

IV 14720313 14720314

IV 14739692 14739724

IV 14739904 14740060

IV 14740149 14740216

IV 14740287 14740310

IV 14740400 14740475

IV 14740592 14740962

IV 14741112 14741226

IV 14747597 14747732

IV 14761569 14761577

IV 14763437 14763459

IV 14764939 14764999

IV 14774035 14774056

IV 14774646 14774658

IV 14774786 14774888

IV 14774941 14775065

IV 14775271 14775278

IV 14775437 14775682

IV 14775736 14775749

IV 14775922 14775972

IV 14776038 14776089

IV 14776394 14776411

IV 14776473 14776687

IV 14776747 14777230

IV 14777477 14777831

IV 14777972 14778039

IV 14778237 14778274

IV 14778904 14778929

IV 14779031 14779223

IV 14779319 14779570

IV 14779717 14779864

IV 14779984 14780062

IV 14780204 14780241

IV 14780472 14780512

IV 14780602 14780661

IV 14781196 14781212

IV 14781349 14781412

IV 14800024 14800030

IV 14800090 14800160

IV 14805238 14805239

IV 14806740 14806772

IV 14837377 14837699

IV 14838793 14838986

IV 14839079 14839084

IV 14839197 14839228

IV 14839485 14839528

IV 14839722 14839853

IV 14840137 14840160

IV 14840504 14840511

IV 14840990 14841030

IV 14841302 14841422

IV 14841683 14841734

IV 14841923 14841951

IV 14842341 14842346

IV 14842651 14842696

IV 14843063 14843078

IV 14843178 14843242

IV 14845255 14845263

IV 14845902 14845914

IV 14846480 14846513

IV 14858765 14858884

IV 14858974 14859024

IV 14860788 14860796

IV 14860922 14860986

IV 14861081 14861111

IV 14881908 14881924

IV 14882183 14882198

IV 14882448 14882804

IV 14910829 14911054

IV 14911164 14911244

IV 14911336 14911574

IV 14915918 14916261

IV 14917849 14918193

IV 14921402 14921406

IV 14922594 14922682

IV 14925380 14925386

IV 14929813 14929838

IV 14931672 14931805

IV 14932705 14932807

IV 14935756 14935769

IV 14935887 14935977

IV 14936297 14936672

IV 14936859 14936957

IV 14937555 14937650

IV 14937848 14938229

IV 14938542 14938631

IV 14938749 14938763

IV 14938968 14939051

IV 14941638 14941770

IV 14947519 14947873

IV 14947970 14948041

IV 14948254 14948507

IV 14948584 14948595

IV 14950239 14950276

IV 14951142 14951179

IV 14963003 14963080

IV 14966183 14966404

IV 14966465 14966546

IV 14994437 14994527

IV 14997146 14997214

IV 15004404 15004494

IV 15006324 15006337

IV 15006567 15006724

IV 15006817 15006849

IV 15007001 15007119

IV 15007329 15007345

IV 15008025 15008052

IV 15009527 15009528

IV 15017454 15017620

IV 15017705 15017751

IV 15043808 15043959

IV 15048042 15048105

IV 15048307 15048314

IV 15058713 15058942

IV 15059016 15059172

IV 15059222 15059294

IV 15059505 15059526

IV 15059580 15059607

IV 15059746 15059772

IV 15059886 15060063

IV 15060171 15060203

IV 15060291 15060343

IV 15060471 15060508

IV 15075789 15075893

IV 15075981 15076475

IV 15076544 15076673

IV 15076760 15077085

IV 15077160 15077470

IV 15077559 15077886

IV 15077940 15077962

IV 15078161 15079063

IV 15079157 15079710

IV 15079780 15080119

IV 15080202 15080287

IV 15080372 15080632

IV 15080687 15080909

IV 15080996 15081142

IV 15081225 15082003

IV 15082076 15082446

IV 15082517 15082899

IV 15082976 15083080

IV 15096118 15096148

IV 15102279 15102299

IV 15130307 15130311

IV 15130407 15130427

IV 15130885 15130904

IV 15133427 15133471

IV 15133682 15133687

IV 15134225 15134267

IV 15148720 15148740

IV 15149869 15149871

IV 15152724 15152740

IV 15152888 15152928

IV 15153008 15153104

IV 15153291 15153365

IV 15153452 15153454

IV 15153550 15153736

IV 15153785 15153840

IV 15154686 15154912

IV 15156710 15156794

IV 15156886 15159713

IV 15160425 15163252

IV 15163349 15163428

IV 15166396 15166407

IV 15166495 15166751

IV 15166801 15166858

IV 15166948 15166972

IV 15167090 15167165

IV 15167247 15167498

IV 15169310 15169541

IV 15169629 15169698

IV 15169784 15169955

IV 15170176 15170349

IV 15170455 15170476

IV 15170566 15170730

IV 15183412 15183575

IV 15183628 15183644

IV 15183733 15183795

IV 15184039 15184067

IV 15184130 15184167

IV 15184359 15184396

IV 15184491 15184603

IV 15184768 15184864

IV 15185104 15185172

IV 15185427 15185435

IV 15185538 15185585

IV 15185697 15185873

IV 15186087 15186099

IV 15186561 15186605

IV 15186699 15186745

IV 15186967 15187044

IV 15187138 15187166

IV 15188336 15188353

IV 15188449 15188703

IV 15189032 15189063

IV 15189153 15189458

IV 15189553 15189647

IV 15189697 15190620

IV 15190673 15190899

IV 15191034 15191290

IV 15193587 15193753

IV 15194564 15194571

IV 15194629 15194666

IV 15195891 15195955

IV 15196127 15196140

IV 15196207 15196246

IV 15197475 15197535

IV 15197585 15197799

IV 15197886 15197898

IV 15198034 15198044

IV 15198136 15198177

IV 15198282 15198290

IV 15198850 15198855

IV 15198956 15198957

IV 15199166 15199175

IV 15199592 15199648

IV 15199715 15199770

IV 15199853 15199878

IV 15200101 15200105

IV 15200701 15200775

IV 15200860 15200917

IV 15201001 15201075

IV 15201560 15201582

IV 15201991 15202032

IV 15202396 15202427

IV 15205907 15205911

IV 15206146 15206249

IV 15206342 15206400

IV 15210119 15210177

IV 15210272 15210374

IV 15210585 15210587

IV 15212820 15212842

IV 15212938 15212999

IV 15213118 15213165

IV 15213986 15214041

IV 15214162 15214195

IV 15218240 15218252

IV 15218374 15218513

IV 15219769 15219843

IV 15219968 15220005

IV 15220121 15220169

IV 15221325 15221789

IV 15221885 15223111

IV 15223305 15223377

IV 15223630 15223704

IV 15223832 15223867

IV 15223947 15225175

IV 15225269 15225732

IV 15225910 15227036

IV 15227115 15227230

IV 15233573 15233633

IV 15234384 15234441

IV 15248219 15248252

IV 15282142 15282170

IV 15282812 15282841

IV 15283251 15283333

IV 15289919 15290086

IV 15290180 15291054

IV 15292291 15293421

IV 15303393 15303397

IV 15303599 15303705

IV 15303796 15303812

IV 15303899 15304112

IV 15304324 15304342

IV 15307590 15307627

IV 15311152 15311163

IV 15311307 15311383

IV 15311527 15311603

IV 15311701 15311960

IV 15318515 15318523

IV 15318664 15318744

IV 15318885 15318961

IV 15319060 15319321

IV 15319909 15319985

IV 15320035 15320258

IV 15320335 15320527

IV 15320625 15320813

IV 15321008 15321017

IV 15321106 15321140

IV 15321217 15321238

IV 15323273 15323322

IV 15324342 15324502

IV 15324701 15324773

IV 15327307 15327374

IV 15327577 15327736

IV 15329505 15329553

IV 15342537 15342981

IV 15343070 15343705

IV 15343790 15344618

IV 15352227 15353424

IV 15353529 15354728

IV 15373621 15373833

IV 15373884 15373937

IV 15374025 15375457

IV 15375551 15375560

IV 15375673 15375701

IV 15375836 15375865

IV 15375950 15375955

IV 15376065 15376081

IV 15376136 15376353

IV 15376447 15376979

IV 15377074 15377102

IV 15390402 15390432

IV 15409459 15409496

IV 15409564 15409584

IV 15409655 15409717

IV 15415281 15415411

IV 15415778 15420015

IV 15420724 15424958

IV 15425403 15425531

IV 15428253 15428375

IV 15428955 15429078

IV 15434516 15434520

IV 15435467 15435488

IV 15435538 15435582

IV 15435725 15435739

IV 15437024 15437147

IV 15437359 15437368

IV 15437797 15437920

IV 15439505 15439546

IV 15440177 15440225

IV 15441425 15441443

IV 15443914 15443919

IV 15444126 15444227

IV 15444765 15444775

IV 15444838 15444912

IV 15445870 15446093

IV 15446224 15446446

IV 15449738 15449918

IV 15450059 15450239

IV 15460457 15460723

IV 15460773 15461125

IV 15461860 15462004

IV 15467868 15468191

IV 15468377 15468388

IV 15480745 15481133

IV 15482024 15482041

IV 15482131 15482520

IV 15484137 15485671

IV 15491764 15491794

IV 15491946 15491985

IV 15498741 15499902

IV 15501101 15501143

IV 15501237 15501400

IV 15501731 15502071

IV 15502156 15502663

IV 15502812 15502822

IV 15503000 15503019

IV 15503091 15503268

IV 15511917 15511976

IV 15512116 15512120

IV 15512202 15512215

IV 15512359 15512363

IV 15512418 15512516

IV 15512592 15513248

IV 15513517 15513673

IV 15513770 15513823

IV 15517261 15517338

IV 15517399 15517466

IV 15519162 15519201

IV 15519295 15519506

IV 15519578 15519674

IV 15519736 15519968

IV 15521549 15521652

IV 15521746 15521750

IV 15521843 15521863

IV 15522071 15522125

IV 15522197 15522483

IV 15522814 15522818

IV 15523268 15523273

IV 15529077 15529112

IV 15529421 15529439

IV 15529769 15529778

IV 15529955 15530031

IV 15530713 15530754

IV 15555633 15555824

IV 15555895 15556017

IV 15557344 15557501

IV 15557615 15557686

IV 15557773 15557810

IV 15558207 15558219

IV 15558314 15558337

IV 15559680 15559695

IV 15559788 15559841

IV 15560272 15560320

IV 15560567 15560579

IV 15577119 15577392

IV 15578904 15580117

IV 15590808 15591316

IV 15591403 15591406

IV 15591511 15591760

IV 15594004 15594911

IV 15595386 15596495

IV 15596578 15596631

IV 15597363 15597766

IV 15597879 15598052

IV 15598138 15598273

IV 15598423 15598679

IV 15598867 15599058

IV 15599117 15599251

IV 15599324 15600075

IV 15600170 15600284

IV 15600370 15600563

IV 15607162 15607173

IV 15607788 15608006

IV 15608122 15608158

IV 15608418 15608635

IV 15609257 15609267

IV 15609940 15609962

IV 15610148 15610212

IV 15614338 15614341

IV 15632030 15632065

IV 15632489 15632554

IV 15634631 15634714

IV 15634807 15634858

IV 15634952 15635073

IV 15635168 15635267

IV 15636193 15636215

IV 15636265 15636290

IV 15636386 15636659

IV 15636825 15636849

IV 15636964 15636987

IV 15639885 15639894

IV 15640784 15640812

IV 15641475 15641860

IV 15642029 15642054

IV 15642351 15642445

IV 15643249 15643259

IV 15643416 15643843

IV 15643968 15644004

IV 15644172 15644230

IV 15644399 15644424

IV 15644796 15644818

IV 15646031 15646034

IV 15646904 15646985

IV 15649696 15649701

IV 15652393 15652529

IV 15652682 15652741

IV 15652804 15652953

IV 15653063 15653273

IV 15653628 15653643

IV 15654013 15654044

IV 15654106 15654343

IV 15656471 15656488

IV 15656584 15656701

IV 15665009 15665033

IV 15665785 15665802

IV 15667102 15667152

IV 15667784 15667817

IV 15667945 15668021

IV 15669658 15669694

IV 15669819 15669896

IV 15670894 15670916

IV 15671631 15671648

IV 15676405 15676452

IV 15676538 15677212

IV 15677876 15678553

IV 15678636 15678681

IV 15680997 15681040

IV 15681224 15681230

IV 15681771 15681809

IV 15684150 15684246

IV 15684342 15684369

IV 15684970 15685109

IV 15685384 15685427

IV 15686059 15686195

IV 15686728 15686755

IV 15686849 15686945

IV 15687234 15687374

IV 15688501 15688660

IV 15691506 15691535

IV 15692722 15692821

IV 15703560 15703567

IV 15703756 15703787

IV 15704171 15704209

IV 15704295 15704326

IV 15708048 15708232

IV 15708288 15708467

IV 15708529 15708874

IV 15708999 15709451

IV 15709582 15709708

IV 15709897 15710121

IV 15710340 15710492

IV 15715269 15715402

IV 15715492 15715792

IV 15715853 15716233

IV 15718685 15718700

IV 15729859 15730085

IV 15730157 15730219

IV 15730279 15730454

IV 15730504 15730541

IV 15730599 15730611

IV 15730764 15730827

IV 15730945 15730986

IV 15733080 15733225

IV 15733320 15733471

IV 15733577 15734503

IV 15734597 15739786

IV 15739878 15745363

IV 15747559 15747567

IV 15752176 15752180

IV 15754215 15754350

IV 15754445 15754794

IV 15756416 15756426

IV 15756769 15756906

IV 15757264 15757613

IV 15760762 15760774

IV 15760890 15760962

IV 15765583 15765594

IV 15765686 15765727

IV 15765934 15766008

IV 15766161 15766390

IV 15766446 15766456

IV 15766539 15766664

IV 15766726 15766735

IV 15766949 15767133

IV 15767220 15767310

IV 15767375 15767407

IV 15767501 15767511

IV 15767955 15768179

IV 15768984 15768987

IV 15769077 15769133

IV 15769281 15769286

IV 15776141 15776324

IV 15776413 15776517

IV 15777960 15778235

IV 15782397 15782433

IV 15782601 15782654

IV 15783083 15783157

IV 15783634 15783666

IV 15783760 15783821

IV 15785171 15785180

IV 15785260 15785325

IV 15785418 15785516

IV 15785577 15785712

IV 15785801 15785880

IV 15785972 15785996

IV 15786205 15786232

IV 15787888 15787932

IV 15788027 15788055

IV 15790848 15791002

IV 15797878 15797966

IV 15798214 15798320

IV 15798709 15798713

IV 15798828 15798843

IV 15798920 15799027

IV 15799079 15799089

IV 15799338 15799390

IV 15799477 15799586

IV 15799715 15799736

IV 15800024 15800154

IV 15800268 15800360

IV 15800443 15800455

IV 15800516 15800575

IV 15800647 15800936

IV 15800985 15801213

IV 15801303 15801481

IV 15801575 15801601

IV 15801677 15801725

IV 15801820 15801932

IV 15802089 15802176

IV 15805233 15805247

IV 15805336 15805501

IV 15826087 15826315

IV 15826364 15826584

IV 15826685 15826753

IV 15826803 15826902

IV 15827000 15827099

IV 15827194 15827252

IV 15827317 15827336

IV 15827590 15827634

IV 15836246 15836277

IV 15836392 15836422

IV 15842642 15842648

IV 15853007 15853015

IV 15859252 15859271

IV 15859327 15859361

IV 15862750 15862904

IV 15887672 15887690

IV 15888299 15888308

IV 15895476 15895535

IV 15895623 15895798

IV 15895914 15895942

IV 15895988 15896083

IV 15896169 15896177

IV 15896444 15896453

IV 15915110 15915117

IV 15915972 15915993

IV 15942656 15942687

IV 15943342 15943360

IV 15943707 15943710

IV 15943711 15943715

IV 15971210 15971593

IV 15971705 15972131

IV 15972213 15972237

IV 15972332 15972341

IV 15972575 15972699

IV 15972802 15972817

IV 15979737 15979802

IV 16008026 16009115

IV 16030706 16030779

IV 16030844 16030927

IV 16031126 16031295

IV 16051068 16051459

IV 16055359 16055390

IV 16055912 16055945

IV 16064995 16065302

IV 16065468 16065500

IV 16065609 16065660

IV 16066023 16066183

IV 16066232 16066271

IV 16066532 16066603

IV 16094716 16094833

IV 16094940 16095130

IV 16095225 16095384

IV 16095449 16095605

IV 16095664 16095725

IV 16095799 16095894

IV 16099297 16099502

IV 16099605 16099671

IV 16099765 16102576

IV 16102779 16102992

IV 16103098 16103129

IV 16106477 16109289

IV 16112976 16113019

IV 16113070 16113076

IV 16121905 16121967

IV 16122070 16122474

IV 16127429 16127478

IV 16127563 16127722

IV 16127785 16127951

IV 16127998 16128022

IV 16128104 16129428

IV 16129517 16129699

IV 16135468 16135481

IV 16135578 16135799

IV 16148067 16148111

IV 16149528 16149570

IV 16153263 16153383

IV 16153468 16153665

IV 16153784 16153841

IV 16153896 16153902

IV 16154043 16154360

IV 16154450 16154462

IV 16154577 16154674

IV 16154726 16154737

IV 16167441 16167485

IV 16171705 16172234

IV 16172325 16172410

IV 16209564 16209632

IV 16209713 16209724

IV 16217338 16217475

IV 16223560 16223632

IV 16224066 16224072

IV 16224250 16224318

IV 16233369 16233521

IV 16234561 16234596

IV 16234744 16234760

IV 16239972 16240530

IV 16240633 16240691

IV 16240768 16240855

IV 16240950 16241302

IV 16241400 16241475

IV 16241549 16241903

IV 16241995 16242118

IV 16242202 16242461

IV 16242552 16242742

IV 16242834 16243276

IV 16243356 16243648

IV 16243740 16243914

IV 16244155 16244318

IV 16244391 16244569

IV 16244619 16244665

IV 16244784 16244785

IV 16244976 16245067

IV 16245158 16245195

IV 16245297 16245361

IV 16249500 16249621

IV 16254237 16254262

IV 16254353 16254355

IV 16254358 16254359

IV 16255587 16255618

IV 16258869 16258899

IV 16260275 16260411

IV 16260503 16260558

IV 16260646 16260664

IV 16261171 16261291

IV 16261390 16261542

IV 16261705 16261767

IV 16262065 16262083

IV 16262496 16262503

IV 16262817 16262911

IV 16263017 16263186

IV 16263740 16263764

IV 16263857 16263948

IV 16264722 16264866

IV 16264961 16264978

IV 16265331 16265413

IV 16265852 16265882

IV 16266186 16266236

IV 16266372 16266424

IV 16266486 16266638

IV 16266737 16266744

IV 16266840 16266858

IV 16267226 16267288

IV 16267337 16267435

IV 16267569 16267661

IV 16268002 16268162

IV 16268256 16268260

IV 16268353 16268499

IV 16276963 16277066

IV 16282092 16282169

IV 16285478 16285585

IV 16285843 16285915

IV 16286059 16286070

IV 16286129 16286225

IV 16286709 16286718

IV 16288174 16288181

IV 16307728 16307800

IV 16307892 16307903

IV 16308225 16308297

IV 16309683 16309798

IV 16310426 16310499

IV 16310594 16310603

IV 16311079 16311151

IV 16312155 16312271

IV 16312375 16312439

IV 16312523 16312591

IV 16318176 16318294

IV 16318399 16318462

IV 16318545 16318612

IV 16319558 16319675

IV 16338817 16341538

IV 16341631 16341649

IV 16342215 16342233

IV 16342326 16345049

IV 16345888 16346168

IV 16348222 16348503

IV 16348914 16348936

IV 16349119 16349146

IV 16349852 16350150

IV 16350279 16350288

IV 16351314 16351450

IV 16352430 16352725

IV 16352907 16352927

IV 16359809 16359818

IV 16359910 16360209

IV 16360298 16360342

IV 16360411 16360504

IV 16360686 16360895

IV 16360987 16360988

IV 16361101 16361142

IV 16361230 16361332

IV 16361427 16361557

IV 16379179 16379189

IV 16386448 16386456

IV 16386840 16387003

IV 16390201 16390383

IV 16390470 16390555

IV 16390625 16390807

IV 16390897 16390979

IV 16408573 16408647

IV 16418460 16418511

IV 16418605 16418774

IV 16418828 16418843

IV 16421608 16421844

IV 16421922 16421926

IV 16422882 16422902

IV 16423834 16424184

IV 16424235 16424280

IV 16424372 16424411

IV 16424507 16424544

IV 16426515 16426564

IV 16426794 16426834

IV 16426918 16427199

IV 16428385 16428388

IV 16428486 16428490

IV 16434029 16434050

IV 16436438 16436458

IV 16440661 16440800

IV 16440971 16441209

IV 16441306 16441328

IV 16441421 16441428

IV 16441524 16441543

IV 16441657 16441929

IV 16442031 16442081

IV 16444562 16444584

IV 16446079 16446103

IV 16451229 16451272

IV 16455908 16455916

IV 16458446 16458676

IV 16458892 16458930

IV 16459168 16459182

IV 16459270 16459366

IV 16459458 16459497

IV 16460593 16460630

IV 16464042 16464067

IV 16464716 16464995

IV 16465089 16465320

IV 16465419 16465649

IV 16465744 16466025

IV 16468347 16468373

IV 16468453 16468514

IV 16470569 16470619

IV 16470711 16471010

IV 16471179 16471269

IV 16480266 16480287

IV 16480381 16480448

IV 16480927 16481026

IV 16481169 16481692

IV 16481862 16481954

IV 16482234 16482327

IV 16482425 16482494

IV 16482579 16482583

IV 16506400 16506491

IV 16506664 16506880

IV 16507395 16507496

IV 16507969 16508033

IV 16508131 16508147

IV 16510809 16510951

IV 16511046 16511849

IV 16511944 16512078

IV 16512170 16512383

IV 16512446 16512600

IV 16512695 16514759

IV 16514846 16515176

IV 16515266 16517995

IV 16518079 16520293

IV 16533138 16533192

IV 16533288 16533338

IV 16534142 16534181

IV 16552029 16552270

IV 16552352 16552412

IV 16552504 16552605

IV 16552671 16552778

IV 16552869 16553042

IV 16553307 16553455

IV 16555648 16555828

IV 16560735 16560834

IV 16566619 16566789

IV 16568253 16568348

IV 16574174 16574221

IV 16574318 16574423

IV 16574592 16574622

IV 16574708 16574812

IV 16575890 16577048

IV 16577203 16577362

IV 16580342 16580356

IV 16581311 16581350

IV 16583762 16583923

IV 16585481 16585630

IV 16585686 16585755

IV 16585887 16585919

IV 16586169 16586171

IV 16586263 16586343

IV 16586732 16586756

IV 16590254 16590362

IV 16590459 16592654

IV 16592890 16592904

IV 16593269 16593272

IV 16593504 16595701

IV 16595796 16595907

IV 16598997 16599086

IV 16599138 16599155

IV 16610897 16610915

IV 16613041 16613162

IV 16614994 16615189

IV 16615400 16615452

IV 16615543 16615552

IV 16615670 16615723

IV 16615817 16615825

IV 16616844 16616858

IV 16617019 16617028

IV 16618651 16618666

IV 16618827 16618834

IV 16619112 16619136

IV 16626257 16626265

IV 16626368 16626444

IV 16629193 16629200

IV 16630973 16631016

IV 16631114 16631192

IV 16632050 16632094

IV 16637052 16637065

IV 16637172 16637311

IV 16637422 16637539

IV 16638377 16638524

IV 16638684 16638695

IV 16638816 16638852

IV 16639008 16639192

IV 16639264 16639297

IV 16639379 16639401

IV 16640511 16640517

IV 16640703 16640741

IV 16640848 16640889

IV 16641426 16641455

IV 16641821 16641880

IV 16642622 16642623

IV 16642762 16642779

IV 16642929 16642975

IV 16643157 16643292

IV 16643497 16643518

IV 16643632 16643746

IV 16644337 16644352

IV 16644528 16644585

IV 16644839 16644855

IV 16645028 16645053

IV 16646208 16646256

IV 16647357 16647360

IV 16647462 16647571

IV 16648390 16648444

IV 16648979 16649019

IV 16649127 16649356

IV 16649587 16649616

IV 16649711 16649783

IV 16650428 16650433

IV 16651013 16651040

IV 16651433 16651571

IV 16652540 16652547

IV 16653293 16653380

IV 16654343 16654372

IV 16654547 16654568

IV 16654666 16654682

IV 16654922 16655026

IV 16655092 16655219

IV 16655488 16655521

IV 16655666 16655709

IV 16655864 16655912

IV 16658248 16658276

IV 16664267 16664410

IV 16664503 16664574

IV 16666295 16666432

IV 16670546 16670591

IV 16670745 16670803

IV 16670901 16670902

IV 16672606 16672744

IV 16672841 16672907

IV 16676051 16676067

IV 16678178 16678319

IV 16678910 16679342

IV 16679485 16679585

IV 16679928 16680010

IV 16680204 16680268

IV 16680344 16680416

IV 16680512 16680672

IV 16680753 16680801

IV 16680883 16680925

IV 16680981 16681294

IV 16681357 16681480

IV 16681555 16681826

IV 16682040 16682044

IV 16682139 16682197

IV 16682351 16682393

IV 16702918 16702925

IV 16704842 16704848

IV 16706220 16706252

IV 16706336 16706638

IV 16708865 16708886

IV 16708972 16709095

IV 16711845 16711870

IV 16711939 16713375

IV 16713469 16714903

IV 16724044 16724104

IV 16724199 16724214

IV 16730293 16730332

IV 16730390 16730458

IV 16730552 16730568

IV 16731011 16731025

IV 16731208 16731268

IV 16731322 16731374

IV 16731528 16731668

IV 16731878 16731889

IV 16733156 16733208

IV 16734786 16734892

IV 16734993 16735335

IV 16735417 16735626

IV 16735752 16735832

IV 16735891 16735904

IV 16737573 16737586

IV 16738084 16738147

IV 16739561 16739568

IV 16741527 16741572

IV 16743033 16743036

IV 16743888 16743969

IV 16744314 16744325

IV 16744395 16744456

IV 16744625 16744637

IV 16744909 16744931

IV 16745100 16745205

IV 16745820 16745822

IV 16746117 16746128

IV 16746261 16746343

IV 16746474 16746477

IV 16746572 16746611

IV 16746701 16746807

IV 16747034 16747224

IV 16747294 16747355

IV 16748680 16748743

IV 16748835 16748854

IV 16750590 16750614

IV 16751820 16751870

IV 16751945 16752026

IV 16752329 16752418

IV 16752510 16752558

IV 16752716 16752949

IV 16753052 16753471

IV 16753579 16753656

IV 16753729 16754173

IV 16754321 16754359

IV 16754817 16755042

IV 16755132 16755238

IV 16755412 16755439

IV 16755857 16755982

IV 16756316 16756372

IV 16756499 16756558

IV 16756652 16756876

IV 16757147 16757173

IV 16757279 16757304

IV 16757404 16757519

IV 16757710 16757751

IV 16757840 16757940

IV 16758199 16758276

IV 16758601 16758771

IV 16758911 16758930

IV 16759173 16759241

IV 16759359 16759369

IV 16759521 16759614

IV 16759701 16759739

IV 16759834 16759888

IV 16760308 16760319

IV 16760730 16760876

IV 16760998 16761022

IV 16761136 16761191

IV 16761243 16761352

IV 16761431 16761450

IV 16761537 16761577

IV 16761774 16761847

IV 16762201 16762207

IV 16762208 16762211

IV 16762291 16762427

IV 16762517 16762628

IV 16762747 16762894

IV 16763017 16763065

IV 16763156 16763168

IV 16763502 16763560

IV 16763668 16763685

IV 16763928 16764030

IV 16764184 16764652

IV 16764743 16764769

IV 16764856 16764879

IV 16765036 16765076

IV 16765236 16765353

IV 16765746 16765868

IV 16766200 16766205

IV 16766327 16767867

IV 16767949 16767963

IV 16768155 16768182

IV 16768308 16768326

IV 16768418 16768438

IV 16768519 16770062

IV 16770390 16770485

IV 16770579 16770622

IV 16770711 16770876

IV 16770963 16771009

IV 16771091 16771138

IV 16788663 16788729

IV 16797477 16797608

IV 16797734 16797749

IV 16803665 16803850

IV 16805259 16805442

IV 16807682 16807734

IV 16807828 16808317

IV 16808493 16808562

IV 16808803 16808845

IV 16809149 16809215

IV 16809391 16809881

IV 16809975 16810026

IV 16813429 16813521

IV 16813684 16813723

IV 16815395 16815444

IV 16815531 16815637

IV 16815982 16816018

IV 16816539 16816997

IV 16818420 16818625

IV 16818803 16818870

IV 16818928 16818980

IV 16819071 16819102

IV 16819207 16819291

IV 16819385 16819400

IV 16819587 16819655

IV 16819756 16819803

IV 16819896 16819928

IV 16822855 16822897

IV 16822990 16823576

IV 16825191 16825199

IV 16825249 16825692

IV 16827254 16827841

IV 16827936 16827977

IV 16828429 16828456

IV 16830403 16830441

IV 16830602 16830699

IV 16843030 16843041

IV 16843435 16843697

IV 16843821 16843859

IV 16843908 16843996

IV 16844056 16844193

IV 16844287 16844428

IV 16844562 16844748

IV 16844898 16844917

IV 16845599 16845663

IV 16845795 16845954

IV 16846719 16846760

IV 16846853 16846894

IV 16846988 16847014

IV 16847112 16847120

IV 16847278 16847341

IV 16847427 16847460

IV 16847539 16847542

IV 16849828 16849849

IV 16853947 16853973

IV 16854039 16854098

IV 16854397 16854577

IV 16866619 16866656

IV 16867042 16867208

IV 16867991 16867994

IV 16879423 16879487

IV 16879602 16879640

IV 16879729 16879800

IV 16879978 16880005

IV 16880568 16880648

IV 16880744 16880766

IV 16880828 16880873

IV 16880957 16881023

IV 16881092 16881357

IV 16881421 16881434

IV 16881515 16881640

IV 16881690 16881954

IV 16882071 16882124

IV 16882213 16882314

IV 16882458 16882563

IV 16882657 16883186

IV 16883250 16883490

IV 16884858 16885009

IV 16885076 16885119

IV 16893651 16893674

IV 16894141 16894245

IV 16895196 16895265

IV 16895858 16896080

IV 16897939 16897965

IV 16898902 16899020

IV 16900564 16900600

IV 16901236 16901286

IV 16901454 16901457

IV 16901659 16901668

IV 16902246 16902270

IV 16902541 16902559

IV 16902670 16902711

IV 16909691 16909762

IV 16909871 16909872

IV 16910126 16910333

IV 16910405 16910458

IV 16910575 16910673

IV 16910738 16910759

IV 16910852 16910963

IV 16911036 16911155

IV 16930225 16930274

IV 16937023 16937045

IV 16943361 16943630

IV 16943690 16943848

IV 16943943 16943971

IV 16945127 16945198

IV 16945317 16945392

IV 16959420 16959475

IV 16959831 16959877

IV 16963453 16964660

IV 16964735 16964760

IV 16964814 16964855

IV 16971150 16971158

IV 16971304 16971333

IV 16971429 16971454

IV 16971544 16971685

IV 16971769 16971778

IV 16971902 16972036

IV 16988903 16988989

IV 16989484 16989512

IV 16997042 16997078

IV 16997165 16997446

IV 16997527 16997536

IV 16997598 16997669

IV 17000438 17000551

IV 17000704 17000719

IV 17000977 17001092

IV 17001962 17002000

IV 17002068 17002254

IV 17002345 17002493

IV 17002575 17002631

IV 17006361 17006408

IV 17006495 17006521

IV 17006596 17006898

IV 17006992 17007137

IV 17007221 17008443

IV 17011371 17011412

IV 17011680 17011718

IV 17019385 17019600

IV 17026063 17026088

IV 17029869 17029894

IV 17030340 17030419

IV 17033608 17033613

IV 17058035 17058089

IV 17058514 17058545

IV 17058711 17058943

IV 17059806 17061560

IV 17061629 17061836

IV 17061899 17062088

IV 17062249 17062804

IV 17063402 17063551

IV 17063739 17063783

IV 17064038 17064054

IV 17064720 17064786

IV 17065113 17065172

IV 17065301 17065341

IV 17065396 17065443

IV 17065608 17065770

IV 17066646 17066649

IV 17066699 17066715

IV 17067771 17067852

IV 17067946 17067947

IV 17068069 17068100

IV 17068862 17068912

IV 17069109 17069444

IV 17069608 17070034

IV 17070229 17070267

IV 17070359 17070547

IV 17070639 17070914

IV 17071232 17071237

IV 17071371 17071398

IV 17071536 17071612

IV 17075505 17075518

IV 17075897 17075916

IV 17076118 17076166

IV 17076481 17076495

IV 17076662 17076699

IV 17093076 17093179

IV 17093283 17093288

IV 17093486 17093612

IV 17096774 17096787

IV 17096840 17096902

IV 17097001 17097177

IV 17101813 17101828

IV 17101971 17101987

IV 17117134 17117146

IV 17126902 17126969

IV 17127252 17127385

IV 17128383 17128678

IV 17128735 17129131

IV 17130706 17130736

IV 17131532 17131809

IV 17131905 17132087

IV 17132181 17132220

IV 17132414 17132837

IV 17133004 17133338

IV 17133535 17133583

IV 17134315 17134344

IV 17134712 17134794

IV 17134997 17135096

IV 17135179 17135308

IV 17135363 17135381

IV 17136174 17136221

IV 17136311 17136576

IV 17136743 17136754

IV 17136931 17136945

IV 17137257 17137263

IV 17137409 17137421

IV 17138131 17138237

IV 17138541 17138542

IV 17138763 17139153

IV 17139213 17139980

IV 17140202 17140209

IV 17140272 17140294

IV 17140380 17140487

IV 17140599 17140720

IV 17140783 17140958

IV 17141043 17141231

IV 17141306 17141334

IV 17141431 17141490

IV 17141574 17141741

IV 17141791 17141821

IV 17141869 17141886

IV 17141997 17142266

IV 17142851 17142862

IV 17147840 17147906

IV 17148551 17148567

IV 17148615 17148681

IV 17148846 17148907

IV 17154160 17154257

IV 17154455 17154458

IV 17155356 17155433

IV 17155526 17155563

IV 17156062 17156093

IV 17156211 17156243

IV 17159306 17159332

IV 17162164 17162177

IV 17162533 17162543

IV 17163402 17163734

IV 17165674 17166003

IV 17167342 17167356

IV 17167415 17167448

IV 17167535 17167556

IV 17167709 17167729

IV 17169010 17169011

IV 17169462 17170659

IV 17177929 17178717

IV 17178796 17179049

IV 17214513 17214742

IV 17214836 17214866

IV 17216795 17216838

IV 17217586 17217603

IV 17218452 17219614

IV 17220647 17220683

IV 17220819 17220850

IV 17222260 17222276

IV 17222370 17222418

IV 17222874 17222887

IV 17236485 17236617

IV 17238110 17238166

IV 17284801 17284814

IV 17285053 17285077

IV 17305522 17305529

IV 17306023 17306089

IV 17306185 17306187

IV 17307507 17307516

IV 17310140 17310189

IV 17311642 17311650

IV 17312482 17312560

IV 17323206 17323307

IV 17325467 17325476

IV 17325606 17325618

IV 17325881 17326008

IV 17376790 17376806

IV 17376887 17376919

IV 17377031 17377096

IV 17388498 17388541

IV 17388687 17388791

IV 17388934 17389245

IV 17389386 17389621

IV 17389693 17390562

IV 17390610 17390620

IV 17390770 17390952

IV 17391047 17391122

IV 17391172 17391247

IV 17391382 17391884

IV 17391923 17391927

IV 17391979 17392136

IV 17392246 17392288

IV 17392436 17392544

IV 17399962 17400055

IV 17407493 17407544

IV 17438902 17439116

IV 17439208 17439272

IV 17439370 17439626

IV 17471090 17471096

IV 17492450 17492457

IV 17492525 17492530

IV 17492653 17492732

IV 17492782 17492906

IV 17493051 17493133

IV 17493270 17493576

IV 17493664 17493829

MtDNA 0 2

MtDNA 13454 13572

MtDNA 13792 13794

V 0 2

V 51 211

V 465 510

V 635 686

V 9971 10047

V 10299 10301

V 19169 19186

V 19387 19444

V 20352 20398

V 20609 20615

V 31979 32029

V 32390 32441

V 51683 51716

V 51865 51910

V 52112 52211

V 52407 52419

V 53808 54065

V 54699 54874

V 64730 64811

V 65181 65231

V 82939 83011

V 91669 91691

V 98468 98474

V 100660 100696

V 100883 100922

V 134251 134257

V 134380 134387

V 152930 153328

V 153422 153454

V 158255 158281

V 161580 161863

V 162092 162111

V 162263 162540

V 168756 168799

V 172543 172550

V 172651 172675

V 172842 172899

V 172989 173105

V 173290 173344

V 173687 173691

V 182083 182644

V 182740 183520

V 188640 188665

V 192080 192135

V 194217 194225

V 194362 194376

V 198361 198442

V 204778 204824

V 207295 207306

V 207590 207674

V 212883 212892

V 212973 212994

V 213373 213401

V 213474 213642

V 213865 213876

V 214530 214543

V 214638 214683

V 214762 214809

V 215341 215356

V 215429 215447

V 222149 222224

V 223421 223484

V 223639 223681

V 223847 223985

V 260845 260857

V 261763 261787

V 261873 261902

V 262067 262068

V 262158 262173

V 262644 262662

V 262755 262764

V 263179 263183

V 263395 263435

V 263634 263648

V 265261 265327

V 265449 265500

V 265661 265665

V 265865 266357

V 266430 266805

V 266900 267153

V 294948 295019

V 299290 299301

V 299499 299509

V 302447 302489

V 306645 306676

V 306976 307013

V 307191 307237

V 308992 309294

V 309377 309683

V 310749 310762

V 313425 313509

V 313661 313691

V 313784 313869

V 313982 314056

V 314202 314209

V 314387 314389

V 314549 314561

V 317035 317039

V 317414 317444

V 318297 318305

V 335259 335261

V 343010 343014

V 351191 351241

V 351404 351463

V 356216 356236

V 356637 356664

V 390352 390364

V 390434 390452

V 390508 390537

V 410331 410405

V 411192 411268

V 413732 413770

V 413863 413869

V 442207 442211

V 458695 458733

V 469125 469220

V 469648 469650

V 470637 470684

V 495348 495351

V 506700 506874

V 573000 573053

V 573145 573155

V 573398 573441

V 574375 574392

V 608085 608125

V 608199 608208

V 608413 608452

V 623579 623582

V 638299 638382

V 651315 651336

V 651440 651458

V 651623 651654

V 658310 658382

V 670499 670514

V 686228 686266

V 686350 686392

V 686520 686568

V 686642 686671

V 690359 690362

V 690473 690485

V 693613 693616

V 695394 695401

V 704732 704815

V 705289 705316

V 705462 705466

V 705557 705672

V 706143 706184

V 706294 706574

V 706698 706843

V 713435 713791

V 714545 714554

V 714744 714798

V 715300 715352

V 716293 716454

V 716901 716920

V 716995 717048

V 717098 717146

V 717543 717573

V 717952 717980

V 718057 718092

V 718345 718530

V 734741 734848

V 737252 737356

V 739480 740291

V 740391 741564

V 765830 771353

V 779328 779390

V 783998 784019

V 788601 788644

V 790893 791189

V 791286 791355

V 797944 798240

V 798394 798396

V 798766 798780

V 799100 799277

V 799568 799696

V 799793 799806

V 806893 807035

V 807516 807611

V 815760 815763

V 819865 820816

V 822160 823111

V 835765 836007

V 836431 836546

V 836640 837243

V 839274 839322

V 839740 839741

V 840219 840462

V 841702 841820

V 841910 842517

V 843177 843190

V 844667 844720

V 845538 845630

V 845719 845734

V 857733 857740

V 859132 859212

V 860819 860825

V 878926 879083

V 885338 885445

V 889768 889849

V 890372 890421

V 892785 892863

V 893300 893436

V 894157 894200

V 906774 906794

V 914818 914826

V 919256 919395

V 924221 924290

V 924838 924911

V 925036 925177

V 925448 925505

V 926924 926937

V 927039 927057

V 927548 927615

V 927745 927785

V 932499 932507

V 935024 935071

V 935207 935242

V 938098 938104

V 941867 941929

V 945478 945526

V 945584 945595

V 953223 953488

V 953542 953741

V 957778 958053

V 958128 958444

V 959088 959135

V 959221 959284

V 959347 959620

V 962094 962136

V 963112 963163

V 963877 963930

V 971094 971616

V 973072 973301

V 973386 973503

V 973575 973622

V 973707 973739

V 973825 973858

V 974007 974149

V 974201 974261

V 974350 974403

V 977051 977266

V 979432 979435

V 980707 980741

V 983484 983498

V 985720 985725

V 987901 988045

V 988102 988204

V 988271 988336

V 988390 988726

V 989646 989654

V 989702 989776

V 989870 989874

V 992211 992528

V 992621 992628

V 992717 992731

V 994853 994880

V 995126 995129

V 998719 998835

V 998940 999187

V 999237 999382

V 1008414 1008441

V 1008531 1008567

V 1015776 1015833

V 1037346 1037483

V 1037572 1037613

V 1053602 1053630

V 1063715 1063719

V 1069959 1070038

V 1070147 1070230

V 1070338 1070351

V 1090799 1090809

V 1094018 1094125

V 1094354 1094382

V 1094496 1094561

V 1097852 1097878

V 1097997 1098062

V 1100032 1100033

V 1100034 1100035

V 1104318 1104326

V 1104752 1105006

V 1105095 1105157

V 1107399 1107492

V 1107634 1107734

V 1107866 1108566

V 1108747 1108774

V 1108826 1108862

V 1109110 1109115

V 1109195 1109234

V 1109317 1109379

V 1109467 1109513

V 1109597 1109608

V 1109798 1109844

V 1109954 1110162

V 1110258 1110325

V 1110407 1110433

V 1110711 1110713

V 1110805 1110976

V 1122953 1122955

V 1136017 1136041

V 1137519 1137690

V 1137801 1137824

V 1137903 1137913

V 1161675 1161680

V 1161769 1161823

V 1161912 1162084

V 1162171 1162184

V 1162310 1162340

V 1162429 1162439

V 1182097 1182165

V 1182260 1182651

V 1183604 1183650

V 1185861 1185936

V 1186026 1186417

V 1189812 1190037

V 1190176 1190187

V 1190274 1190366

V 1190451 1190478

V 1190559 1190795

V 1190896 1190954

V 1191050 1191087

V 1191176 1191313

V 1195001 1195041

V 1195352 1195394

V 1197296 1197580

V 1200065 1200147

V 1201189 1201218

V 1202066 1203847

V 1203916 1204207

V 1204286 1204346

V 1204415 1204531

V 1205165 1205166

V 1205422 1205516

V 1205677 1205773

V 1206430 1206458

V 1206692 1206695

V 1215200 1217489

V 1217613 1217931

V 1218083 1218095

V 1220897 1220901

V 1234670 1234684

V 1246312 1246361

V 1246448 1246729

V 1254313 1254437

V 1254542 1254670

V 1254785 1254936

V 1255061 1255612

V 1256006 1256455

V 1256507 1256977

V 1257056 1257111

V 1257185 1257284

V 1257380 1257673

V 1257737 1257923

V 1257989 1258106

V 1258178 1258581

V 1258670 1258830

V 1259981 1259984

V 1260802 1260855

V 1267677 1267679

V 1273453 1273500

V 1273594 1273774

V 1273869 1273989

V 1274197 1274235

V 1278377 1278523

V 1289129 1289242

V 1304394 1304483

V 1330156 1330252

V 1330389 1330609

V 1330817 1331333

V 1331502 1331510

V 1331602 1331618

V 1331844 1331898

V 1331916 1331922

V 1347192 1347194

V 1370370 1370440

V 1375635 1375640

V 1376158 1376163

V 1378772 1378851

V 1379358 1379361

V 1381988 1381991

V 1386573 1386644

V 1391362 1391375

V 1391492 1391493

V 1393778 1393782

V 1398185 1398247

V 1400761 1400763

V 1402235 1402239

V 1403483 1403506

V 1403575 1403576

V 1403747 1403753

V 1403812 1403816

V 1403908 1404038

V 1404114 1404231

V 1404404 1404623

V 1404768 1404788

V 1408826 1408835

V 1408905 1408911

V 1409264 1409278

V 1409489 1409507

V 1409571 1409581

V 1415554 1415570

V 1457300 1458449

V 1469925 1469926

V 1469927 1469928

V 1470068 1470074

V 1477036 1477046

V 1480066 1480077

V 1490918 1490996

V 1530961 1530994

V 1531040 1531070

V 1537498 1537527

V 1537615 1537706

V 1560367 1560667

V 1560801 1560991

V 1561081 1561301

V 1561451 1561620

V 1561687 1561711

V 1587908 1588131

V 1589627 1589640

V 1589704 1589799

V 1592681 1592728

V 1592799 1592865

V 1592960 1593031

V 1593115 1593185

V 1593248 1593295

V 1593391 1593404

V 1594889 1594917

V 1595057 1595145

V 1595309 1595442

V 1595553 1595576

V 1595671 1595674

V 1598594 1598882

V 1598946 1599105

V 1605418 1605436

V 1605506 1605513

V 1605617 1605699

V 1609205 1609229

V 1609288 1609300

V 1629723 1629767

V 1629980 1630004

V 1638004 1638008

V 1638103 1638403

V 1638732 1638740

V 1638790 1638908

V 1653117 1653122

V 1666235 1666256

V 1666618 1666631

V 1666907 1666940

V 1688223 1688244

V 1688861 1688864

V 1689833 1689850

V 1689979 1689990

V 1691077 1691107

V 1691343 1691348

V 1691631 1691637

V 1693083 1693392

V 1693485 1693497

V 1693616 1693620

V 1693827 1693972

V 1694225 1694250

V 1703657 1703693

V 1704647 1704702

V 1704793 1704862

V 1704967 1705014

V 1705187 1705437

V 1705492 1705524

V 1705620 1705655

V 1705767 1705822

V 1705978 1706011

V 1706161 1706410

V 1708154 1708188

V 1712389 1712418

V 1712541 1712597

V 1712794 1712892

V 1713016 1713072

V 1713128 1713537

V 1713736 1713817

V 1713874 1713883

V 1714018 1714075

V 1714192 1714548

V 1714611 1714628

V 1722909 1722910

V 1727862 1727917

V 1728061 1728089

V 1728211 1728226

V 1728316 1728332

V 1728398 1728435

V 1775912 1775917

V 1776005 1776060

V 1790192 1790212

V 1797871 1797970

V 1813673 1813687

V 1814794 1814903

V 1816533 1816549

V 1825472 1825511

V 1825596 1825609

V 1826109 1826281

V 1826928 1826945

V 1827033 1827038

V 1827238 1827253

V 1827319 1827385

V 1827693 1827777

V 1827835 1827889

V 1828317 1828322

V 1830755 1835802

V 1835893 1835901

V 1836110 1841247

V 1850098 1850168

V 1850595 1850614

V 1862806 1862835

V 1865745 1865746

V 1877220 1877232

V 1877467 1877493

V 1877838 1877843

V 1881555 1881571

V 1891875 1891893

V 1894808 1894886

V 1894981 1894987

V 1899571 1899630

V 1899677 1899804

V 1899900 1899972

V 1900035 1900089

V 1900287 1900371

V 1900488 1900507

V 1900752 1900819

V 1901453 1901521

V 1902030 1902081

V 1904812 1905016

V 1905523 1905525

V 1907979 1908061

V 1908159 1908233

V 1908292 1908338

V 1915616 1915652

V 1915863 1915869

V 1916035 1916047

V 1916192 1916231

V 1916280 1916290

V 1926053 1926283

V 1926474 1926681

V 1954657 1954995

V 1955080 1955130

V 1958877 1958934

V 1970172 1970205

V 1978446 1978490

V 1990402 1990440

V 1993496 1993507

V 1995258 1995273

V 2005812 2005859

V 2006559 2006656

V 2012266 2012311

V 2012748 2012750

V 2013163 2013257

V 2052607 2052636

V 2052764 2052802

V 2054742 2054750

V 2054902 2054979

V 2055494 2055505

V 2057924 2057941

V 2058102 2058137

V 2058808 2058816

V 2063774 2063810

V 2063883 2063948

V 2064112 2064117

V 2065145 2065173

V 2073485 2073493

V 2073586 2073593

V 2076689 2076706

V 2077047 2077126

V 2077253 2077278

V 2077471 2077526

V 2077619 2077634

V 2078732 2078797

V 2079361 2079417

V 2080255 2080263

V 2086112 2086113

V 2088553 2088567

V 2088663 2088690

V 2090556 2090572

V 2092500 2092571

V 2096226 2096232

V 2098095 2098105

V 2102815 2102904

V 2108498 2108839

V 2117326 2117673

V 2134996 2135050

V 2140772 2140832

V 2140900 2141235

V 2141334 2141471

V 2141561 2141693

V 2141779 2141907

V 2141986 2142036

V 2144669 2144778

V 2146597 2146811

V 2161703 2161712

V 2172718 2173875

V 2194555 2194799

V 2194894 2195032

V 2195207 2195347

V 2195438 2195653

V 2236810 2236858

V 2237150 2237173

V 2237513 2237532

V 2237680 2237710

V 2238944 2239153

V 2241598 2241632

V 2241729 2241960

V 2243605 2243656

V 2243951 2243972

V 2246542 2246554

V 2246710 2246739

V 2255324 2255349

V 2255511 2255570

V 2256461 2256577

V 2256736 2256835

V 2267707 2268156
[truncated: 171,498 more chars]
