# Supplementary material for: Evaluating alignment and variant-calling software for mutation identification in C. elegans by whole-genome sequencing
Source: PLoS One. 2017 Mar 23;12(3):e0174446. doi: 10.1371/journal.pone.0174446 (PMC5363872; doi:10.1371/journal.pone.0174446)
Supplement: S5 File — (DOCX) [file pone.0174446.s007.docx]

**SUPPLEMENT FILE S5: BBMAP + FREEBAYES WORKFLOW**

(**note:** requires Linux operating system; assumes knowledge of directory structure/PATHs)

**CONVENTIONS FOR COMMAND-LINE INSTRUCTIONS**

A. Commands are preceded by greater-than sign (>)

B. User-specific variables are shown in *UPPER_CASE_ITALICS*

C. Single-line commands that are too long are split by backslash (\)

(**note**: do not include backslash in command)

**1. Download the following software; install per developer’s instructions**

BBMap: <https://sourceforge.net/projects/bbmap/>

SAMtools/BCFtools: <https://sourceforge.net/projects/samtools/files/samtools/>

FreeBayes: <https://github.com/ekg/freebayes>

**2.** **Download a copy of the reference genome**

(**note:** it is essential to use the identical reference genome for the entire workflow. For example, if you intend to use the UCSC Genome Browser at the end of this pipeline, then download the reference from that source.)

For this study, we downloaded the *C. elegans* reference from WormBase (available at: <ftp://ftp.wormbase.org/pub/wormbase/species/c_elegans/sequence/genomic/c_elegans.PRJNA13758.WS_VERSION.genomic.fa.gz>)

(**note:** optional for quality filtering) The *E. coli* reference is available for download at: <http://bacteria.ensembl.org/Escherichia_coli_str_k_12_substr_mg1655/Info/Index>.

**3. Extract and rename the reference genome**

> gunzip c_elegans.PRJNA13758.*WS_VERSION*.genomic.fa.gz

> mv c_elegans.PRJNA13758.*WS_VERSION*.genomic.fa *WS_VERSION.FA*

**4. Index reference genome for use with SAMtools**

> samtools faidx *WS_VERSION.FA*

**5. Quality filtering of data with BBMap (recommended)**

(**note1**: not utilized in manuscript for simulated reads, but recommended for real data)

(**note2**: first command removes *E. coli* contamination; requires *E. coli* genome in FASTA format)

(**note3**: second command removes adapter contamination; *TRUSEQ.FQ.GZ* included with BBMap)

(**note 4**: third command removes low-quality sequences from reads)

> bbduk.sh in=*RAW_DATA.FASTQ* out=*FILTERED_DATA.FASTQ* ref=*E_COLI.FA* k=31 hdist=1

> bbduk.sh in=*FILTERED_DATA.FASTQ* out=*FILTERED2_DATA.FASTQ* ref=*TRUSEQ.FQ.GZ* \

outktrim=r k=23 hdist=1 mink=11 minlen=25

> bbduk.sh in=*FILTERED2_DATA.FASTQ* out= *SEQUENCE_DATA.FASTQ* qtrim=rl \

trimq=10 minlen=25

**5. Align data to reference genome with BBMap**

(**note 1**: ref=*WS_VERSION.FA* creates index of the reference genome)

(**note 2**: optional flag 'sam=1.3' is used for compatibility with variant caller)

(**note 3**: optional flag ‘ambiguous=toss’ ignores non-unique alignments)

> bbmap.sh ref=*WS_VERSION.FA* sam=1.3 ambiguous=toss in=*SEQUENCE_DATA.FASTQ* \

out=*SEQUENCE_DATA.BAM*

**6. Sort data by chromosome/position with SAMtools**

(**note**: optional flag '-@ 4' specifies the number of threads)

> samtools sort -O bam -o *SEQUENCE_DATA_SORTED.BAM* -T *TEMP* -@ 4 \

*SEQUENCE_DATA.BAM*

**7. Remove duplicate reads from data with SAMtools**

> samtools rmdup -s *SEQUENCE_DATA_SORTED.BAM SEQUENCE_DATA_DEDUP.BAM*

**8. Index data using SAMtools**

> samtools index *SEQUENCE_DATA_DEDUP.BAM*

**9. Perform candidate gene variant calling with FreeBayes**

> freebayes -f *WS_VERSION.FA* *SEQUENCE_DATA_DEDUP.BAM* > *SEQUENCE_DATA.VCF*

**10. Filter for a minimum read depth of three**

(**note:** vcffilter command is located in /freebayes/vcflib/bin directory)

> vcffilter -f "DP > 2" *SEQUENCE_DATA.VCF* > *CANDIDATE_MUTATIONS.VCF*

**11. Perform Hawaiian SNP calling with FreeBayes**

(**note1**: optional, required only when using Hawaiian mapping cross)

(**note2**: optional flags '-F 0.01' for 1% variant calls and '-C 1' supported by at least one read)

> freebayes -f *WS_VERSION.FA* -F 0.01 -C 1 --pooled-continuous \ *SEQUENCE_DATA_DEDUP.BAM* > *SEQUENCE_DATA_F01C1.VCF*

**12. Filter VCF to retain only Hawaiian SNPs with BEDtools**

(**note:** Hawaiian SNPs VCF is available as Supplement Table S2)

> bedtools intersect -a *SEQUENCE_DATA_F01C1.VCF* -b *HAWAIIAN_SNPS.VCF* > \

*SEQUENCE_DATA_HAW.VCF*

**Note:** *SEQUENCE_DATA_HAW.VCF* is used to generate Hawaiian SNP frequency plots

(e.g.,via Galaxy’s CloudMap or locally with R)
